# 5-(3,5-Dinitrophenyl)-1,3,4-oxadiazol-2-amine derivatives, their precursors, and analogues: Synthesis and evaluation of novel highly potent antitubercular agent

**DOI:** 10.1371/journal.pone.0324608

**Published:** 2025-05-29

**Authors:** Václav Pflégr, Jiřina Stolaříková, Galina Karabanovich, Jana Maixnerová, Adrián Pál, Jana Korduláková, Zuzana Šanderová, Michaela Liegertová, Jaroslav Roh, František Trejtnar, Jarmila Vinšová, Martin Krátký

**Affiliations:** 1 Department of Organic and Bioorganic Chemistry, Faculty of Pharmacy in Hradec Králové, Charles University, Hradec Králové, Czech Republic; 2 Laboratory for Mycobacterial Diagnostics and Tuberculosis, Regional Institute of Public Health in Ostrava, Ostrava, Czech Republic; 3 Department of Pharmacology and Toxicology, Faculty of Pharmacy in Hradec Králové, Charles University, Hradec Králové, Czech Republic; 4 Department of Biochemistry, Faculty of Natural Sciences, Comenius University in Bratislava, Bratislava, Slovakia; 5 Centre of Nanomaterials and Biotechnology, Faculty of Science, Jan Evangelista Purkyně University in Ústí nad Labem, Ústí nad Labem, Czech Republic; Kafrelsheikh University Faculty of Pharmacy, EGYPT

## Abstract

Drug resistance is a growing problem for many pathogens, including mycobacteria. Small heterocyclic molecules are among the leading scaffolds for developing potential antimycobacterial agents. Therefore, based on the molecular hybridization approach, we have prepared an extensive series of *N*-substituted 5-(3,5-dinitrophenyl)-1,3,4-oxadiazol-2-amine derivatives. We also investigated their isosteres and acyclic synthetic precursors. The compounds were evaluated for their *in vitro* activity against *Mycobacterium tuberculosis* (*Mtb*) H_37_Rv, a panel of multidrug- and extensively drug-resistant *Mtb* isolates and two nontuberculous mycobacterial strains (NTM; *M. avium* and *M. kansasii*). The ability to inhibit mycobacterial growth was quantified using minimum inhibitory concentration (MIC) values. Many compounds achieved MIC values ≤ 0.03 µM for NTM and *Mtb*, regardless of their resistance profile. The highest activity was associated with oxadiazole and thiadiazole scaffolds with benzylamino or C_5_-C_9_ alkylamino substitution. The experimentally confirmed mechanism of action of these compounds consists of disruption of mycobacterial cell wall biosynthesis *via* inhibition of decaprenylphosphoryl-β-D-ribose 2’-epimerase (DprE1). *In vitro* toxicity evaluation was performed in a hepatocyte model (HepG2), while *in vivo* toxicity was evaluated using *Danio rerio* embryos. These findings identify a promising new chemotype with potent, broad-spectrum and selective antimycobacterial activity, including efficacy against resistant strains, and support its further development as a potential therapeutic candidate.

## Introduction

Tuberculosis (TB) is a collective term for all forms of disease caused by *Mycobacterium tuberculosis* (*Mtb*) complex. Until the SARS-CoV-2 pandemic, TB was the leading cause of death from a single infectious agent, even ranking above HIV/AIDS. TB is an easily transmissible disease. It is curable, yet it remains one of the most common causes of death worldwide [1]. TB is usually treated for a long period (months), and the patient’s adherence to the therapeutic regimen is essential. However, it can cause some discomfort for the patients. Adherence to strict hygiene requirements, inability to attend work, personal isolation, side and adverse effects of administered drug combinations, as well as other restrictions, may not be tolerated by the patients and often result in early termination or discontinuation of treatment. This phenomenon has been implicated in the selection of *Mtb* strains resistant to otherwise highly effective antituberculosis drugs, such as multidrug-resistant (MDR) and extensively drug-resistant (XDR) *Mtb*. MDR-TB is resistant to both isoniazid (INH) and rifampicin (RIF), while XDR-TB consists of additional resistance to any fluoroquinolone (levofloxacin, moxifloxacin) and at least one additional Group A drug (bedaquiline or linezolid) [2]. TB infection, in combination with other diseases (HIV, diabetes mellitus, various lung diseases, *etc.*), can be a severe and life-threatening state [3]. Although prevention plays a crucial role in eradication of TB [4], but given the current situation, there is increasing pressure to develop new compounds with antimycobacterial activity with no cross-resistance to currently used drugs.

In addition to *Mtb*, other mycobacterial strains with significant pathogenic potential are also known. Atypical or nontuberculous mycobacterial (NTM) species are widespread pathogens that cause mycobacterioses, and like *Mtb*, NTMs pose a severe health risk to immunocompromised patients with an increasing and alarming incidence. These infections may or may not be similar to TB symptoms and include cutaneous, pulmonary, and systemic manifestations. For example, *M. avium intracellulare* complex (MAC) or *M. kansasii* often attacks patients with pre-existing lung disease (e.g., cystic fibrosis, bronchiectasis, chronic obstructive pulmonary disease). The symptoms are often non-specific and may overlap with those of the underlying disease. Treatment consists of administering multiple antimycobacterial agents to which the causative strain is sensitive, often rifabutin or ethambutol (EMB), rarely INH due to primary or acquired resistance. Aminoglycoside antibiotics, such as streptomycin (STR), amikacin, or fluoroquinolones, are also used in therapy [5,6].

**Fig 1:**
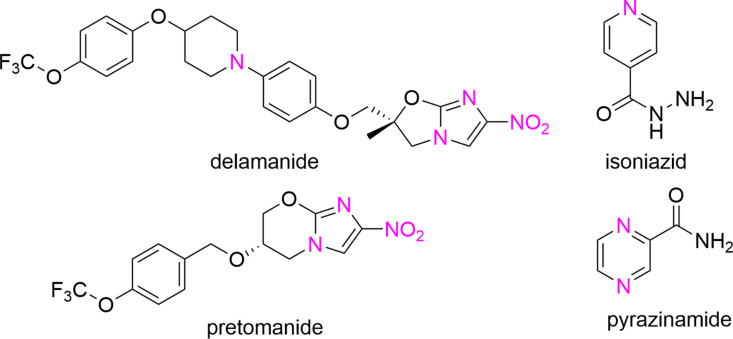
Structures of novel nitroimidazole anti-TB drugs (delamanid, pretomanid), isoniazid and pyrazinamide.

Small heterocyclic molecules have an essential position among the most important antituberculosis agents. The most recent examples are two drugs from the nitroimidazole class, delamanid and pretomanid (Fig 1), approved for the treatment of TB with various resistance profiles (especially for MDR) [7]. Even two essential first-line anti-TB drugs, INH and pyrazinamide (PZA), contain such a nitrogen heterocyclic ring (Fig 1). Among other extensively studied heterocyclic scaffolds with antitubercular activity are 1,2,3-triazoles [8–11].

Oxadiazoles are of particular interest in the design of potential antimicrobial agents, including antimycobacterial agents. Several unique modifications of oxadiazoles, with very promising properties, have been described previously [7,11,12].

**Fig 2:**
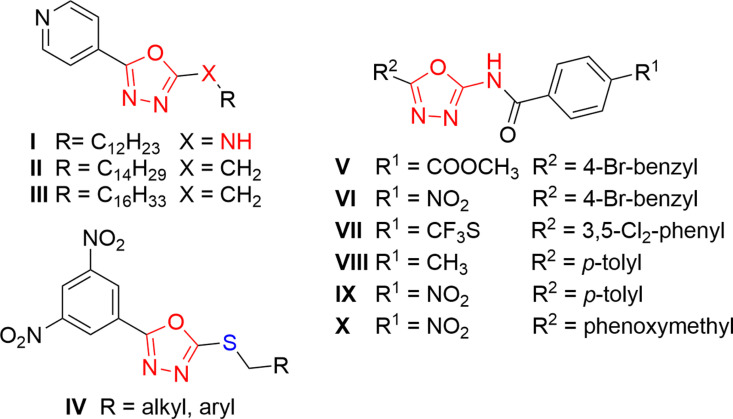
Promising 1,3,4-oxadiazoles with antimicrobial activity.

Some of the investigated oxadiazoles with promising biological activity are shown in Fig 2. Vosátka *et al.* [13] prepared a series of seventeen *N*-alkyl-5-(pyridin-4-yl)-1,3,4-oxadiazol-2-amines to find new compounds with potential antimycobacterial activity. Compound **I** was able to inhibit selected MDR and XDR-TB strains (minimum inhibitory concentrations, MIC, of 4–8 µM), as well as intrinsically INH-resistant *Mycobacterium avium* (MIC = 8–16 µM), without any cross-resistance to commonly used antimycobacterial drugs.

Navarrete-Vazquez *et al.* [14] investigated the synthesis and evaluation of antimycobacterial properties of 4-(5-substituted-1,3,4-oxadiazol-2-yl)pyridine derivatives with MIC values of 0.09–49.69 μM for drug-susceptible strains. Compounds **II** and **III** were able to inhibit polyresistant *Mtb* strains (resistant to various drugs, e.g., INH, STR, and EMB), although only at higher concentrations (≥2.59 μM). However, the authors did not investigate the mechanism of action.

Also, *S*-substituted 1,3,4-oxadiazole-2-thiol derivatives **IV** showed very potent activity against drug-susceptible *Mtb* H_37_Rv, as well as against clinically isolated MDR and XDR strains. Furthermore, no cross-resistance with first- and second-line anti-TB drugs was identified. The MIC values of the best compounds reached 0.03-0.06 µM for both susceptible and resistant strains including NTM. Importantly, they were selective for mycobacteria since no significant activity against several strains of bacteria and fungi was found [15].

Another study [16] reported potent activity of 1,3,4-oxadiazole-2-amines **V** and **VI** against *Salmonella typhi*. A series of *N*-(1,3,4-oxadiazol-2-yl)benzamides represented by compound **VII** containing a trifluoromethylthio moiety was superior to vancomycin against a panel of clinical isolates of *Clostridium difficile* with MIC values as low as 0.007 μM [17]. Li *et al.* [18] investigated antimicrobial properties of 2-acylamino-1,3,4-oxadiazoles. They found that the cyclization of starting acylthiosemicarbazides to the corresponding oxadiazoles **V**–**VII** significantly increased their activity. Compound **VIII** was the most active against *Staphylococcus aureus* with a MIC of 1.56 mg/mL, and derivatives **IX** and **X** were the most active against *Bacillus subtilis* with a MIC of 0.78 mg/mL.

As demonstrated, 1,3,4-oxadiazoles and their 2-amines can be considered key pharmacophores for antimycobacterial activity [19]. Also, isosteric 1,3,4-thiadiazoles are well-known for their antimycobacterial properties [20].

**Fig 3:**
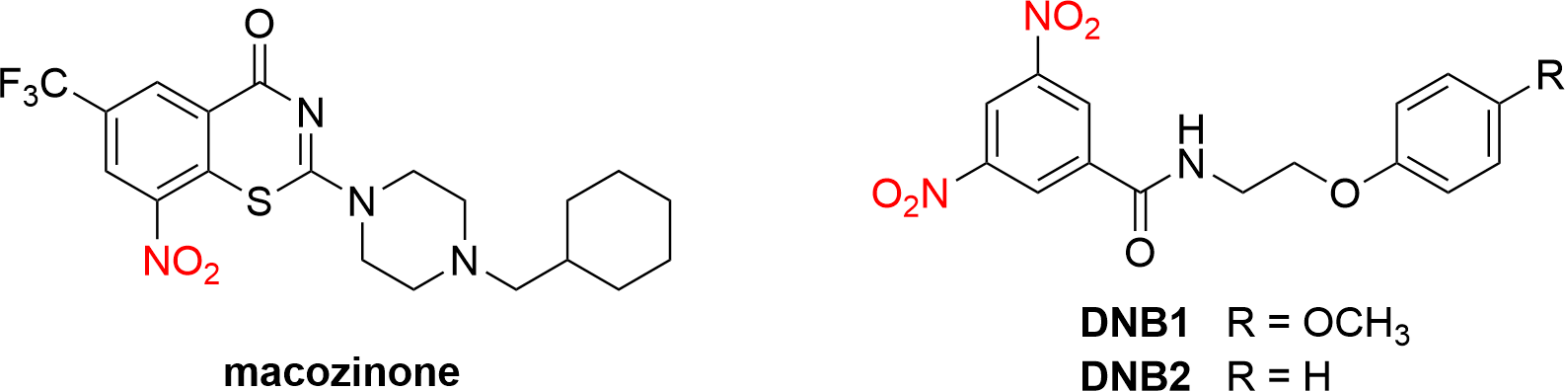
Structures of nitro compounds macozinone, DNB1, and DNB2.

However, favourable anti-TB properties may also be related to the presence of nitro moiety. This has been demonstrated, e.g., for the aforementioned anti-TB drugs in clinical use, delamanid and pretomanid. The presence of nitro groups in positions 3 and 5 of the scaffold **IV** has also been shown to be essential for antimycobacterial properties. The same observation on the necessity of the nitro group has been found, for example, for highly antimycobacterial potent 8-nitrobenzothiazinones. The aryl nitro moiety in macozinone (MIC for *Mtb* of 0.002 µM; Fig 3) is necessary because it is activated *in vivo* within mycobacteria by the flavoenzyme decaprenyl-phosphoribose 2’-epimerase DprE1 to a reactive nitroso form that subsequently binds to the enzyme [21]. Other excellent examples are the DNB1 and DNB2 (Fig 3). Both are highly effective against MDR- and XDR-TB clinical isolates with low MIC values. In addition, DNB1 and DNB2 are associated with low levels of spontaneous resistance. Resistant mutants arise with a similar frequency to that of INH. Importantly, incubation of *Mtb* with DNB1 or DNB2 inhibited the formation of lipoarabinomannan and arabinogalactan due to the inhibition of decaprenyl-phosphoryl-arabinose synthesis catalysed by DprE1 [22]. DprE1 is a validated and vulnerable target for development of antimycobacterial drugs, both covalent and non-covalent inhibitors of this enzyme [23,24]. In addition, many other promising antimycobacterial hits with NO_2_ group(s) have been overviewed in the literature [25].

Based on the facts presented here, we prepared a series of unique compounds that draw inspiration from these structures. We have synthesized molecules that combine 3,5-dinitrophenyl, 1,3,4-oxadiazole-2-amine, and long hydrocarbon/benzyl moieties as potential antimycobacterial agents. In addition, we investigated their isosteres and synthetic precursors.

## Results and discussion

### Synthesis

The starting material for the synthesis of the 1,3,4-oxadiazoles was 3,5-dinitrobenzohydrazide **3**. The most preferred way to obtain hydrazide **3** is its *in-house* preparation by simple hydrazinolysis of the methyl 3,5-dinitrobenzoate **2**. This ester was prepared by a Fischer-Speier esterification of commercially available 3,5-dinitrobenzoic acid **1** by methanol in the presence of a catalytic amount of sulfuric acid. This well-known and reliable method provides satisfactory yield (89%) of ester **2**. In addition, it does not need to be additionally purified (*via* extraction only) and can be used immediately for subsequent reaction. Hydrazide **3** was prepared by hydrazinolysis of the starting ester **2**. The reaction was performed in boiling methanol with hydrazine monohydrate in a moderate excess related to the stoichiometry (2 equivalents). The reaction conversion of ester **2** to hydrazide **3** is quantitative (according to TLC); however, crude product **3** was recrystallized at least once from boiling methanol. The overall yield of product **3** after recrystallization was 72%.

Our oxadiazole-(or thiadiazole)-2-amines decorated with various alkyl chains were prepared by dehydrative cyclization of the corresponding precursors **4a-s**. Starting materials **4a-s** can be derivatives of (thio)semicarbazide and are readily available by reaction of the hydrazide **3** with alkyl iso(thio)cyanates. Most alkyl isocyanates and lauryl isothiocyanate were commercially available. However, longer-chain alkyl isocyanates with an odd number of carbon atoms for synthesizing compounds **4m**, **4o**, and **4q** (tridecyl, pentadecyl, and heptadecyl) were unavailable. Methyl isocyanate was not available as well (nor in solution). Unfortunately, the common synthetical substitute (*N*-succinimidyl-*N*-methylcarbamate, known as MIC substituent) or the generation of isocyanates *in situ* from appropriate amines with triphosgene [26] has not brought satisfactory yields. Preferably, Curtius rearrangement was used to prepare the isocyanates mentioned above. First, the corresponding acyl chloride was prepared by reacting a commonly available and cheap carboxylic acid with thionyl chloride (neat), using a few drops of DMF as a catalyst. These reactions work very well, and the reaction conversion is usually quantitative. Corresponding acyl chloride was dissolved/suspended in anhydrous toluene with a slight stoichiometric excess of sodium azide (1.1 of equivalents) at low temperature (approximately -20°C) under a nitrogen atmosphere. Acyl azides were prepared for 1 hour, then the temperature was raised to 60°C, and the entire amount of acyl azide decomposed to give the corresponding isocyanate. Methyl isocyanate was prepared from commercially available acetyl chloride. The afforded isocyanates were used as a solution, and a calculated amount was added to a boiling solution of the hydrazide **3** in anhydrous acetonitrile. When the starting hydrazide **3** disappeared from TLC (ca. 2 hours), the reaction mixture was cooled to room temperature, and precipitation of the resulting products **4a-s** was observed. The precipitate was filtered off and washed with a small volume of diethyl ether. The compounds **4a-s** of high purity and with high yields were usually obtained. If necessary, the compounds were recrystallized from acetonitrile (especially in synthesizing compounds with a longer alkyl chain) because the products began to precipitate immediately after the addition of the isocyanate (from **4m** to **4r**). Yields ranged from 58–99%.

**Fig 4:**
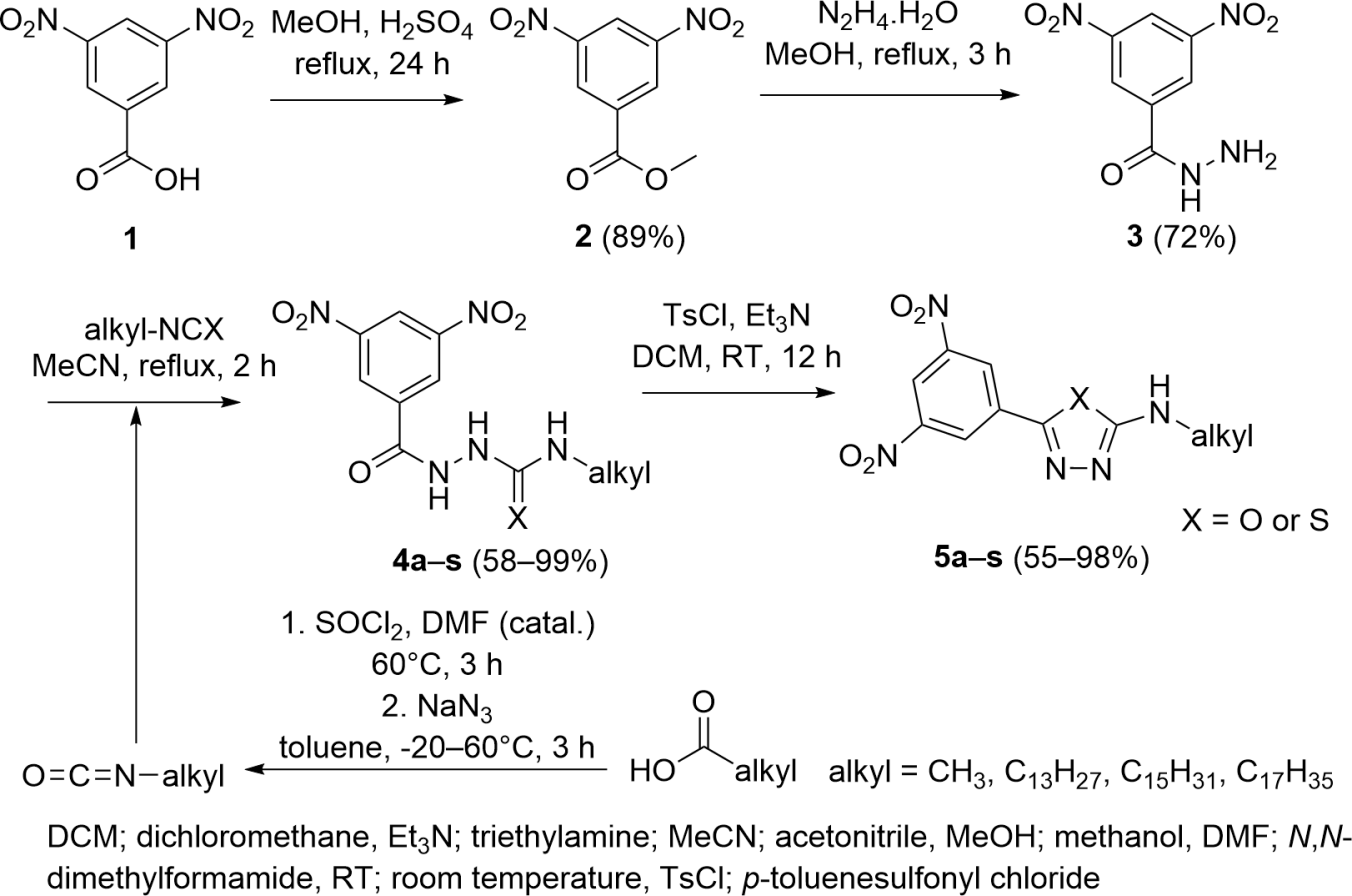
Synthetic scheme of 1,3,4-oxa(thia)diazole-2-amines 5a–s and their precursors 4a–s.

As a proven method [27] for the cyclization of the carboxamides (carbothioamides) **4a-s**, we chose dehydrative cyclization with *p*-toluenesulfonyl chloride in the presence of a base. This rapid and universal process has prepared 1,3,4-oxadiazoles and 1,3,4-thiadiazoles **5a-s** with good regioselectivity in high yields. In the case of the cyclization of 2-(3,5-dinitrobenzoyl)-*N*-dodecylhydrazine-1-carbothioamide **4s**, *N*-alkyl-5-(3,5-dinitrophenyl)-1,3,4-thiadiazol-2-amine **5s** is preferentially formed under chosen conditions (dichloromethane as solvent, triethylamine as base); no desulfurative cyclization was observed at all. In general, the method is very robust and gives very good yields without special requirements. Yields ranged from 55 to 95%. Fig 4 overviews the synthesis of **4a–s** and **5a–s**.

**Fig 5:**
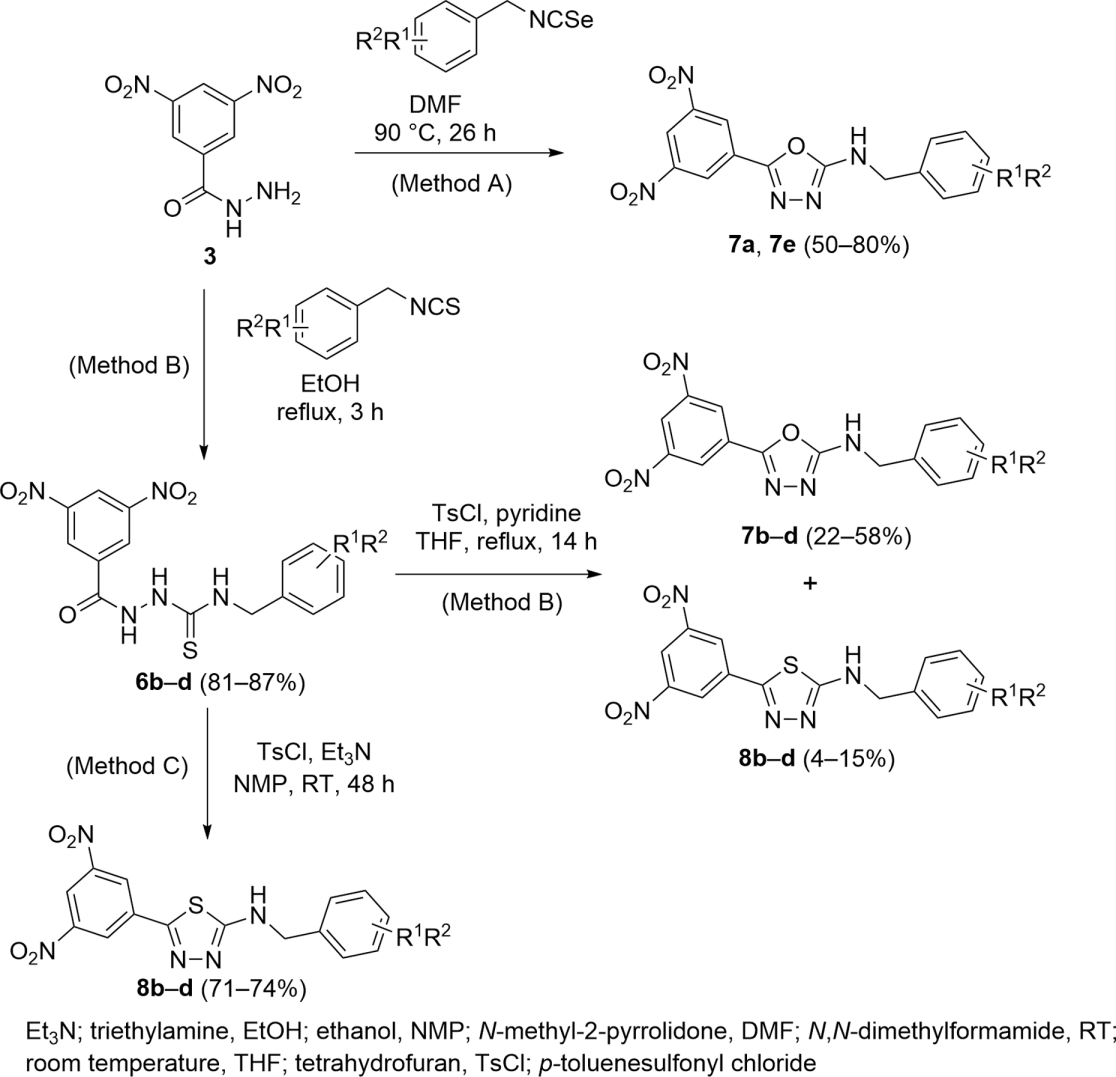
Synthesis of *N*-benzyl analogues 6–8.

The most significant advantage of the synthesis of *N*-benzyl-1,3,4-oxadiazole-2-amines **7** *via* the reaction of 3,5-dinitrobenzohydrazide **3** with isoselenocyanate in DMF is the formation of sole products **7a** and **7e** in rational yields (50–80%). On the other hand, there is a requirement to synthesize corresponding isoselenocyanates starting from *N*-benzylformamide. The alternative partway for synthesising series **7** is the preparation of thiosemicarbazide **6b**–**d** followed by further cyclization with *p*-toluenesulfonyl chloride in the presence of pyridine in THF. However, during this reaction, the formation of 1,3,4-oxadiazoles **7b**–**d** as the main products (22–58%) along with 1,3,4-thiadiazoles **8b**–**d** as the by-products (4–15%) was seen. Unfortunately, products **7** and **8** are hardly separable. The cyclization of **6b**–**d** with *p*-toluenesulfonyl chloride in the presence of triethylamine in *N*-methyl-2-pyrrolidone led to a formation of 1,3,4-thiadiazole-2-amines **8** as the only product in good yields (71–74%). Synthesis of *N*-benzyl analogues **6**–**8** is illustrated in Fig 5.

**Fig 6:**
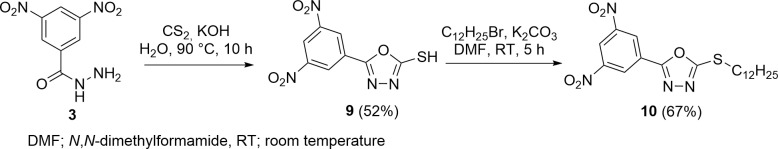
Synthesis of the compound 10.

The synthesis of 5-(3,5-dinitrophenyl)-1,3,4-oxadiazole-2-thiol **9** as a starting material for the synthesis of 2-(3,5-dinitrophenyl)-5-(dodecylthio)-1,3,4-oxadiazole **10** has been described repeatedly. Karabanovich *et al.* [12] prepared thiol **9** by reacting hydrazide **3** with carbon disulfide in aqueous potassium hydroxide. In this work, we used the identical method. As previously, we also observed the formation of undesired by-products; the yield was only 52%. Compound **10** was obtained in good yield (67%) by simple alkylation of the thiol moiety in **9** with a mild excess of dodecyl bromide using potassium carbonate as a base. The synthesis scheme is shown in Fig 6.

### Antimycobacterial activity

First, we evaluated the prepared compounds against three mycobacterial strains: drug-susceptible *Mtb* (331/88, i.e., H_37_Rv) and two NTM, namely polyresistant *Mycobacterium avium* 330/88 and a clinically isolated strain of *Mycobacterium kansasii* 6509/96. INH was used as a reference drug. Activity against *Mtb* was evaluated in the concentration range of 0.03–32 µM, while against NTM, 0.03-1000 µM. Results are summarized in Table 1. All the compounds **4a**–**s** and **5a**–**s** exhibited antimycobacterial properties, especially for *Mtb* and *M. kansasii*, with MIC values starting from ≤ 0.03 µM (**5f**, **5g**, **5h**, **5i**, and **5m**). This contrasts with our expectation, as the high activity of the less lipophilic precursors **4** was surprising.

**Table 1:** Antimycobacterial activity of compounds 4a–4s and 5a–5s.

Code	R	MIC [μM]									
		*Mtb* H_37_Rv			*M. avium* 330/88			*M. kansasii* 6509/96			Clog*P*
		14 days	21 days	14 days		21 days	7 days		14 days	21 days	
**4a**	Methyl	> 32	> 32	10^3^		10^3^	250		500	500	-0.27
**5a**		8	16	500		500	16		32	32	1.17
**4b**	Ethyl	32	> 32	10^3^		10^3^	64		125	125	-0.06
**5b**		8	16	> 10^3^		> 10^3^	16		32	32	1.70
**4c**	Propyl	16	16	10^3^		10^3^	8		16	16	0.4
**5c**		4	4	500*		500*	4		8	16	2.24
**4d**	Butyl	2	2	10^3^		10^3^	2		4	4	0.85
**5d**		0.5	0.5	64		125	1		2	2	2.76
**4e**	Pentyl	**1**	**1**	10^3^		10^3^	**1**		**1**	2	1.31
**5e**		**0.25**	**0.25**	500*		500*	**0.25**		**0.5**	**0.5**	3.29
**4f**	Hexyl	**1**	**1**	250		250	**1**		**1**	**1**	1.77
**5f**		**≤ 0.03**	**≤ 0.03**	**≤ 1**		**≤ 1**	**0.25**		**0.5**	**0.5**	3.82
**4g**	Heptyl	**0.5**	**0.5**	125		250	**1**		**1**	**1**	2.22
**5g**		**≤ 0.03**	**≤ 0.03**	**4**		**8**	**≤ 0.03**		**0.06**	**0.125**	4.35
**4h**	Octyl	**0.25**	**0.5**	125		250	**1**		**1**	**1**	2.68
**5h**		**0.06**	**0.06**	**8**		**16**	**≤ 0.03**		**0.06**	**0.06**	4.88
**4i**	Nonyl	**0.5**	**1**	125		250	**1**		**1**	**1**	3.13
**5i**		**0.06**	**0.06**	**8**		**16**	**≤ 0.03**		**0.06**	**0.06**	5.40
**4j**	Decyl	**1**	**1**	500*		500*	**1**		2	4	3.59
**5j**		0.5	1	**4**		**8**	1		1	1	5.94
**4k**	Undecyl	**1**	**1**	500*		500*	2		4	4	4.05
**5k**		0.5	1	**4**		**4**	1		1	1	6.46
**4l**	Dodecyl	2	2	250*		250*	2		2	4	4.50
**5l**		1	1	**4**		**8**	1		1	1	6.99
**4m**	Tridecyl	2	4	500*		500*	2		2	4	4.96
**5m**		**0.125**	0.5	**8**		**16**	**≤ 0.03**		**0.06**	**0.125**	7.52
**4n**	Tetradecyl	4	8	500*		500*	4		8	8	5.41
**5n**		1	1	**8**		**16**	**0.25**		**0.5**	1	8.05
**4o**	Pentadecyl	8	16	500*		500*	8		8	16	5.87
**5o**		2	2	**8**		**16**	**0.5**		1	2	8.58
**4p**	Hexadecyl	8	16	> 10^3^		> 10^3^	8		16	16	6.33
**5p**		8	8	**8**		**16**	2		4	8	9.11
**4q**	Heptadecyl	8	16	500*		500*	8		16	16	6.87
**5q**		32	> 32	32		64	8		16	32	9.64
**4r**	Octadecyl	8	16	> 10^3^		> 10^3^	8		16	16	7.24
**5r**		> 32	> 32	> 10^3^		> 10^3^	64		64	64	10.17
**4s**	Dodecyl	2	2	16		16	**1**		**1**	**2**	5.01
**5s**		1	1	64		125	1		1	1	7.79
	**INH**	0.5	0.5	> 250		> 250	4		8	8	-0.64

INH; isoniazid, *; determination of MIC value was not possible; we report the concentration at which strain growth was still observed, but at following higher concentration turbidity or precipitate was present.

For series **4** and **5**, the activity against TB strongly depends on the alkyl length. Shorter alkyls yield lower activity, which increases steadily up to the maximum associated with the presence of intermediate-length alkyls. Then, the potency remains high as the length increases and only decreases at the end of the homologous alkyl series. For oxadiazoles **5**, the best growth inhibition is provided by C_6_-C_9_ alkyls (MIC 0.03–0.06 µM), whereas for hydrazinecarboxamides **4**, the length optimum is broader (C_5_-C_11_: 0.25–1 µM). Interestingly, oxadiazoles **5** are consistently more potent than their precursors **4** (up to ≥33.3-fold for the counterparts **4f** and **5f**), with hepta- and octadecyl analogues being exceptions (**4q** vs. **5q**, **4r** vs. **5r**). This structure-activity relationship (SAR) may be a consequence of altered lipophilicity and/or modulation of interactions with target structure. When compared to the antimycobacterial activity of comparator INH, many compounds were significantly more potent *in vitro* (**5e**–**5i**, **5m**; up to ≥ 16.7-fold for the most active oxadiazoles) or fully comparable (**4e**–**k**, **5d**, **5j**–**l**, and **5n**).

Moreover, activity against polyresistant *M. avium* with limited susceptibility to many antimicrobial compounds was also promising. *N*-Hexyl oxadiazole **5f** inhibited this strain at 1 µM, and other compounds were also active at low micromolar concentrations (**5g**–**p**). Two oxadiazoles showed no growth inhibition at 1000 µM (**5b**, **5r**). Among 2-(3,5-dinitrobenzoyl)hydrazine-1-carboxamides **4a**–**r**, activity was significantly lower, with only four compounds exhibiting action, but at higher concentrations of 125–250 µM (substituted by C_6_-C_9_ alkyls: **4f**, **4g**, **4h**, **4i**). Thus, the results confirmed the importance of cyclization for excellent antimycobacterial efficacy. The remaining derivatives were less potent or inactive. Also, for this strain, the active nitro compounds were more effective than INH.

The last strain investigated was *M. kansasii*, a clinical isolate with lower susceptibility to INH. Even for this NTM isolate, all compounds were efficacious with MIC values of ≤ 0.03–500 µM. SAR found is similar to those of *Mtb.* Oxadiazoles **5** are generally more potent than hydrazinecarboxamides **4** (with hepta- and octadecyl compounds **q** and **r** as exceptions), the best activity being associated with the intermediate length of the alkyl (C_7_–C_9_) plus *N*-tridecylated oxadiazole **5m**, but C_5_, C_6_, and C_14_ contributed to potent inhibition too. Focusing on acyclic precursors **4**, the requirement for optimal activity is analogous: from five to nine carbons in the alkyl chain. Compared to INH, most compounds exhibited consistently lower (**4e**–**j**, **5d**–**o**) or the same (**4c**, **4d**, **4k**–**r**, **5c**, **5p**) MIC values.

Also, more lipophilic thio isosteres were effective *in vitro*. Thiocarboxamide **4s** showed similar activity as its oxo analogue **4l** against *Mtb* and *M. kansasii*, but it was many times more active against *M. avium*. Its cyclization to thiadiazole **5s**, designed as a heterocyclic thio analogue of the model structure *I* (Fig 1), retained inhibition of *M. kansasii* and *Mtb*, but *M. avium* is less susceptible, analogously in comparison with oxadiazole isostere **5l**. Against NTM, their activity is superior to INH, for *Mtb* is identical.

We also evaluated *N*-benzyl-1,3,4-oxadiazole-2-amines **7a-e** and their more lipophilic thiadiazole isosteres **8b**–**d** (Table 2). Both series were highly active against all mycobacterial strains with MIC values of ≤ 0.03–4 µM. *Mtb* was the most susceptible strain (≤ 0.03–0.25 µM), closely followed by *M. kansasii* (≤ 0.03–1 µM). *M. avium* was inhibited in the range of 1–4 µM. Importantly, all heterocycles showed lower MIC values than INH for all three strains.

**Table 2:** Antimycobacterial activity of compounds 7a–e, 8b–d, and 10.

Code	R^1^R^2^	MIC [μM]								
		*Mtb* H_37_Rv			*M. avium* 330/88		*M. kansasii* 6509/96			Clog*P*
		14 days	21 days	14 days		21 days	7 days	14 days	21 days	
**7a**	H	**0.125**	0.25	4		4	0.5	1	1	2.63
**7b**	4-OCH_3_	**≤ 0.03**	**≤ 0.03**	**2**		**2**	**≤ 0.03**	**≤ 0.03**	**≤ 0.03**	2.54
**7c**	4-Cl	**0.125**	**0.125**	**1**		**2**	**0.125**	**0.125**	0.25	3.33
**7d**	2,4-Cl_2_	**0.06**	**0.125**	**1**		**1**	**0.06**	**0.125**	**0.125**	4.05
**7e**	4-Br	0.25	0.25	**2**		**4**	0.25	0.5	0.5	3.49
**8b**	4-OCH_3_	**≤ 0.03**	**≤ 0.03**	**4**		**4**	**0.06**	**0.06**	**0.125**	3.34
**8c**	4-Cl	**≤ 0.03**	**≤ 0.03**	**2**		**2**	**0.06**	**0.125**	**0.125**	4.13
**8d**	2,4-Cl_2_	**0.125**	**0.125**	**2**		**2**	0.25	0.5	0.5	4.84
**10**	–	8	8	500		500	2	4	8	6.89
**INH**		0.5	0.5	> 250		> 250	4	8	8	-0.64

INH; isoniazid.

Focusing on SAR, substituting *N*-benzyloxadiazole-2-amine **7a** gave predominantly more active compounds (**7b**–**d**), and 4-bromo derivative **7e** was similarly efficient. The best activity was associated with the small electron-donating methoxy substituent; compound **7b** is the most potent against *M. kansasii* and among the most effective agents against *Mtb.* The introduction of one chlorine atom (**7c**) was also beneficial, but the presence of a second chlorine (**7d**) did not improve antimycobacterial properties, nor did replacement with bromine (**7e**). Isosteric 1,3,4-thiadiazoles **8b**–**d** exhibited similar submicromolar MIC values.

Highly lipophilic thioether **10**, prepared as a thio analogue of oxadiazole-2-amines *I* and **5l** by replacing dodecylamino chain with dodecylthio chain, retained activity against *Mtb*, but for NTM, the results are different. Its activity against *M. avium* is considerably lower; conversely, it led to significantly better inhibition of *M. kansasii*.

### Activity against multidrug-resistant *Mtb* strains

With promising results against drug-susceptible *Mtb*, we selected representatives of the most active compounds from all series and screened them against seven MDR-TB strains (Table 3). We included acyclic hydrazinecarboxamides (**4g**, **4h**, **4i**), carbothioamide **4s**, thiadiazoles **5s**, and **8b**, and oxadiazoles **5e**–**m**, **7b**, and **7d** to cover a broad spectrum of structural motifs.

**Table 3:** MIC of selected compounds against drug-resistant *Mtb* strains.

MIC [µM]														
	*Mtb* Praha 1		*Mtb* Praha 4		*Mtb* Praha 131		*Mtb* 234/2005		*Mtb* 9449/2007		*Mtb* 7357/1998		*Mtb* 8666/2010	
	14 days	21 days	14 days	21 days	14 days	21 days	14 days	21 days	14 days	21 days	14 days	21 days	14 days	21 days
**4g**	1	2	1	1	1	2	1	2	1	1	1	1	1	1
**4h**	1	1	1	1	1	1	1	1	1	1	1	1	1	1
**4i**	1	2	1	1	1	2	1	2	1	1	1	1	1	1
**4s**	2	2	2	2	2	4	2	4	2	2	1	2	1	2
**5d**	1	1	0.5	1	0.5	1	0.5	1	0.5	0.5	0.5	1	0.5	0.5
**5e**	0.125	0.25	0.125	0.5	0.125	0.125	0.125	0.125	0.125	0.125	0.125	0.125	0.125	0.125
**5f**	**≤ 0.03**	**≤ 0.03**	**≤ 0.03**	**≤ 0.03**	**≤ 0.03**	**≤ 0.03**	**≤ 0.03**	**≤ 0.03**	**≤ 0.03**	**≤ 0.03**	**≤ 0.03**	**≤ 0.03**	**≤ 0.03**	**≤ 0.03**
**5g**	**0.06**	**0.06**	**0.06**	**0.06**	**0.06**	**0.06**	**0.06**	**0.06**	**≤ 0.03**	**0.06**	**0.06**	**0.06**	**≤ 0.03**	**0.06**
**5h**	0.125	0.125	0.125	0.125	0.125	0.125	0.125	0.125	**0.06**	0.125	0.125	0.125	**0.06**	0.125
**5i**	0.125	0.125	0.125	0.125	**0.06**	**0.06**	0.125	0.125	**0.06**	**0.06**	0.125	0.125	**0.06**	**0.06**
**5j**	0.25	0.5	0.25	0.5	0.25	0.5	0.25	0.5	0.25	0.5	0.25	0.5	0.25	0.5
**5k**	0.25	0.5	0.25	0.5	0.25	0.5	0.25	0.5	0.25	0.5	0.25	0.5	0.25	0.5
**5l**	0.25	0.5	0.25	0.5	0.25	0.5	0.25	0.5	0.25	0.5	0.5	0.5	0.25	0.5
**5m**	0.125	0.125	**0.06**	0.125	0.125	0.125	0.125	0.125	**0.06**	0.125	0.25	0.25	**0.06**	0.125
**5s**	1	1	1	1	1	1	0.5	1	1	2	0.5	1	0.5	1
**7b**	**≤ 0.03**	**≤ 0.03**	**≤ 0.03**	**≤ 0.03**	**≤ 0.03**	**≤ 0.03**	**≤ 0.03**	**0.06**	**≤ 0.03**	**≤ 0.03**	**≤ 0.03**	**≤ 0.03**	**≤ 0.03**	**≤ 0.03**
**7d**	0.125	0.125	**0.06**	0.125	0.125	0.125	**0.06**	0.125	**0.06**	0.125	0.125	0.125	**0.06**	0.125
**8b**	**≤ 0.03**	**≤ 0.03**	**≤ 0.03**	**≤ 0.03**	**≤ 0.03**	**≤ 0.03**	**≤ 0.03**	**≤ 0.03**	**≤ 0.03**	**≤ 0.03**	**≤ 0.03**	**≤ 0.03**	**≤ 0.03**	**≤ 0.03**

*Mtb* Praha 1; resistant to STM, INH, EMB, RIF, CFZ. *Mtb* Praha 4; resistant to STM, INH, EMB, RIF, OFX, CFZ. *Mtb*. Praha 131; resistant to STM, INH, EMB, RIF, OFX, GEN, AMK. *Mtb* 9449/2007; resistant to STM, INH, RIF. *Mtb.* 234/2005; resistant to STM, INH, EMB, RIF. *Mtb* 7357/1998; resistant to STM, INH, EMB, RIF, OFX. *Mtb* 8666/2010; resistant to STM, INH, EMB, RIF, OFX, CFZ.

STM: streptomycin, INH: isoniazid, EMB: ethambutol, RIF: rifampicin, OFX: ofloxacin, GEN: gentamicin, CFZ: clofazimine, AMK: amikacin.

All mycobacterial strains were resistant to INH and rifamycins (i.e., MDR-TB) plus STM; additional resistance was present in some strains (EMB, ofloxacin, clofazimine, gentamicin, and amikacin). Clinical isolate Praha 131 was resistant to INH, rifamycins, STM, EMB, ofloxacin, gentamicin, and amikacin, i.e., it is extensively drug-resistant (XDR) strain according to previous WHO definition.

Noteworthy, all the derivatives showed uniform inhibition of all MDR-TB strains independently on resistance patterns. Their MIC values are identical or like those obtained for fully susceptible *Mtb* 331/88 (H_37_Rv). The best potency, corresponding to the susceptible strain, was found for oxadiazoles **5f**, **5g**, **7b**, and thiadiazole **8b**. Taken together, this indicates an absence of any cross-resistance to clinically used antitubercular drugs and a different mechanism of action. Based on our best knowledge, we present the activity of acyclic hydrazinecarboxamides and carbothioamide against MDR-TB for the first time.

### Determination of mechanism of action

Considering the activity against MDR-TB, the structural similarity with known DprE1 inhibitors, and the presence of 3,5-dinitrophenyl moiety, we decided to investigate the mechanism of action in this way. We selected the most potent antitubercular hydrazinecarboxamide **4h**, oxadiazole **5g**, carbothioamide **4s**, and thiadiazole **5s**.

First, we analysed the MIC of these compounds against *Mtb* H_37_Ra strains, which overproduce DprE2 or DprE1/DprE2 complex. In principle, a strain overproducing protein proposed target DprE1 should be more resistant to the inhibitors due to higher content of the inhibited protein, i.e., showing significantly higher MIC values.

Table 4 summarizes MIC data for various H_37_Ra strains, i.e., those without inserted additional genes carrying an empty vector (pVV2), overproducing DprE2 (pVV2-*dprE2*) and DprE1/DprE2 (pVV2-*dprE1*/*dprE2*). The strain of *Mtb* H_37_Ra overproducing DprE1/DprE2 showed a substantially higher resistance rate to all four compounds tested when compared to strain with only an empty vector and the DprE2 overproducer. This suggests that DprE1 is a real target, and our compounds interfere with the biosynthesis of mycobacterial cell wall.

**Table 4:** Analysis of susceptibility of *Mtb* H_37_Ra overproducing DprE2 or DprE1/DprE2 complex to the investigated compounds.

*Mtb* H_37_Ra strains	MIC [μM]			
	4h	4s	5g	5s
**pVV2**	< 0.5	2	< 0.06	< 0.1
**pVV2-*dprE2***	< 0.5	2	< 0.06	< 0.1
**pVV2-*dprE1*/*dprE2***	> 2.5	> 4	> 0.3	0.5

*M. tuberculosis* H_37_Ra: pVV2 strain with no recombinant enzyme overproduction; pVV2-*dprE2*: strain overproducing DprE2; pVV2-*dprE1/dprE2*: strain overproducing DprE1 and DprE2.

**Fig 7:**
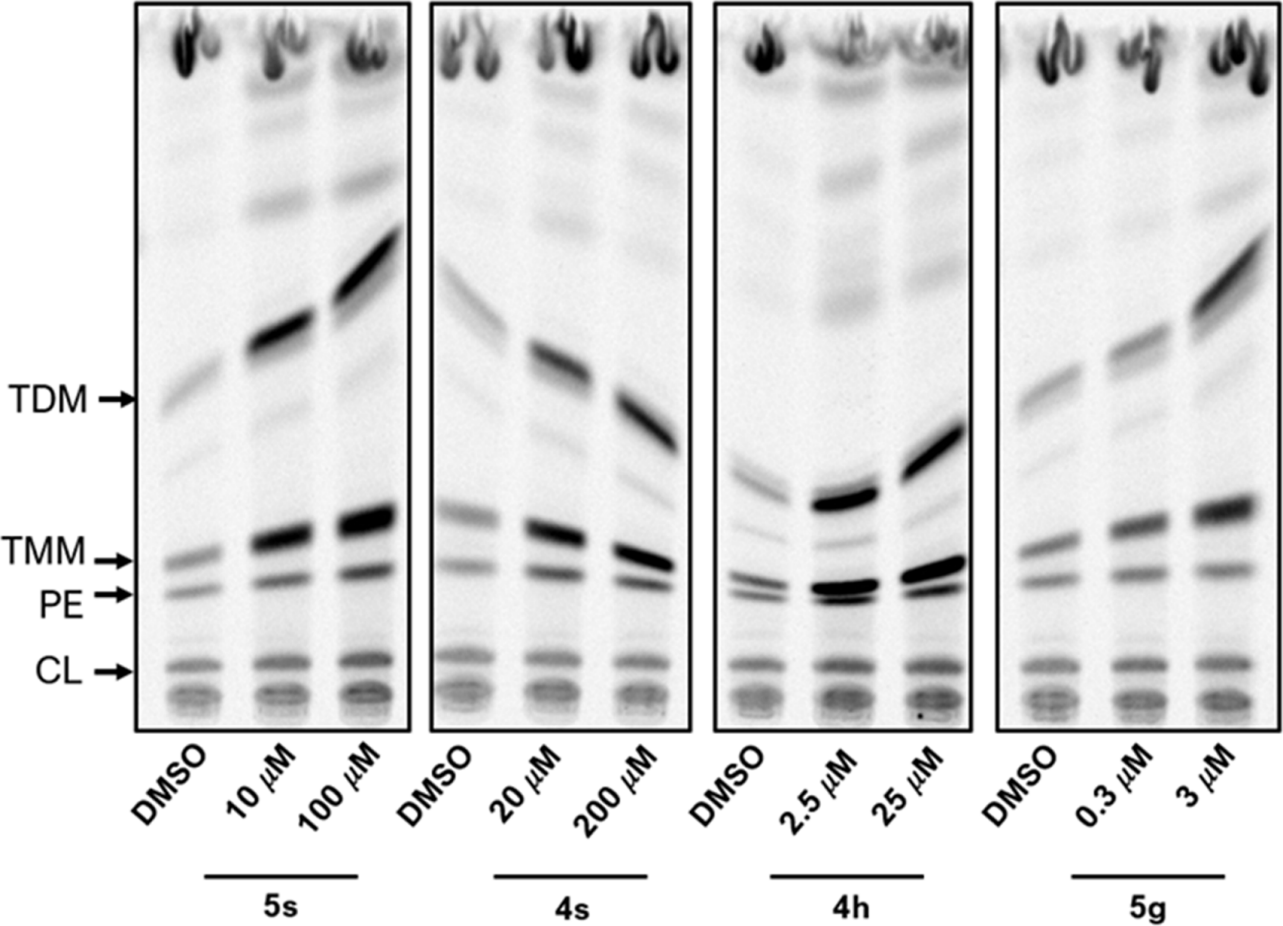
Autoradiogram of TLC analysis of lipids from ^14^C labeled cells of *Mtb* H_37_Rv treated with tested compounds or DMSO. Lipids were separated in chloroform/methanol/water (20:4:0.5). TDM: trehalose dimycolates; TMM: trehalose monomycolates; PE: phosphatidylethanolamine; CL: cardiolipin.

The inhibition of DprE1 activity in the mycobacterial cells leads to the lack of arabinan chains in the cell wall core, which serve as attachment sites for mycolic acids. Mycolic acids are thus accumulated as trehalose monomycolates (TMM) and trehalose dimycolates (TDM). This typical phenotype was described for several DprE1 inhibitors [28,29]. To verify whether the studied compounds target DprE1, we investigated the lipid profiles of mycobacterial cells treated with these inhibitors. *Mtb* H_37_Rv cells were grown in the presence of two different concentrations of **4h**, **4s**, **5g**, and **5s** (corresponding to 10× and 100 × their MIC) and metabolically labeled with ^14^C acetate to monitor the synthesis of lipids. Lipid analysis revealed that the treatment with all of the tested compounds resulted specifically in the accumulation of TMM and TDM (Fig 7) when compared to untreated cells. This finding confirms targeting the proposed epimerase DprE1.

It should be noted that several oxadiazoles, such as **5f** or **5g**, showed excellent activity against NTM strain *M. avium*, which is naturally resistant to nitro group-dependent covalent DprE1 inhibitors like benzothiazinones. Natural resistance of *M. avium* is caused by the replacement of crucial cysteine 387 residue by alanine in its DprE1 enzyme [30]. Therefore, we cannot completely exclude that the antimycobacterial effectiveness of some series **5**, **7**, and **8** compounds is supported by other effects beyond the DprE1 inhibition.

### Toxicity evaluation

#### *In vitro* cytotoxicity.

Hepatic tissue is one of the frequent targets of toxicity and side effects of clinically used antitubercular drugs. The incidence of anti-TB drug-related hepatotoxicity has been reported in 5–28% of treated patients. For example, INH, RIF, PZA, and some fluoroquinolones are metabolized mainly in liver tissue, causing potentially harmful effects [31]. That is why hepatotoxicity assessment of potential antimycobacterial agents is particularly interesting, especially if they are to be given long-term.

Therefore, we evaluated the cytotoxicity of selected potent derivatives *in vitro* on human hepatocellular carcinoma cells (HepG2). This cell line is a convenient and often used model for hepatotoxicity in early drug development. Our study used an MTT assay to determine IC_50_ values and selectivity. IC_50_ is an inhibitory concentration, which reduces the viability of the cell population to 50% from the maximal viability. In living cells, tetrazolium dye MTT is reduced to formazan, which is determined colorimetrically. The reduction rate is related to the availability of NAD(P)H. A drop in their cell levels led to a lower production of formazan. SI was defined as a ratio of IC_50_ to MIC. Based on an analogy with the therapeutic index, a SI value greater than 10 indicates an acceptable level of toxicity (Table 5).

**Table 5:** Cytotoxicity of selected compounds on HepG2 cell line.

Code	IC_50_ [µM]	The range of concentrations tested	SI for *Mtb* 331/88	SI for *M. kansasii* 6509/96
**4d**	197.8	1–500	98.9	49.5
**4e**	133.6	1–500	133.6	66.8
**4f**	119.0	1–500	119.0	119.0
**4g**	116.8	1–500	233.6	116.8
**4h**	136.4	1–500	272.8	136.4
**4i**	124.8	1–500	124.8	124.8
**4j**	141.0	1–500	141.0	35.3
**4k**	99.87	1–500	99.9	25.0
**4l**	165.8	1–500	82.9	41.5
**4m**	162.6	1–500	40.7	40.7
**4n**	207.1	1–500	25.9	25.9
**4o**	82.49	1–500	5.2	5.2
**5d**	> 250	1–250	> 500	> 125
**5e**	> 250	1–250	> 1000	> 500
**5f**	> 100	1–100	> 3,333	> 200
**5g**	> 100	1–100	> 3,333	> 800
**5h**	> 100	1–100	> 1,667	> 1,667
**5i**	> 100	1–100	> 1,667	> 1,667
**5j**	> 100	1–100	> 100	> 100
**5k**	> 50	1–50	> 50	> 50
**5l**	> 50	1–50	> 50	> 50
**5m**	> 50	1–50	> 100	> 400
**5n**	> 50	1–50	> 50	> 50
**5o**	> 50	1–50	> 25	> 25
**INH**	> 1000	1–1000	> 1000	≥ 62.5

INH: isoniazid

Table 5 compares the cytotoxic effect of the tested compounds in the HepG2 cell line using the commercial CellTiter 96 kit. The determination was complicated by poor solubility that prevented evaluation at high concentrations. As expected from the chemical structure and lipophilicity, the acyclic precursors **4** were more soluble than their heterocyclic analogues **5**, and the solubility decreased with the elongation of the alkyl chain.

Fortunately, all oxadiazoles **5** showed no toxicity at the highest available concentration, providing excellent selectivity for *Mtb* (up to > 3,333), *M. kansasii* (up to > 1,667; Table 5), and also against less susceptible *M. avium* (e.g., > 100 for **5f**). This is a consequence of their low toxicity.

The hydrazinecarboxamides **4** showed mild cytotoxicity for HepG2 cells with IC_50_ values of 82.49-207.1 µM, suggesting relatively low differences in cytotoxicity. Only two compounds showed IC_50_ below 100 µM (undecyl and pentadecyl derivatives **4k** and **4o**). Interestingly, derivatives with an even number of carbons in the alkyl chain were more toxic than their lower homologues with an odd number of carbons. Because these precursors **4** were generally somewhat less active, their selectivity is lower than that of oxadiazoles **5**, but, except for **4o**, still sufficient. SI for *Mtb* and *M. kansasii* ranged from 25.9 to 272.8 and 25.9 to 136.4, respectively, favouring octyl and nonyl derivatives **4h** and **4i**.

In summary, the dinitro compounds are selective for *Mtb* and NTM.

### *In vivo* toxicity fish embryo test (FET)

*In vivo* toxicity of **5g** and **4h** and analogous compounds could be influenced by several factors, such as nitro moieties in the structure. It could be a limiting factor for future medicinal applications. The chosen test may also serve well to estimate the effect of the emission of compounds in the environment. We decided to inspect the *in vivo* toxicity of two best-evaluated compounds: one precursor **4h** and one final compound **5g** (the choice was based on the results of antimycobacterial screening) using *Danio rerio* embryos as an experimental model to help us define the potential health and environmental risks associated with their use in tuberculosis treatment. *D. rerio* is a suitable model organism due to the similarities in anatomy and physiology to other vertebrates, short generation time, and external embryonic development [32–34]. It is often used in (eco)toxicological studies [35–37], enabling the expansion of the interpretation of *in vivo* toxicity data into an environmental context. The fish embryo test (FET) follows modified guidelines of the OECD Test No. 236 [38] and serves as a relatively cheap and easy tool for *in vivo* toxicity evaluation [39–41].

**Fig 8:**
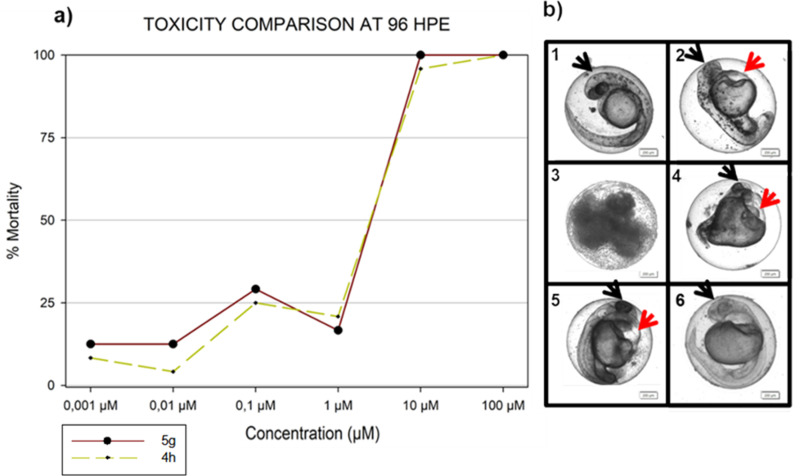
FET results for embryos exposed to 5g and 4h. a) Toxicity comparison of **5g** and **4h** at the end of the 96 hours. The graph shows dose-response curves for the 96-hour time point. *Note: The absence of error bars means a single FET experiment with 24 embryos per concentration as individual replicates.* b) Representative micrographs of toxicity endpoints in our analysis. The exposure to individual concentrations over the whole 96-hour causes morphological effects. **b1**) Untreated control embryo; **b2**) Pericardial oedema; **b3**) Embryo coagulation; **b4**) Embryo showing severe developmental effect/head and tail deformation; **b5**) Oedema of yolk sac; **b6**) Growth retardation and lack of skin pigmentation. Black arrows show the head, and red arrows show observed lethal signs. Embryos were recorded at 72 hours hpe. Scale-bar = 200 µm.

FET experiments were focused on intact embryos following the OECD standards (OECD 2013). The *in vivo* toxicity of both compounds **5g** and **4h**, was inspected for the concentration range of 0.001–100 µM. In the standard 96-hour exposure period, the embryos showed a concentration- and time-dependent response to **5g** and **4h** exposure (Fig 8a). At the end of the 96 hours, the concentration of 100 µM induced 100% lethality in all performed FET tests. The observed lethal endpoints for **5g** were represented mainly by pericardial oedema at lower concentrations and by growth retardation at higher concentrations. For **4h**, the most frequently observed endpoints were embryo coagulation and growth retardation (involving the lack of skin pigmentation and melanocyte formation).

Observed morphological abnormalities included: embryo coagulation (> 25% for **5g** and > 35% for **4h** of all observed endpoints during the whole exposure period), developmental abnormalities/malformations of the head and tail region, growth retardation, lack of body movement or missing heartbeat, and pericardial oedema. In most cases, morphological abnormalities were combined (i.e., body malformations with heart oedema and/or retarded growth), producing severe morphological defects (Fig 8b). Exposure to 10 and 100 µM concentrations was sufficient to induce 100% mortality as early as 48 h for both compounds. This is the most critical and sensitive period of embryonic development when organogenesis occurs [35,42]. Rapid cell proliferation and organ differentiation are toxicity-prone, leading to the most severe structural defects. In most cases, the hatching success of unaffected embryos (Fig 8b) did not differ significantly from negative control. Both **5g** and **4h** seem to have similar toxicity levels with no significant mortality at the concentration of 1 µM (identical to low submicromolar concentrations of **4h** and **5g**) with increasing mortality rate over this concentration.

The mortality rate in negative controls was 4.17% (i.e., one embryo) in all four cases for pure E3 medium and E3 + 0.05% DMSO with pericardial oedema and/or coagulation. The test is valid if the mortality rate is no more than 10% after 96 hours.

It is complicated to compare FET results for various substances within the literature due to varying experimental protocols and a lack of data. No comparable zebrafish study is available for any 1,3,4-oxadiazol-2-amine and hydrazinecarboxamide-based compounds.

Here, we report, for the first time, *in vivo* toxicity data for **5g** and **4h**. Both compounds show relatively low *in vivo* toxicity and environmental risk if we consider possible applications as an antitubercular agent.

## Materials and methods

### Chemistry

#### General.

All chemicals for synthesis (starting materials, solvents) and analysis were obtained from Merck KGaA (Darmstadt, Germany), Acros Organics BVBA (Geel, Belgium), VWR International s. r. o. (Stříbrná Skalice, Czech Republic) Penta Chemicals Unlimited (Prague, Czech Republic) or Lach-Ner (Neratovice, Czech Republic). They were used as received.

The structural identity of the prepared substances was confirmed by ^1^H NMR and ^13^CNMR spectroscopy analyses. NMR spectra (600 MHz for ^1^H and 151 MHz for ^13^C; B_0_ ≈ 14.1 T, alternatively 500 MHz for ^1^H and 126 MHz for ^13^C; B_0_ ≈ 11.7 T or 300 MHz for ^1^H and 75 MHz for ^13^C; B_0_ ≈ 7.0 T) were measured in DMSO-*d*_*6*_ or acetone-*d*_*6*_ as solvents (%_D_ ≥ 99.8, without TMS) at ambient temperature (20–25°C) or 50°C by a JEOL JNM-ECZ 600R (JEOL Ltd., Tokyo, Japan), Varian VNMR S500 instrument (Varian Comp. Palo Alto, CA, USA) or Varian Mercury Vx BB 300 (Varian Comp. Palo Alto, CA, USA). Magnetic presaturation of solvent was not used, residual signal of the non-deuterated solvents was left unaffected in the spectrum for further referencing. Chemical shift values (*δ*) were reported in parts per million (ppm), and spectra were referenced internally to tetramethylsilane as standard by the residual signal of the protic solvent (DMSO-*d*_*6*_: 2.50 for ^1^H, 39.70 for ^13^C and acetone-*d*_*6*_: 2.09 for ^1^H, 30.60 and 205.87 for ^13^C). The coupling constants (*J*) are given in Hz. The concentration of analytical samples for NMR spectroscopy was 2.5% m/V. The purity of all desired compounds was 95% or higher and was measured by elemental analysis using a Vario MICRO Cube element analyzer (Elementar Analysensysteme, Hanau, Germany). Calculated and found values are given as percentages. Melting points were recorded using a Büchi B-545 apparatus (BÜCHI Labortechnik AG, Flawil, Switzerland) with no corrections. Infrared spectra were recorded by a Nicolet 6700 FT-IR spectrometer (Thermo Fisher Scientific, Waltham, MA, USA) in the 600–4,000 cm^-1^ range. ATR (Ge) technique of measuring was used. Retention factors (R*f*) were determined by thin-layer chromatography (TLC). TLC was performed on ALUGRAM SIL G/UV_254_ aluminium plates (Macherey-Nagel GmbH, Düren, Germany) coated with a 0.2 mm silica gel layer (60A, with a fluorescent indicator for 254 nm). Various solvent mixtures were used for elution, most often a mixture of dichloromethane (DCM) and methanol (93:7 V/V; S1) or a mixture of dichloromethane and methanol (97:3 V/V; S2). Avantor Silica Gel with particle size 40–60 µm was used for column chromatography without prior activation. The calculated log*P* values (Clog*P*), which are the logarithms of the partition coefficients for octan-1-ol/water, were calculated, and the reaction schemes were drawn using the ChemDraw Professional 20.0 program (PerkinElmer Inc., MA, USA).

#### Synthesis.

Synthesis of methyl 3,5-dinitrobenzoate [43] **2**

3,5-Dinitrobenzoic acid **1** (12.00 g, 56.58 mmol) was placed into the round bottom flask and dissolved in methanol (200 mL), then concentrated sulfuric acid (10.0 mL) was slowly added with stirring. The reaction mixture was heated to reflux for 24 hours. The solvent was evaporated under reduced pressure, and the crude product was suspended in water and extracted with ethyl acetate (200 mL). The organic phase was extracted with a 10% sodium bicarbonate solution (2 × 200 mL) and brine (200 mL). The organic phase was dried by standing over anhydrous sodium sulfate. Methyl 3,5-dinitrobenzoate **2** was obtained as a white solid and used immediately for the following reaction without further purification. The yield was 11.40 g, 89%, mp 110–111°C. Elemental analysis for C_8_H_6_N_2_O_6_ (226.14); calculated C, 42.49; H, 2.67; N, 12.39, found: C, 42.58; H, 2.55; N, 12.30. R_*f*_: 0.7 (S1). The measured characteristics and spectra were consistent with the literature [44].

Synthesis of 3,5-dinitrobenzohydrazide **3**

Methyl 3,5-dinitrobenzoate **2** (10.00 g, 44.22 mmol) was dissolved in 100 mL of methanol. Hydrazine monohydrate 65% (4.23 mL, 88.44 mmol, 2 eq.) was added in one portion. The reaction mixture was heated to reflux for 3 hours. The progress of the reaction was confirmed by thin-layer chromatography. The mixture S2 was used as an eluent. The solvent was partially evaporated under reduced pressure, and the product was allowed to crystallize at 8°C for 1 hour. The crude 3,5-dinitrobenzohydrazide **3** was filtered off and then recrystallized from boiling methanol. The yield was 7.20 g, 72% (yellow crystalline solid), mp 153–155°C. Elemental analysis for C_7_H_6_N_4_O_5_ (226.15); calculated C, 37.18; H, 2.67; N, 24.77, found: C, 37.30; H, 2.58; N, 24.86. R_*f*_: 0.3 (S1). The measured characteristics were consistent with the literature [12].

Synthesis of semicarbazides **4a–r** and thiosemicarbazide **4s**

*N*-Alkyl-2-(3,5-dinitrobenzoyl)hydrazine-1-carboxamides **4a–r** and *N*-dodecyl-2-(3,5-dinitrobenzoyl)hydrazine-1-carbothioamide **4s** were prepared by reacting the 3,5-dinitrobenzohydrazide **3** with the corresponding iso(thio)cyanates. Methyl, tridecyl, pentadecyl, and heptadecyl isocyanates were prepared in-house by Curtius rearrangement, while the remaining isocyanates and dodecyl isothiocyanate were purchased.

To a suspension of sodium azide (6.9 mmol, 450 mg; 1.1 eq.) in 6.5 mL of anhydrous toluene at -20°C was added appropriate acyl chloride dropwise (6.3 mmol; 1 eq.), the mixture was allowed to react for 1 hour. The mixture was heated to 60°C under a nitrogen atmosphere for 6 hours and cooled to room temperature. The supernatant was decanted and immediately used as 1.0 M alkyl isocyanate solution in toluene [45].

1 mmol of 3,5-dinitrobenzohydrazide **3** (226 mg) was dissolved in 30 mL of anhydrous acetonitrile under a nitrogen atmosphere. Then, 1.1 mmol of the appropriate isocyanate or isothiocyanate (alternatively, a solution thereof in toluene) was added in one portion. The reaction mixture was heated to reflux for 2 hours, and satisfactory conversion was monitored by thin-layer chromatography; a mixture S2 was used as the eluent. The reaction mixture was allowed to crystallize at 8°C for 1 hour, then the product was filtered off, washed with a small volume of diethyl ether, and, if necessary, product **4** was purified by recrystallization from boiling acetonitrile.

Synthesis of 1,3,4-oxadiazoles **5a–r** and 1,3,4-thiadiazole **5s**

0.5 mmol of *N*-alkyl-2-(3,5-dinitrobenzoyl)hydrazine-1-carboxamide **4a–r** or *N*-dodecyl-2-(3,5-dinitrobenzoyl)hydrazine-1-carbothioamide **4s** was suspended in 30 mL of dichloromethane with stirring and five equivalents of triethylamine (2.5 mmol, 348 µL) were added dropwise. Then, three equivalents of *p*-toluenesulfonyl chloride (1.5 mmol, 287 mg) were added in one portion. The mixture was allowed to react at room temperature for 12 hours (or to a satisfactory conversion of the starting material). The reaction progress was confirmed by thin-layer chromatography. The S2 mixture was used as an eluent. The solvent was evaporated under reduced pressure, and crude product **5** was purified by column chromatography, using a mixture of dichloromethane and methanol (99:1 V/V) as eluent.

Synthesis of *N*-benzyl-1,3,4-oxadiazole-2-amines **7a–e**

Method A

1 mmol of 3,5-dinitrobenzohydrazide **3** (226 mg) and 1 mmol of appropriate benzyl isoselenocyanate were dissolved in 10 mL of *N*,*N*-dimethylformamide (DMF). The reaction mixture was heated to 90°C for 24 hours. The solid phase was filtered off and washed with 10 mL of ethyl acetate. The solvent was evaporated under reduced pressure to dryness, and the crude product **7a** or **7e** was purified by column chromatography using a mixture of *n*-hexane/ethyl acetate (4:1 V/V) as eluent [46].

Isoselenocyanates were prepared according to Fernandez-Bolanos *et al*. [47]. However, the synthesis of 4-bromobenzyl isoselenocyanate is not described in the literature. 4-Bromobenzyl isoselenocyanate was prepared using a well-described method [24]. Triphosgene (3.71 mmol, 1.1 g) as a solution in 10 mL dichloromethane was added to the refluxing solution of *N*-(4-bromobenzyl)formamide (7 mmol, 1.5 g), and triethylamine (30 mmol, 3.0 g, 4.2 mL) in 40 mL dichloromethane. The reaction mixture was heated for 2 hours until the spot of *N*-(4-bromobenzyl)formamide disappeared (TLC). Then, selenium powder (14 mmol, 1.11 g) was added, and the reaction mixture was refluxed for 4 hours. After completion, residual selenium was filtered off and washed with ethyl acetate (20 mL), the organic solvent was evaporated under recused pressure, and the product was purified by column chromatography (mobile phase: *n*-hexane/ethyl acetate, 30:1 V/V). 4-Bromobenzyl isoselenocyanate was obtained as a light brown solid in 35% yield. ^1^H NMR (300 MHz, CDCl_3_) δ 7.53 (2H, d, *J* = 8.4 Hz, H3, H5), 7.20 (2H, d, *J* = 8.1 Hz, H2, H6), 4.76 (2H, s, CH_2_). ^13^C NMR (75 MHz, CDCl_3_) δ 132.22, 131.92, 128.53, 122.70, 48.42.

Method B

*N*-Benzyl-2-(3,5-dinitrobenzoyl)hydrazine-1-carbothioamides **6b**, **6c**, and **6d** were prepared by reaction of 1 mmol of 3,5-dinitrobenzohydrazide **3** (226 mg) and 1 mmol of appropriate benzyl isothiocyanate in ethanol. The reaction mixture was heated to reflux for 3 hours and then cooled to room temperature. The precipitate was filtered off and washed with a small amount of cold ethanol, giving a pure product [46].

1 mmol of *N*-benzyl-2-(3,5-dinitrobenzoyl)hydrazine-1-carbothioamide **6b–d** was suspended in 30 mL of tetrahydrofuran with stirring and 1.2 equivalents (1.2 mmol, 229 mg) of *p*-toluenesulfonyl chloride were added followed by 2.1 mmol (169 µL) of pyridine. The mixture was refluxed for 14 hours. The solvent was evaporated under reduced pressure to dryness, and the crude product **7b–d** was purified by column chromatography, using chloroform as an eluent [46].

Synthesis of *N*-benzyl-1,3,4-thiadiazole-2-amines **8b-d**

Method C

1 mmol of *N*-benzyl-2-(3,5-dinitrobenzoyl)hydrazine-1-carbothioamide **6b–d** was suspended in 15 mL of *N*-methyl-2-pyrrolidone (NMP) with stirring and 1.2 equivalents (1.2 mmol, 229 mg) of *p*-toluenesulfonyl chloride were added followed by 2.1 mmol (169 µL) of triethylamine. The mixture was allowed to react at room temperature for 48 hours. The solvent was evaporated under reduced pressure to dryness, and the crude product **8b–d** was purified by column chromatography using various eluents (details are in the characterisation of the compounds) [46].

Synthesis of 5-(3,5-dinitrophenyl)-1,3,4-oxadiazole-2-thiol **9**

1.4 g (6.2 mmol) of 3,5-dinitrobenzohydrazide **3** was dissolved together with 1.12 mL (18.4 mmol; 3 eq.) of carbon disulfide and 0.42 g (7.25 mmol; 1.2 eq.) of potassium hydroxide in 20 mL of water. The reaction mixture was heated at 90°C for 10 hours, then treated with 6 mL of 0.1 M sodium hydroxide solution. The formed precipitate was filtered. The aqueous solution was acidified with 10% hydrochloric acid to pH 2**–**3 and extracted twice with ethyl acetate (2 × 25 mL). The organic layers were combined and washed with water (25 mL) and brine (25 mL). The organic solution was dried by standing over sodium sulfate. Ethyl acetate was evaporated under reduced pressure, and the crude product was purified by column chromatography using a mixture of chloroform/*n*-hexane/acetic acid (24:6:1 V/V) as eluent. Yield: 52% as a yellow solid. mp 203–210°C. Elemental analysis for C_8_H_4_N_4_O_5_S (268.20); calculated C, 35.83; H, 1.50; N, 20.89, found: C, 35.91; H, 1.60; N, 20.92. R_*f*_: 0.2; chloroform/*n*-hexane/acetic acid (24:6:1 V/V). The measured characteristics were according to the literature [12].

Synthesis of 2-(3,5-dinitrophenyl)-5-(dodecylthio)-1,3,4-oxadiazole **10**

268 mg (1 mmol) of 5-(3,5-dinitrophenyl)-1,3,4-oxadiazole-2-thiol **9** was placed in the round bottom flask and dissolved in 7 mL of DMF. Then, 276 mg (2 mmol; 2 eq.) of potassium carbonate was added in one portion, followed by 264 µL (1.1 mmol; 1.1 eq.) of 1-bromododecane. The reaction mixture was stirred at room temperature for 5 hours and diluted with 10 mL of diethyl ether. The formed precipitate was filtered off and washed with a small volume of diethyl ether. The crude product was recrystallised from diethyl ether, giving a pure product **10** as a yellow solid, yielding 67%.

2-(3,5-Dinitrobenzoyl)-*N*-methylhydrazine-1-carboxamide **4a**

Beige solid, yield 58%; mp 200–202°C. IR (ATR): 650, 659, 724, 732, 775, 841, 927, 1085, 1151, 1167, 1257, 1319, 1345 (*ν*_*sym*_ NO_2_), 1417, 1509, 1539 (*ν*_*asym*_ NO_2_), 1579, 1593, 1660 (*ν* **CO**NH), 1679 (*ν* **CO**NH), 3078, 3097 (*ν*_*sym*_ C-H arom.), 3335 (*ν* N-H) cm^-1^. ^1^H NMR (500 MHz, DMSO-*d*_*6*_) *δ* 10.81 (1H, s, NH), 9.06 (2H, d, *J* = 2.1 Hz, H2, H6), 8.99 (1H, t, *J* = 2.1 Hz, H4), 8.13 (1H, s, NH), 6.64 (1H, s, NH-CH_3_), 2.59 (3H, d, *J* = 4.5 Hz, NH-CH_3_). ^13^C NMR (126 MHz, DMSO) *δ* 163.14, 158.96, 148.55, 135.99, 129.36, 121.71, 26.66. Elemental analysis for C_9_H_9_N_5_O_6_ (283.20); calculated C, 38.17; H, 3.20; N, 24.73, found: C, 38.25; H, 3.11; N, 24.81. R_*f*_: 0.4 (S1).

2-(3,5-Dinitrobenzoyl)-*N*-ethylhydrazine-1-carboxamide **4b**

Yellow solid, yield 65%; mp 194–196°C. IR (ATR): 717, 729, 743, 916, 1077, 1249, 1342 (*ν*_*sym*_ NO_2_), 1376, 1457, 1484, 1534 (*ν*_*asym*_ NO_2_), 1567, 1594, 1626 (*ν* **CO**NH), 2976 (*ν*_*asym*_ C-H aliph.), 3108 (*ν*_*sym*_ C-H arom.), 3249 (*ν* N-H), 3352 (*ν* N-H) cm^-1^. ^1^H NMR (500 MHz, 50°C, DMSO-*d*_*6*_) *δ* 10.81 (1H, s, NH), 9.06 (2H, d, *J* = 2.1 Hz, H2, H6), 8.99 (1H, t, *J* = 2.1 Hz, H4), 8.07 (1H, s, NH), 6.69 (1H, s, NH-CH_2_CH_3_), 3.11–3.02 (2H, m, NH-CH_2_-CH_3_), 1.01 (3H, t, *J* = 7.1 Hz, NH-CH_2_-CH_3_). ^13^C NMR (126 MHz, 50°C, DMSO) *δ* 162.76, 158.01, 148.32, 135.69, 128.04, 121.44, 34.25, 15.75. Elemental analysis for C_10_H_11_N_5_O_6_ (297.23); calculated C, 40.41; H, 3.73; N, 23.56, found: C, 40.49; H, 3.81; N, 23.60. R_*f*_: 0.52 (S1).

2-(3,5-Dinitrobenzoyl)-*N*-propylhydrazine-1-carboxamide **4c**

Yellow solid, yield 68%; mp 175–176°C. IR (ATR): 717, 729, 916, 1076, 1242, 1343 (*ν*_*sym*_ NO_2_), 1484, 1537 (*ν*_*asym*_ NO_2_), 1557, 1566, 1625 (*ν* **CO**NH), 2978 (*ν*_*asym*_ C-H aliph.), 3104 (*ν*_*sym*_ C-H arom.), 3211 (*ν* N-H) cm^-1^. ^1^H NMR (500 MHz, 50°C, DMSO-*d*_*6*_) *δ* 10.81 (1H, s, NH), 9.06 (2H, d, *J* = 2.1 Hz, H2, H6), 8.99 (1H, t, *J* = 2.1 Hz, H4), 8.05 (1H, s, NH), 6.69 (1H, s, NH-CH_2_-CH_2_), 3.00 (2H, dt, *J* = 7.5, 6.1 Hz, NH-CH_2-_CH_2_), 1.45–1.37 (2H, m, NH-CH_2_-CH_2_), 0.84 (3H, t, *J* = 7.4 Hz, CH_3_). ^13^C NMR (126 MHz, 50°C, DMSO) *δ* 162.74, 158.14, 148.32, 135.69, 128.03, 121.43, 41.18, 23.25, 11.44. Elemental analysis for C_11_H_13_N_5_O_6_ (311.25); calculated C, 42.45; H, 4.21; N, 22.50, found: C, 42.50; H, 4.10; N, 22.52. R_*f*_: 0.54 (S1).

*N*-Butyl-2-(3,5-dinitrobenzoyl)hydrazine-1-carboxamide **4d**

White solid, yield 75%; mp 174–175°C. IR (ATR): 717, 730, 752, 762, 917, 922, 1078, 1146, 1217, 1243, 1343 (*ν*_*sym*_ NO_2_), 1466, 1537 (*ν*_*asym*_ NO_2_), 1558, 1573, 1650 (*ν* **CO**NH), 1663 (*ν* **CO**NH), 1683, 1706, 2875 (*ν*_*sym*_ C-H aliph.), 2957 (*ν*_*asym*_ C-H aliph.), 3107 (*ν*_*sym*_ C-H arom.), 3224 (*ν* N-H), 3411 (*ν* N-H) cm^-1^. ^1^H NMR (500 MHz, DMSO-*d*_*6*_) *δ* 10.80 (1H, s, NH), 9.06 (2H, d, *J* = 2.1 Hz, H2, H6), 8.99 (1H, t, *J* = 2.1 Hz, H4), 8.04 (1H, s, NH), 6.66 (1H, s, NH-CH_2_-CH_2_), 3.03 (2H, td, *J* = 7.0, 5.7 Hz, NH-CH_2_-CH_2_), 1.39 (2H, tt, *J* = 7.6, 6.2 Hz, NH-CH_2_-CH_2_), 1.33–1.22 (2H, m, C^3^H_2_), 0.87 (3H, t, *J* = 7.3 Hz, CH_3_). ^13^C NMR (126 MHz, DMSO) *δ* 162.72, 158.09, 148.31, 135.68, 128.00, 121.41, 39.06, 32.18, 19.61, 13.91. Elemental analysis for C_12_H_15_N_5_O_6_ (325.10); calculated C, 44.31; H, 4.65; N, 21.53, found: C, 44.42; H, 4.71; N, 21.47. R_*f*_: 0.54 (S1).

2-(3,5-Dinitrobenzoyl)-*N*-pentylhydrazine-1-carboxamide **4e**

Yellow solid, yield 72%; mp 160–162°C. IR (ATR): 718, 730, 752, 763, 917, 1078, 1236, 1258, 1343 (*ν*_*sym*_ NO_2_), 1477, 1538 (*ν*_*asym*_ NO_2_), 1566, 1594, 1650 (*ν* **CO**NH), 1663 (*ν* **CO**NH), 2872 (*ν*_*sym*_ C-H aliph.), 2934 (*ν*_*asym*_ C-H aliph.), 3104 (*ν*_*sym*_ C-H arom.), 3237 (*ν* N-H) cm^-1^. ^1^H NMR (600 MHz, DMSO-*d*_*6*_) *δ* 10.77 (1H, s, NH), 9.02 (2H, d, *J* = 2.1 Hz, H2, H6), 8.95 (1H, t, *J* = 2.1 Hz, H4), 8.00 (1H, s, NH), 6.63 (1H, s, NH-CH_2_-CH_2_), 2.99 (2H, td, *J* = 7.1, 5.8 Hz, NH-CH_2_-CH_2_), 1.41–1.33 (2H, m, NH-CH_2_-CH_2_), 1.28–1.18 (4H, m, C^3^H_2_, C^4^H_2_), 0.83 (3H, t, *J* = 7.1 Hz, CH_3_). ^13^C NMR (151 MHz, DMSO) *δ* 163.08, 158.45, 148.67, 136.05, 128.37, 121.77, 39.64, 30.08, 29.05, 22.43, 14.49. Elemental analysis for C_13_H_17_N_5_O_6_ (339.31); calculated C, 46.02; H, 5.05; N, 20.64, found: C, 46.13; H, 5.07; N, 20.59. R_*f*_: 0.56 (S1).

2-(3,5-Dinitrobenzoyl)-*N*-hexylhydrazine-1-carboxamide **4f**

Yellowish solid, yield 80%; mp 148–149°C. IR (ATR): 721, 730, 920, 1075, 1149, 1247, 1302, 1343 (*ν*_*sym*_ NO_2_), 1468, 1542 (*ν*_*asym*_ NO_2_), 1616 (*ν* **CO**NH), 1653 (*ν* **CO**NH), 2859 (*ν*_*sym*_ C-H aliph.), 2930 (*ν*_*asym*_ C-H aliph.), 3103 (*ν*_*sym*_ C-H arom.), 3237 (*ν* N-H) cm^-1^. ^1^H NMR (500 MHz, DMSO-*d*_*6*_) *δ* 10.81 (1H, s, NH), 9.06 (2H, d, *J* = 2.1 Hz, H2, H6), 8.99 (1H, t, *J* = 2.1 Hz, H4), 8.04 (1H, s, NH), 6.67 (1H, s, NH-CH_2_-CH_2_), 3.02 (2H, td, *J* = 7.1, 5.9 Hz, NH-CH_2_-CH_2_), 1.44–1.34 (2H, m, NH-CH_2_-CH_2_), 1.32–1.19 (6H, m, C^3^H_2_, C^4^H_2_, C^5^H_2_), 0.86 (3H, t, *J* = 7.1 Hz, CH_3_). ^13^C NMR (126 MHz, DMSO) *δ* 162.73, 158.11, 148.32, 135.70, 128.02, 121.43, 39.20, 31.24, 30.02, 26.16, 22.29, 14.11. Elemental analysis for C_14_H_19_N_5_O_6_ (353.34); calculated C, 47.59; H, 5.42; N, 19.82, found: C, 47.62; H, 5.49; N, 19.91. R_*f*_: 0.56 (S1).

2-(3,5-Dinitrobenzoyl)-*N*-heptylhydrazine-1-carboxamide **4g**

White solid, yield 88%; mp 153–155°C. IR (ATR): 717, 731, 753, 920, 1083, 1242, 1350 (*ν*_*sym*_ NO_2_), 1476, 1541 (*ν*_*asym*_ NO_2_), 1569, 1616 (*ν* **CO**NH), 1653 (*ν* **CO**NH), 2854 (*ν*_*sym*_ C-H aliph.), 2935 (*ν*_*asym*_ C-H aliph.), 3099 (*ν*_*sym*_ C-H arom.), 3236 (*ν* N-H), 3355 (*ν* N-H) cm^-1^. ^1^H NMR (500 MHz, DMSO-*d*_*6*_) *δ* 10.81 (1H, s, NH), 9.07–9.05 (2H, m, H2, H6), 9.00–8.98 (1H, m, H4), 8.04 (1H, s, NH), 6.67 (1H, s, NH-CH_2_-CH_2_), 3.02 (2H, td, *J* = 7.1, 5.9 Hz, NH-CH_2_-CH_2_), 1.42–1.36 (2H, m, NH-CH_2_-CH_2_), 1.26–1.23 (8H, m, C^3^H_2_, C^4^H_2_, C^5^H_2_, C^6^H_2_), 0.85 (3H, t, *J* = 6.6 Hz, CH_3_). ^13^C NMR (126 MHz, DMSO) *δ* 162.99, 158.38, 148.58, 135.96, 128.29, 121.70, 39.46, 31.75, 30.33, 28.95, 26.72, 22.52, 14.42. Elemental analysis for C_15_H_21_N_5_O_6_ (367.36); calculated C, 49.04; H, 5.76; N, 19.06, found: C, 49.11; H, 5.83; N, 19.05. R_*f*_: 0.54 (S1).

2-(3,5-Dinitrobenzoyl)-*N*-octylhydrazine-1-carboxamide **4h**

White solid, yield 90%; mp 156–158°C. IR (ATR): 661, 717, 731, 753, 763, 920, 1083, 1237, 1350 (*ν*_*sym*_ NO_2_), 1475, 1539 (*ν*_*asym*_ NO_2_), 1571, 1616 (*ν* **CO**NH), 2849 (*ν*_*sym*_ C-H aliph.), 2925 (*ν*_*asym*_ C-H aliph.), 2956, 3100 (*ν*_*sym*_ C-H arom.), 3233 (*ν* N-H), 3356 (*ν* N-H) cm^-1^. ^1^H NMR (500 MHz, DMSO-*d*_*6*_) *δ* 10.80 (1H, s, NH), 9.06 (2H, d, *J* = 2.1 Hz, H2, H6), 8.99 (1H, t, *J* = 2.1 Hz, H4), 8.04 (1H, s, NH), 6.66 (1H, s, NH-CH_2_-CH_2_), 3.02 (2H, td, *J* = 7.1, 6.0 Hz, NH-CH_2_-CH_2_), 1.41–1.37 (2H, m, NH-CH_2_-CH_2_), 1.26–1.23 (10H, m, C^3^H_2_, C^4^H_2_, C^5^H_2_, C^6^H_2_, C^7^H_2_), 0.85 (3H, t, *J* = 6.7 Hz, CH_3_). ^13^C NMR (126 MHz, DMSO) *δ* 162.72, 158.10, 148.31, 135.70, 128.01, 121.42, 39.20, 31.43, 30.05, 28.97, 28.90, 26.49, 22.29, 14.13. Elemental analysis for C_16_H_23_N_5_O_6_ (381.39); calculated C, 50.39; H, 6.08; N, 18.36, found: C, 50.44; H, 6.18; N, 18.27. R_*f*_: 0.56 (S1).

2-(3,5-Dinitrobenzoyl)-*N*-nonylhydrazine-1-carboxamide **4i**

White solid, yield 93%; mp 154–155°C. IR (ATR): 661, 717, 733, 753, 763, 920, 1083, 1239, 1350 (*ν*_*sym*_ NO_2_), 1476, 1539 (*ν*_*asym*_ NO_2_), 1571, 1616 (*ν* **CO**NH), 2851 (*ν*_*sym*_ C-H aliph.), 2928 (*ν*_*asym*_ C-H aliph.), 3102 (*ν*_*sym*_ C-H arom.), 3233 (*ν* N-H), 3356 (*ν* N-H) cm^-1^. ^1^H NMR (500 MHz, DMSO-*d*_*6*_) *δ* 10.80 (1H, s, NH), 9.06 (2H, d, *J* = 2.1 Hz, H2, H6), 8.99 (1H, t, *J* = 2.1 Hz, H4), 8.04 (1H, s, NH), 6.66 (1H, s, NH-CH_2_-CH_2_), 3.02 (2H, q, *J* = 6.8 Hz, NH-CH_2_-CH_2_), 1.43–1.34 (2H, m, NH-CH_2_-CH_2_), 1.26–1.23 (12H, m, C^3^H_2_, C^4^H_2_, C^5^H_2_, C^6^H_2_, C^7^H_2_, C^8^H_2_), 0.85 (3H, t, *J* = 6.7 Hz, CH_3_). ^13^C NMR (126 MHz, DMSO) *δ* 162.70, 158.09, 148.30, 135.70, 128.00, 121.40, 39.20, 31.48, 30.04, 29.20, 29.02, 28.85, 26.48, 22.28, 14.13. Elemental analysis for C_17_H_25_N_5_O_6_ (395.42); calculated C, 51.64; H, 6.37; N, 17.71, found: C, 51.70; H, 6.45; N, 17.77. R_*f*_: 0.52 (S1).

*N*-Decyl-2-(3,5-dinitrobenzoyl)hydrazine-1-carboxamide **4j**

White solid, yield 95%; mp 153–155°C. IR (ATR): 717, 731, 753, 764, 920, 1084, 1235, 1250, 1350 (*ν*_*sym*_ NO_2_), 1475, 1539 (*ν*_*asym*_ NO_2_), 1559, 1571, 1615 (*ν* **CO**NH), 1654 (*ν* **CO**NH), 2850 (*ν*_*sym*_ C-H aliph.), 2925 (*ν*_*asym*_ C-H aliph.), 3100 (*ν*_*sym*_ C-H arom.), 3212 (*ν* N-H), 3358 (*ν* N-H) cm^-1^. ^1^H NMR (600 MHz, DMSO-*d*_*6*_) *δ* 10.77 (1H, s, NH), 9.02 (2H, d, *J* = 2.1 Hz, H2, H6), 8.95 (1H, t, *J* = 2.1 Hz, H4), 8.00 (1H, s, NH), 6.62 (1H, s, NH-CH_2_-CH_2_), 2.98 (2H, q, *J* = 6.6 Hz, NH-CH_2_-CH_2_), 1.37–1.33 (2H, m, NH-CH_2_-CH_2_), 1.23–1.18 (14H, m, C^3^H_2_, C^4^H_2_, C^5^H_2_, C^6^H_2_, C^7^H_2_, C^8^H_2_, C^9^H_2_), 0.81 (3H, t, *J* = 6.9 Hz, CH_3_). ^13^C NMR (151 MHz, DMSO) *δ* 163.06, 158.46, 148.67, 136.07, 128.36, 121.77, 39.65, 31.84, 30.40, 29.61, 29.52, 29.37, 29.26, 26.84, 22.63, 14.48. Elemental analysis for C_18_H_27_N_5_O_6_ (409.44); calculated C, 52.80; H, 6.65; N, 17.10, found: C, 52.88; H, 6.70; N, 17.18. R_*f*_: 0.50 (S1).

2-(3,5-Dinitrobenzoyl)-*N*-undecylhydrazine-1-carboxamide **4k**

White solid, yield 94%; mp 157–158°C. IR (ATR): 718, 731, 764, 920, 1084, 1110, 1245, 1350 (*ν*_*sym*_ NO_2_), 1475, 1540 (*ν*_*asym*_ NO_2_), 1559, 1571, 1615 (*ν* **CO**NH), 1653 (*ν* **CO**NH), 2848 (*ν*_*sym*_ C-H aliph.), 2924 (*ν*_*asym*_ C-H aliph.), 3100 (*ν*_*sym*_ C-H arom.), 3212 (*ν* N-H), 3360 (*ν* N-H) cm^-1^. ^1^H NMR (600 MHz, DMSO-*d*_*6*_) *δ* 10.77 (1H, s, NH), 9.02 (2H, d, *J* = 2.1 Hz, H2, H6), 8.95 (1H, t, *J* = 2.2 Hz, H4), 8.00 (1H, s, NH), 6.62 (1H, s, NH-CH_2_-CH_2_), 2.98 (2H, q, *J* = 6.6 Hz, NH-CH_2_-CH_2_), 1.37–1.33 (2H, m, NH-CH_2_-CH_2_), 1.23–1.18 (16H, m, C^3^H_2_, C^4^H_2_, C^5^H_2_, C^6^H_2_, C^7^H_2_, C^8^H_2_, C^9^H_2_, C^10^H_2_), 0.81 (3H, t, *J* = 6.9 Hz, CH_3_). ^13^C NMR (151 MHz, DMSO) *δ* 163.06, 158.45, 148.67, 136.06, 128.37, 121.77, 39.65, 31.84, 30.40, 29.62, 29.60, 29.56, 29.37, 29.25, 26.84, 22.63, 14.48. Elemental analysis for C_19_H_29_N_5_O_6_ (423.47); calculated C, 53.89; H, 6.90; N, 16.54, found: C, 53.96; H, 6.91; N, 16.60. R_*f*_: 0.50 (S1).

2-(3,5-Dinitrobenzoyl)-*N*-dodecylhydrazine-1-carboxamide **4l**

White solid, yield 96%; mp 157–159°C. IR (ATR): 717, 732, 920, 1084, 1241, 1350 (*ν*_*sym*_ NO_2_), 1466, 1476, 1538 (*ν*_*asym*_ NO_2_), 1569, 1615 (*ν* **CO**NH), 1653 (*ν* **CO**NH), 2848 (*ν*_*sym*_ C-H aliph.), 2924 (*ν*_*asym*_ C-H aliph.), 3099 (*ν*_*sym*_ C-H arom.), 3216 (*ν* N-H), 3357 (*ν* N-H) cm^-1^. ^1^H NMR (500 MHz, 50°C, DMSO-*d*_*6*_) *δ* 10.81 (1H, s, NH), 9.06 (2H, d, *J* = 2.0 Hz, H2, H6), 8.99 (1H, t, *J* = 2.1 Hz, H4), 8.04 (1H, s, NH), 6.67 (1H, s, NH-CH_2_-CH_2_), 3.02 (2H, q, *J* = 6.6 Hz, NH-CH_2_-CH_2_), 1.41–1.36 (2H, m, NH-CH_2_-CH_2_), 1.27–1.21 (18H, m, C^3^H_2_, C^4^H_2_, C^5^H_2_, C^6^H_2_, C^7^H_2_, C^8^H_2_, C^9^H_2_, C^10^H_2_, C^11^H_2_), 0.85 (3H, t, *J* = 6.9 Hz, CH_3_). ^13^C NMR (126 MHz, 50°C, DMSO) *δ* 162.70, 158.10, 148.31, 135.70, 128.01, 121.41, 39.20, 31.49, 30.06, 29.27, 29.25, 29.22, 29.20, 29.03, 28.91, 26.49, 22.29, 14.14. Elemental analysis for C_20_H_31_N_5_O_6_ (437.50); calculated C, 54.91; H, 7.14; N, 16.01, found: C, 55.01; H, 7.21; N, 16.00. R_*f*_: 0.50 (S1).

2-(3,5-Dinitrobenzoyl)-*N*-tridecylhydrazine-1-carboxamide **4m**

White solid, yield 98%; mp 158–160°C. IR (ATR): 718, 731, 751, 920, 1083, 1239, 1350 (*ν*_*sym*_ NO_2_), 1475, 1489, 1540 (*ν*_*asym*_ NO_2_), 1559, 1571, 1615 (*ν* **CO**NH), 1654 (*ν* **CO**NH), 2848 (*ν*_*sym*_ C-H aliph.), 2924 (*ν*_*asym*_ C-H aliph.), 3100 (*ν*_*sym*_ C-H arom.), 3359 (*ν* N-H) cm^-1^. ^1^H NMR (600 MHz, DMSO-*d*_*6*_) *δ* 10.77 (1H, s, NH), 9.03 (2H, d, *J* = 2.1 Hz, H2, H6), 8.95 (1H, t, *J* = 2.1 Hz, H4), 8.00 (1H, s, NH), 6.62 (1H, s, NH-CH_2_-CH_2_), 2.98 (2H, q, *J* = 6.6 Hz, NH-CH_2_-CH_2_), 1.39–1.32 (2H, m, NH-CH_2_-CH_2_), 1.21–1.14 (20H, m, C^3^H_2_, C^4^H_2_, C^5^H_2_, C^6^H_2_, C^7^H_2_, C^8^H_2_, C^9^H_2_, C^10^H_2_, C^11^H_2_, C^12^H_2_), 0.81 (3H, t, *J* = 6.9 Hz, CH_3_). ^13^C NMR (151 MHz, DMSO) *δ* 163.05, 158.45, 148.67, 136.08, 128.36, 121.76, 39.65, 31.83, 30.40, 29.63, 29.61, 29.59, 29.57, 29.56, 29.37, 29.25, 26.84, 22.63, 14.47. Elemental analysis for C_21_H_33_N_5_O_6_ (451.52); calculated C, 55.86; H, 7.37; N, 15.51, found: C, 55.92; H, 7.41; N, 15.57. R_*f*_: 0.48 (S1).

2-(3,5-Dinitrobenzoyl)-*N*-tetradecylhydrazine-1-carboxamide **4n**

White solid, yield 99%; mp 161–162°C. IR (ATR): 717, 731, 753, 920, 1083, 1237, 1251, 1350 (*ν*_*sym*_ NO_2_), 1475, 1487, 1538 (*ν*_*asym*_ NO_2_), 1571, 1615 (*ν* **CO**NH), 2848 (*ν*_*sym*_ C-H aliph.), 2923 (*ν*_*asym*_ C-H aliph.), 2955, 3100 (*ν*_*sym*_ C-H arom.), 3234 (*ν* N-H), 3359 (*ν* N-H) cm^-1^. ^1^H NMR (600 MHz, DMSO-*d*_*6*_) *δ* 10.77 (1H, s, NH), 9.03 (2H, d, *J* = 2.1 Hz, H2, H6), 8.95 (1H, t, *J* = 2.2 Hz, H4), 8.00 (1H, s, NH), 6.62 (1H, s, NH-CH_2_-CH_2_), 2.98 (2H, q, *J* = 6.6 Hz, NH-CH_2_-CH_2_), 1.36–1.33 (2H, m, NH-CH_2_-CH_2_), 1.21–1.19 (22H, m, C^3^H_2_, C^4^H_2_, C^5^H_2_, C^6^H_2_, C^7^H_2_, C^8^H_2_, C^9^H_2_, C^10^H_2_, C^11^H_2_, C^12^H_2_, C^13^H_2_), 0.81 (3H, t, *J* = 6.9 Hz, CH_3_). ^13^C NMR (151 MHz, DMSO) *δ* 163.05, 158.46, 148.67, 136.07, 128.37, 121.76, 39.64, 31.83, 30.40, 29.61, 29.59, 29.57, 29.56, 29.54, 29.52, 29.37, 29.25, 26.84, 22.63, 14.47. Elemental analysis for C_22_H_35_N_5_O_6_ (465.55); calculated C, 56.76; H, 7.58; N, 15.04, found: C, 56.80; H, 7.50; N, 15.04. R_*f*_: 0.50 (S1).

2-(3,5-Dinitrobenzoyl)-*N*-pentadecylhydrazine-1-carboxamide **4o**

White solid, yield 92%; mp 156–157°C. IR (ATR): 717, 731, 752, 920, 1083, 1236, 1248, 1351 (*ν*_*sym*_ NO_2_), 1466, 1475, 1538 (*ν*_*asym*_ NO_2_), 1569, 1616 (*ν* **CO**NH), 2849 (*ν*_*sym*_ C-H aliph.), 2924 (*ν*_*asym*_ C-H aliph.), 3102 (*ν*_*sym*_ C-H arom.), 3212 (*ν* N-H), 3353 (*ν* N-H) cm^-1^. ^1^H NMR (600 MHz, DMSO-*d*_*6*_) *δ* 10.78 (1H, s, NH), 9.03 (2H, d, *J* = 2.3 Hz, H2, H6), 8.95 (1H, t, *J* = 2.1 Hz, H4), 8.01 (1H, s, NH), 6.63 (1H, s, NH-CH_2_-CH_2_), 2.98 (2H, q, *J* = 6.6 Hz, NH-CH_2_-CH_2_), 1.36–1.34 (2H, m, NH-CH_2_-CH_2_), 1.22–1.17 (24H, m, C^3^H_2_, C^4^H_2_, C^5^H_2_, C^6^H_2_, C^7^H_2_, C^8^H_2_, C^9^H_2_, C^10^H_2_, C^11^H_2_, C^12^H_2_, C^13^H_2_, C^14^H_2_), 0.81 (3H, t, *J* = 6.9 Hz, CH_3_). ^13^C NMR (151 MHz, DMSO) *δ* 163.04, 158.46, 148.67, 136.10, 128.37, 121.74, 39.65, 31.83, 30.40, 29.60, 29.57, 29.55, 29.53, 29.51, 29.50, 29.49, 29.37, 29.24, 26.84, 22.63, 14.47. Elemental analysis for C_23_H_37_N_5_O_6_ (479.58); calculated C, 57.60; H, 7.78; N, 14.60, found: C, 57.69; H, 7.79; N, 14.67. R_*f*_: 0.52 (S1).

2-(3,5-Dinitrobenzoyl)-*N*-hexadecylhydrazine-1-carboxamide **4p**

White solid, yield 98%; mp 157–158°C. IR (ATR): 717, 731, 753, 919, 1083, 1233, 1244, 1350 (*ν*_*sym*_ NO_2_), 1475, 1487, 1539 (*ν*_*asym*_ NO_2_), 1571, 1616 (*ν* **CO**NH), 2848 (*ν*_*sym*_ C-H aliph.), 2923 (*ν*_*asym*_ C-H aliph.), 2956, 3100 (*ν*_*sym*_ C-H arom.), 3227 (*ν* N-H), 3358 (*ν* N-H) cm^-1^. ^1^H NMR (500 MHz, DMSO-*d*_*6*_) *δ* 10.81 (1H, s, NH), 9.06 (2H, d, *J* = 2.3 Hz, H2, H6), 8.99 (1H, t, *J* = 2.1 Hz, H4), 8.04 (1H, s, NH), 6.66 (1H, s, NH-CH_2_-CH_2_), 3.01 (2H, q, *J* = 6.6 Hz, NH-CH_2_-CH_2_), 1.40–1.36 (2H, m, NH-CH_2_-CH_2_), 1.24–1.22 (26H, m, C^3^H_2_, C^4^H_2_, C^5^H_2_, C^6^H_2_, C^7^H_2_, C^8^H_2_, C^9^H_2_, C^10^H_2_, C^11^H_2_, C^12^H_2_, C^13^H_2_, C^14^H_2_, C^15^H_2_), 0.84 (3H, t, *J* = 6.8 Hz, CH_3_). ^13^C NMR (126 MHz, DMSO) *δ* 162.70, 158.11, 148.31, 135.71, 128.02, 121.41, 39.20, 31.49, 30.06, 29.29, 29.27, 29.25, 29.23, 29.21, 29.19, 29.18, 29.15, 29.04, 28.91, 26.50, 22.29, 14.14. Elemental analysis for C_24_H_39_N_5_O_6_ (493.61); calculated C, 58.40; H, 7.96; N, 14.19, found: C, 58.31; H, 8.01; N, 14.29. R_*f*_: 0.54 (S1).

2-(3,5-Dinitrobenzoyl)-*N*-heptadecylhydrazine-1-carboxamide **4q**

White solid, yield 90%; mp 149–151°C. IR (ATR): 717, 731, 752, 919, 1083, 1235, 1246, 1351 (*ν*_*sym*_ NO_2_), 1475, 1540 (*ν*_*asym*_ NO_2_), 1571, 1616 (*ν* **CO**NH), 2850 (*ν*_*sym*_ C-H aliph.), 2921 (*ν*_*asym*_ C-H aliph.), 3100 (*ν*_*sym*_ C-H arom.), 3220 (*ν* N-H), 3354 (*ν* N-H) cm^-1^. ^1^H NMR (600 MHz, DMSO-*d*_*6*_) *δ* 10.77 (1H, s, NH), 9.02 (2H, d, *J* = 2.2 Hz, H2, H6), 8.95 (1H, t, *J* = 2.1 Hz, H4), 8.00 (1H, s, NH), 6.62 (1H, s, NH-CH_2_-CH_2_), 2.98 (2H, q, *J* = 6.6 Hz, NH-CH_2_-CH_2_), 1.36–1.33 (2H, m, NH-CH_2_-CH_2_), 1.21–1.17 (28H, m, C^3^H_2_, C^4^H_2_, C^5^H_2_, C^6^H_2_, C^7^H_2_, C^8^H_2_, C^9^H_2_, C^10^H_2_, C^11^H_2_, C^12^H_2_, C^13^H_2_, C^14^H_2_, C^15^H_2_, C^16^H_2_), 0.81 (3H, t, *J* = 6.9 Hz, CH_3_). ^13^C NMR (151 MHz, DMSO) *δ* 163.01, 158.44, 148.67, 136.15, 128.35, 121.73, 39.65, 31.82, 30.40, 30.00, 29.58, 29.56, 29.54, 29.53, 29.52, 29.50, 29.48, 29.47, 29.37, 29.23, 26.84, 22.62, 14.47. Elemental analysis for C_25_H_41_N_5_O_6_ (507.63); calculated C, 59.15; H, 8.14; N, 13.80, found: C, 59.22; H, 8.10; N, 13.79. R_*f*_: 0.54 (S1).

2-(3,5-Dinitrobenzoyl)-*N*-octadecylhydrazine-1-carboxamide **4r**

White solid, yield 99%; mp 159–160°C. IR (ATR): 717, 731, 751, 920, 1083, 1239, 1252, 1351 (*ν*_*sym*_ NO_2_), 1475, 1488, 1539 (*ν*_*asym*_ NO_2_), 1571, 1616 (*ν* **CO**NH), 2849 (*ν*_*sym*_ C-H aliph.), 2923 (*ν*_*asym*_ C-H aliph.), 3101 (*ν*_*sym*_ C-H arom.), 3225 (*ν* N-H), 3356 (*ν* N-H) cm^-1^. ^1^H NMR (500 MHz, 50°C, DMSO-*d*_*6*_) *δ* 10.71 (1H, s, NH), 9.06 (2H, d, *J* = 2.1 Hz, H2, H6), 8.98 (2H, t, *J* = 2.1 Hz, H4), 7.92 (1H, s, NH), 6.50 (1H, s, NH-CH_2_-CH_2_), 3.04 (2H, q, *J* = 6.6 Hz, NH-CH_2_-CH_2_), 1.42–1.40 (2H, m, NH-CH_2_-CH_2_), 1.27–1.23 (30H, m, C^3^H_2_, C^4^H_2_, C^5^H_2_, C^6^H_2_, C^7^H_2_, C^8^H_2_, C^9^H_2_, C^10^H_2_, C^11^H_2_, C^12^H_2_, C^13^H_2_, C^14^H_2_, C^15^H_2_, C^16^H_2_, C^17^H_2_), 0.86 (3H, t, *J* = 6.8 Hz, CH_3_). ^13^C NMR (126 MHz, 50°C, DMSO) *δ* 162.40, 157.75, 148.17, 135.72, 127.70, 121.01, 39.66, 31.17, 29.75, 29.73, 29.71, 29.69, 29.67, 29. 65, 29.63, 29.61, 28.94, 28.90, 28.86, 28.72, 28.55, 26.24, 21.94, 13.74. Elemental analysis for C_26_H_43_N_5_O_6_ (521.66); calculated C, 59.86; H, 8.31; N, 13.43, found: C, 59.91; H, 8.20; N, 13.49. R_*f*_: 0.54 (S1).

2-(3,5-Dinitrobenzoyl)-*N*-dodecylhydrazine-1-carbothioamide **4s**

Brown solid, yield 95%; mp 174–175°C. IR (ATR): 731, 914, 1073, 1171, 1238, 1257, 1342 (*ν*_*sym*_ NO_2_), 1525, 1545 (*ν*_*asym*_ NO_2_), 1559, 1568, 1697, 1702, 2855 (*ν*_*sym*_ C-H aliph.), 2924 (*ν*_*asym*_ C-H aliph.), 3106 (*ν*_*sym*_ C-H arom.), 3167 (*ν* N-H), 3347 (*ν* N-H) cm^-1^. ^1^H NMR (600 MHz, DMSO-*d*_*6*_) *δ* 10.96 (1H, s, NH), 9.38 (1H, s, NH), 9.03 (2H, d, *J* = 2.1 Hz, H2, H6), 8.97 (1H, t, J = 2.1 Hz, H4), 8.23 (1H, s, NH-CH_2_-CH_2_), 3.40 (2H, q, *J* = 6.6 Hz, NH-CH_2_-CH_2_), 1.46 (2H, p, *J* = 7.0 Hz, NH-CH_2_-CH_2_), 1.26–1.14 (18H, m, C^3^H_2_, C^4^H_2_, C^5^H_2_, C^6^H_2_, C^7^H_2_, C^8^H_2_, C^9^H_2_, C^10^H_2_, C^11^H_2_), 0.81 (3H, t, *J* = 7.0 Hz, CH_3_). ^13^C NMR (151 MHz, DMSO) *δ* 162.88, 148.60, 136.01, 128.56, 121.91, 44.28, 31.83, 29.60, 29.55, 29.45, 29.43, 29.41, 29.35, 29.26, 26.77, 22.63, 14.47. Elemental analysis for C_20_H_31_N_5_O_5_S (453.56); calculated C, 52.96; H, 6.89; N, 15.44, found: C, 53.00; H, 6.80; N, 15.53. R_*f*_: 0.36 (S1).

5-(3,5-Dinitrophenyl)-*N*-methyl-1,3,4-oxadiazol-2-amine **5a**

Yellow solid, yield 55%; mp 213–214°C. IR (ATR): 688, 731, 914, 1019, 1077, 1109, 1345 (*ν*_*sym*_ NO_2_), 1354, 1415, 1535 (*ν*_*asym*_ NO_2_), 1568, 1661 (C = N), 3104 (*ν*_*sym*_ C-H arom.) cm^-1^. ^1^H NMR (600 MHz, DMSO-*d*_*6*_) *δ* 8.83 (1H, t, *J* = 2.1 Hz, H4), 8.72 (2H, d, *J* = 2.1 Hz, H2, H6), 7.97 (1H, q, *J* = 4.8 Hz, NH-CH_3_), 2.88 (3H, d, *J* = 4.9 Hz, NH-CH_3_). ^13^C NMR (151 MHz, DMSO) *δ* 165.36, 155.47, 149.26, 127.34, 125.05, 119.74, 29.54. Elemental analysis for C_9_H_7_N_5_O_5_ (265.19); calculated C, 40.76; H, 2.66; N, 26.41, found: C, 40.83; H, 2.61; N, 26.50. R_*f*_: 0.29 (S2).

5-(3,5-Dinitrophenyl)-*N*-ethyl-1,3,4-oxadiazol-2-amine **5b**

Yellow solid, yield 67%; mp 236–237°C. IR (ATR): 687, 729, 920, 1037, 1082, 1344 (*ν*_*sym*_ NO_2_), 1476, 1539 (*ν*_*asym*_ NO_2_), 1567, 1661 (C = N), 2972 (*ν*_*asym*_ C-H aliph.), 3093 (*ν*_*sym*_ C-H arom.) cm^-1^. ^1^H NMR (500 MHz, DMSO-*d*_*6*_) *δ* 8.86 (1H, t, *J* = 2.1 Hz, H4), 8.76 (2H, d, *J* = 2.1 Hz, H2, H6), 8.12 (1H, t, *J* = 5.6 Hz, NH-CH_2_-CH_3_), 3.35–3.29 (2H, m, NH-CH_2_-CH_3_), 1.20 (3H, t, *J* = 7.2 Hz, NH-CH_2_-CH_3_). ^13^C NMR (126 MHz, DMSO) *δ* 164.60, 155.26, 149.16, 127.27, 124.96, 119.63, 37.95, 14.94. Elemental analysis for C_10_H_9_N_5_O_5_ (279.21); calculated C, 43.02; H, 3.25; N, 25.08, found: C, 43.12; H, 3.27; N, 25.18. R_*f*_: 0.36 (S2).

5-(3,5-Dinitrophenyl)-*N*-propyl-1,3,4-oxadiazol-2-amine **5c**

Yellow solid, yield 62%; mp 225–226°C. IR (ATR): 687, 728, 914, 1030, 1078, 1145, 1344 (*ν*_*sym*_ NO_2_), 1477, 1537 (*ν*_*asym*_ NO_2_), 1566, 1662 (C = N), 2942 (*ν*_*asym*_ C-H aliph.), 3094 (*ν*_*sym*_ C-H arom.) cm^-1^. ^1^H NMR (500 MHz, DMSO-*d*_*6*_) *δ* 8.86 (1H, t, *J* = 2.1 Hz, H4), 8.76 (2H, d, *J* = 2.1 Hz, H2, H6), 8.15 (1H, t, *J* = 5.7 Hz, NH-CH_2_-CH_2_), 3.25 (2H, td, *J* = 7.0, 5.7 Hz, NH-CH_2_-CH_2_), 1.64–1.56 (2H, m, NH-CH_2_-CH_2_), 0.93 (3H, t, *J* = 7.4 Hz, CH_3_). ^13^C NMR (126 MHz, DMSO) *δ* 164.78, 155.20, 149.16, 127.28, 124.96, 119.61, 44.82, 22.49, 11.65. Elemental analysis for C_11_H_11_N_5_O_5_ (293.24); calculated C, 45.06; H, 3.78; N, 23.88, found: C, 45.12; H, 3.71; N, 23.97. R_*f*_: 0.41 (S2).

*N*-Butyl-5-(3,5-dinitrophenyl)-1,3,4-oxadiazol-2-amine **5d**

Yellow solid, yield 70%; mp 216–218°C. IR (ATR): 687, 729, 911, 1037, 1078, 1324, 1346 (*ν*_*sym*_ NO_2_), 1539 (*ν*_*asym*_ NO_2_), 1568, 1662 (C = N), 2876 (*ν*_*sym*_ C-H aliph.), 2959 (*ν*_*asym*_ C-H aliph.), 3099 (*ν*_*sym*_ C-H arom.) cm^-1^. ^1^H NMR (500 MHz, DMSO-*d*_*6*_) *δ* 8.86 (1H, t, *J* = 2.1 Hz, H4), 8.75 (2H, d, *J* = 2.1 Hz, H2, H6), 8.14 (1H, t, *J* = 5.7 Hz, NH-CH_2_-CH_2_), 3.28 (2H, td, *J* = 7.0, 5.7 Hz, NH-CH_2_-CH_2_), 1.62–1.52 (2H, m, NH-CH_2_-CH_2_), 1.42–1.31 (2H, m, C^3^H_2_), 0.91 (3H, t, *J* = 7.4 Hz, CH_3_). ^13^C NMR (126 MHz, DMSO) *δ* 164.75, 155.19, 149.15, 127.28, 124.94, 119.60, 42.73, 31.27, 19.83, 14.05. Elemental analysis for C_12_H_13_N_5_O_5_ (307.27); calculated C, 46.91; H, 4.26; N, 22.79, found: C, 47.01; H, 4.29; N, 22.86. R_*f*_: 0.45 (S2).

5-(3,5-Dinitrophenyl)-*N*-pentyl-1,3,4-oxadiazol-2-amine **5e**

Yellow solid, yield 70%; mp 198–200°C. IR (ATR): 730, 912, 1029, 1082, 1106, 1346 (*ν*_*sym*_ NO_2_), 1472, 1539 (*ν*_*asym*_ NO_2_), 1559, 1568, 1668 (C = N), 2959 (*ν*_*asym*_ C-H aliph.), 3080 (*ν*_*sym*_ C-H arom.) cm^-1^. ^1^H NMR (500 MHz, DMSO-*d*_*6*_) *δ* 8.86 (1H, t, *J* = 2.1 Hz, H4), 8.75 (2H, d, *J* = 2.1 Hz, H2, H6), 8.13 (1H, t, *J* = 5.7 Hz, NH-CH_2_-CH_2_), 3.27 (2H, td, *J* = 7.1, 5.7 Hz, NH-CH_2_-CH_2_), 1.62–1.56 (2H, m, NH-CH_2_-CH_2_), 1.34–1.31 (4H, m, C^3^H_2_, C^4^H_2_), 0.92 (3H, t, *J* = 7.4 Hz, CH_3_). ^13^C NMR (126 MHz, DMSO) *δ* 164.74, 155.19, 149.16, 127.28, 124.94, 119.60, 43.01, 28.84, 28.82, 22.24, 14.35. Elemental analysis for C_13_H_15_N_5_O_5_ (321.29); calculated C, 48.60; H, 4.71; N, 21.80, found: C, 48.62; H, 4.80; N, 21.77. R_*f*_: 0.5 (S2).

5-(3,5-Dinitrophenyl)-*N*-hexyl-1,3,4-oxadiazol-2-amine **5f**

Yellow solid, yield 84%; mp 172–173°C. IR (ATR): 687, 730, 912, 1029, 1081, 1346 (*ν*_*sym*_ NO_2_), 1467, 1538 (*ν*_*asym*_ NO_2_), 1559, 1668 (C = N), 2958 (*ν*_*asym*_ C-H aliph.), 3088 (*ν*_*sym*_ C-H arom.) cm^-1^. ^1^H NMR (600 MHz, DMSO-*d*_*6*_) *δ* 8.83 (1H, t, *J* = 2.1 Hz, H4), 8.72 (2H, d, *J *= 2.1 Hz, H2, H6), 8.10 (1H, t, *J *= 5.7 Hz, NH-CH_2_-CH_2_), 3.24 (2H, td, *J* = 7.1, 5.7 Hz, NH-CH_2_-CH_2_), 1.55 (2H, p, *J* = 7.2 Hz, NH-CH_2_-CH_2_), 1.34–1.20 (6H, m, C^3^H_2_, C^4^H_2_, C^5^H_2_), 0.88 (3H, t, *J* = 7.4 Hz, CH_3_). ^13^C NMR (151 MHz, DMSO) *δ* 164.84, 155.28, 149.25, 127.37, 125.03, 119.69, 43.12, 40.60, 31.43, 29.20, 26.37, 22.58, 14.43. Elemental analysis for C_14_H_17_N_5_O_5_ (335.32); calculated C, 48.60; H, 4.71; N, 21.80, found: C, 48.62; H, 4.80; N, 21.77. R_*f*_: 0.54 (S2).

5-(3,5-Dinitrophenyl)-*N*-heptyl-1,3,4-oxadiazol-2-amine **5g**

Yellow solid, yield 81%; mp 196–197°C. IR (ATR): 687, 729, 911, 1029, 1080, 1324, 1346 (*ν*_*sym*_ NO_2_), 1467, 1539 (*ν*_*asym*_ NO_2_), 1559, 1668 (C = N), 2854 (*ν*_*sym*_ C-H aliph.), 2958 (*ν*_*asym*_ C-H aliph.), 3100 (*ν*_*sym*_ C-H arom.) cm^-1^. ^1^H NMR (500 MHz, DMSO-*d*_*6*_) *δ* 8.86 (1H, t, *J* = 2.1 Hz, H4), 8.75 (2H, d, *J* = 2.1 Hz, H2, H6), 8.13 (1H, t, *J* = 5.7 Hz, NH-CH_2_-CH_2_), 3.27 (2H, td, *J* = 7.1, 5.7 Hz, NH-CH_2_-CH_2_), 1.61–1.55 (2H, m, NH-CH_2_-CH_2_), 1.36–1.25 (8H, m, C^3^H_2_, C^4^H_2_, C^5^H_2_, C^6^H_2_), 0.87 (3H, t, *J* = 7.4 Hz, CH_3_). ^13^C NMR (126 MHz, DMSO) *δ* 164.46, 154.91, 148.88, 127.00, 124.66, 119.32, 42.75, 31.40, 28.89, 28.52, 26.31, 22.22, 14.11. Elemental analysis for C_15_H_19_N_5_O_5_ (349.35); calculated C, 51.57; H, 5.48; N, 20.05, found: C, 51.65; H, 5.52; N, 20.11. R_*f*_: 0.56 (S2).

5-(3,5-Dinitrophenyl)-*N*-octyl-1,3,4-oxadiazol-2-amine **5h**

Yellow solid, yield 89%; mp 164–166°C. IR (ATR): 688, 729, 914, 1054, 1079, 1324, 1348 (*ν*_*sym*_ NO_2_), 1467, 1539 (*ν*_*asym*_ NO_2_), 1568, 1668 (C = N), 2858 (*ν*_*sym*_ C-H aliph.), 2927 (*ν*_*asym*_ C-H aliph.), 3106 (*ν*_*sym*_ C-H arom.) cm^-1^. ^1^H NMR (500 MHz, DMSO-*d*_*6*_) *δ* 8.86 (1H, t, *J* = 2.1 Hz, H4), 8.76 (2H, d, *J* = 2.0 Hz, H2, H6), 8.13 (1H, t, *J* = 5.7 Hz, NH-CH_2_-CH_2_), 3.27 (2H, td, *J* = 7.0, 5.7 Hz, NH-CH_2_-CH_2_), 1.60–1.55 (2H, m, NH-CH_2_-CH_2_), 1.35–1.24 (10H, m, C^3^H_2_, C^4^H_2_, C^5^H_2_, C^6^H_2_, C^7^H_2_), 0.85 (3H, t, *J* = 7.4 Hz, CH_3_) ^13^C NMR (126 MHz, DMSO) *δ* 164.46, 154.91, 148.88, 127.00, 124.66, 119.32, 42.75, 31.41, 28.88, 28.86, 28.82, 26.35, 22.26, 14.11. Elemental analysis for C_16_H_21_N_5_O_5_ (363.37); calculated C, 52.89; H, 5.83; N, 19.27, found: C, 52.95; H, 5.81; N, 19.35. R_*f*_: 0.58 (S2).

5-(3,5-Dinitrophenyl)-*N*-nonyl-1,3,4-oxadiazol-2-amine **5i**

Yellow solid, yield 93%; mp 156–158°C. IR (ATR): 687, 729, 914, 1056, 1079, 1324, 1347 (*ν*_*sym*_ NO_2_), 1467, 1539 (*ν*_*asym*_ NO_2_), 1568, 1668 (C = N), 2857 (*ν*_*sym*_ C-H aliph.), 2927 (*ν*_*asym*_ C-H aliph.), 3106 (*ν*_*sym*_ C-H arom.) cm^-1^. ^1^H NMR (500 MHz, DMSO-*d*_*6*_) *δ* 8.86 (1H, t, *J* = 2.1 Hz, H4), 8.75 (2H, d, *J* = 2.1 Hz, H2, H6), 8.13 (1H, t, *J* = 5.7 Hz, NH-CH_2_-CH_2_), 3.27 (2H, td, *J* = 7.0, 5.7 Hz, NH-CH_2_-CH_2_), 1.59–1.55 (2H, m, NH-CH_2_-CH_2_), 1.33–1.23 (12H, m, C^3^H_2_, C^4^H_2_, C^5^H_2_, C^6^H_2_, C^7^H_2_, C^8^H_2_), 0.84 (3H, t, *J* = 7.1 Hz, CH_3_). ^13^C NMR (126 MHz, DMSO) *δ* 164.74, 155.19, 149.16, 127.28, 124.94, 119.61, 43.03, 31.74, 29.41, 29.15, 29.13, 29.11, 26.63, 22.56, 14.40. Elemental analysis for C_17_H_23_N_5_O_5_ (377.40); calculated C, 54.10; H, 6.14; N, 18.56, found: C, 54.19; H, 6.22; N, 18.49. R_*f*_: 0.59 (S2).

*N*-Decyl-5-(3,5-dinitrophenyl)-1,3,4-oxadiazol-2-amine **5j**

Yellow solid, yield 80%; mp 138–139°C. IR (ATR): 729, 911, 1029, 1072, 1120, 1341 (*ν*_*sym*_ NO_2_), 1353, 1472, 1539 (*ν*_*asym*_ NO_2_), 1545, 1667 (C = N), 2849 (*ν*_*sym*_ C-H aliph.), 2923 (*ν*_*asym*_ C-H aliph.), 3100 (*ν*_*sym*_ C-H arom.) cm^-1^. ^1^H NMR (600 MHz, acetone-*d*_*6*_) *δ* 8.96 (1H, t, *J* = 2.0 Hz, H4), 8.89 (2H, d, *J* = 2.1 Hz, H2, H6), 7.10 (1H, t, *J* = 5.8 Hz, NH-CH_2_-CH_2_), 3.43 (2H, td, *J* = 7.1, 5.8 Hz, NH-CH_2_-CH_2_), 1.72–1.69 (2H, m, NH-CH_2_-CH_2_), 1.44–1.39 (2H, m, C^3^H_2_), 1.37–1.22 (12H, m, C^4^H_2_, C^5^H_2_, C^6^H_2_, C^7^H_2_, C^8^H_2_, C^9^H_2_), 0.84 (3H, t, *J* = 6.9 Hz, CH_3_). ^13^C NMR (151 MHz, acetone) *δ* 164.75, 155.37, 149.29, 127.96, 124.60, 119.09, 43.24, 43.12, 31.80, 29.49, 29.46, 29.22, 29.18, 26.59, 22.49, 13.51. Elemental analysis for C_18_H_25_N_5_O_5_ (391.43); calculated C, 55.23; H, 6.44; N, 17.89, found: C, 55.29; H, 6.40; N, 18.01. R_*f*_: 0.61 (S2).

5-(3,5-Dinitrophenyl)-*N*-undecyl-1,3,4-oxadiazol-2-amine **5k**

Yellow solid, yield 80%; mp 134–135°C. IR (ATR): 686, 728, 910, 1035, 1073, 1342 (*ν*_*sym*_ NO_2_), 1353, 1469, 1546 (*ν*_*asym*_ NO_2_), 1570, 1672 (C = N), 2851 (*ν*_*sym*_ C-H aliph.), 2924 (*ν*_*asym*_ C-H aliph.), 3100 (*ν*_*sym*_ C-H arom.) cm^-1^. ^1^H NMR (600 MHz, acetone-*d*_*6*_) *δ* 8.96 (1H, t, *J* = 2.1 Hz, H4), 8.89 (2H, d, *J* = 2.1 Hz, H2, H6), 7.12 (1H, t, *J* = 6.6 Hz, NH-CH_2_-CH_2_), 3.43 (2H, td, *J* = 7.0, 5.6 Hz, NH-CH_2_-CH_2_), 1.71–1.69 (2H, m, NH-CH_2_-CH_2_), 1.44–1.39 (2H, m, C^3^H_2_), 1.33–1.22 (14H, m, C^4^H_2_, C^5^H_2_, C^6^H_2_, C^7^H_2_, C^8^H_2_, C^9^H_2_, C^10^H_2_), 0.84 (3H, t, *J* = 7.3 Hz, CH_3_). ^13^C NMR (151 MHz, acetone) *δ* 164.76, 155.37, 149.29, 127.97, 124.59, 119.09, 43.24, 43.12, 31.80, 29.51, 29.50, 29.48, 29.45, 29.18, 26.59, 22.50, 13.51. Elemental analysis for C_19_H_27_N_5_O_5_ (405.46); calculated C, 56.28; H, 6.71; N, 17.27, found: C, 56.37; H, 6.79; N, 17.32. R_*f*_: 0.61 (S2).

5-(3,5-Dinitrophenyl)-*N*-dodecyl-1,3,4-oxadiazol-2-amine **5l**

Yellow solid, yield 92%; mp 131–133°C. IR (ATR): 685, 728, 911, 1027, 1072, 1135, 1343 (*ν*_*sym*_ NO_2_), 1352, 1469, 1545 (*ν*_*asym*_ NO_2_), 1558, 1568, 1671 (C = N), 2850 (*ν*_*sym*_ C-H aliph.), 2921 (*ν*_*asym*_ C-H aliph.), 3100 (*ν*_*sym*_ C-H arom.) cm^-1^. ^1^H NMR (500 MHz, DMSO-*d*_*6*_): *δ* 8.86 (1H, t, *J* = 2.1 Hz, H4), 8.76 (2H, d, *J* = 2.1 Hz, H2, H6), 8.13 (1H, t, *J* = 5.7 Hz, NH-CH_2_-CH_2_), 3.27 (2H, q, *J* = 6.7 Hz, NH-CH_2_-CH_2_), 1.58 (2H, p, *J* = 7.1 Hz, NH-CH_2_-CH_2_), 1.37-1.20 (18H, m, C^3^H_2_, C^4^H_2_, C^5^H_2_, C^6^H_2_, C^7^H_2_, C^8^H_2_, C^9^H_2_, C^10^H_2_, C^11^H_2_), 0.85 (3H, t, *J* = 6.8 Hz, CH_3_). ^13^C NMR (126 MHz, DMSO): *δ* 164.47, 154.91, 148.89, 127.01, 124.66, 119.33, 42.75, 31.48, 29.23, 29.20, 29.17, 29.16, 28.90, 28.87, 28.85, 26.33, 22.28, 14.12. Elemental analysis for C_20_H_29_N_5_O_5_ (419.48); calculated C, 57.27; H, 6.97; N, 16.70, found: C, 57.34; H, 7.00; N, 16.78. R_*f*_: 0.62 (S2).

5-(3,5-Dinitrophenyl)-*N*-tridecyl-1,3,4-oxadiazol-2-amine **5m**

Yellow solid, yield 75%; mp 126–127°C. IR (ATR): 686, 728, 910, 1033, 1073, 1136, 1322, 1343 (*ν*_*sym*_ NO_2_), 1353, 1470, 1546 (*ν*_*asym*_ NO_2_), 1570, 1670 (C = N), 2851 (*ν*_*sym*_ C-H aliph.), 2921 (*ν*_*asym*_ C-H aliph.), 3100 (*ν*_*sym*_ C-H arom.) cm^-1^. ^1^H NMR (500 MHz, 50°C, DMSO-*d*_*6*_) *δ* 8.87 (1H, t, *J* = 2.1 Hz, H4), 8.78 (2H, d, *J* = 2.0 Hz, H2, H6), 7.99 (1H, t, *J* = 5.8 Hz, NH-CH_2_-CH_2_), 3.29 (2H, q, *J* = 6.6 Hz, NH-CH_2_-CH_2_), 1.60 (2H, p, *J* = 7.1 Hz, NH-CH_2_-CH_2_), 1.33–1.22 (20H, m, C^3^H_2_, C^4^H_2_, C^5^H_2_, C^6^H_2_, C^7^H_2_, C^8^H_2_, C^9^H_2_, C^10^H_2_, C^11^H_2_, C^12^H_2_), 0.85 (3H, t, *J* = 6.9 Hz, CH_3_). ^13^C NMR (126 MHz, 50°C, DMSO) *δ* 164.36, 154.69, 148.75, 126.97, 124.40, 119.01, 42.62, 31.16, 28.91, 28.89, 28.87, 29.85, 28.83, 28.81, 28.67, 28.54, 26.06, 21.93, 13.73. Elemental analysis for C_21_H_31_N_5_O_5_ (433.51); calculated C, 58.18; H, 7.21; N, 16.16, found: C, 58.20; H, 7.25; N, 16.24. R_*f*_: 0.63 (S2).

5-(3,5-Dinitrophenyl)-*N*-tetradecyl-1,3,4-oxadiazol-2-amine **5n**

Yellow solid, yield 82%; mp 127–129°C. IR (ATR): 728, 911, 1028, 1073, 1343 (*ν*_*sym*_ NO_2_), 1470, 1507, 1544 (*ν*_*asym*_ NO_2_), 1569, 1671 (C = N), 2851 (*ν*_*sym*_ C-H aliph.), 2921 (*ν*_*asym*_ C-H aliph.), 3100 (*ν*_*sym*_ C-H arom.) cm^-1^. ^1^H NMR (500 MHz, 50°C, DMSO-*d*_*6*_) *δ* 8.87 (1H, t, *J* = 2.1 Hz, H4), 8.77 (2H, d, *J* = 2.0 Hz, H2, H6), 7.98 (1H, t, *J* = 5.8 Hz, NH-CH_2_-CH_2_), 3.30 (2H, q, *J* = 6.6 Hz, NH-CH_2_-CH_2_), 1.60 (2H, p, *J* = 7.0 Hz, NH-CH_2_-CH_2_), 1.40–1.25 (22H, m, C^3^H_2_, C^4^H_2_, C^5^H_2_, C^6^H_2_, C^7^H_2_, C^8^H_2_, C^9^H_2_, C^10^H_2_, C^11^H_2_, C^12^H_2_, C^13^H_2_), 0.85 (3H, t, *J* = 6.7 Hz, CH_3_). ^13^C NMR (126 MHz, 50°C, DMSO) *δ* 164.36, 154.69, 148.75, 126.98, 124.40, 119.00, 42.63, 31.17, 28.91, 28.90, 28.88, 28.86, 28.84, 28.82, 28.80, 28.67, 28.55, 26.07, 21.93, 13.73. Elemental analysis for C_22_H_33_N_5_O_5_ (447.54); calculated C, 59.04; H, 7.43; N, 15.65, found: C, 58.98; H, 7.50; N, 15.59. R_*f*:_ 0.66 (S2).

5-(3,5-Dinitrophenyl)-*N*-pentadecyl-1,3,4-oxadiazol-2-amine **5o**

Yellow solid, yield 77%; mp 131–133°C. IR (ATR): 686, 728, 911, 1033, 1073, 1343 (*ν*_*sym*_ NO_2_), 1352, 1470, 1546 (*ν*_*asym*_ NO_2_), 1570, 1674 (C = N), 2851 (*ν*_*sym*_ C-H aliph.), 2919 (*ν*_*asym*_ C-H aliph.), 3099 (*ν*_*sym*_ C-H arom.) cm^-1^. ^1^H NMR (500 MHz, 50°C, DMSO-*d*_*6*_) *δ* 8.87 (1H, t, *J* = 2.1 Hz, H4), 8.77 (2H, d, *J* = 2.0 Hz, H2, H6), 7.99 (1H, t, *J* = 5.8 Hz, NH-CH_2_-CH_2_), 3.29 (2H, q, *J* = 6.6 Hz, NH-CH_2_-CH_2_), 1.60 (2H, p, *J* = 7.0 Hz, NH-CH_2_-CH_2_), 1.37–1.24 (24H, m, C^3^H_2_, C^4^H_2_, C^5^H_2_, C^6^H_2_, C^7^H_2_, C^8^H_2_, C^9^H_2_, C^10^H_2_, C^11^H_2_, C^12^H_2_, C^13^H_2_, C^14^H_2_), 0.85 (3H, t, *J* = 6.8 Hz, CH_3_). ^13^C NMR (126 MHz, 50°C, DMSO) *δ* 164.36, 154.69, 148.75, 126.98, 124.40, 119.01, 42.62, 31.17, 28.90, 28.89, 28.87, 28.86, 28.85, 28.84, 28.82, 28.80, 28.67, 28.55, 26.07, 21.93, 13.73. Elemental analysis for C_23_H_35_N_5_O_5_ (461.56); calculated C, 59.85; H, 7.64; N, 15.17, found: C, 59.97; H, 7.71; N, 15.24. R_*f*_: 0.66 (S2).

5-(3,5-Dinitrophenyl)-*N*-hexadecyl-1,3,4-oxadiazol-2-amine **5p**

Yellow solid, yield 88%; mp 126–127°C. IR (ATR): 728, 912, 1032, 1073, 1342 (*ν*_*sym*_ NO_2_), 1353, 1469, 1545 (*ν*_*asym*_ NO_2_), 1569, 1671 (C = N), 2851 (*ν*_*sym*_ C-H aliph.), 2921 (*ν*_*asym*_ C-H aliph.), 3100 (*ν*_*sym*_ C-H arom.) cm^-1^. ^1^H NMR (500 MHz, 50°C, DMSO-*d*_*6*_) *δ* 8.87 (1H, t, *J* = 2.1 Hz, H4), 8.78 (2H, d, *J* = 2.1 Hz, H2, H6), 7.99 (1H, t, *J* = 5.8 Hz, NH-CH_2_-CH_2_), 3.29 (2H, q, *J* = 6.6 Hz, NH-CH_2_-CH_2_), 1.60 (2H, p, *J* = 7.0 Hz, NH-CH_2_-CH_2_), 1.30–1.17 (26H, m, C^3^H_2_, C^4^H_2_, C^5^H_2_, C^6^H_2_, C^7^H_2_, C^8^H_2_, C^9^H_2_, C^10^H_2_, C^11^H_2_, C^12^H_2_, C^13^H_2_, C^14^H_2_, C^15^H_2_), 0.85 (3H, t, *J* = 6.8 Hz, CH_3_). ^13^C NMR (126 MHz, 50°C, DMSO) *δ* 164.36, 154.69, 148.75, 126.97, 124.39, 119.00, 42.62, 31.16, 28.90, 28.89, 28.88, 28.87 28.86, 28.85, 28.84, 28.83, 28.82, 28.66, 28.54, 26.06, 21.92, 13.73. Elemental analysis for C_24_H_37_N_5_O_5_ (475.59); calculated C, 60.61; H, 7.84; N, 14.73, found: C, 60.69; H, 7.80; N, 14.84. R_*f*_: 0.68 (S2).

5-(3,5-Dinitrophenyl)-*N*-heptadecyl-1,3,4-oxadiazol-2-amine **5q**

Yellow solid, yield 70%; mp 118–120°C. IR (ATR): 686, 728, 913, 1036, 1074, 1321, 1343 (*ν*_*sym*_ NO_2_), 1353, 1469, 1539 (*ν*_*asym*_ NO_2_), 1546, 1569, 1674 (C = N), 2851 (*ν*_*sym*_ C-H aliph.), 2921 (*ν*_*asym*_ C-H aliph.), 3100 (*ν*_*sym*_ C-H arom.) cm^-1^. ^1^H NMR (500 MHz, 50°C, DMSO-*d*_*6*_) *δ* 8.87 (1H, t, *J* = 2.1 Hz, H4), 8.78 (2H, d, *J* = 2.0 Hz, H2, H6), 7.99 (1H, t, *J* = 5.8 Hz, NH-CH_2_-CH_2_), 3.29 (2H, q, *J* = 6.6 Hz, NH-CH_2_-CH_2_), 1.60 (2H, p, *J* = 7.1 Hz, NH-CH_2_-CH_2_), 1.37–1.19 (28H, m, C^3^H_2_, C^4^H_2_, C^5^H_2_, C^6^H_2_, C^7^H_2_, C^8^H_2_, C^9^H_2_, C^10^H_2_, C^11^H_2_, C^12^H_2_, C^13^H_2_, C^14^H_2_, C^15^H_2_, C^16^H_2_), 0.85 (3H, t, *J* = 6.8 Hz, CH_3_). ^13^C NMR (126 MHz, 50°C, DMSO) *δ* 164.36, 154.70, 148.76, 126.98, 124.41, 119.02, 42.62, 31.17, 28.90, 28.89, 28.88, 28.87, 28.86, 28.85, 28.84, 28.83, 28.82, 28.81, 28.67, 28.54, 26.07, 21.93, 13.74. Elemental analysis for C_25_H_39_N_5_O_5_ (489.62); calculated C, 61.33; H, 8.03; N, 14.30, found: C, 61.32; H, 8.11; N, 14.22. R_*f*_: 0.68 (S2).

5-(3,5-Dinitrophenyl)-*N*-octadecyl-1,3,4-oxadiazol-2-amine **5r**

Yellow solid, yield 95%; mp 125–126°C. IR (ATR): 689, 731, 913, 1042, 1077, 1343 (*ν*_*sym*_ NO_2_), 1472, 1544 (*ν*_*asym*_ NO_2_), 1571, 1671 (C = N), 2851 (*ν*_*sym*_ C-H aliph.), 2920 (*ν*_*asym*_ C-H aliph.), 3094 (*ν*_*sym*_ C-H arom.) cm^-1^. ^1^H NMR (500 MHz, 50°C, DMSO-*d*_*6*_) *δ* 8.86 (1H, t, *J* = 2.1 Hz, H4), 8.78 (2H, d, *J* = 2.0 Hz, H2, H6), 7.99 (1H, t, *J* = 5.8 Hz, NH-CH_2_-CH_2_), 3.29 (2H, q, *J* = 6.6 Hz, NH-CH_2_-CH_2_), 1.60 (2H, p, *J* = 7.1 Hz, NH-CH_2_-CH_2_), 1.37–1.19 (30H, m, C^3^H_2_, C^4^H_2_, C^5^H_2_, C^6^H_2_, C^7^H_2_, C^8^H_2_, C^9^H_2_, C^10^H_2_, C^11^H_2_, C^12^H_2_, C^13^H_2_, C^14^H_2_, C^15^H_2_, C^16^H_2_, C^17^H_2_), 0.85 (3H, t, *J* = 6.8 Hz, CH_3_). ^13^C NMR (126 MHz, 50°C, DMSO) *δ* 164.36, 154.70, 148.76, 126.98, 124.41, 119.02, 42.62, 31.17, 28.90, 28.89, 28.88, 28.87, 28.86, 28.85, 28.84, 28.83, 28.82, 28.81, 28.80, 28.67, 28.54, 26.07, 21.93, 13.74. Elemental analysis for C_26_H_41_N_5_O_5_ (503.64); calculated C, 62.01; H, 8.21; N, 13.91, found: C, 62.11; H, 8.15; N, 14.01. R_*f*_: 0.70 (S2).

5-(3,5-Dinitrophenyl)-*N*-dodecyl-1,3,4-thiadiazol-2-amine **5s**

Brown solid, yield 76%; mp 138–140°C. IR (ATR): 659, 731, 897, 918, 1075, 1162, 1323, 1340 (*ν*_*sym*_ NO_2_), 1350, 1374, 1473, 1540 (*ν*_*asym*_ NO_2_), 1598, 2856 (*ν*_*sym*_ C-H aliph.), 2925 (*ν*_*sym*_ C-H aliph.), 2958, 3096 (*ν*_*sym*_ C-H arom.) cm^-1^. ^1^H NMR (500 MHz, DMSO-*d*_*6*_) *δ* 8.79 (1H, t, *J* = 2.1 Hz, H4), 8.77 (2H, d, *J* = 2.0 Hz, H2, H6), 8.33 (1H, t, *J* = 5.4 Hz, NH-CH_2_-CH_2_), 3.34 (2H, td, *J* = 7.0, 5.3 Hz, NH-CH_2_-CH_2_), 1.60 (2H, p, *J* = 7.1 Hz, NH-CH_2_-CH_2_), 1.37–1.19 (18H, m, C^3^H_2_, C^4^H_2_, C^5^H_2_, C^6^H_2_, C^7^H_2_, C^8^H_2_, C^9^H_2_, C^10^H_2_, C^11^H_2_), 0.84 (3H, t, *J* = 6.5 Hz, CH_3_). ^13^C NMR (126 MHz, DMSO) *δ* 170.34, 151.64, 148.75, 133.82, 125.60, 118.40, 45.20, 31.47, 29.22, 29.19, 29.15, 28.88, 28.87, 28.85, 28.54, 26.49, 22.26, 14.10. Elemental analysis for C_20_H_29_N_5_O_4_S (435.54); calculated C, 55.15; H, 6.71; N, 16.08, found: C, 55.19; H, 6.80; N, 16.18. R_*f*_: 0.84 (S2).

2-(3,5-Dinitrobenzoyl)-*N*-(4-methoxybenzyl)hydrazine-1-carbothioamide **6b**

Yellow solid, yield: 87% (method B); mp 180–182°C. IR (ATR): 619, 660, 719, 730, 817, 909, 921, 973, 1027, 1077, 1166, 1231, 1244, 1342 (*ν*_*sym*_ NO_2_), 1512, 1541 (*ν*_*asym*_ NO_2_), 1613, 1629, 1677 (*ν* **CO**NH), 1707, 1729, 2963 (*ν* C-H aliph), 3090 (*ν*_*sym*_ C-H arom.), 3113, 3283 (*ν*_*sym*_ N-H) cm^-1^.^1^H NMR (500 MHz, DMSO-*d*_*6*_) *δ* 11.08 (1H, s, NH), 9.61 (1H, s, NH), 9.05 (2H, d, *J* = 2.1 Hz, H2, H6), 8.99 (1H, t, *J* = 2.1 Hz, H4), 8.79 (s, 1H, NH), 7.23 (2H, d, *J* = 8.5 Hz, H2’, H6’), 6.86 (2H, d, *J* = 8.5 Hz, H3’, H5’), 4.69 (2H, d, *J* = 5.9 Hz, CH_2_), 3.71 (3H, s, CH_3_). ^13^C NMR (126 MHz, DMSO) δ 162.61, 158.35, 148.20, 135.56, 131.19, 128.50, 128.20, 121.56, 113.69, 55.24, 46.43. Elemental analysis for C_16_H_15_N_5_O_6_S (405.39); calculated C, 47.41; H, 3.73; N, 17.28, found: C, 47.39; H, 3.80; N, 17.20.

*N*-(4-Chlorobenzyl)-2-(3,5-dinitrobenzoyl)hydrazine-1-carbothioamide **6c**

Yellow solid, yield: 87% (method B); mp 204–206°C. IR (ATR): 702, 719, 731, 810, 848, 917, 1017, 1075, 1093, 1173, 1222, 1272, 1285, 1342 (*ν*_*sym*_ NO_2_), 1491, 1520, 1542 (*ν*_*asym*_ NO_2_), 1562, 1631, 1695 (*ν* **CO**NH), 2985 (*ν* C-H aliph), 3091 (*ν*_*sym*_ C-H arom.), 3144, 3320 (*ν*_*sym*_ N-H) cm^-1^.^1^H NMR (500 MHz, DMSO-*d*_*6*_) *δ* 11.13 (s, 1H, N*H*), 9.71 (s, 1H, N*H*), 9.06 (2H, d, *J* = 2.1 Hz, H2, H6), 9.00 (1H, t, *J* = 2.1 Hz, H4), 8.86 (s, 1H, N*H*), 7.37 (2H, d, *J* = 8.4 Hz, H3’, H5’), 7.31 (2H, d, *J* = 8.4 Hz, H2’, H6’), 4.74 (2H, d, *J* = 6.0 Hz, CH_2_). ^13^C NMR (126 MHz, DMSO) δ 182.33, 162.63, 148.22, 138.42, 135.48, 131.38, 128.97, 128.20, 121.61, 46.25. Elemental analysis for C_15_H_12_ClN_5_O_5_S (409.80); calculated C, 43.96; H, 2.95; N, 17.09, found: C, 44.01; H, 2.90; N, 17.05.

*N*-(2,4-Dichlorobenzyl)-2-(3,5-dinitrobenzoyl)hydrazine-1-carbothioamide **6d**

Yellow solid, yield: 81% (Method B); mp 188–190°C. IR (ATR): 717, 732, 809, 833, 946, 966, 1050, 1074, 1103, 1177, 1236, 1284, 1343 (*ν*_*sym*_ NO_2_), 1474, 1519, 1544 (*ν*_*asym*_ NO_2_), 1558, 1590, 1698 (*ν* **CO**NH), 2980 (*ν* C-H aliph), 3101 (*ν*_*sym*_ C-H arom.), 3158, 3339 (*ν*_*sym*_ N-H) cm^-1^.^1^H NMR (300 MHz, Acetone-*d*_*6*_) *δ* 10.62 (s, 1H, NH), 9.13–9.10 (3H, m, H2, H4, H6), 9.03 (s, 1H, NH), 8.55 (s, 1H, NH), 7.46–7.41 (2H, m, H3’, H5’), 7.35–7.30 (1H, m, H6’), 4.88 (2H, d, *J* = 6.1 Hz, CH_2_). ^13^C NMR (75 MHz, acetone) δ 185.29, 164.02, 149.53, 136.37, 135.91, 133.76, 133.54, 130.65, 129.37, 128.64, 127.86, 122.43, 45.52. Elemental analysis for C_15_H_11_Cl_2_N_5_O_5_S (444.24); calculated C, 40.56; H, 2.50; N, 15.77, found: C, 40.59; H, 2.55; N, 15.70.

*N*-Benzyl-5-(3,5-dinitrophenyl)-1,3,4-oxadiazol-2-amine **7a**

Yellow solid, yield: 50% (Method A); mp 170–172°C. IR (ATR): 690, 728, 920, 1025, 1076, 1318, 1341 (*ν*_*sym*_ NO_2_), 1460, 1506, 1542 (*ν*_*asym*_ NO_2_), 1585, 1652 (C = N), 3091 (*ν*_*sym*_ C-H arom.), 3201 (*ν*_*sym*_ N-H) cm^-1^. ^1^H NMR (500 MHz, DMSO-*d*_*6*_) *δ* 8.86 (1H, t, *J* = 2.0 Hz, H4), 8.76 (2H, d, *J* = 2.0 Hz, H2, H6), 8.70 (1H, t, *J* = 6.2 Hz, NH-CH_2_), 7.47–7.21 (5H, m, C_6_H_5_), 4.51 (2H, d, *J* = 6.2 Hz, CH_2_). ^13^C NMR (126 MHz, DMSO) δ 164.50, 155.22, 148.85, 138.53, 128.56, 127.58, 127.43, 126.91, 124.77, 119.43, 46.22. Elemental analysis for C_15_H_11_N_5_O_5_ (341.28); calculated C, 52.79; H, 3.25; N, 20.52, found: C, 52.39; H, 3.36; N, 20.42. The product was purified by column chromatography (mobile phase: *n-*hexane/ethyl acetate, 4:1 V/V). R_*f*_: 0.44; *n*-hexane/ethyl acetate (1:1 V/V).

5-(3,5-Dinitrophenyl)-*N*-(4-methoxybenzyl)-1,3,4-oxadiazol-2-amine **7b**

Yellow solid, yield 53% (Method C); mp 169–171°C. IR (ATR): 710, 727, 752, 833, 918, 997, 1027, 1079, 1116, 1181, 1254, 1341 (*ν*_*sym*_ NO_2_), 1440, 1514, 1538 (*ν*_*asym*_ NO_2_), 1568, 1613, 1661 (C = N), 2941 (*ν* C-H aliph), 3078 (*ν*_*sym*_ C-H arom.) cm^-1^. ^1^H NMR (500 MHz, DMSO-*d*_*6*_) *δ* 8.86 (1H, t, *J* = 2.1 Hz, H4), 8.76 (2H, d, *J* = 2.1 Hz, H2, H6), 8.61 (1H, t, *J* = 6.0 Hz, NH-CH_2_), 7.32 (2H, d, *J* = 8.6 Hz, H2´, H6´), 6.90 (2H, t, *J* = 8.6 Hz, H3´, H5´), 4.42 (2H, d, *J* = 6,0 Hz, CH_2_), 3.72 (3H, s, OCH_3_). ^13^C NMR (126 MHz, DMSO) *δ* 163.75, 158.02, 154.46, 148.15, 129.74, 128.34, 126.22, 124.04, 118.70, 113.24, 54.53, 45.03. Elemental analysis for C_16_H_13_N_5_O_6_ (371.31); calculated C, 51.76; H, 3.53; N, 18.86, found: C, 51.58; H, 3.73; N, 18.68. The product was purified by column chromatography (mobile phase: *n*-hexane/chloroform, 1:6 V/V). R_*f*_: 0.18; chloroform.

*N*-(4-Chlorobenzyl)-5-(3,5-dinitrophenyl)-1,3,4-oxadiazol-2-amine **7c**

Yellow solid, yield 58% (Method C); mp 187–188°C. IR (ATR): 654, 727, 788, 914, 1014, 1031, 1094, 1077, 1340 (*ν*_*sym*_ NO_2_), 1458, 1494, 1533 (*ν*_*asym*_ NO_2_), 1552, 1660 (C = N), 2936 (*ν* C-H aliph), 3091 (*ν*_*sym*_ C-H arom.) cm^-1^. ^1^H NMR (500 MHz, DMSO-*d*_*6*_) *δ* 8.87 (1H, t, *J* = 2.0 Hz, H4), 8.77 (2H, d, *J* = 2.0 Hz, H2, H6), 8.72 (1H, t, *J* = 6.2 Hz, NH-CH_2_), 7.44-7.37 (4H, m, H2´, H3´, H5´, H6´), 4.50 (2H, d, *J* = 6.2 Hz, CH_2_). ^13^C NMR (126 MHz, DMSO) *δ* 164.39, 155.33, 148.86, 137.62, 132.00, 129.47, 128.52, 126.88, 124.82, 119.50, 45.50. Elemental analysis for C_15_H_10_ClN_5_O_5_ (375.73); calculated C, 47.95; H, 2.68; N, 18.64, found: C, 47.68; H, 2.87; N, 18.25. The product was purified by column chromatography (mobile phase: chloroform). R_*f*_: 0.14; chloroform.

*N*-(2,4-Dichlorobenzyl)-5-(3,5-dinitrophenyl)-1,3,4-oxadiazol-2-amine **7d**

Light beige solid, yield 22% (Method C); mp 180–181°C. IR (ATR): 688, 728, 799, 829, 863, 909, 979, 1032, 1045, 1249, 1307, 1323, 1338 (*ν*_*sym*_ NO_2_), 1426, 1471, 1535 (*ν*_*asym*_ NO_2_), 1549, 1641 (C = N), 2878 (*ν* C-H aliph), 3090 (*ν*_*sym*_ C-H arom.), 3153 (*ν*_*sym*_ N-H) cm^-1^. ^1^H NMR (300 MHz, DMSO-*d*_*6*_) *δ* 8.87 (1H t, *J* = 2.1 Hz, H4), 8.83–8.76 (3H, m, H2, H6, NH), 7.65 (1H, d, *J* = 2.1 Hz, H3´), 7.53 (1H, d, *J* = 8.2 Hz, H6´), 7.44 (1H, dd, *J* = 8.2, 2.1 Hz, H5´), 4.57 (2H, d, *J* = 5.9 Hz, CH_2_). ^13^C NMR (75 MHz, DMSO) *δ* 164.21, 155.49, 148.88, 134.70, 133.50, 132.99, 130.92, 128.98, 127.61, 126.84, 124.89, 119.59, 43.66. Elemental analysis for C_15_H_9_Cl_2_N_5_O_5_ (410.17); calculated C, 43.92; H, 2.21; N, 17.29; N, found: C, 44.21; H, 2.65; N, 17.13; N. Product was purified by column chromatography (mobile phase: *n*-hexane/ethyl acetate, 2:1 V/V). R_*f*_: 0.60; *n*-hexane/ethyl acetate (1:1 V/V).

*N*-(4-Bromobenzyl)-5-(3,5-dinitrophenyl)-1,3,4-oxadiazol-2-amine **7e**

Yellow solid, yield 80% (Method A); mp 185–187°C. IR (ATR): 686, 726, 787, 914, 1011, 1031, 1072, 1141, 1310, 1339 (*ν*_*sym*_ NO_2_), 1384, 1408, 1489, 1531 (*ν*_*asym*_ NO_2_), 1552, 1592, 1657 (C = N), 2875 (*ν* C-H aliph), 2926 (*ν* C-H aliph), 3084 (*ν*_*sym*_ C-H arom.), 3184 (*ν*_*sym*_ N-H) cm^-1^. ^1^H NMR (500 MHz, DMSO-*d*_*6*_) *δ* 8.86 (1H, t, *J* = 2.1 Hz, H4), 8.76 (2H, d, *J* = 2.1 Hz, H2, H6), 8.73 (1H, t, *J* = 6.2 Hz, NH-CH_2_), 7.54 (2H, d, *J* = 8.4 Hz, H3´, H5´), 7.36 (2H, d, *J* = 8.4 Hz, H2´, H6´), 4.49 (2H, d, *J* = 6.2 Hz, CH_2_). ^13^C NMR (126 MHz, DMSO) *δ* 164.38, 155.32, 148.85, 138.04, 131.43, 129.82, 126.87, 124.81, 120.48, 119.49, 45.54. Elemental analysis for C_15_H_10_BrN_5_O_5_ (420.18); calculated C, 42.88; H, 2.40; N, 16.67, found: C, 42.60; H, 2.15; N, 16.70. The product was purified by column chromatography (mobile phase: *n*-hexane/ethyl acetate, 2:1 V/V). R_*f*_: 0.33; *n*-hexane/ethyl acetate (1:1 V/V).

5-(3,5-Dinitrophenyl)-*N*-(4-methoxybenzyl)-1,3,4-thiadiazol-2-amine **8b**

Yellow solid, yield 10% (Method C) or 71% (Method D); mp 210–213°C. IR (ATR): 679, 724, 740, 812, 900, 998, 1035, 1070, 1111, 1129, 1172, 1250, 1276, 1303, 1339 (*ν*_*sym*_ NO_2_), 1416, 1454, 1511, 1541 (*ν*_*asym*_ NO_2_), 1570 (C = N), 2833 (*ν* C-H aliph), 2924 (*ν* C-H aliph), 3052 (*ν*_*sym*_ C-H arom.), 3113 (*ν*_*sym*_ C-H arom.), 3177 (*ν*_*sym*_ N-H) cm^-1^. ^1^H NMR (500 MHz, DMSO-*d*_*6*_) *δ* 8.80–8.74 (4H, m, H2, H4, H6, NH-CH_2_), 7.32 (2H, d, *J* = 8.6 Hz, H2´, H6´), 6.92 (2H, d, *J* = 8.8 Hz, H3´, H5´), 4.49 (2H, d, *J* = 5.5 Hz, CH_2_), 3.73 (3H, s, OCH_3_). ^13^C NMR (126 MHz, DMSO) *δ* 170.17, 158.78, 152.24, 148.74, 133.69, 130.02, 129.29, 125.72, 118.53, 114.03, 55.26, 47.95. Elemental analysis for C_16_H_13_N_5_O_5_S (387.37); calculated C, 49.61; H, 3.38; N, 18.08, found: C, 49.49; H, 3.53; N, 18.07. The product was purified by column chromatography (mobile phase: *n*-hexane/chloroform, 1:6 V/V). R_*f*_: 0.27; chloroform.

*N*-(4-Chlorobenzyl)-5-(3,5-dinitrophenyl)-1,3,4-thiadiazol-2-amine **8c**

Yellow solid, yield 15% (Method C) or 72% (Method D); mp 225–227°C. IR (ATR): 673, 717, 849, 899, 1015, 1057, 1075, 1135, 1282, 1325, 1342 (*ν*_*sym*_ NO_2_), 1411, 1449, 1492, 1539 (*ν*_*asym*_ NO_2_), 1577 (C = N), 2863 (*ν* C-H aliph), 3050 (*ν*_*sym*_ C-H arom.), 3095 (*ν*_*sym*_ C-H arom.), 3139 (*ν*_*sym*_ N-H) cm^-1^. ^1^H NMR (500 MHz, DMSO-*d*_*6*_) *δ* 8.86 (1H, t, *J* = 5.8 Hz, NH-CH_2_), 8.81–8.77 (3H, m, H2, H4, H6), 7.44–7.37 (4H, m, H2´, H3´, H5´, H6´), 4.58 (2H, d, *J* = 5.8 Hz, CH_2_). ^13^C NMR (126 MHz, DMSO) *δ* 170.11, 152.58, 148.74, 137.37, 133.60, 132.02, 129.66, 128.58, 125.80, 118.63, 47.56. Elemental analysis for C_15_H_10_ClN_5_O_4_S (391.79); C, 45.99; H, 2.57; N, 17.88, found: C, 46.21; H, 2.59; N, 18.19. The product was purified by column chromatography (mobile phase: chloroform). R_*f*_: 0.17; chloroform.

*N*-(2,4-Dichlorobenzyl)-5-(3,5-dinitrophenyl)-1,3,4-thiadiazol-2-amine **8d**

Yellowish solid, yield 4% (Method C) or 74% (Method D); mp 230–232°C. IR (ATR): 680, 719, 727, 824, 860, 923, 957, 992, 1043, 1071, 1111, 1281, 1349 (*ν*_*sym*_ NO_2_), 1420, 1483, 1502, 1533, 1545 (*ν*_*asym*_ NO_2_), 1588 (C = N), 3010 (*ν*_*sym*_ C-H arom.), 3097 (*ν*_*sym*_ C-H arom.), 3191 (*ν*_*sym*_ N-H) cm^-1^. ^1^H NMR (300 MHz, DMSO-*d*_*6*_) *δ* 8.85 (1H, t, *J* = 5.7 Hz, NH-CH_2_), 8.81–8.78 (3H, m, H2, H4, H6), 7.64 (1H, dd, *J* = 2.1, 1.0 Hz, H6´), 7.50 (1H, d, *J* = 8.3 Hz, H3´), 7.44 (1H, dd, *J* = 8.3, 2.1 Hz, H5´), 4.64 (2H, d, *J* = 5.6 Hz, CH_2_). ^13^C NMR (126 MHz, DMSO) *δ* 169.73, 152.94, 148.75, 134.63, 133.72, 133.59, 133.01, 131.14, 129.02, 127.62, 125.88, 118.72, 45.55. Elemental analysis for C_15_H_9_Cl_2_N_5_O_4_S (426.23); C, 42.27; H, 2.13; N, 16.43, found: C, 42.62; H, 2.36; N, 16.57. The product was purified by column chromatography (mobile phase: *n*-hexane/ethyl acetate, 2:1 V/V). R_*f*_ 0.79; *n*-hexane/ethyl acetate (1:1 V/V).

2-(3,5-Dinitrophenyl)-5-(dodecylthio)-1,3,4-oxadiazole **10**

Brown solid, yield 67%; mp 70–71°C. IR (ATR): 730, 737, 755, 912, 1078, 1138, 1203, 1319, 1344, 1469, 1544, 1574, 1602, 2851, 2916, 3087 cm^-1^. ^1^H NMR (500 MHz, DMSO-*d*_*6*_) *δ* 8.98 (1H, t, *J* = 2.0 Hz, H4), 8.95 (2H, d, *J* = 2.1 Hz, H2, H6), 3.36 (2H, t, *J* = 7.3 Hz, S-CH_2_-CH_2_), 1.79 (2H, p, *J* = 7.4 Hz, S-CH_2_-CH_2_), 1.46–1.37 (2H, m, C^3^H_2_), 1.32–1.18 (16H m, C^4^H_2_, C^5^H_2_, C^6^H_2_, C^7^H_2_, C^8^H_2_, C^9^H_2_, C^10^H_2_, C^11^H_2_), 0.84 (3H, t, *J* = 6.9 Hz, CH_3_). ^13^C NMR (126 MHz, DMSO) *δ* 166.04, 162.55, 148.88, 126.32, 126.04, 121.04, 32.29, 31.45, 29.18, 29.16, 29.10, 29.07, 29.02, 28.86, 28.55, 27.98, 22.25, 14.09. Elemental analysis for C_20_H_28_N_4_O_5_S (436.53); calculated C, 55.03; H, 6.47; N, 12.83, found: C, 55.11; H, 6.52; N, 12.92. R_*f*_: 0.66; CH_2_Cl_2_.

### Antimycobacterial activity

#### *In vitro* antimycobacterial activity.

The *in vitro* antimycobacterial activity was evaluated using a previously published method [48] against drug-susceptible *Mtb* 331/88 (i.e., H_37_Rv; dilution of this strain was 10^−3^), *Mycobacterium avium* 330/88 (resistant to INH, rifamycines, EMB and ofloxacin; dilution of the strain was 10^−5^) and a clinical isolate of *Mycobacterium kansasii* (6509/96; dilution of the strain was 10^−4^). The micro method for determining minimum inhibitory concentration (MIC) involved Šula’s semisynthetic medium (SEVAC, Prague, Czech Republic). The derivatives were added to the medium as solutions in DMSO; the final volume contained 1.0% DMSO (v/v). The following concentrations were used: 1000, 500, 250, 125, 62.5, 32, 16, 8, 4, 2, 1, 0.5, 0.25, 0.125, 0.06, and 0.03 μM. The MIC values were determined after incubation at 37°C for 14 and 21 days, for *M. kansasii*, additionally for seven days. MIC (in μM) was the lowest concentration producing the complete inhibition of bacterial growth. First-line oral drug INH was involved as a reference drug.

The selected derivatives were evaluated against seven drug-resistant tuberculous strains (dilution of 10^−3^) with different resistance patterns. All of the strains were resistant to INH, RIF, rifabutin, and STM, and an additional resistance was present in some cases: strain 7357/1998 was resistant additionally to EMB and ofloxacin; strain 234/2005 to EMB; strain 8666/2010 resistant to EMB, ofloxacin, and clofazimine; Praha 1 was resistant to EMB and clofazimine; Praha 4 to EMB, ofloxacin, and clofazimine (all MDR-TB strains); and Praha 131 to EMB, ofloxacin, gentamicin and amikacin (i.e., XDR-TB strain according to former WHO definition). The following concentrations were used for drug-resistant strains: 1000, 500, 250, 125, 62.5, 32, 16, 8, 4, 2, 1, 0.5, 0.25, 0.125, 0.06, and 0.03 μM.

#### Investigation of mode of action.

The mechanism of action was analysed by metabolic labeling of *Mtb* H_37_Rv strain with ^14^C acetate. *Mtb* H_37_Rv culture was cultured in 7H9 broth supplemented with 10% albumin–dextrose–catalase and 0.05% Tween 80 at 37°C until OD_600_ reached ≈ 0.2. The studied compounds were dissolved in DMSO and added in final concentrations corresponding to their 0 × , 10 × , and 100 × MIC values for *Mtb* H_37_Rv. The final concentration of DMSO was kept at 2%. ^14^C acetate was added to each of the cultures to reach a final concentration of 0.5 µCi/mL at the same time, and after 24 h of cultivation, the cells were harvested, and lipid analysis was performed.

The lipids from the cells grown in 100 µL cultures were extracted with 1.5 mL chloroform/methanol (1:2 V/V) at 65°C for two hours. Subsequently, 150 µL of water was added to each sample; the samples were mixed and centrifuged at room temperature, 1,000 × g. The organic phases were transferred to 2 mL tubes, dried under nitrogen flow, and subjected to biphasic Folch washing [49]. Isolated lipids were dissolved in 30 μL of chloroform/methanol (2:1 V/V) and 5 μL were loaded on TLC silica gel plates F254 (Merck). Lipids were separated in chloroform/methanol/water mixture (20:4:0.5 V/V/V) and visualized using an Amersham^TM^ Typhoon^TM^ Biomolecular Imager.

Analysis of susceptibility of *Mtb* H_37_Ra strains overproducing DprE1/DprE2. These proteins were overproduced in *Mtb* H_37_Ra using pVV2-*dprE2* and pVV2-*dprE1*/*dprE2* constructs [50]. The production of recombinant proteins was confirmed by western blot using antiHis antibodies. To determine the susceptibility of *Mtb* H_37_Ra overproducing strains, along with control strain carrying empty vector pVV2, the selected derivatives **4h**, **4s**, **5g**, and **5s** were evaluated by determining MIC values using the drop dilution method. The cultures grown in 7H9 broth supplemented with albumin–dextrose–catalase and 0.05% Tween 80 were adjusted to optical density (O.D._600_) ~ 0.5, and 4 μL of 1×, 10×, and 100× dilutions from each culture were dropped on 7H11 broth supplemented with oleic acid–albumin–dextrose–catalase and various concentrations of tested compounds dissolved in DMSO (2% final concentration). Plates were incubated for 28 days at 37°C.

### Toxicity assessment

#### Cytotoxicity evaluation.

The human hepatocellular liver carcinoma cell line HepG2 [51] (ATCC HB-8065) purchased directly from Health Protection Agency Culture Collections (ECACC, Salisbury, UK; passage 8–10; the catalogue number 85011430) was cultured in minimum essentials eagle medium (Sigma-Aldrich, St. Louis, MO, USA) supplemented with 10% foetal bovine serum, 1% L-glutamine solution (Sigma-Aldrich) and nonessential amino acid solution (Sigma-Aldrich) in a humidified atmosphere containing 5% CO_2_ at 37°C.

The cells were harvested for subculturing after trypsin/EDTA (Sigma-Aldrich) treatment at 37°C. To evaluate the cytotoxicity of the compounds, the cells treated with them were used as experimental groups, whereas untreated HepG2 cells served as controls.

The cells were seeded in a density of 15,000 cells per well in a 96-well plate. The next day, the cells were treated with each tested substance at a broad range of concentrations in triplicates; the compounds were dissolved in DMSO (maximal incubation concentration of DMSO was 1% V/V). The controls representing 100% cell viability, 0% cell viability (the cells treated with 10% DMSO), no cell control, and vehiculum controls were incubated in parallel. After 24 h of incubation in a humidified atmosphere containing 5% CO_2_ at 37°C, the reagent from the kit CellTiter 96 AQueous One Solution Cell Proliferation Assay (CellTiter 96; PROMEGA, Fitchburg, WI, USA) was added. After incubation at 37°C (2 hours), the absorbance of samples was recorded at 490 nm (TECAN, Infinita M200, Grödig, Austria). A standard toxicological parameter IC_50_ was calculated by nonlinear regression from a semilogarithmic plot of incubation concentration versus the percentage of absorbance relative to untreated controls using GraphPad Prism 8 software (GraphPad Software, Inc., La Jolla, CA, USA).

The results of the experiments are presented as inhibitory concentrations that reduce the viability of the cell population to 50% from the maximal viability (IC_50_). Parent INH was involved as a reference agent.

### *In vivo* toxicity testing using *Danio rerio*

Fish maintenance. A day before the experiment, adult wild-type zebrafish (*Danio rerio*) were transferred into plastic spawning cages at the end of the 14-hour photoperiod. All fish were spawned at the onset of the following photoperiod. Fertilized eggs were collected within 30 minutes post-fertilization and inspected for health state and developmental stage using a stereomicroscope before exposure to **5g** and **4h**. Tested compounds were dissolved in DMSO; its concentration in 1 mM stock solution was 0.5%.

Fish embryo test (FET). Fish embryo tests were carried out using six different concentrations (0.001, 0.01, 0.1, 1, 10, and 100 μM) of each of the tested substances and controls over 96 hours at a constant temperature of 26.9°C (±1°C) under semi-static experimental set-up. The used medium was E3 according to Cold Spring Harbor Protocols (5 mM NaCl, 0.17 mM KCl, 0.33 mM CaCl_2_, 0.33 mM MgSO_4_). Each FET consists of 24 embryos per concentration, where each embryo is considered an individual replicate. Before the tests, all wells in the testing culture well plates were saturated by overnight incubation with corresponding concentrations of **5g** and **4h**. Before loading the embryos into the well plates, all solutions were replaced by freshly aerated ones with corresponding concentrations or controls. All solutions in the well plates were changed again after 48 hours of exposure to minimize a potential reduction in oxygen levels and compound concentration. Fish embryos were inspected and scored for mortality, morphological changes, or hatching success rates at 24-, 48-, 72-, and 96-hours post-exposure (hpe) under an inverted microscope (Olympus IX71; Olympus Czech Group, s.r.o., Czech Republic). The FET was considered valid if less than 90% of the embryos in the negative control (artificial water) displayed no mortality or morphological changes. In comparison, > 90% of the embryos in the positive control (3,4-dichloroaniline) were considered dead at the end of the 96-hour exposure. Morphological endpoints in our analysis were: (1) coagulation of fertilised eggs or developing embryos; (2) lack of somite formation; (3) lack of detachment of the tail bud from the yolk sac; (4) lack of heartbeat or heart defects (oedema); (5) malformations of body parts (head and tail region); (6) growth retardation. All morphological endpoints were used to determine the mortality (a positive endpoint means the embryo was considered dead).

For both compounds, the negative controls involved pure E3 medium and E3 medium containing 0.05% DMSO.

### Ethics approval

We confirm that all the research meets ethical guidelines and adheres to the legal requirements of the study country. The University of Jan Evangelista Purkyně is a certified facility for the use of animals in research (Veterinary Approval Number CZ 42760032, Ministry of Agriculture of the Czech Republic Approval Number MZE-19331/2022–13143). The experimental project was approved by the Ministry of Education of the Czech Republic (Approval Number: MSMT-8474/2018–3) and by the Institutional Committee for Animal Welfare. ML and ZŠ are certified for planning and performing experiments on animals. In addition, zebrafish (*Danio rerio*) larvae up to 120 hours post-fertilization (hpf) are not considered live animals under European Union legislation.

## Conclusions

This study describes a series of novel *N*-substituted 5-(3,5-dinitrophenyl)-1,3,4-oxadiazol-2-amines, their precursors, and isosteres with potent *in vitro* antimycobacterial activity that were designed based on a molecular hybridization approach. Various synthetic approaches were employed to achieve good to excellent yields. These compounds were investigated as potential broad-spectrum antimycobacterial agents.

The compounds of all structural motifs showed outstanding antimycobacterial activity against all the strains used: drug-susceptible *Mtb*, MDR-TB, *M. kansasii*, and *M. avium* with MIC values as low as ≤ 0.03 µM (**5f**-**5i**, **5m**, **7b**, **8b**, and **8c**) and no cross-resistance to established drugs. MIC values for many compounds were lower than for the highly potent oral drug isoniazid. Oxadiazoles and thiadiazoles were more active than their acyclic synthetic precursors. Focusing on *N*-substituent, C_6_-C_9_ alkyls or substituted benzyl were the best choices. Four compounds (**4h**, **4s**, **5g**, and **5s**) were subjected to investigation of the mechanism of action. They all inhibited epimerase DprE1, which is involved in the biosynthesis of arabinogalactan essential for mycobacterial cell wall formation, thus confirming a mechanism different from that of clinically used drugs. Both acyclic precursors and aimed oxa- and thiadiazoles showed high selectivity for all mycobacterial strains compared to the eukaryotic cell line (with one exception, SI values were ≥25 up to >3,333). Two compounds (**4h** and **5g**) were investigated *in vivo* in the *Danio rerio* model; they were also selective in this assay.

Taken together, we report the series of promising, highly potent, broad-spectrum, and selective antimycobacterial agents with a mechanism of action distinct from other drugs that merit further investigation.

## Supporting information

S1 File ^1^H, ^13^C NMR, and IR spectra of the target compounds. (PDF)

S2 raw image Autoradiograms. (PDF)
