# Supplementary material for: 5-(3,5-Dinitrophenyl)-1,3,4-oxadiazol-2-amine derivatives, their precursors, and analogues: Synthesis and evaluation of novel highly potent antitubercular agent
Source: PLoS One. 2025 May 29;20(5):e0324608. doi: 10.1371/journal.pone.0324608 (PMC12121777; doi:10.1371/journal.pone.0324608)
Supplement: S1 File — (PDF) [file pone.0324608.s001.pdf]

Supporting information

5-(3,5-Dinitrophenyl)-1,3,4-oxadiazol-2-amine derivatives, their precursors, and analogues: Synthesis and evaluation of novel highly potent antitubercular agents

Václav Pflégr[1], Jiřina Stolaříková[2], Galina Karabanovich[1], Jana Maixnerová[3], Adrián Pál[4], Jana Korduláková[4], Zuzana Šanderová[5], Michaela Liegertová[5], Jaroslav Roh[1], František Trejtnar[3], Jarmila Vinšová[1] and Martin Krátký[1,*]

[1] Department of Organic and Bioorganic Chemistry, Faculty of Pharmacy in Hradec Králové, Charles University, Hradec Králové, Czech Republic.

[2] Laboratory for Mycobacterial Diagnostics and Tuberculosis, Regional Institute of Public Health in Ostrava, Ostrava, Czech Republic

[3] Department of Pharmacology and Toxicology, Faculty of Pharmacy in Hradec Králové, Charles University, Hradec Králové, Czech Republic

[4] Department of Biochemistry, Faculty of Natural Sciences, Comenius University in Bratislava, Bratislava, Slovakia

[5] Centre of Nanomaterials and Biotechnology, Faculty of Science, Jan Evangelista Purkyně University in Ústí nad Labem, Ústí nad Labem, Czech Republic

* Corresponding author

E-mail: martin.kratky@faf.cuni.cz (MK)

1. ^{1}H, ^{13}C NMR and IR spectra of the target compounds

1. ^{1}H, ^{13}C NMR and IR spectra of the target compounds

2-(3,5-Dinitrobenzoyl)-*N*-methylhydrazine-1-carboxamide **4a**

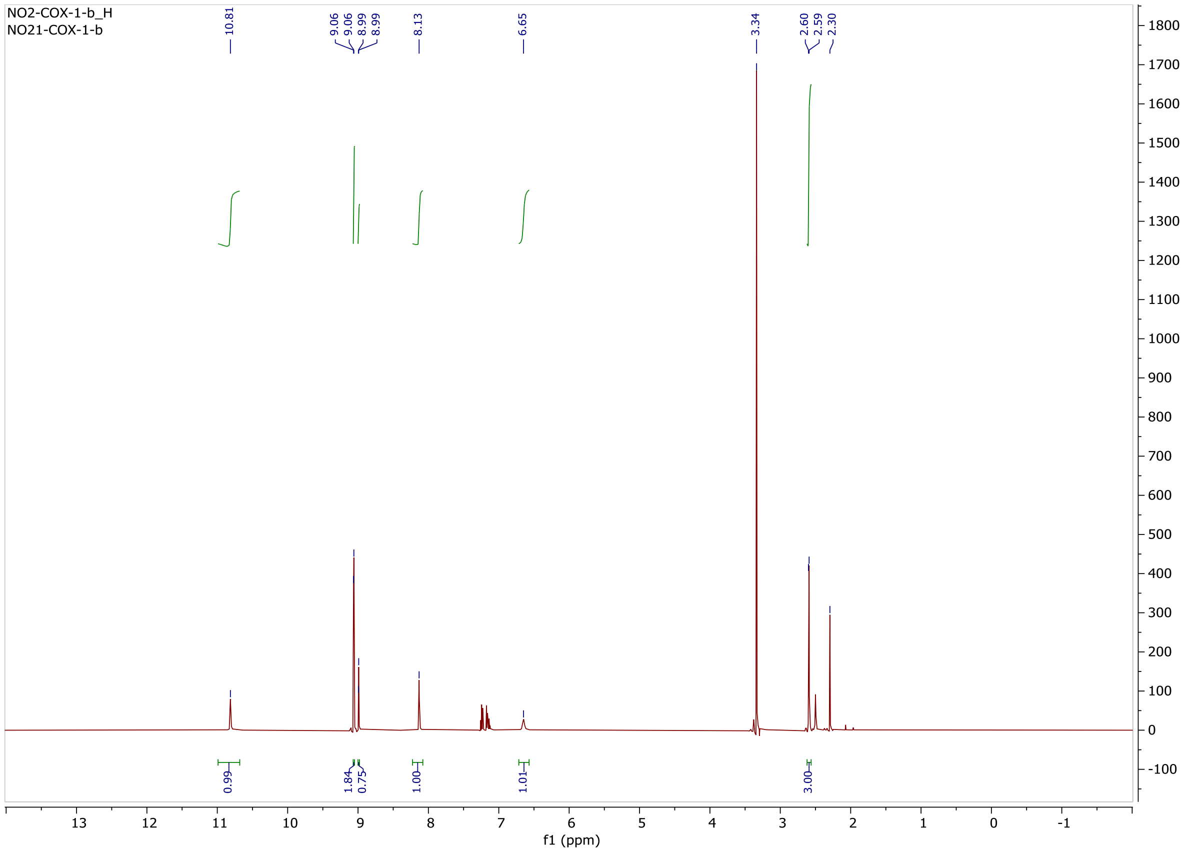

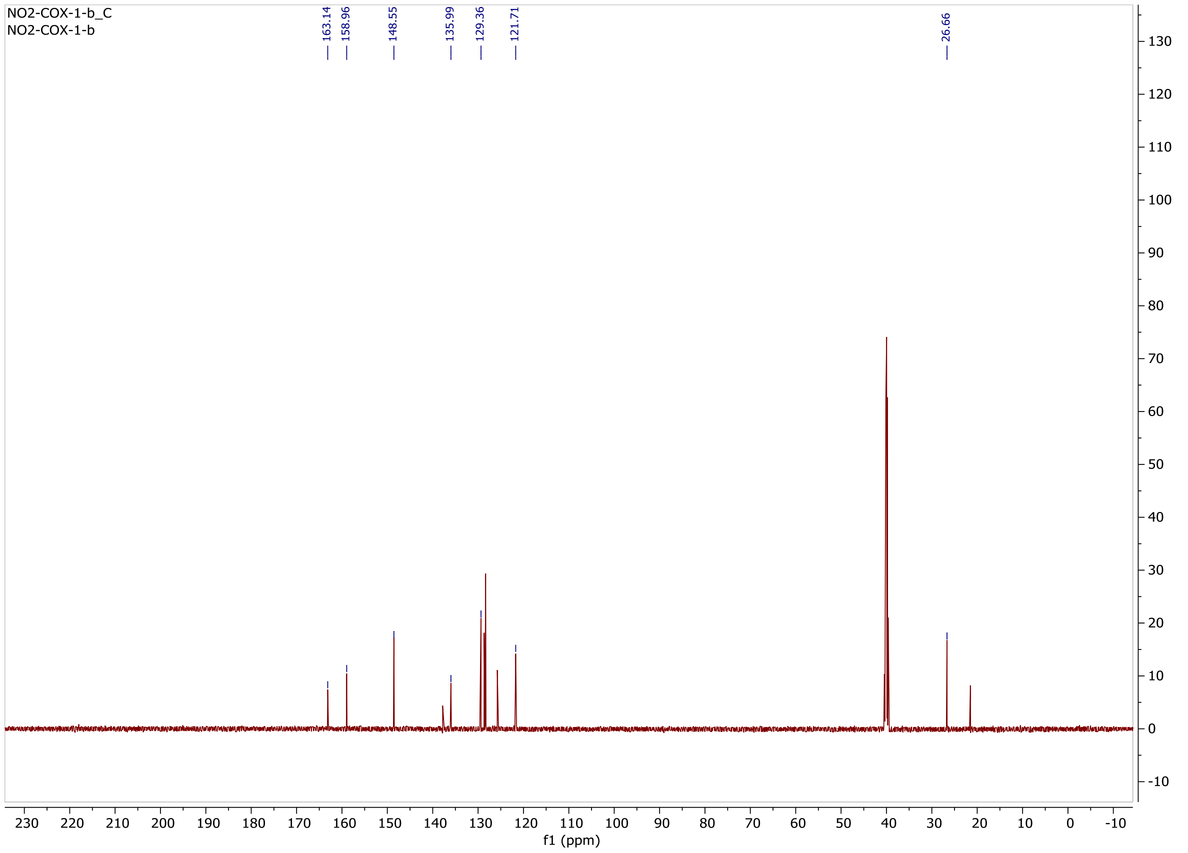

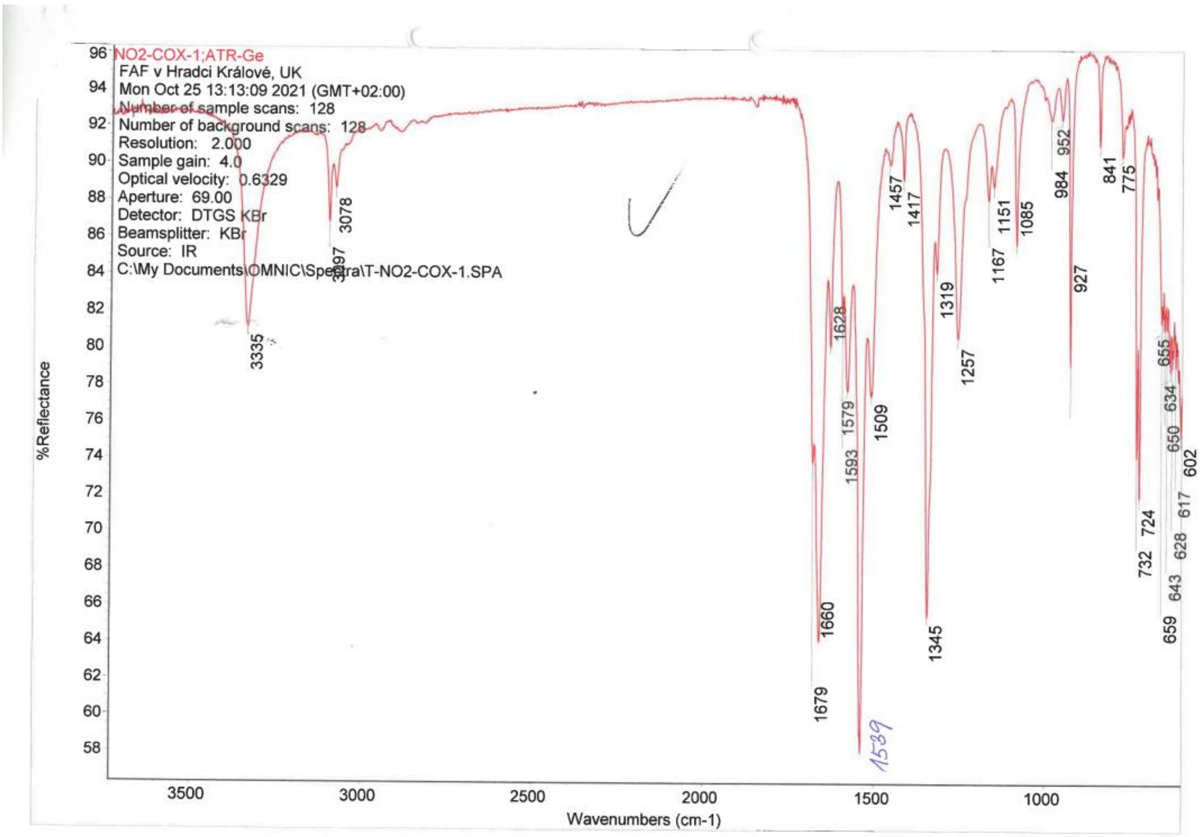

2-(3,5-Dinitrobenzoyl)-*N*-ethylhydrazine-1-carboxamide **4b**

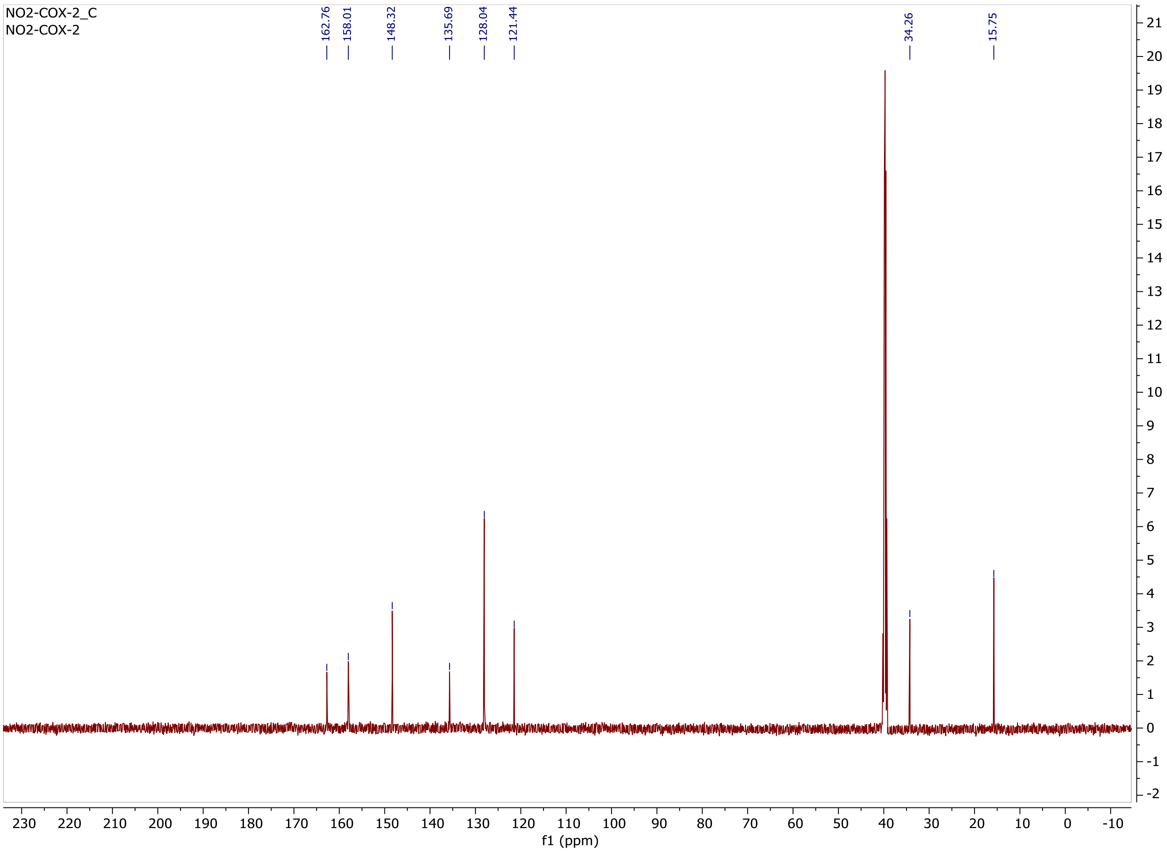

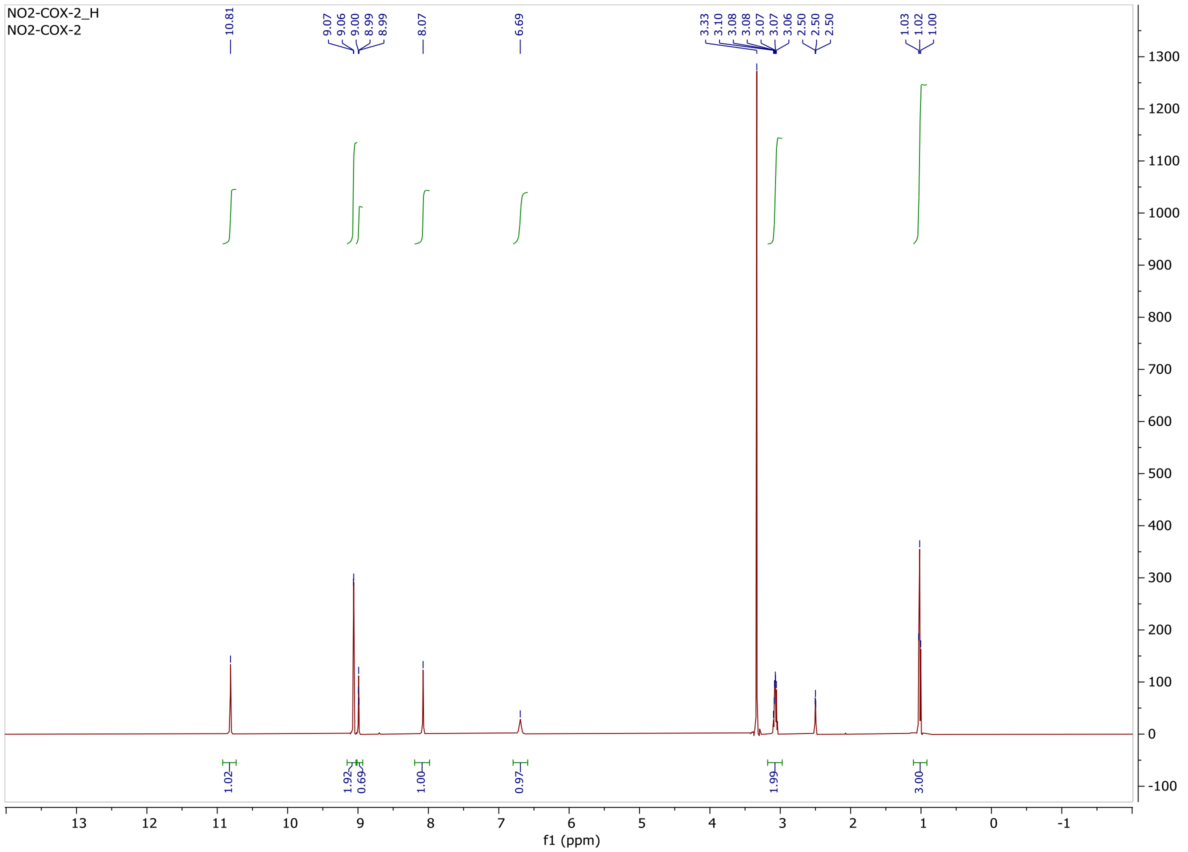
NO2-COX-2_H
NO2-COX-2
10.81
9.07
9.06
9.00
8.99
8.99
8.07
6.69
3.33
3.10
3.08
3.08
3.07
3.07
3.06
2.50
2.50
2.50
1.03
1.02
1.00
1.02
1.92
0.69
1.00
0.97
1.99
3.00
f1 (ppm)

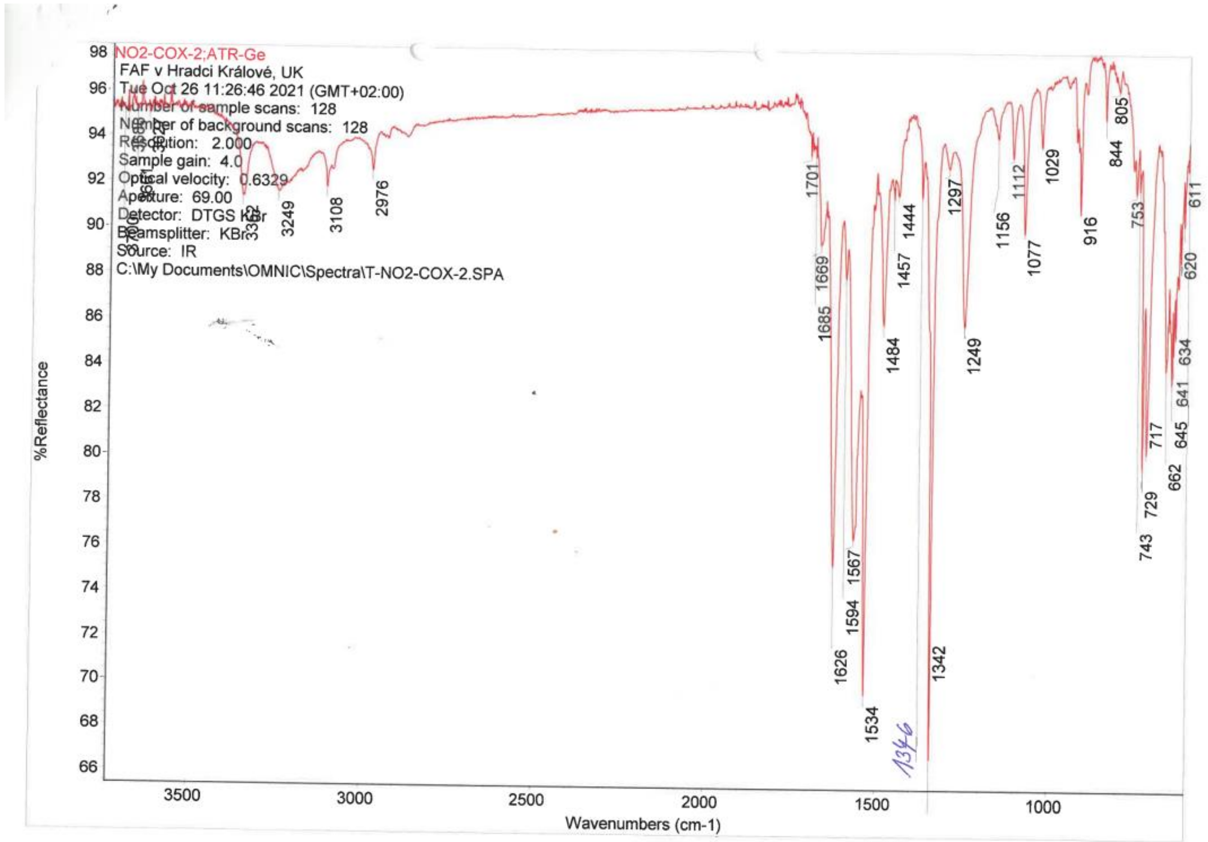
NO2-COX-2;ATR-Ge
FAF v Hradci Králové, UK
Tue Oct 26 11:26:46 2021 (GMT+02:00)
Number of sample scans: 128
Number of background scans: 128
Resolution: 2.000
Sample gain: 4.0
Optical velocity: 0.6329
Aperture: 69.00
Detector: DTGS KBr
Beamsplitter: KBr
Source: IR
C:\My Documents\OMNIC\Spectra\T-NO2-COX-2.SPA
%Reflectance
Wavenumbers (cm-1)
3249
3108
2976
1701
1669
1685
1626
1594
1567
1534
1346
1342
1484
1457
1444
1297
1249
1156
1112
1077
1029
916
844
805
753
743
729
717
662
645
641
634
620
611

2-(3,5-Dinitrobenzoyl)-*N*-propylhydrazine-1-carboxamide **4c**

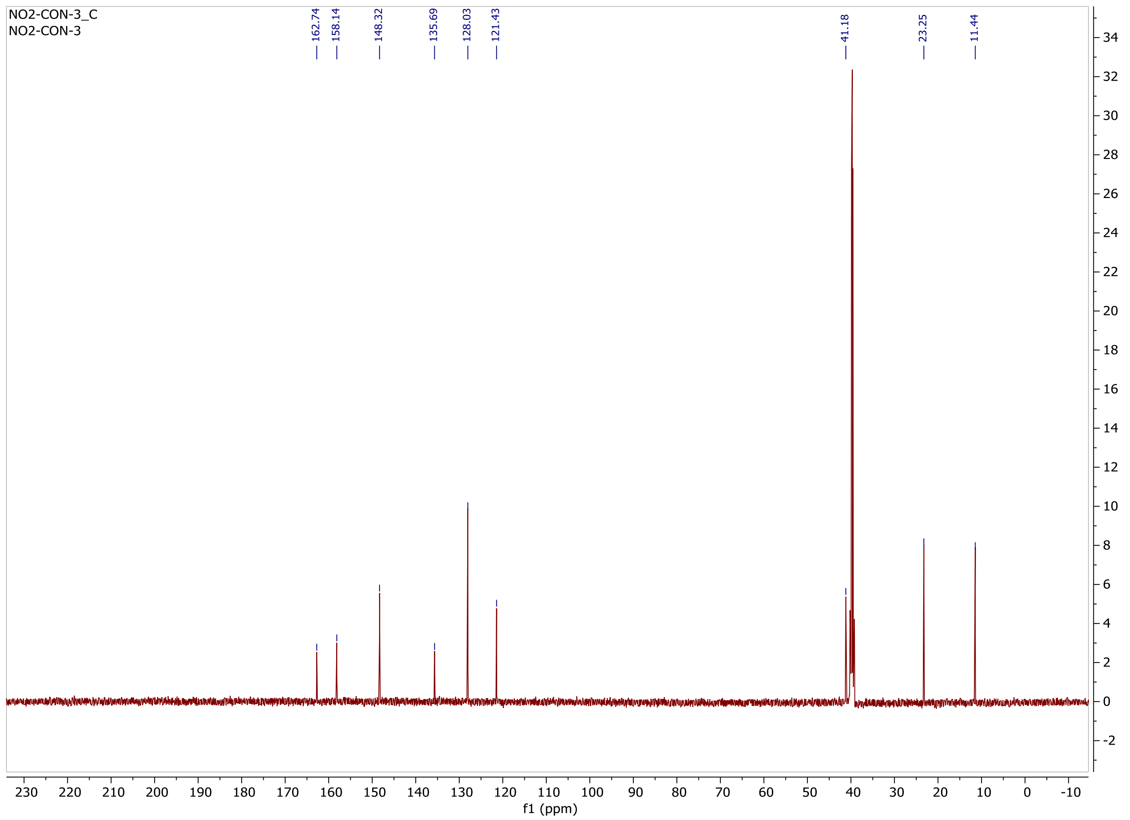

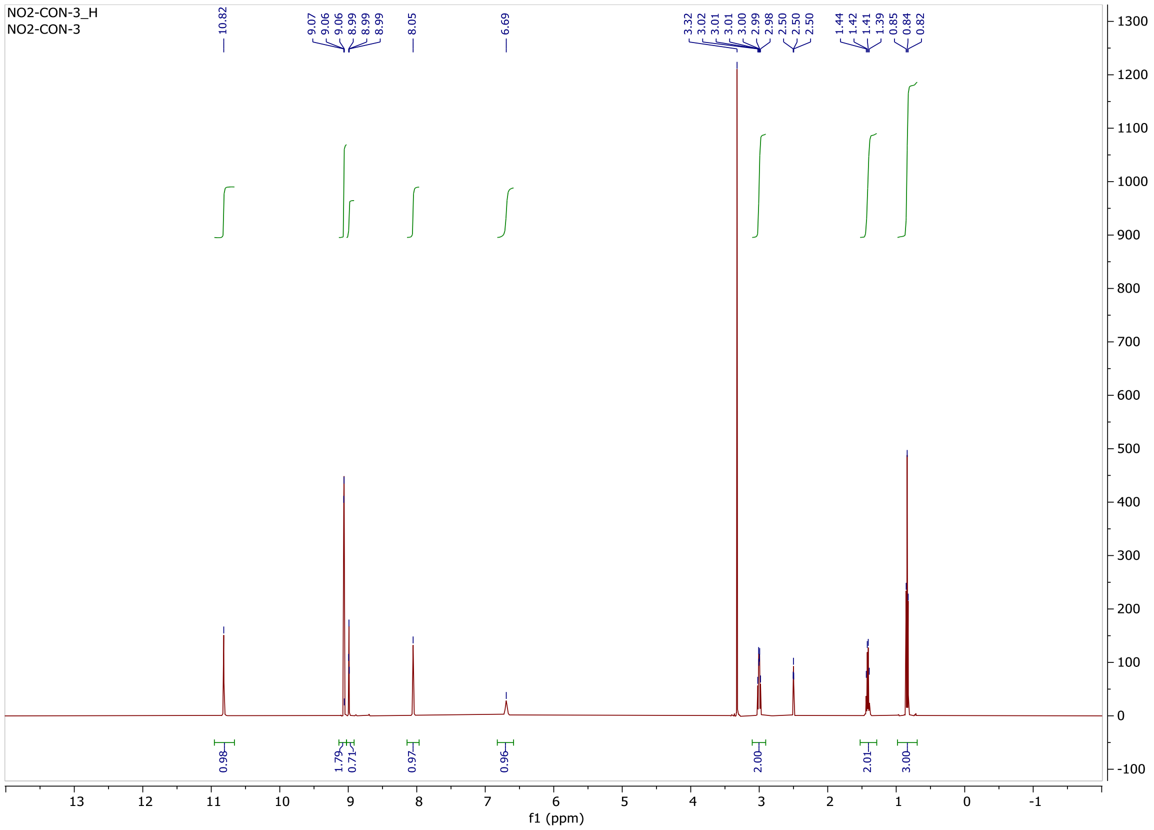

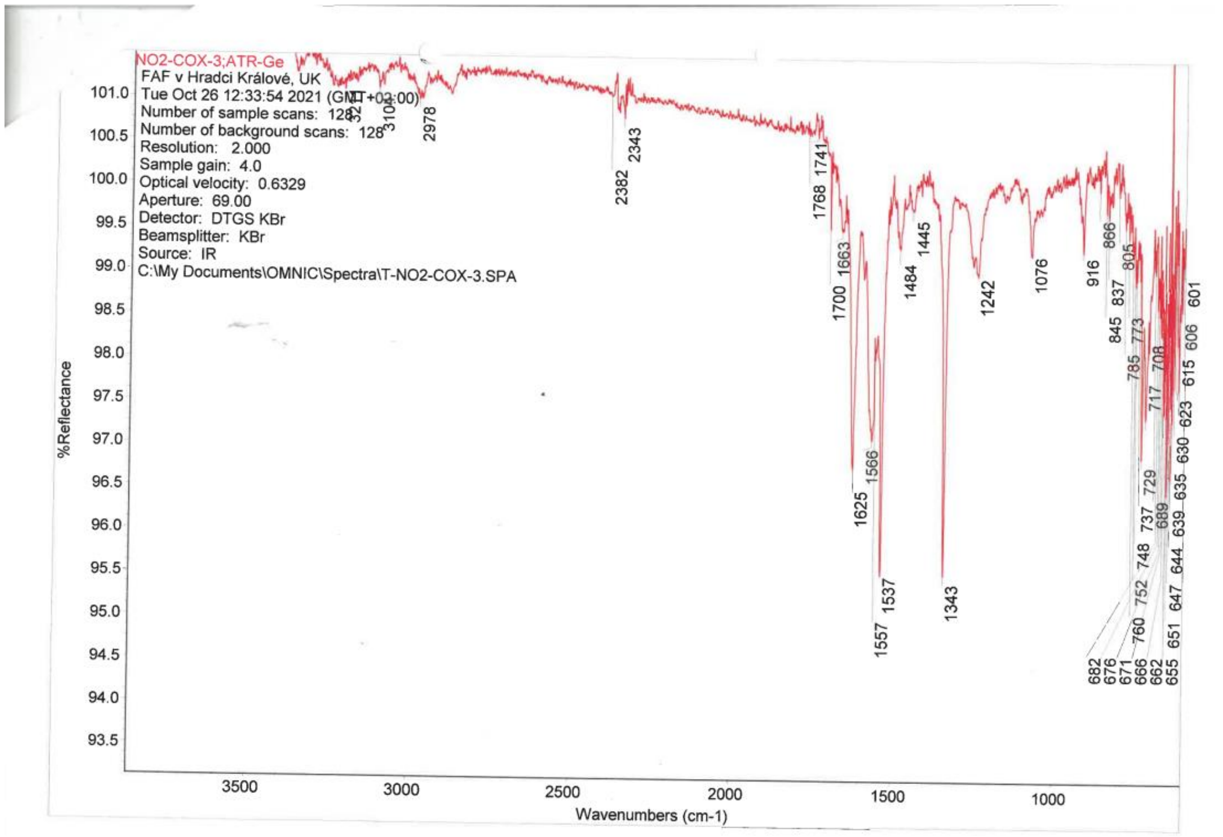

N-Butyl-2-(3,5-dinitrobenzoyl)hydrazine-1-carboxamide **4d**

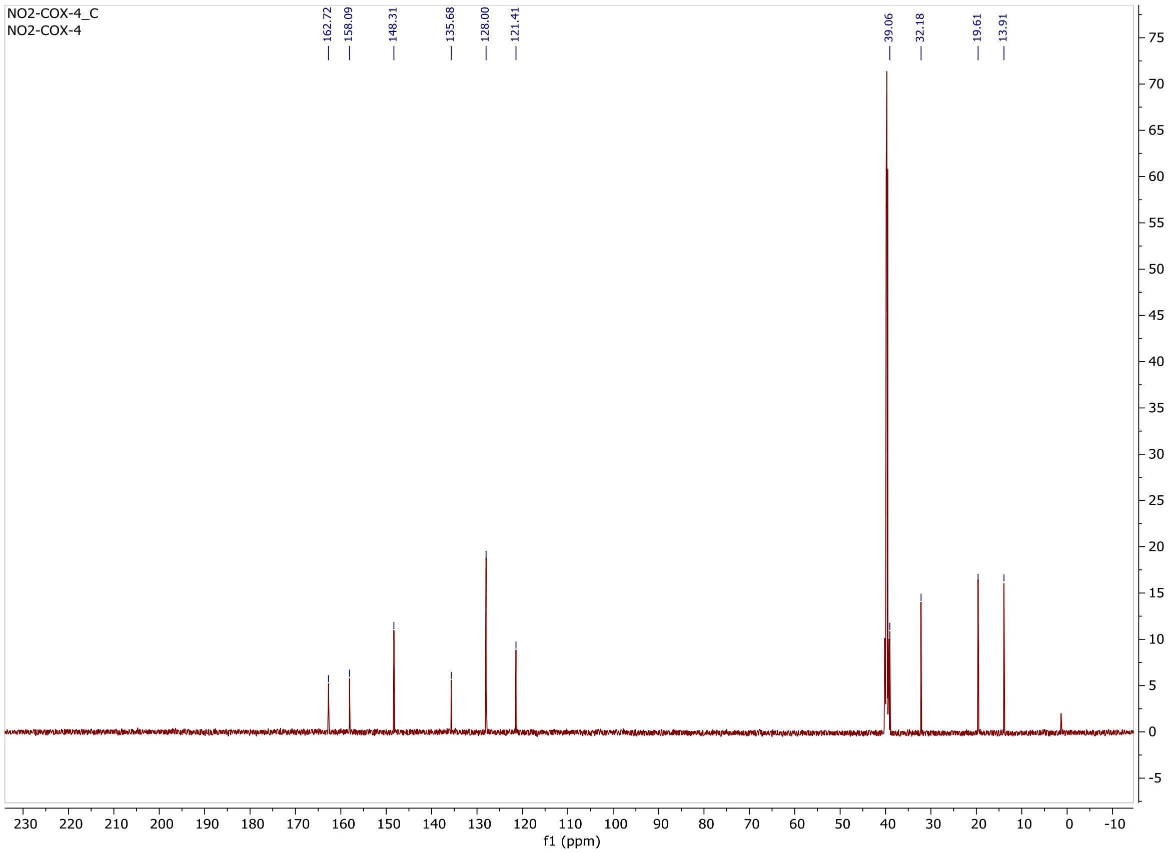

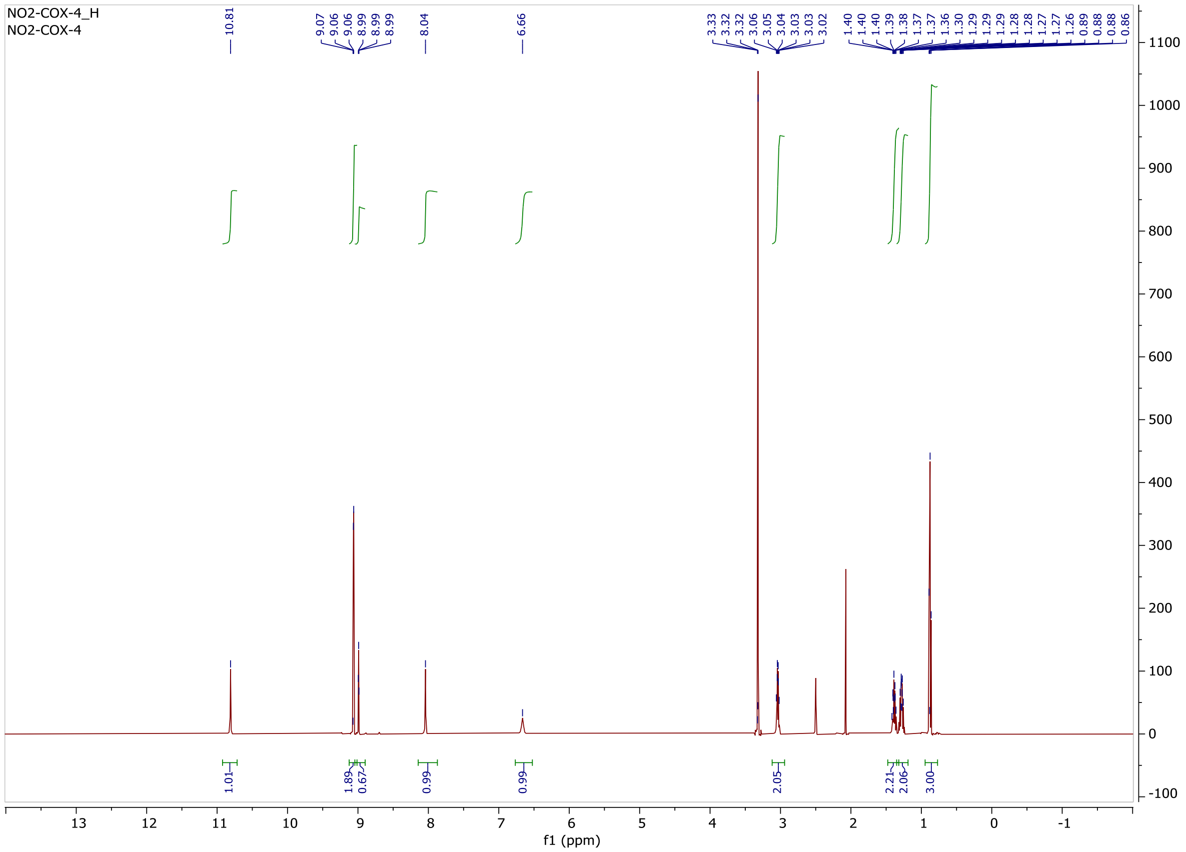
NO2-COX-4_H
NO2-COX-4
f1 (ppm)

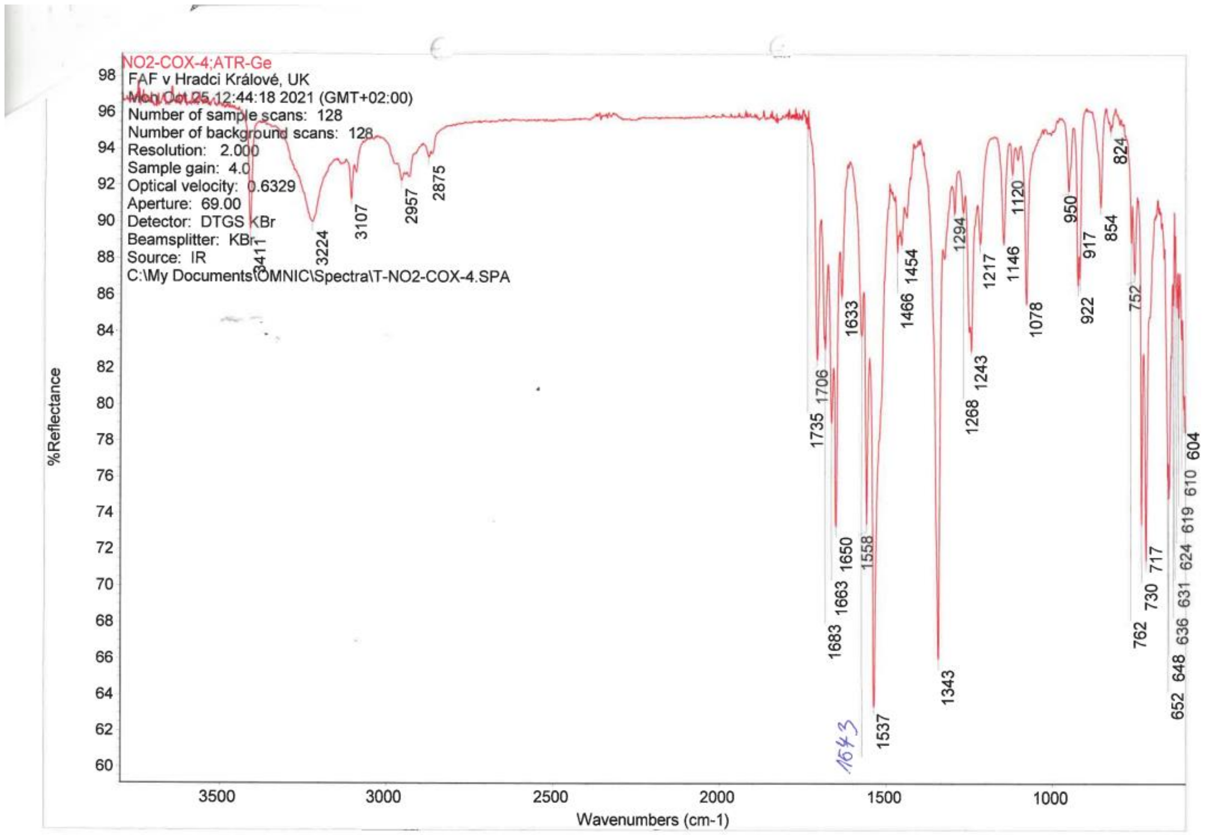
NO2-COX-4:ATR-Ge
FAF v Hradci Králové, UK
Mon Oct 25 12:44:18 2021 (GMT+02:00)
Number of sample scans: 128
Number of background scans: 128
Resolution: 2.000
Sample gain: 4.0
Optical velocity: 0.6329
Aperture: 69.00
Detector: DTGS KBr
Beamsplitter: KBr
Source: IR
C:\My Documents\OMNIC\Spectra\T-NO2-COX-4.SPA
%Reflectance
Wavenumbers (cm-1)

2-(3,5-Dinitrobenzoyl)- *N*-pentylhydrazine-1-carboxamide **4e**

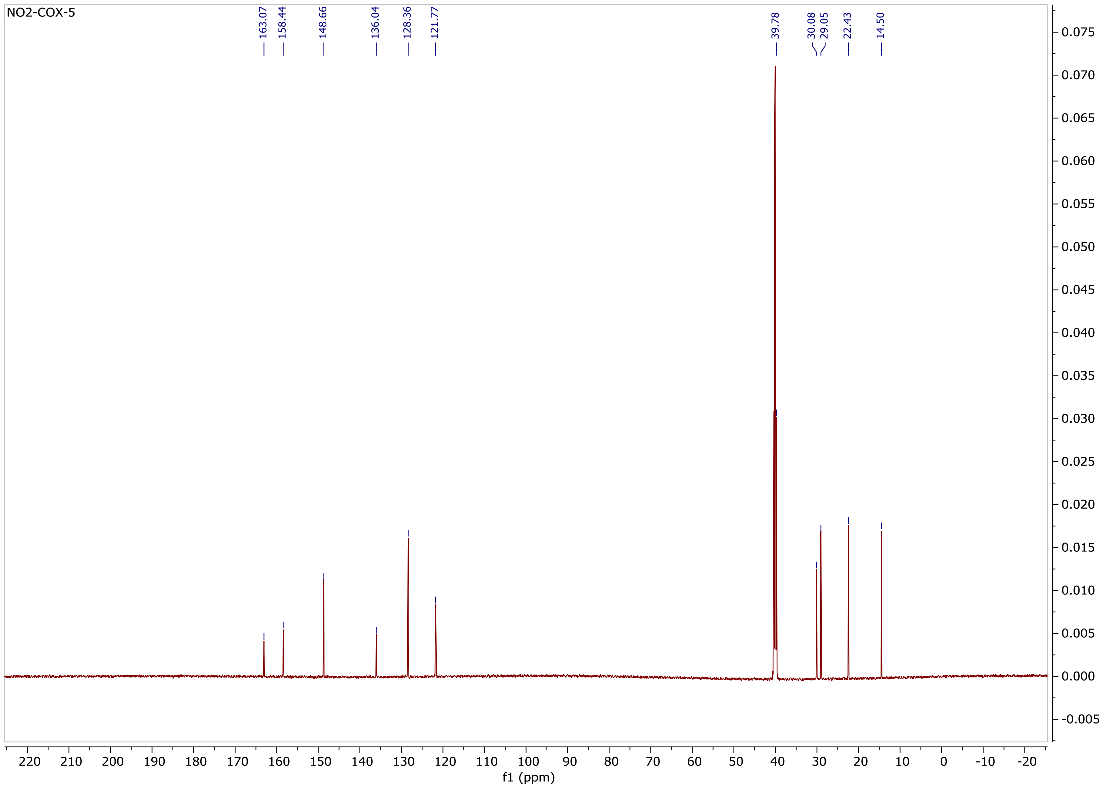

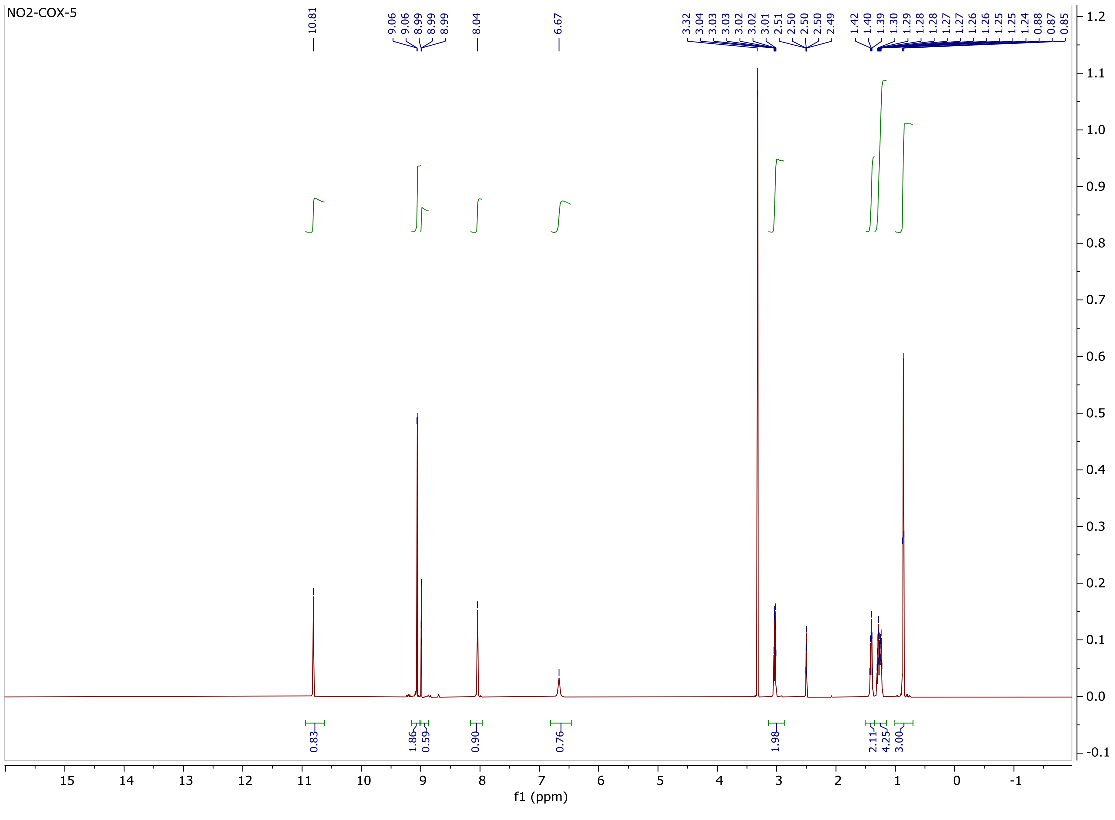

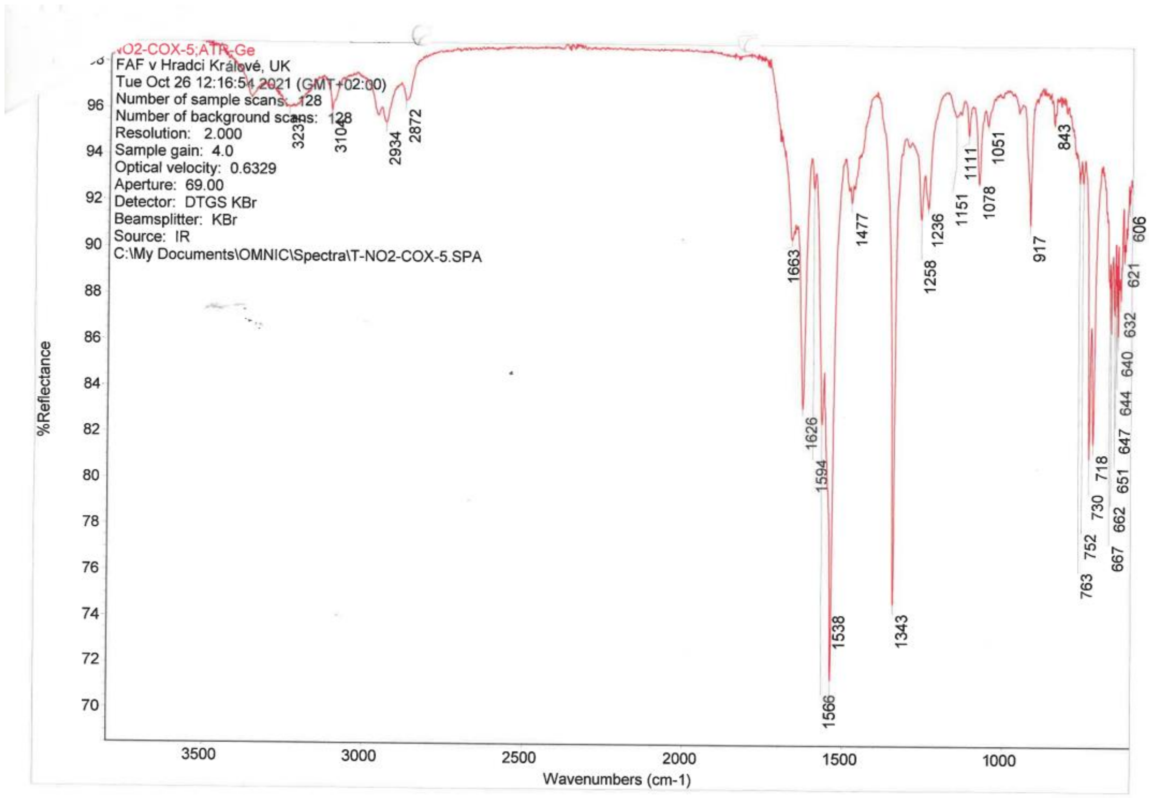

2-(3,5-Dinitrobenzoyl)-*N*-hexylhydrazine-1-carboxamide **4f**

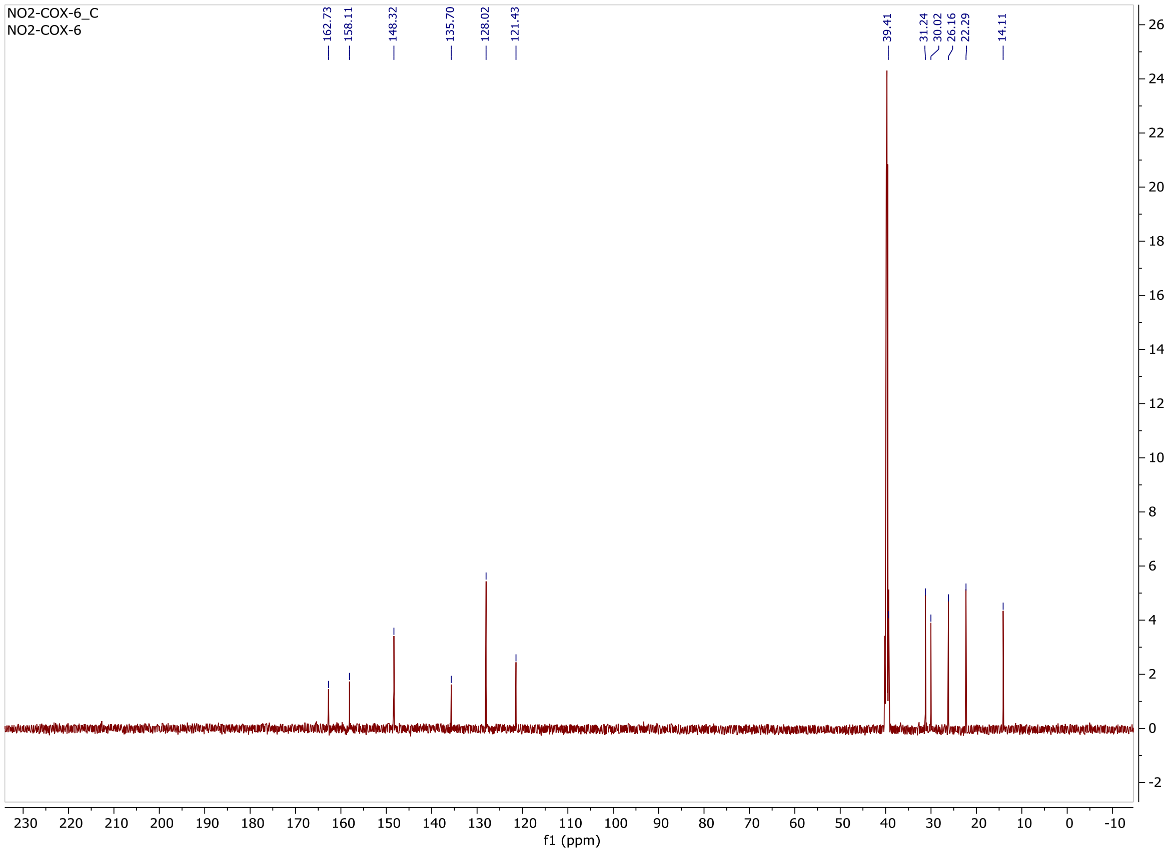

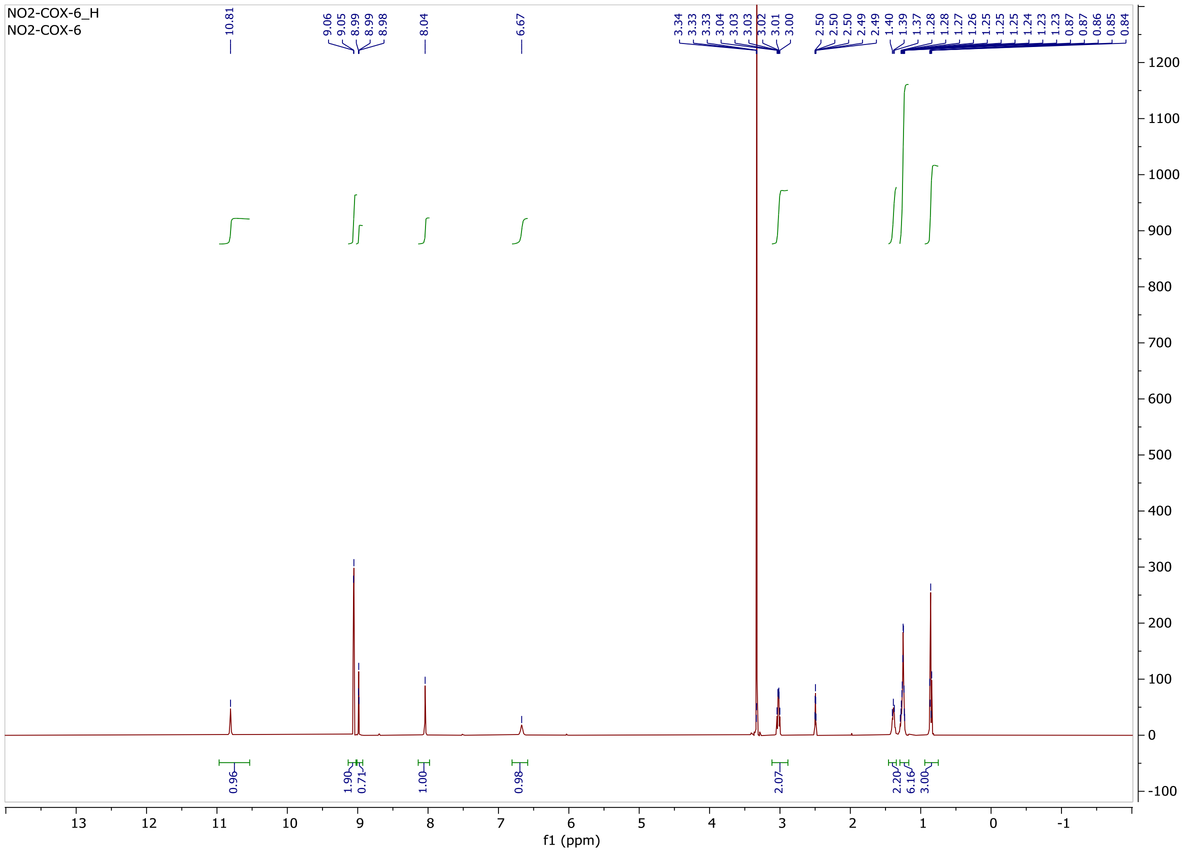
NO2-COX-6_H
NO2-COX-6
f1 (ppm)

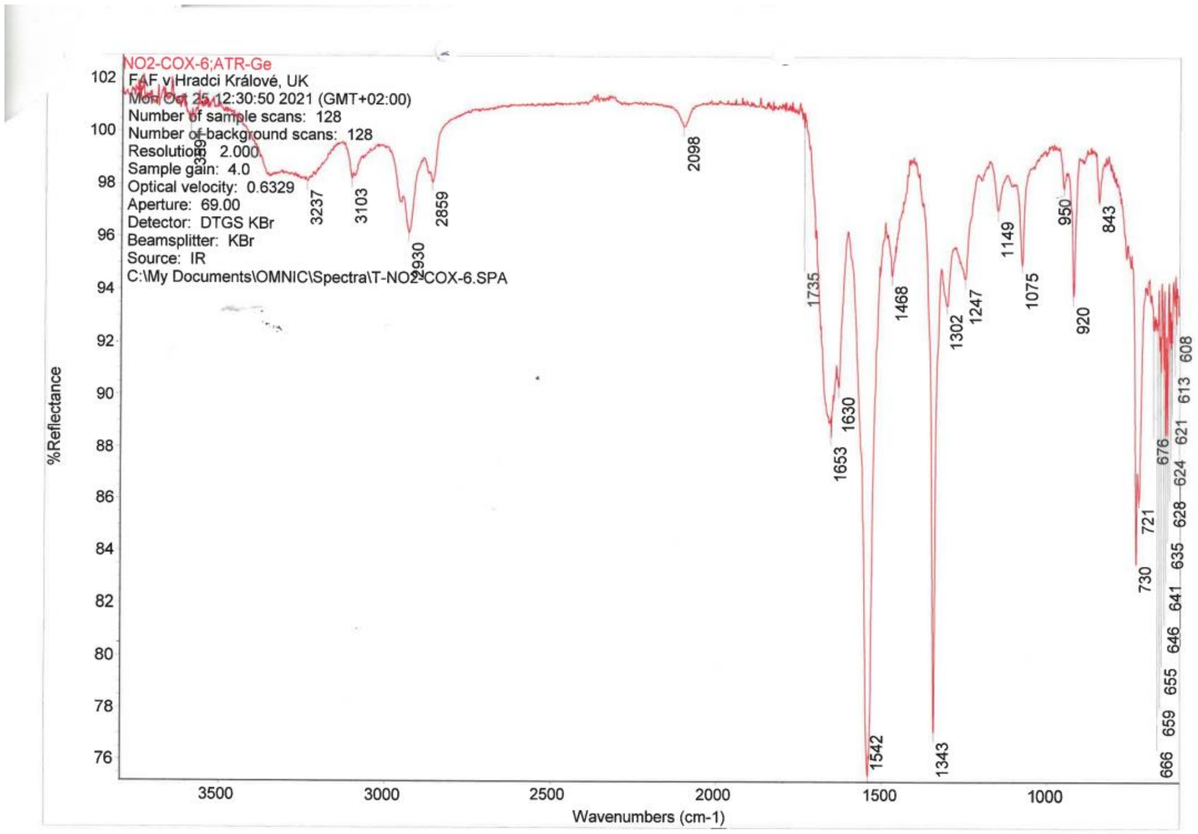
NO2-COX-6;ATR-Ge
FAF v Hradci Králové, UK
Mon Oct 25 12:30:50 2021 (GMT+02:00)
Number of sample scans: 128
Number of background scans: 128
Resolution: 2.000
Sample gain: 4.0
Optical velocity: 0.6329
Aperture: 69.00
Detector: DTGS KBr
Beamsplitter: KBr
Source: IR
C:\My Documents\OMNIC\Spectra\T-NO2-COX-6.SPA
%Reflectance
Wavenumbers (cm-1)

2-(3,5-Dinitrobenzoyl)-*N*-heptylhydrazine-1-carboxamide **4g**

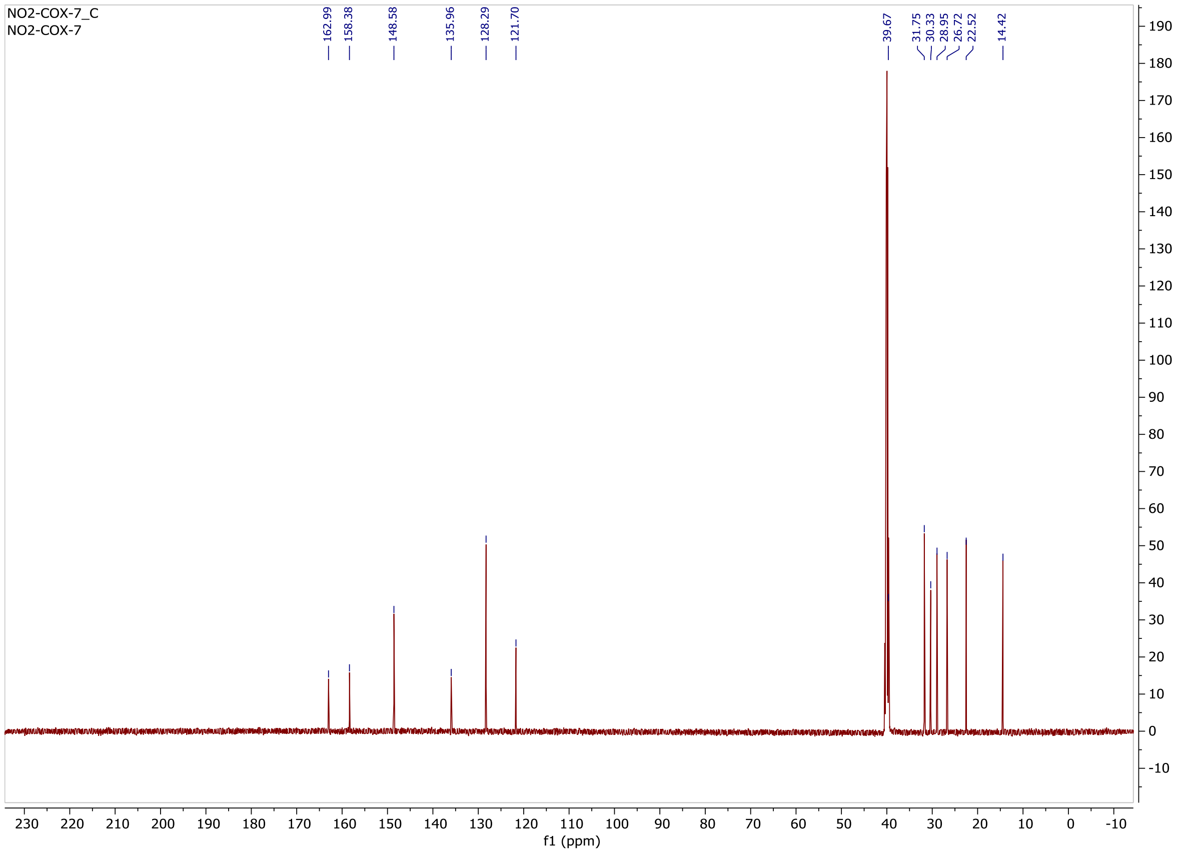

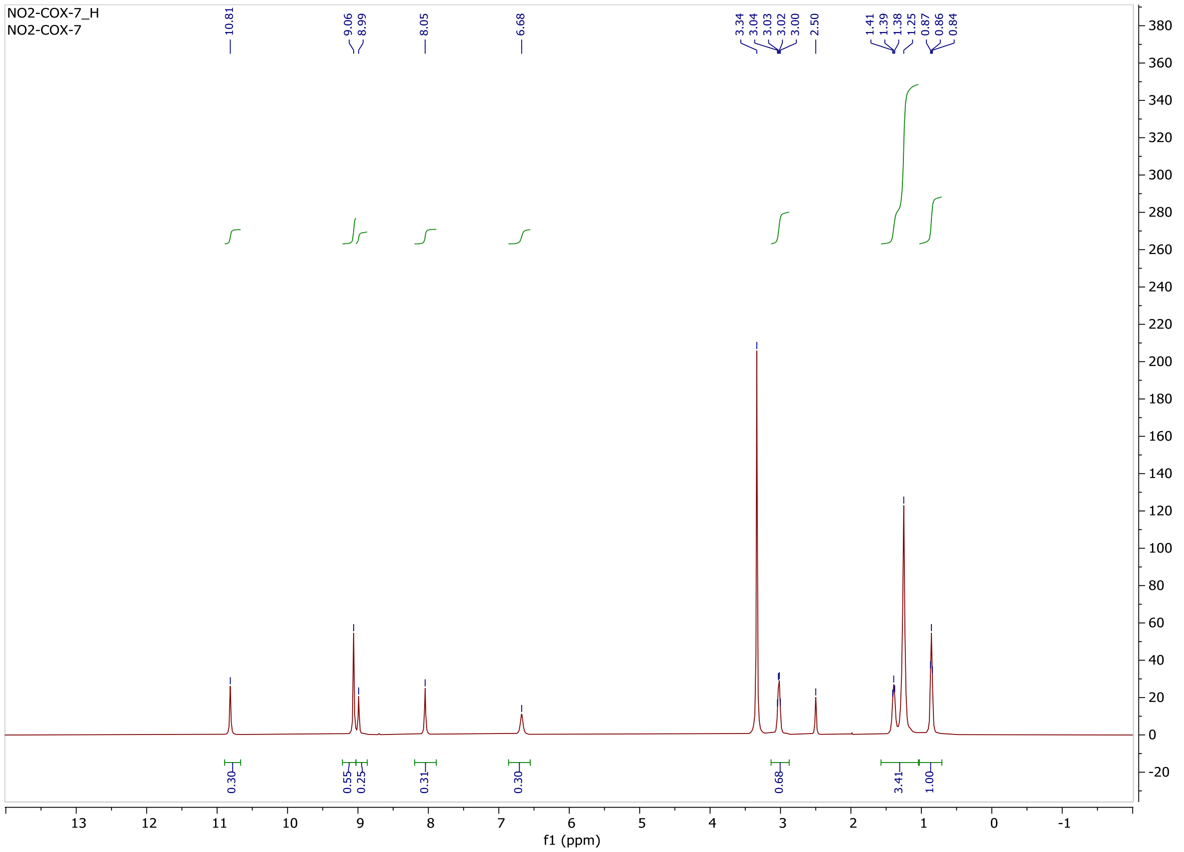

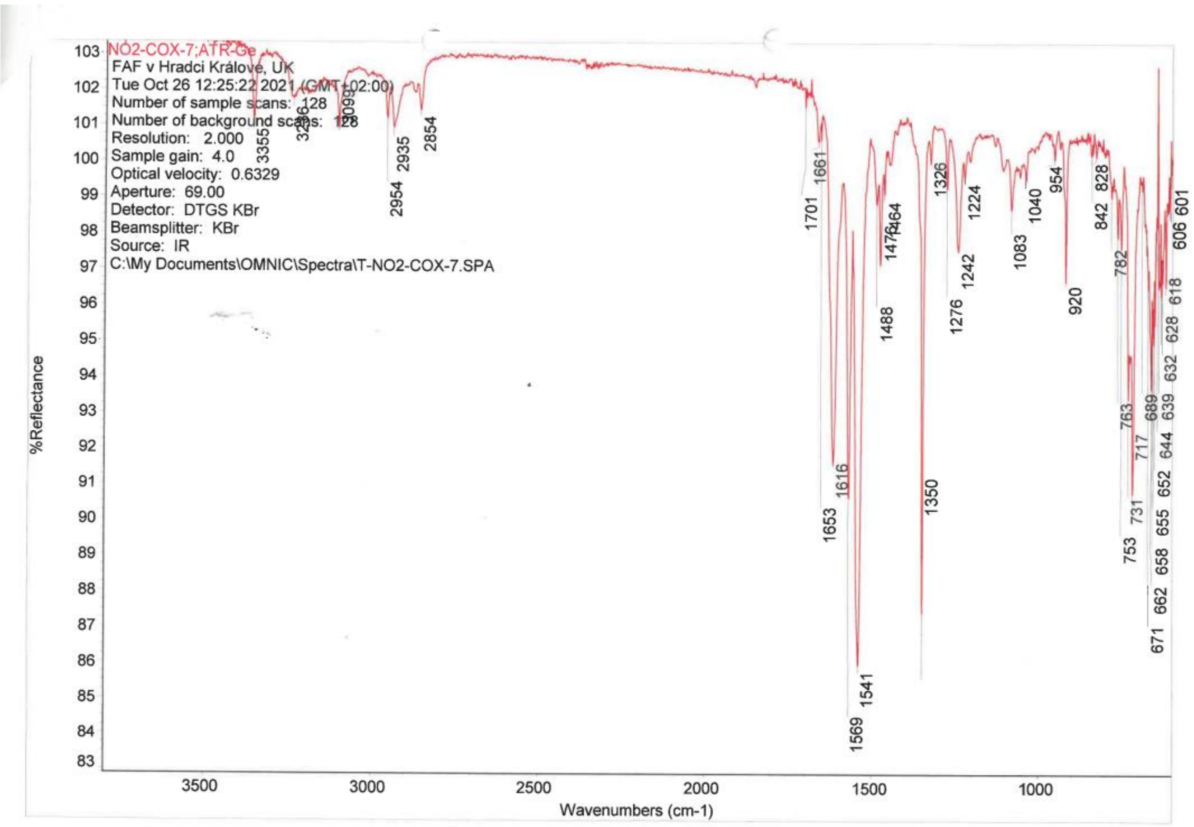

2-(3,5-Dinitrobenzoyl)-*N*-octylhydrazine-1-carboxamide **4h**

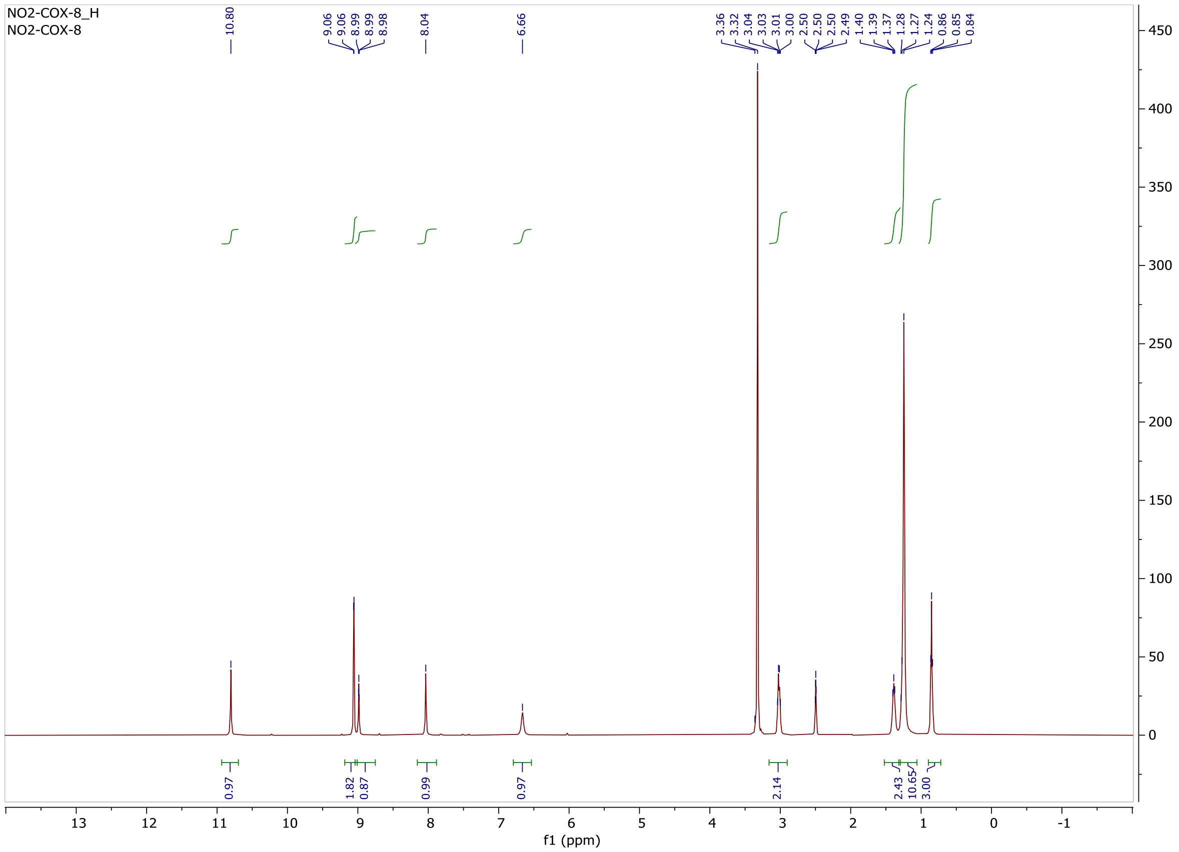

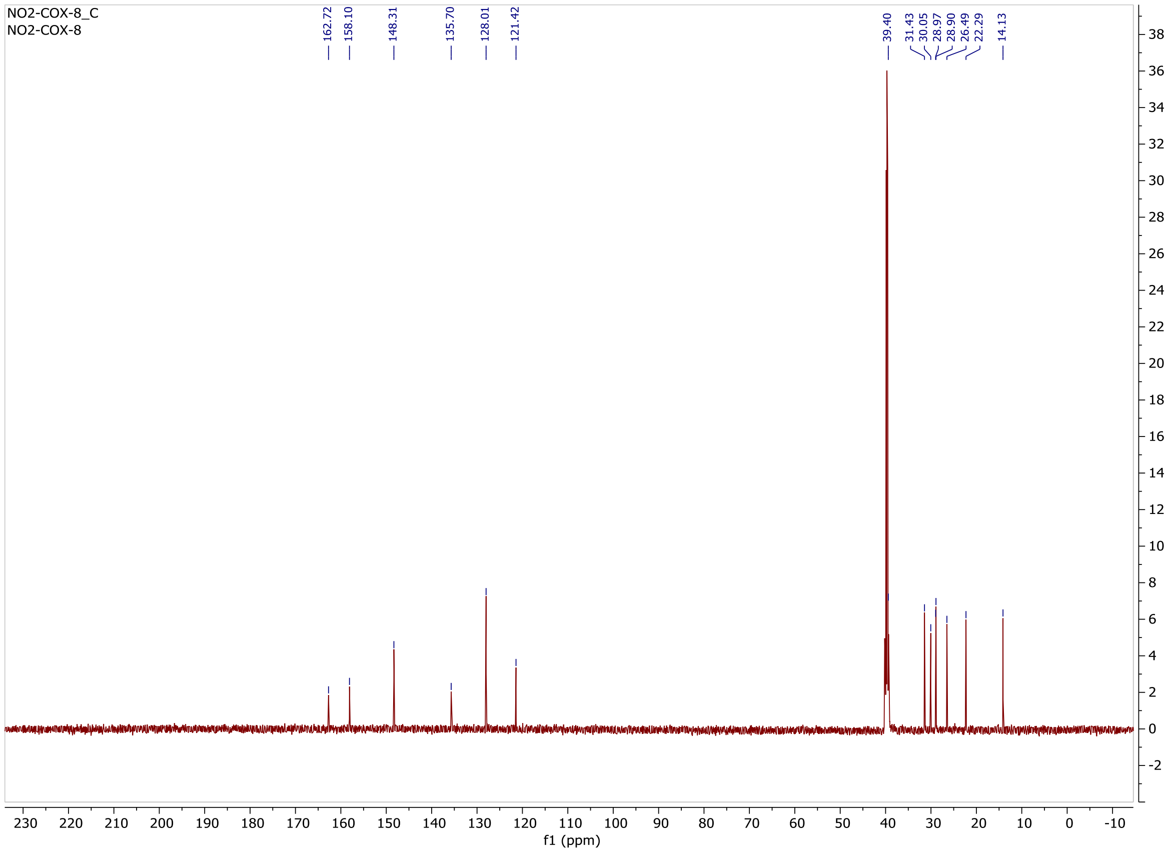

NO2-COX-8_C
NO2-COX-8
162.72
158.10
148.31
135.70
128.01
121.42
39.40
31.43
30.05
28.97
28.90
26.49
22.29
14.13
f1 (ppm)

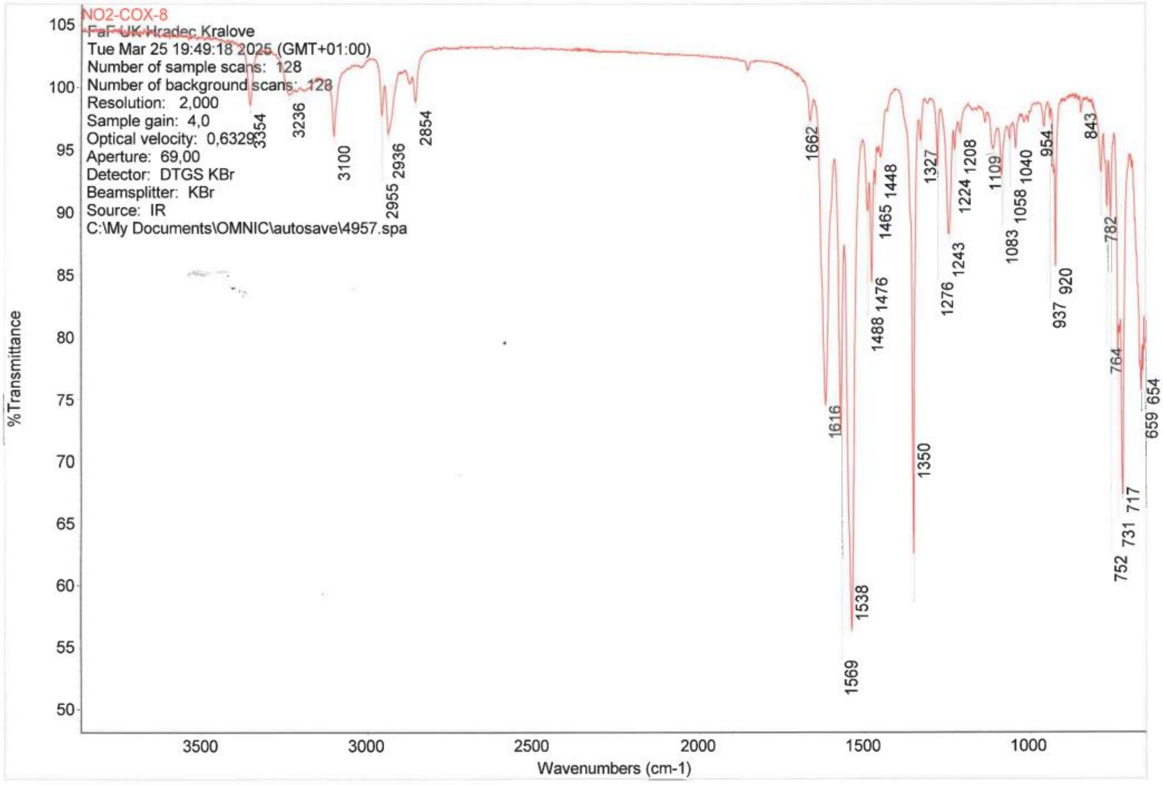

NO2-COX-8
FaF UK Hradec Kralove
Tue Mar 25 19:49:18 2025 (GMT+01:00)
Number of sample scans: 128
Number of background scans: 128
Resolution: 2,000
Sample gain: 4,0
Optical velocity: 0,6329
Aperture: 69,00
Detector: DTGS KBr
Beamsplitter: KBr
Source: IR
C:\My Documents\OMNIC\autosave\4957.spa
%Transmittance
Wavenumbers (cm-1)
3354
3236
3100
2955
2936
2854
1662
1616
1569
1538
1488
1476
1465
1448
1350
1327
1276
1243
1224
1208
1109
1083
1058
1040
954
937
920
843
782
764
752
731
717
659
654

2-(3,5-Dinitrobenzoyl)-*N*-nonylhydrazine-1-carboxamide **4i**

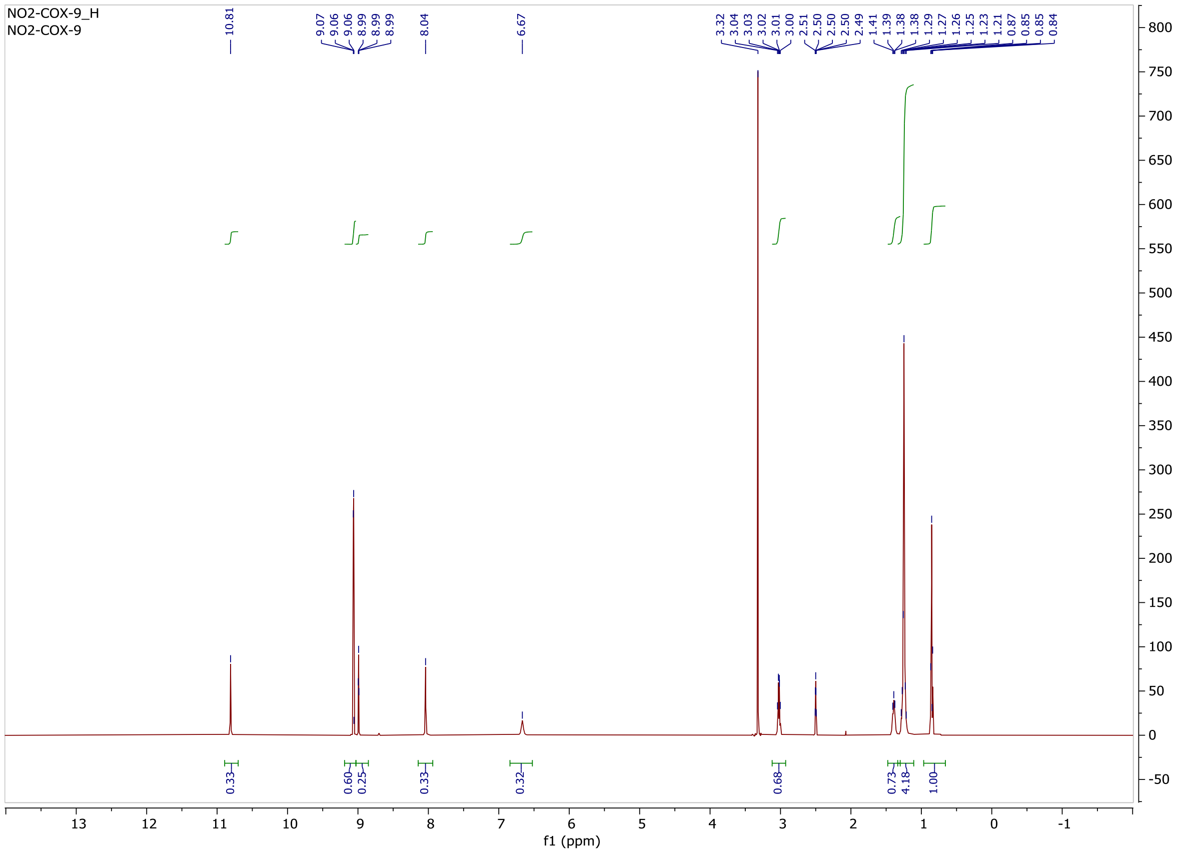

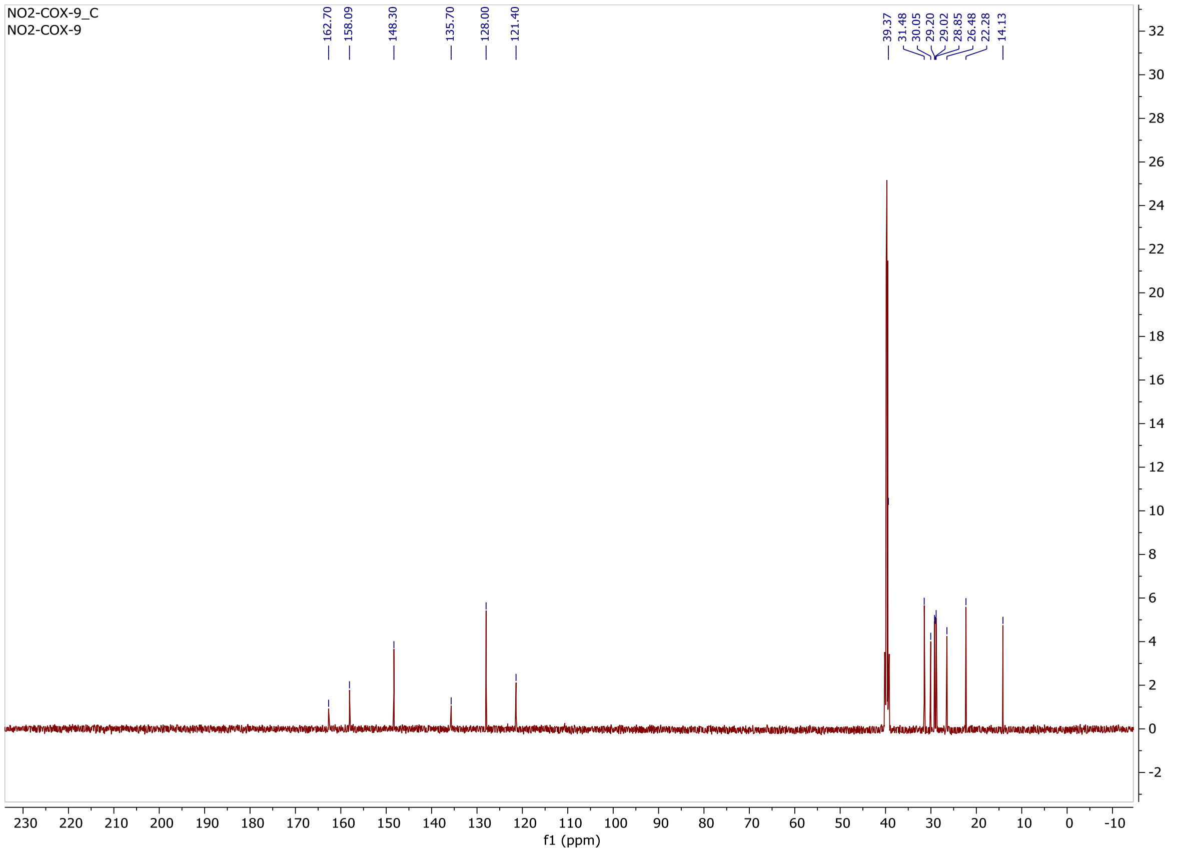

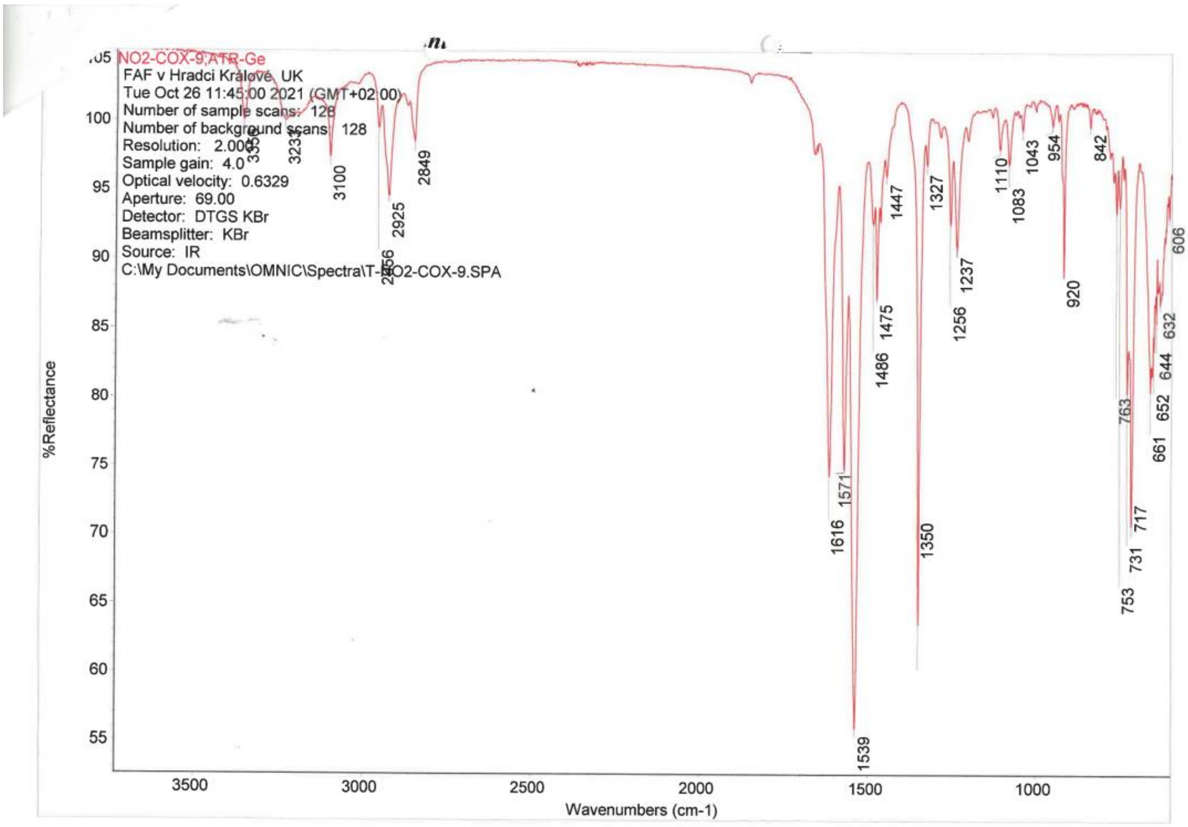

N-Decyl-2-(3,5-dinitrobenzoyl)hydrazine-1-carboxamide **4j**

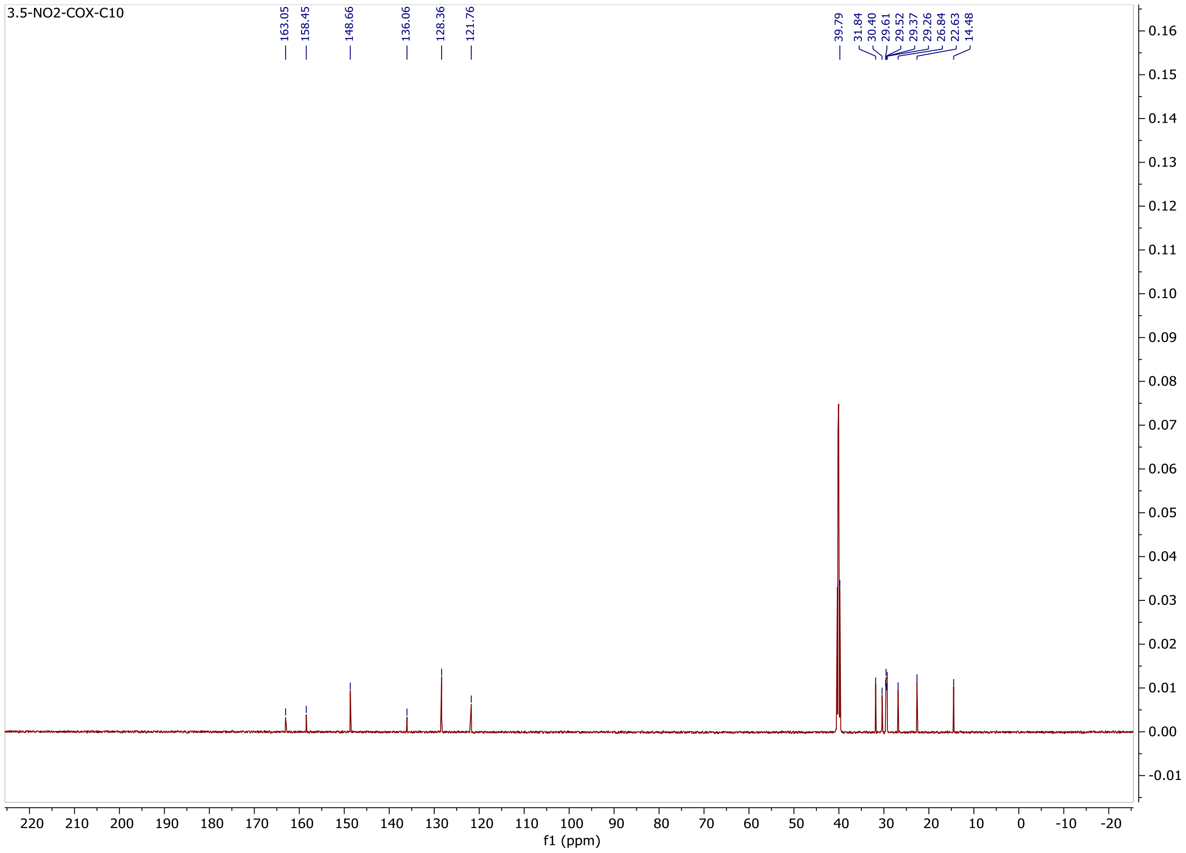

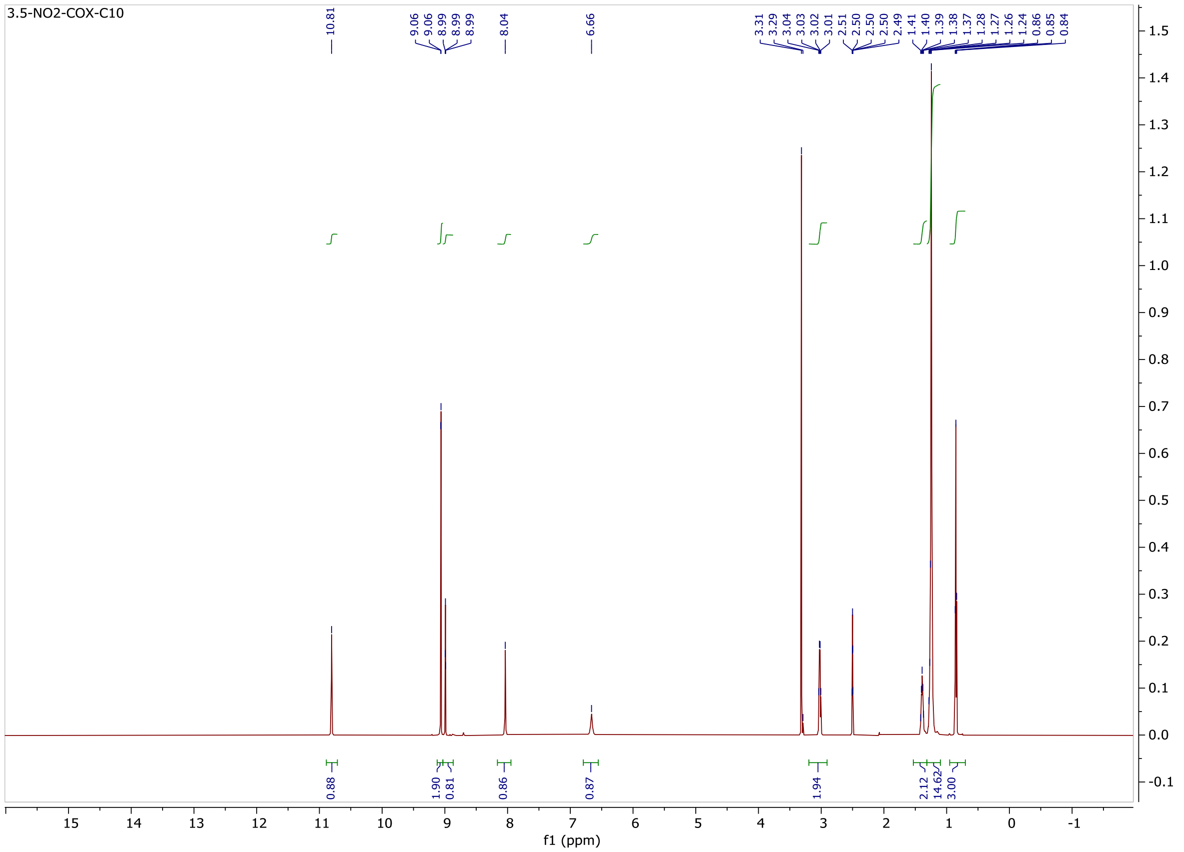
3.5-NO2-COX-C10
f1 (ppm)

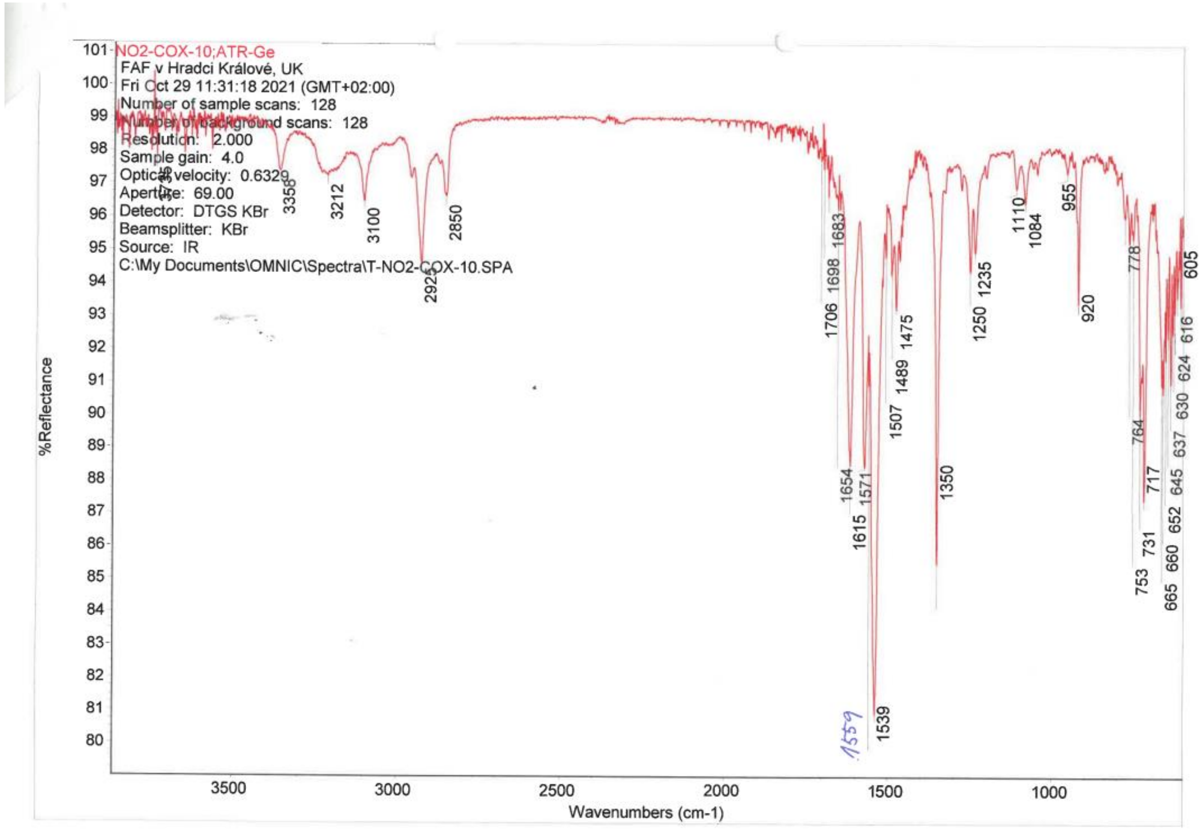
NO2-COX-10;ATR-Ge
FAF v Hradci Králové, UK
Fri Oct 29 11:31:18 2021 (GMT+02:00)
Number of sample scans: 128
Number of background scans: 128
Resolution: 2.000
Sample gain: 4.0
Optical velocity: 0.6329
Aperture: 69.00
Detector: DTGS KBr
Beamsplitter: KBr
Source: IR
C:\My Documents\OMNIC\Spectra\T-NO2-COX-10.SPA
%Reflectance
Wavenumbers (cm-1)
1559

2-(3,5-Dinitrobenzoyl)-*N*-undecylhydrazine-1-carboxamide **4k**

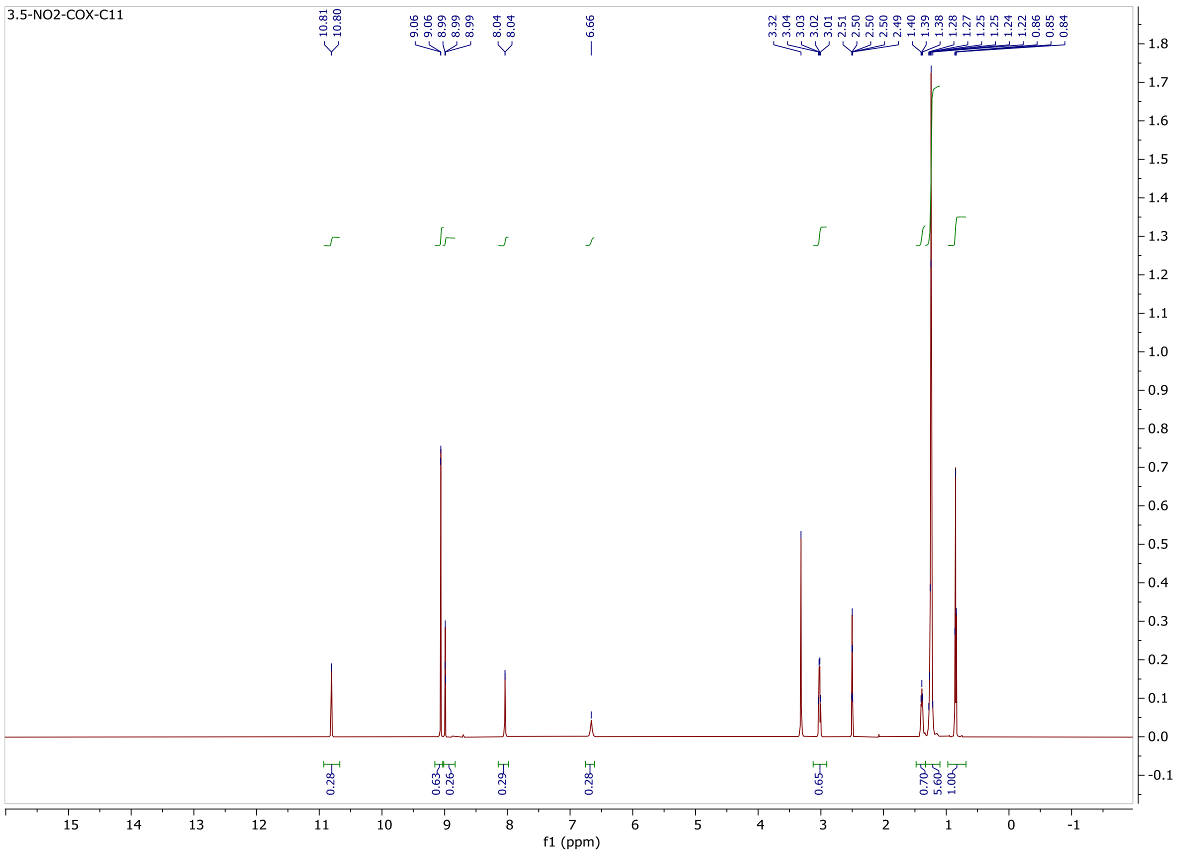

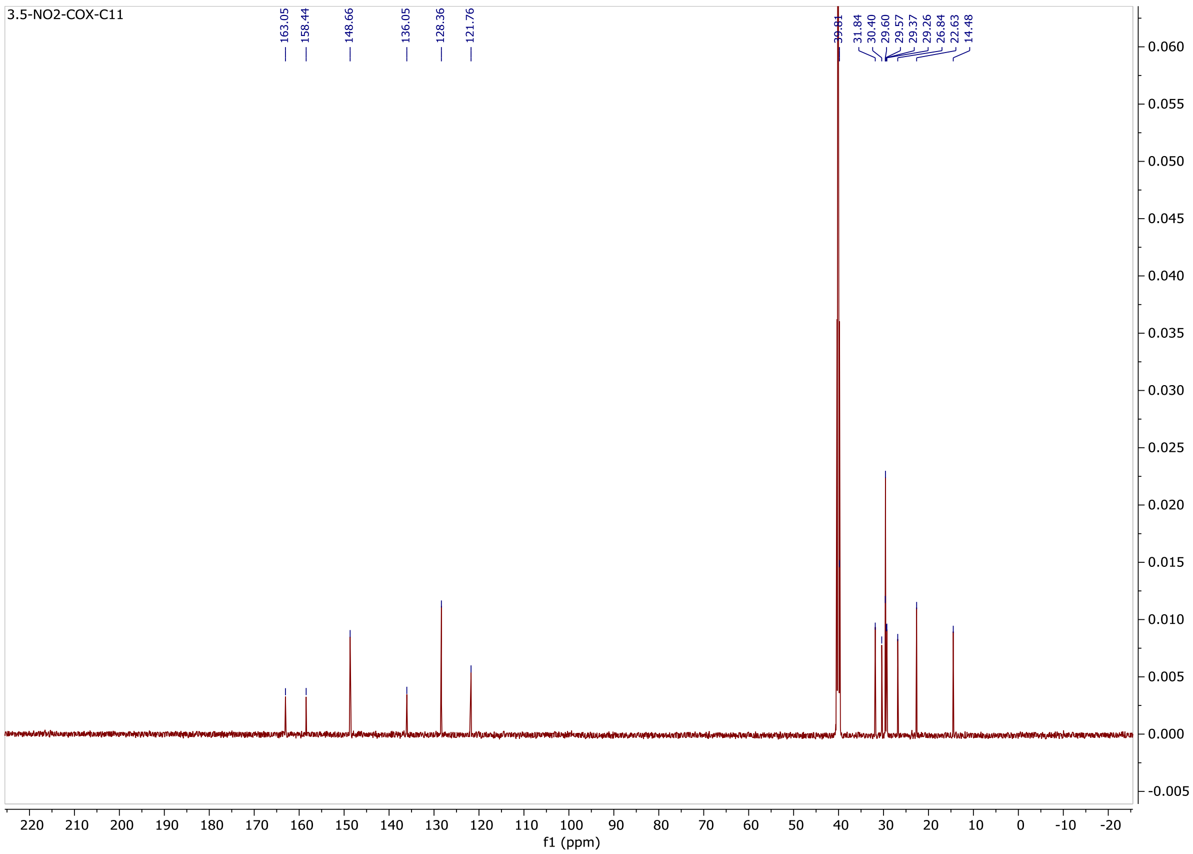

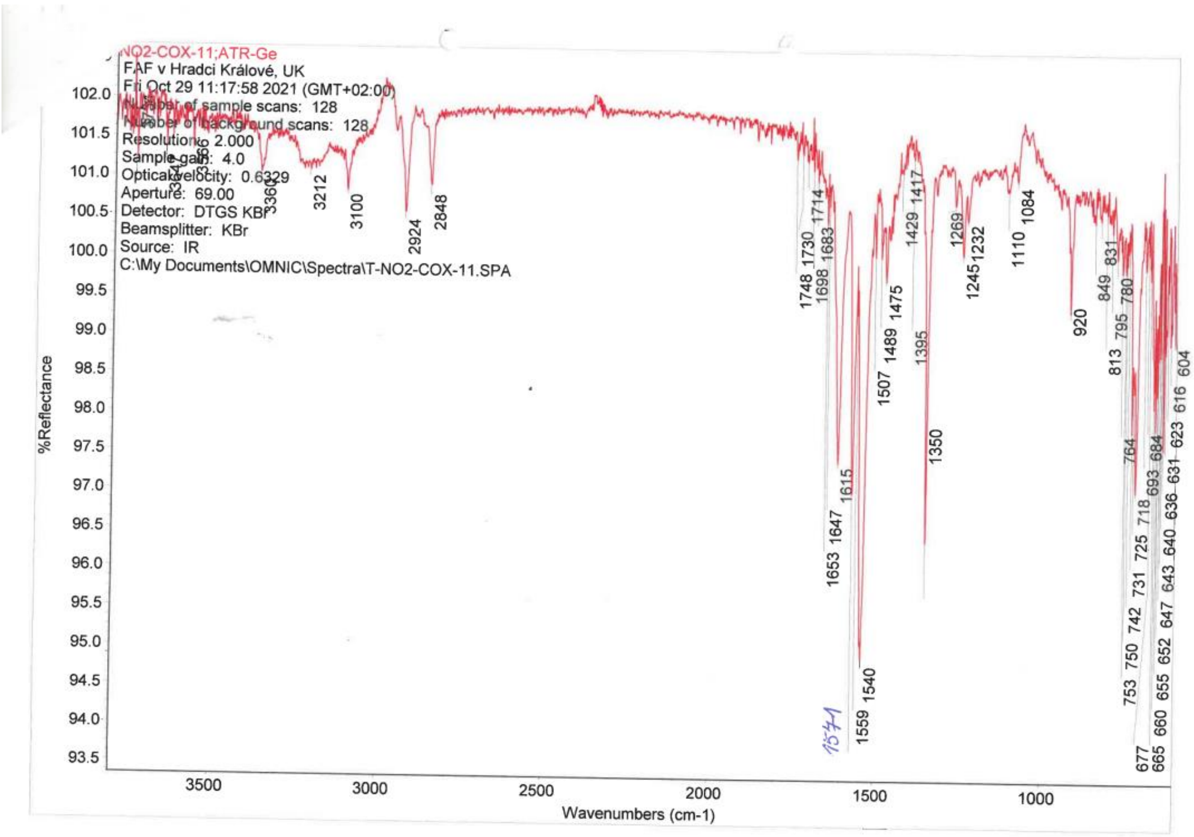

2-(3,5-Dinitrobenzoyl)-*N*-dodecylhydrazine-1-carboxamide **4l**

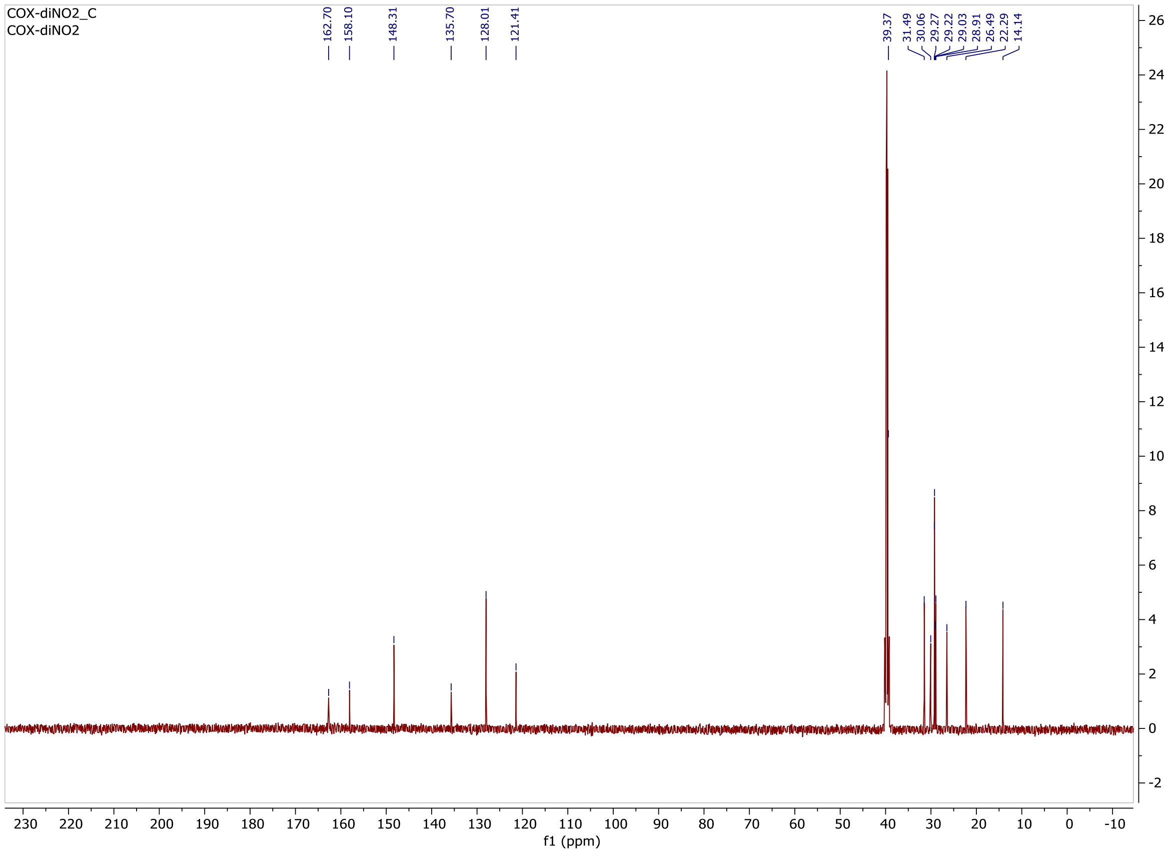

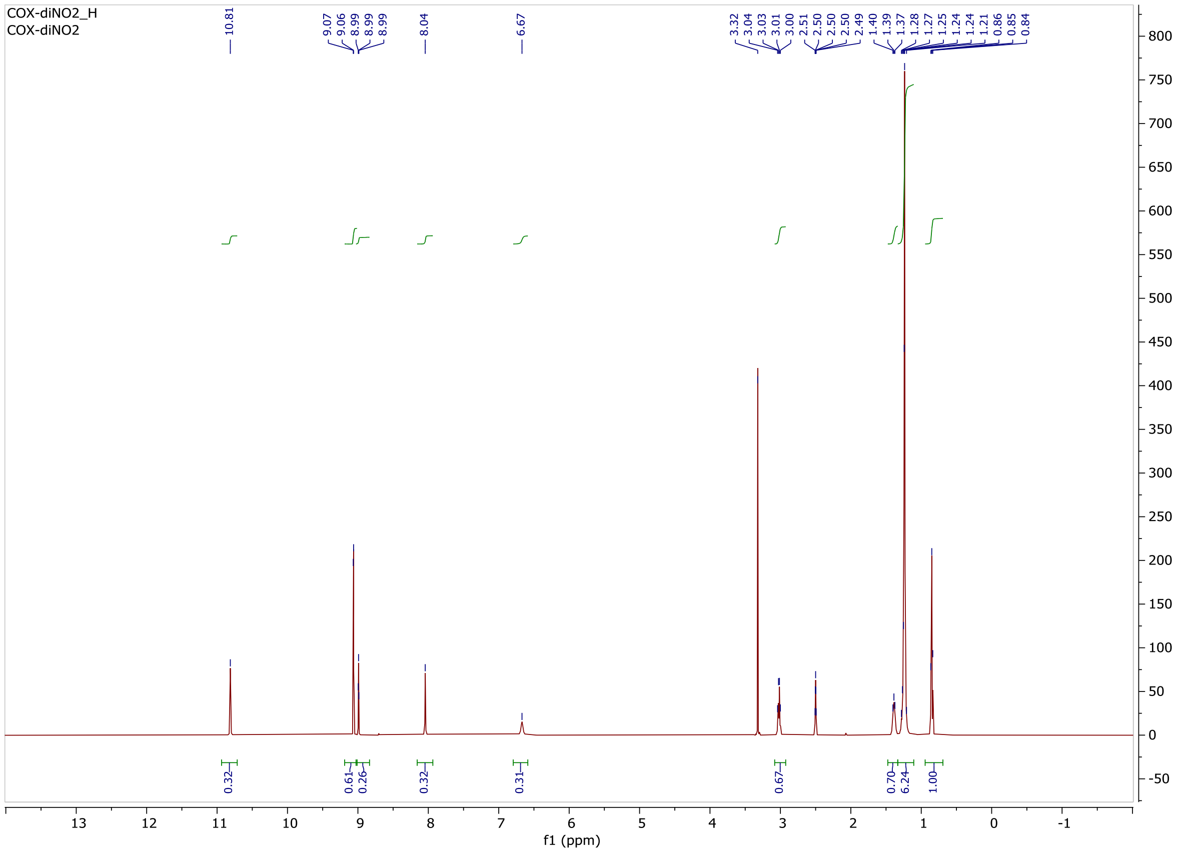
COX-diNO2_H
COX-diNO2
f1 (ppm)

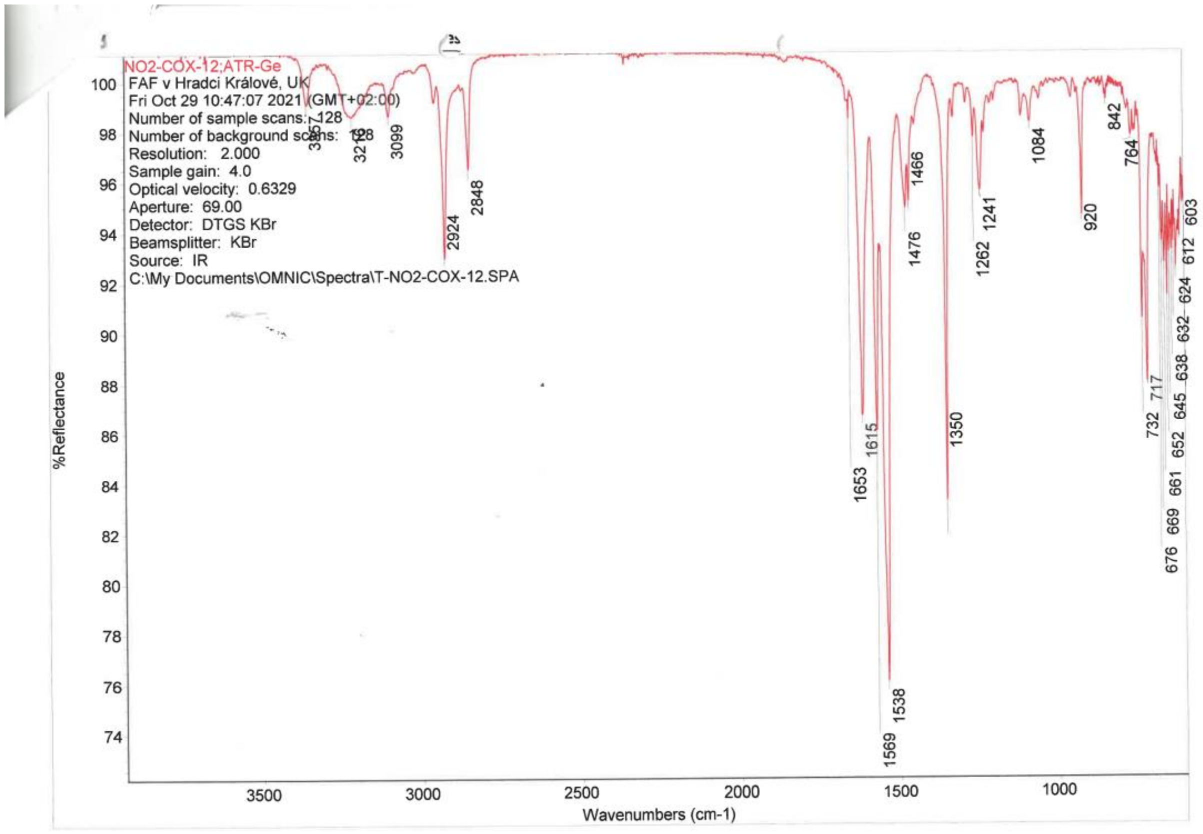
NO2-COX-12;ATR-Ge
FAF v Hradci Králové, UK
Fri Oct 29 10:47:07 2021 (GMT+02:00)
Number of sample scans: 128
Number of background scans: 128
Resolution: 2.000
Sample gain: 4.0
Optical velocity: 0.6329
Aperture: 69.00
Detector: DTGS KBr
Beamsplitter: KBr
Source: IR
C:\My Documents\OMNIC\Spectra\T-NO2-COX-12.SPA
%Reflectance
Wavenumbers (cm-1)

2-(3,5-Dinitrobenzoyl)-*N*-tridecylhydrazine-1-carboxamide **4m**

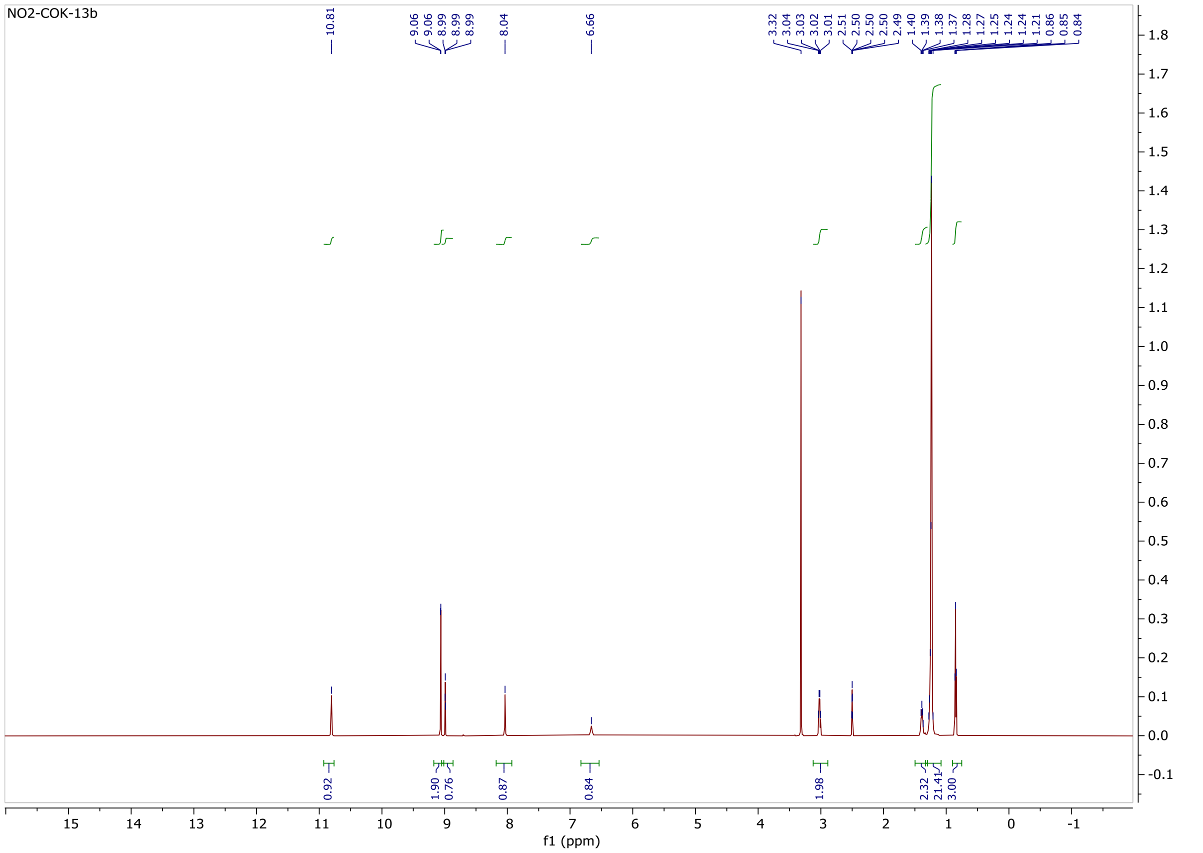

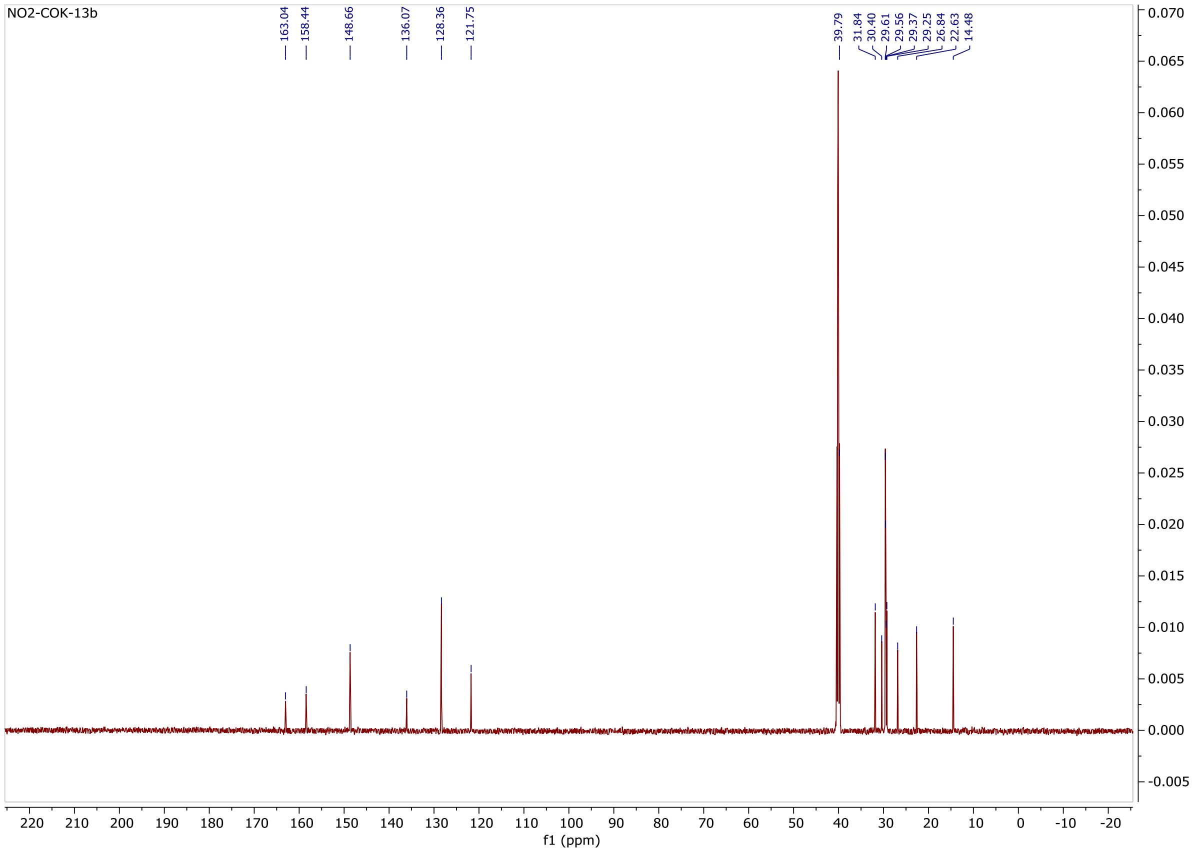

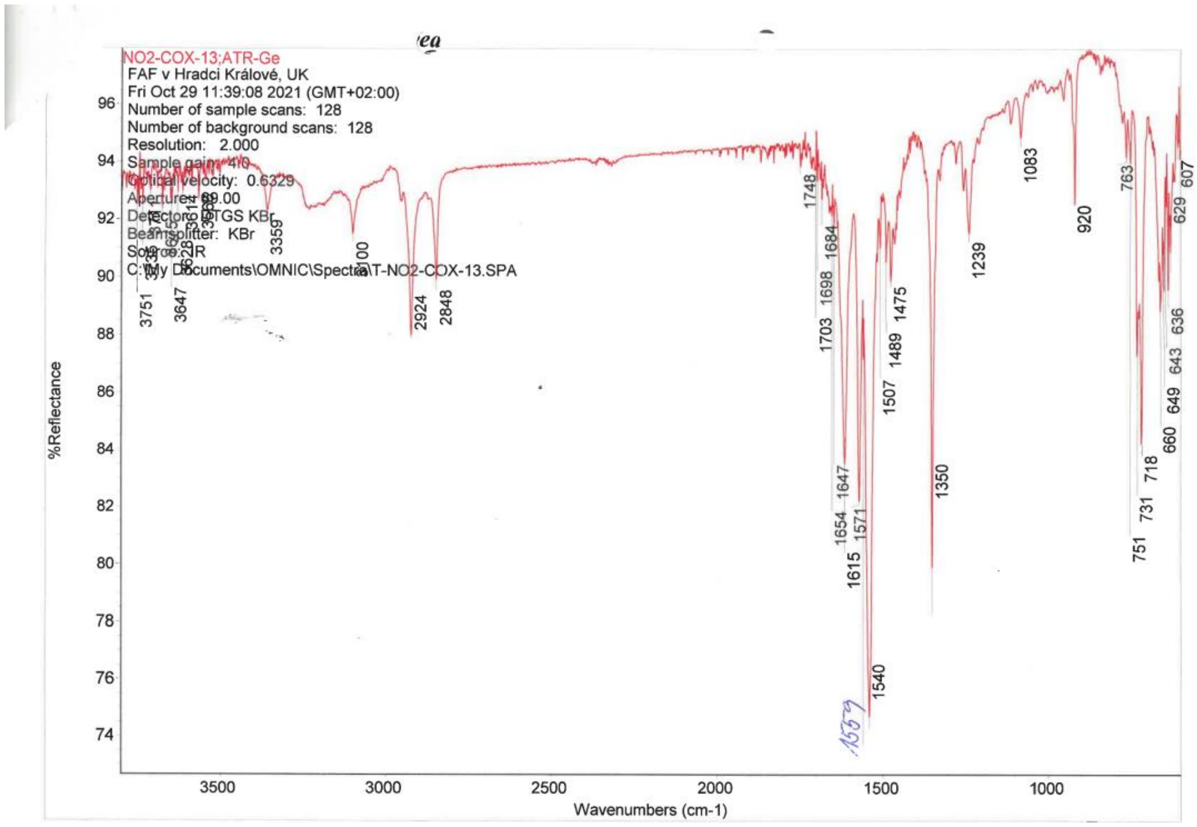

2-(3,5-Dinitrobenzoyl)-*N*-tetradecylhydrazine-1-carboxamide **4n**

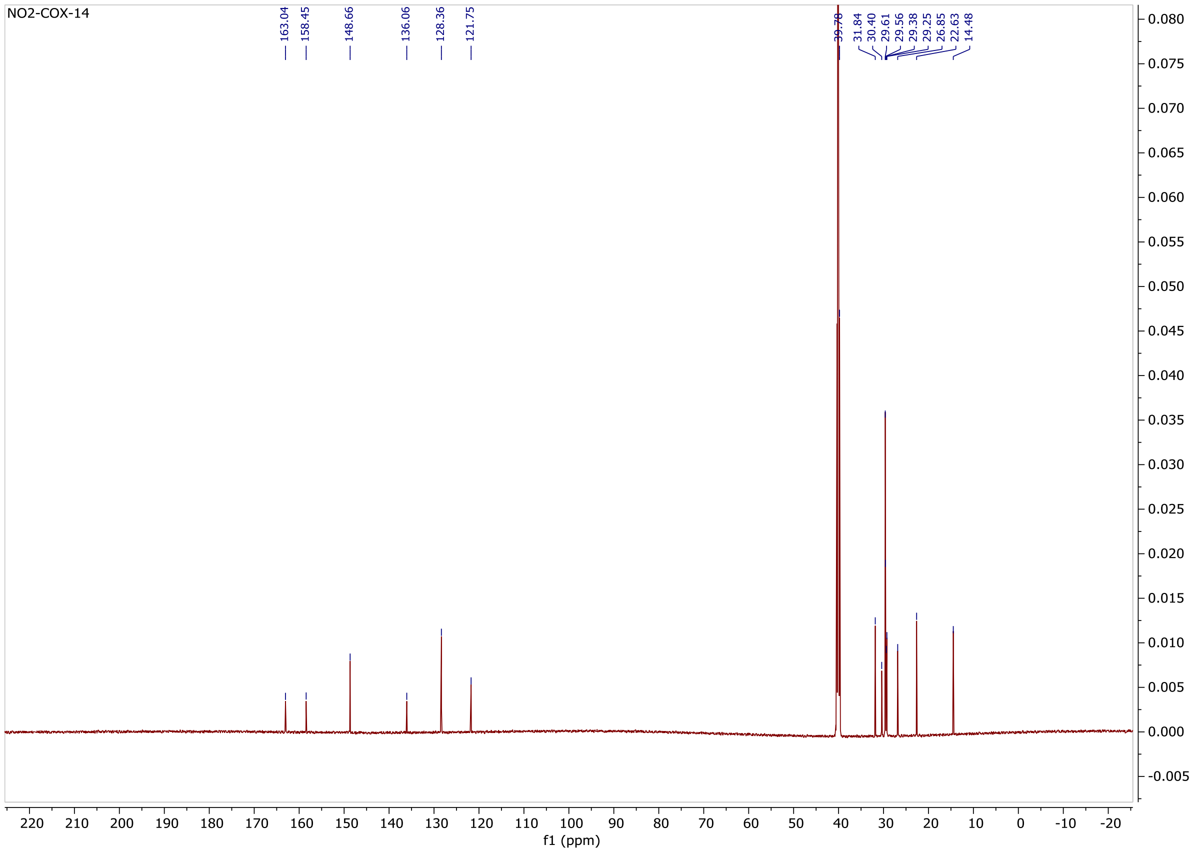

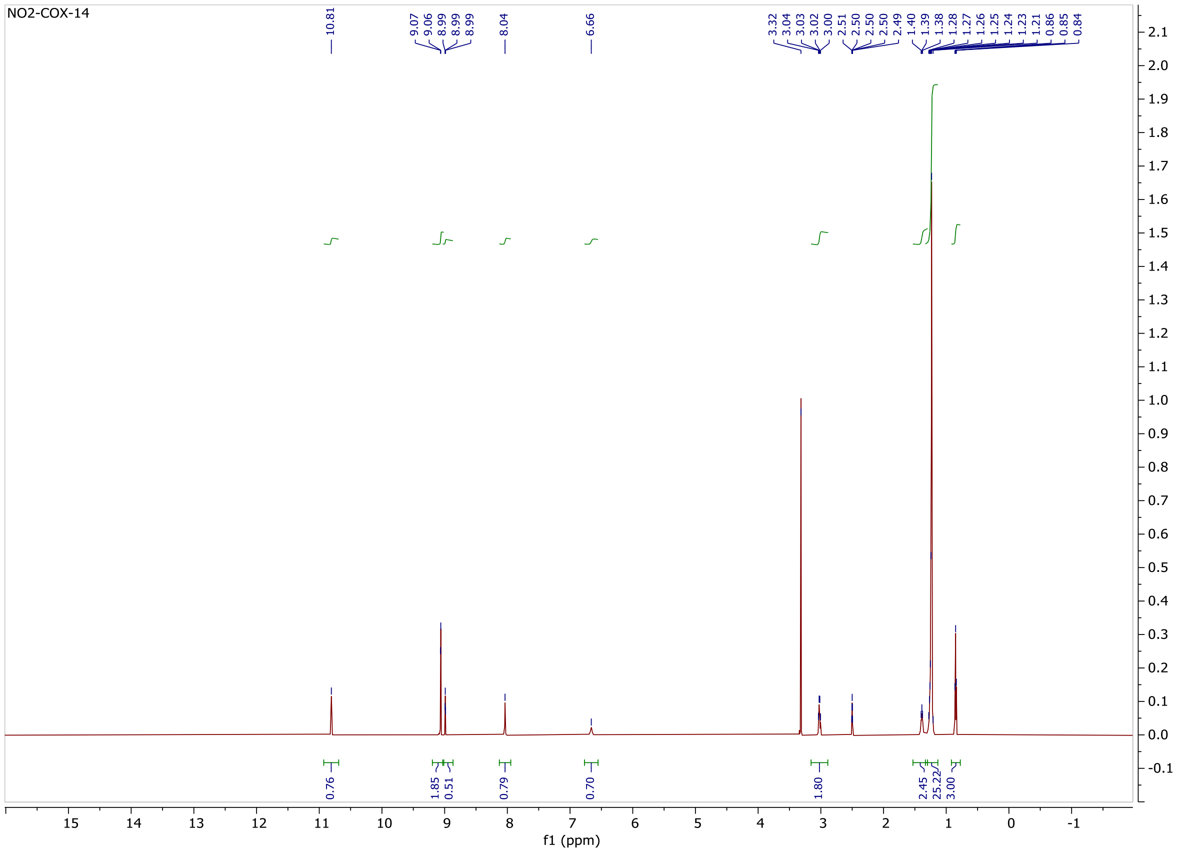
NO2-COX-14
10.81
9.07
9.06
8.99
8.99
8.99
8.04
6.66
3.32
3.04
3.03
3.02
3.00
2.51
2.50
2.50
2.50
2.49
1.40
1.39
1.38
1.28
1.27
1.26
1.25
1.24
1.23
1.21
0.86
0.85
0.84
0.76
1.85
0.51
0.79
0.70
1.80
2.45
25.22
3.00
f1 (ppm)

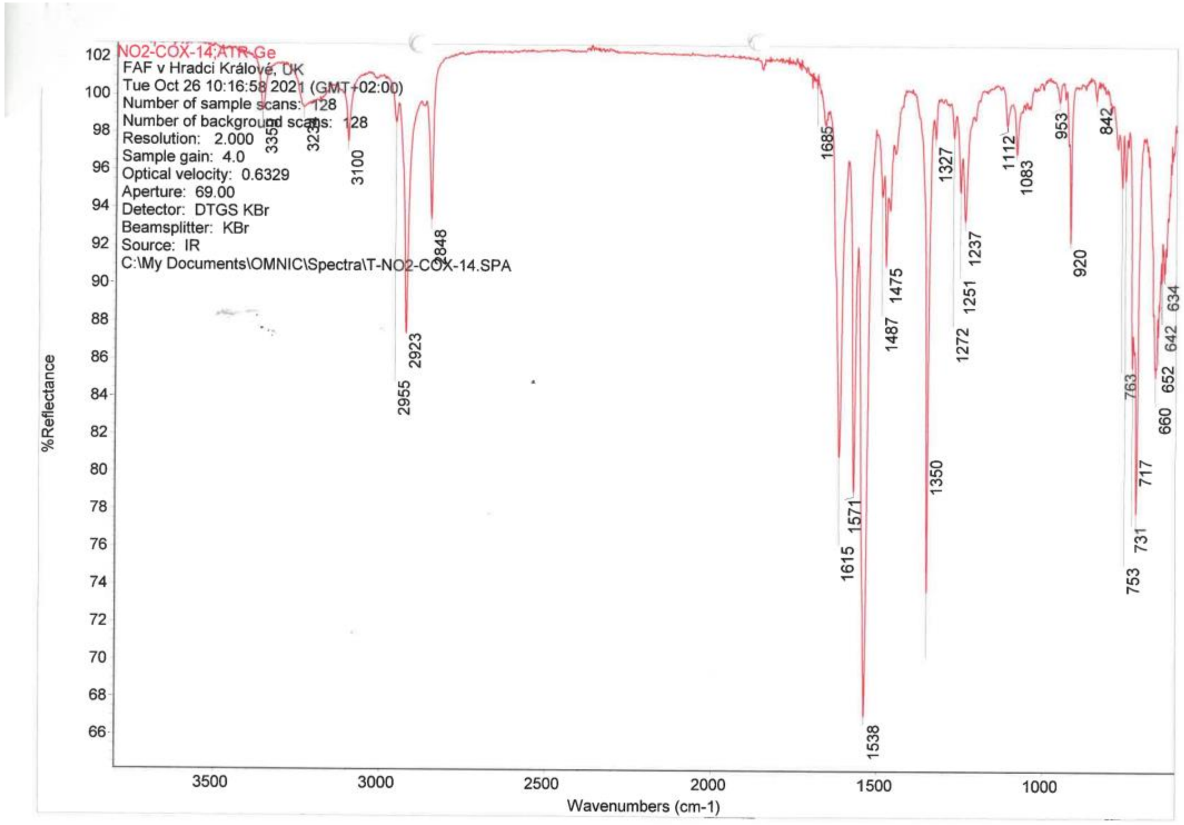
NO2-COX-14 ATR Ge
FAF v Hradci Králové, UK
Tue Oct 26 10:16:58 2021 (GMT+02:00)
Number of sample scans: 128
Number of background scans: 128
Resolution: 2.000
Sample gain: 4.0
Optical velocity: 0.6329
Aperture: 69.00
Detector: DTGS KBr
Beamsplitter: KBr
Source: IR
C:\My Documents\OMNIC\Spectra\T-NO2-COX-14.SPA
%Reflectance
Wavenumbers (cm-1)
3100
2955
2923
2848
1685
1615
1571
1538
1487
1475
1350
1327
1272
1251
1237
1112
1083
953
920
842
763
753
731
717
660
652
642
634

2-(3,5-Dinitrobenzoyl)-*N*-pentadecylhydrazine-1-carboxamide **4o**

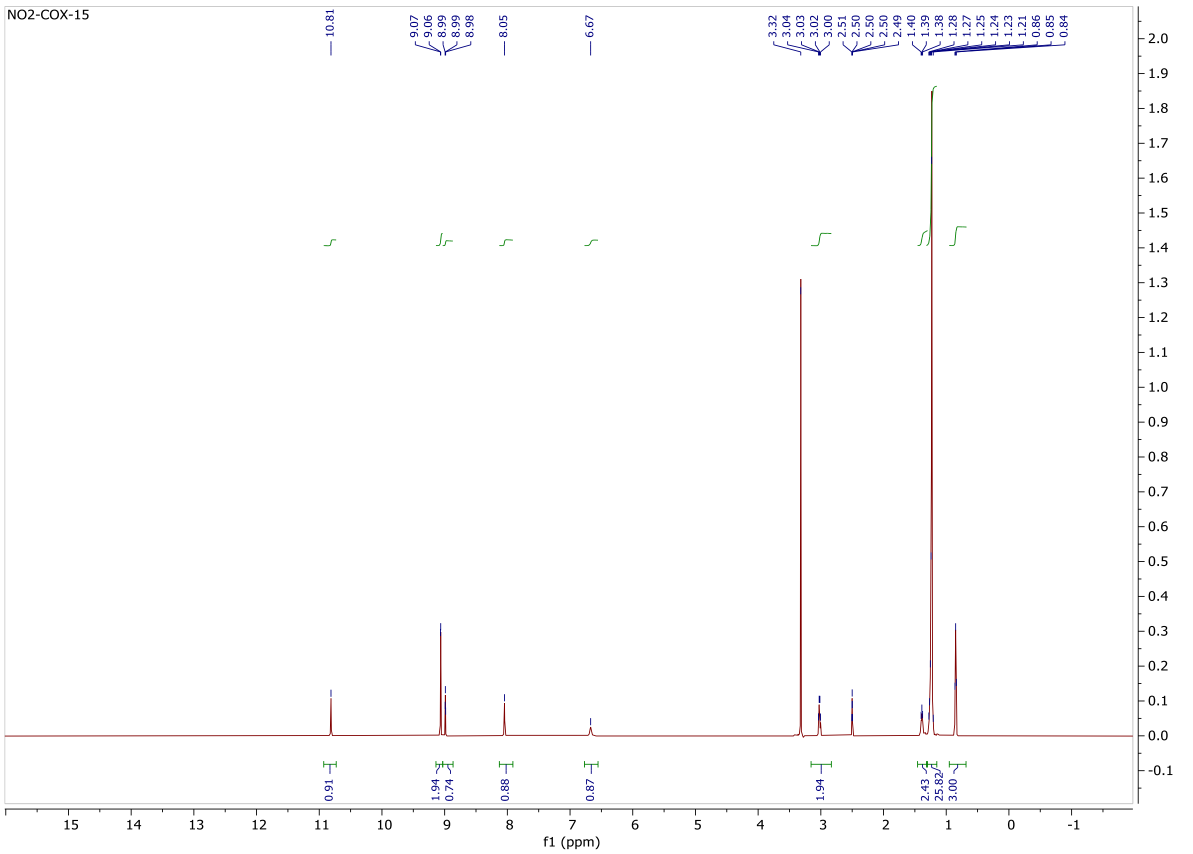

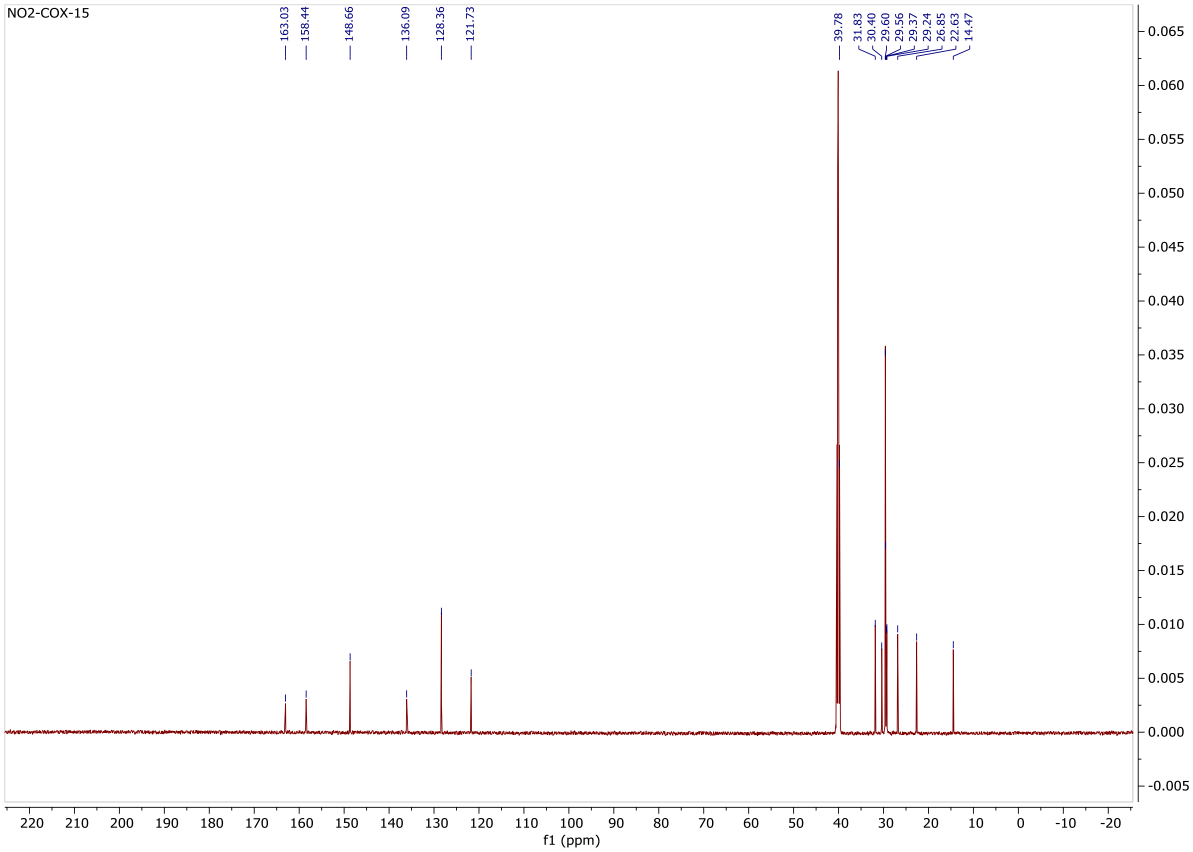

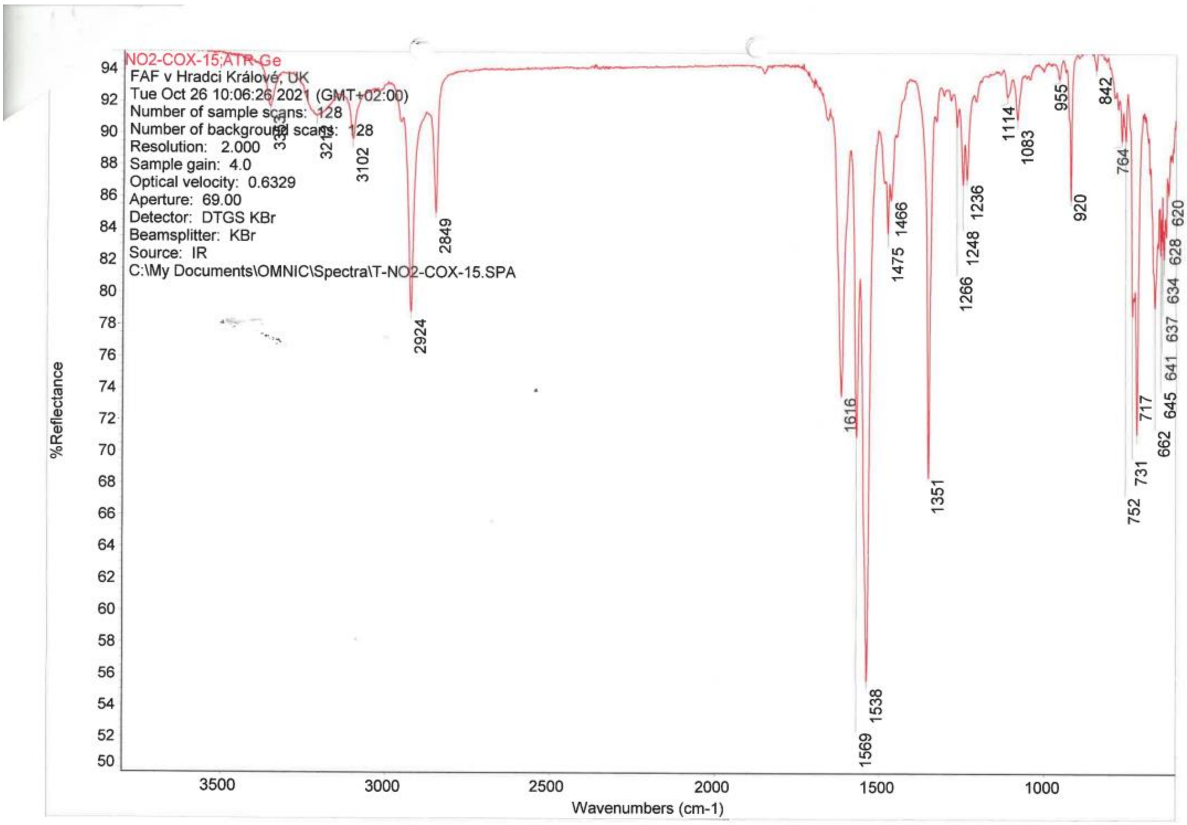

2-(3,5-Dinitrobenzoyl)-*N*-hexadecylhydrazine-1-carboxamide **4p**

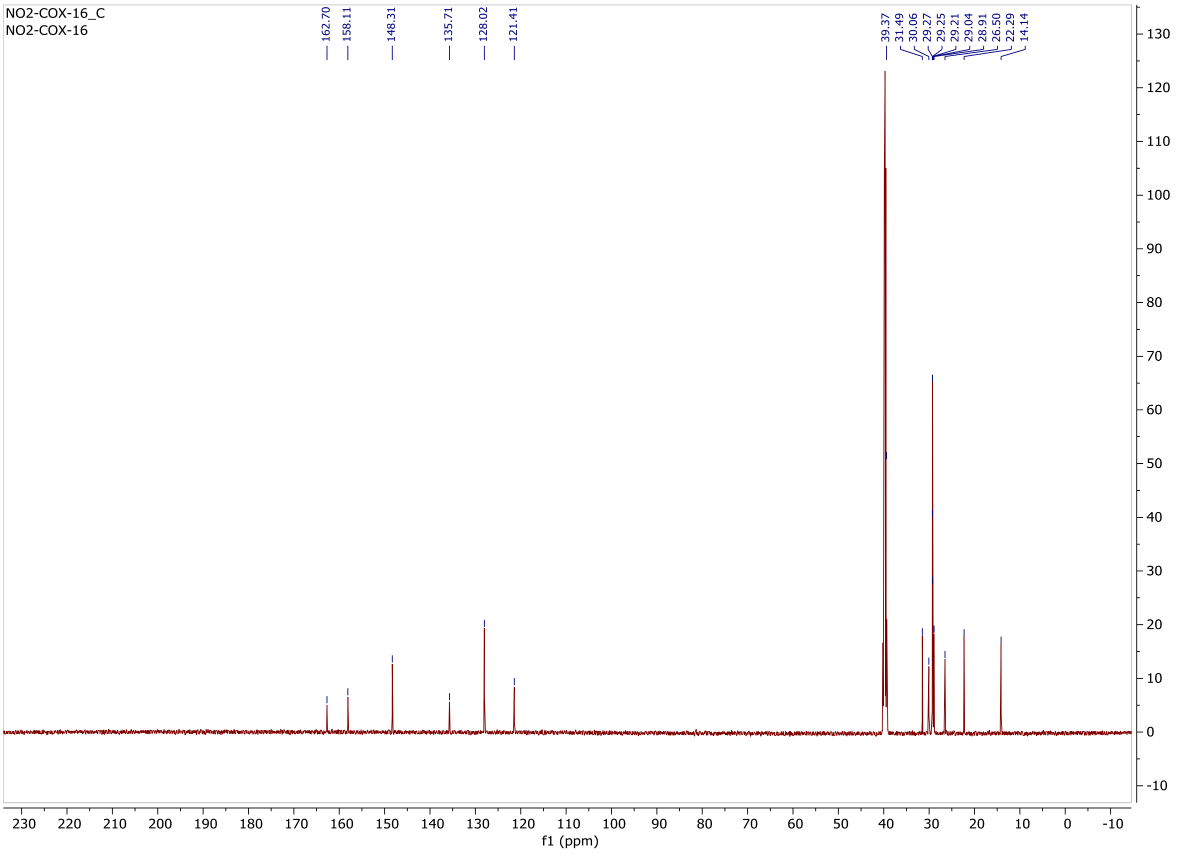

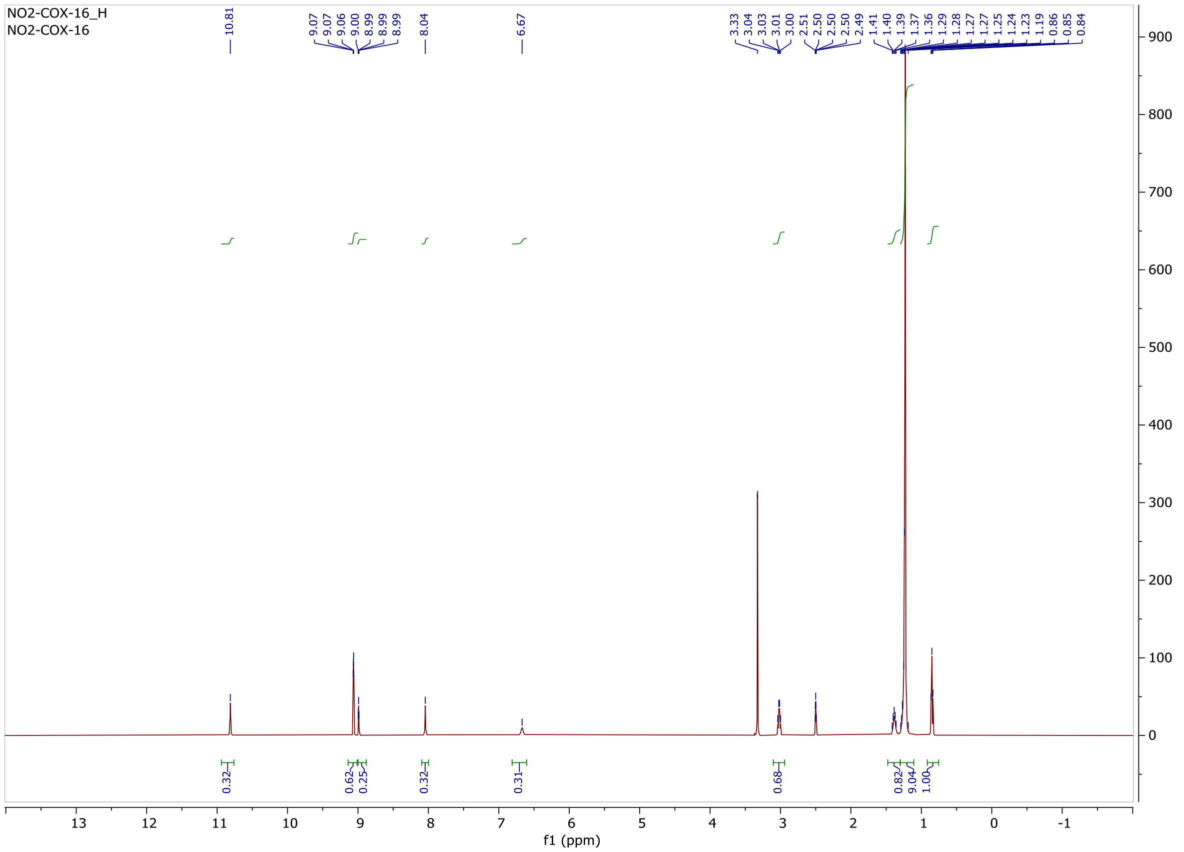
NO2-COX-16_H
NO2-COX-16
10.81
9.07
9.07
9.06
9.00
8.99
8.99
8.99
8.04
6.67
3.33
3.04
3.03
3.01
3.00
2.51
2.50
2.50
2.50
2.49
1.41
1.40
1.39
1.37
1.36
1.29
1.28
1.27
1.27
1.25
1.24
1.23
1.19
0.86
0.85
0.84
0.32
0.62
0.25
0.32
0.31
0.68
0.82
9.04
1.00
f1 (ppm)

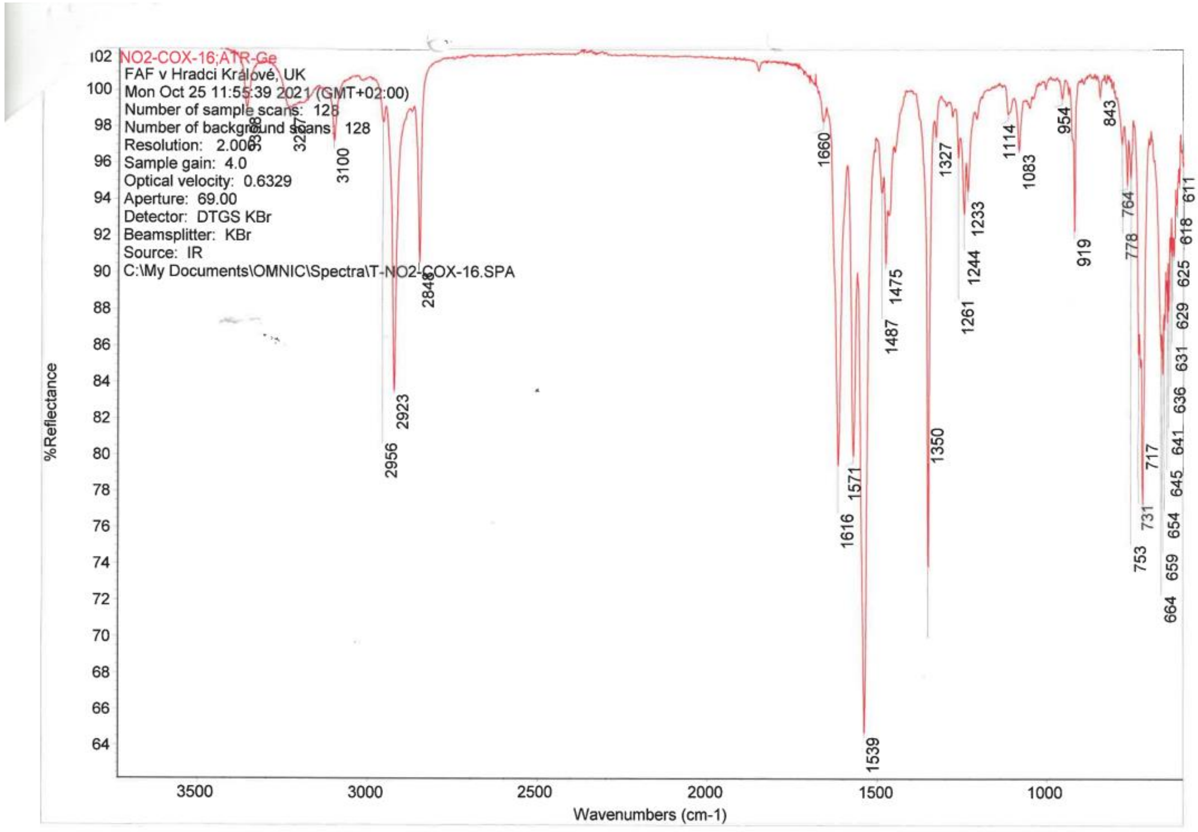
NO2-COX-16;ATR-Ge
FAF v Hradci Králové, UK
Mon Oct 25 11:55:39 2021 (GMT+02:00)
Number of sample scans: 128
Number of background scans: 128
Resolution: 2.000
Sample gain: 4.0
Optical velocity: 0.6329
Aperture: 69.00
Detector: DTGS KBr
Beamsplitter: KBr
Source: IR
C:\My Documents\OMNIC\Spectra\T-NO2-COX-16.SPA
%Reflectance
Wavenumbers (cm-1)
3100
2956
2923
2848
1660
1616
1571
1539
1487
1475
1350
1327
1261
1244
1233
1114
1083
954
919
843
778
764
753
731
717
664
659
654
645
641
636
631
629
625
618
611

2-(3,5-Dinitrobenzoyl)-*N*-heptadecylhydrazine-1-carboxamide **4q**

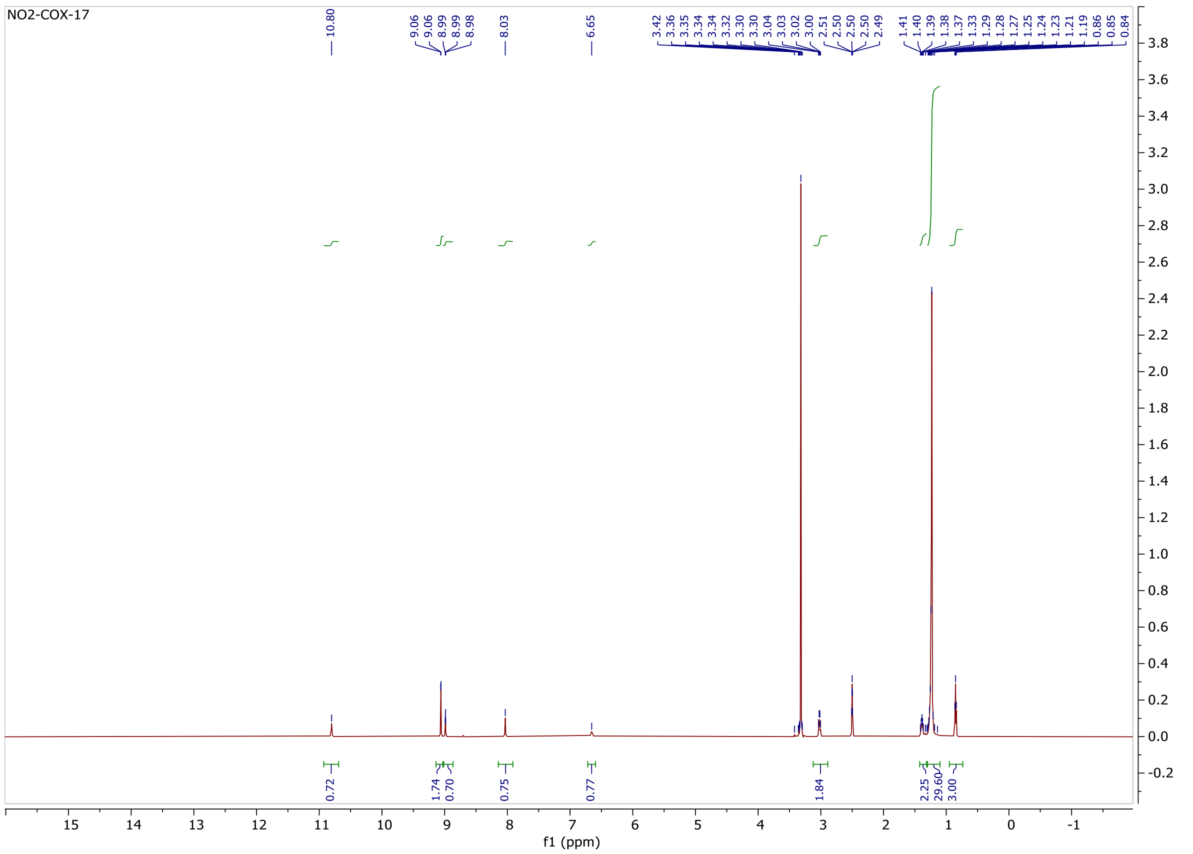

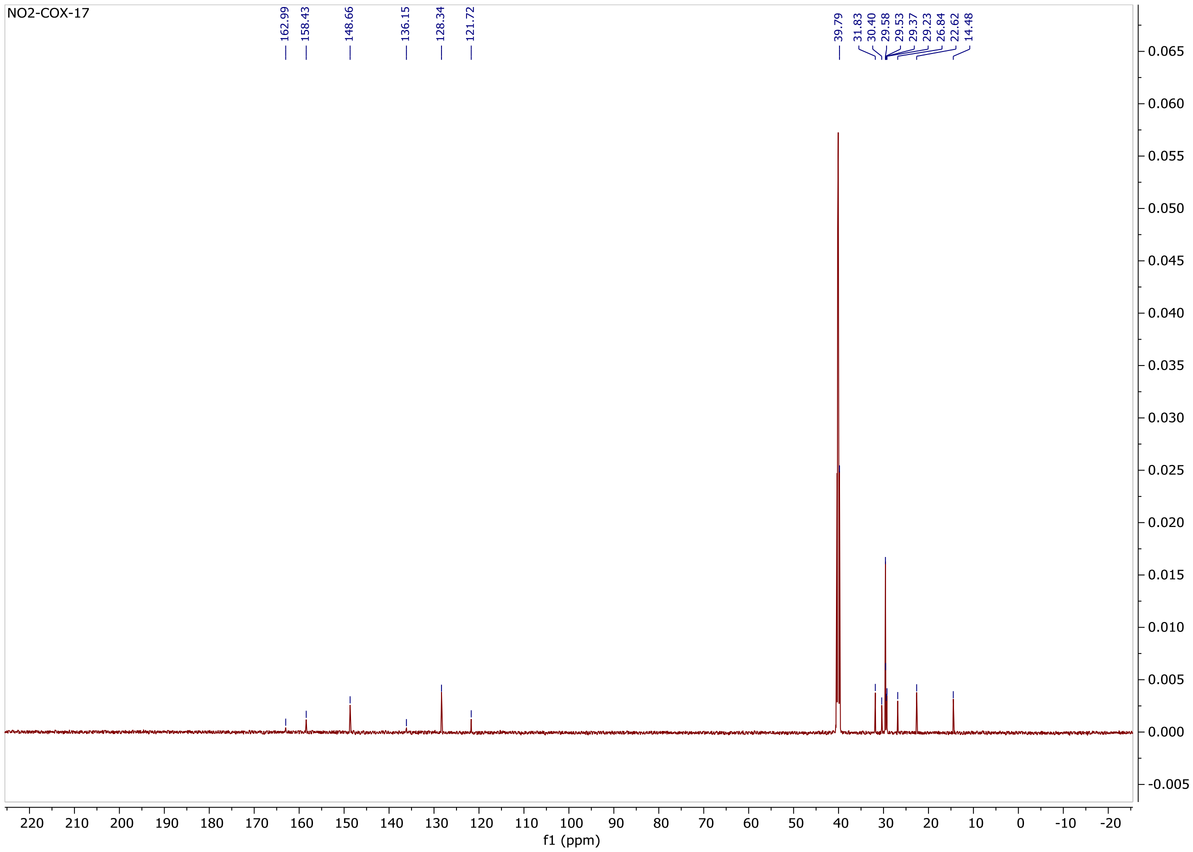

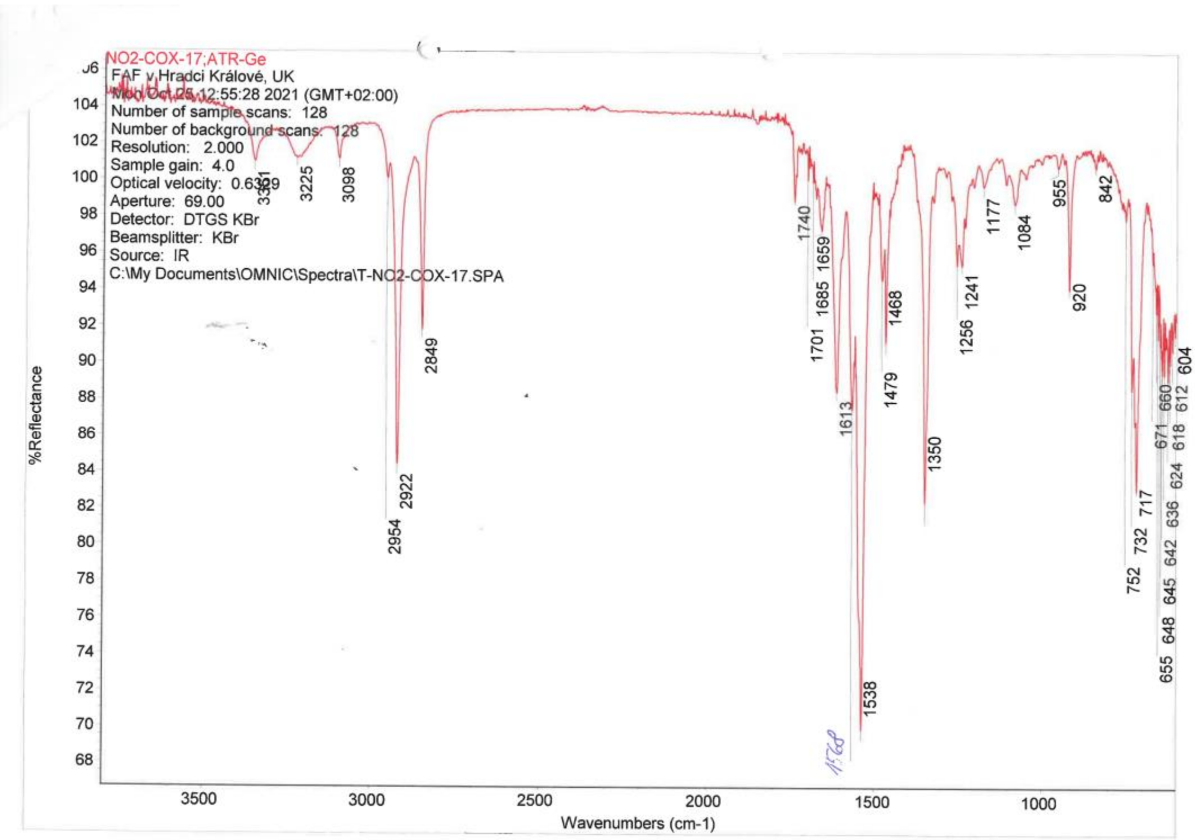

2-(3,5-Dinitrobenzoyl)-*N*-octadecylhydrazine-1-carboxamide **4r**

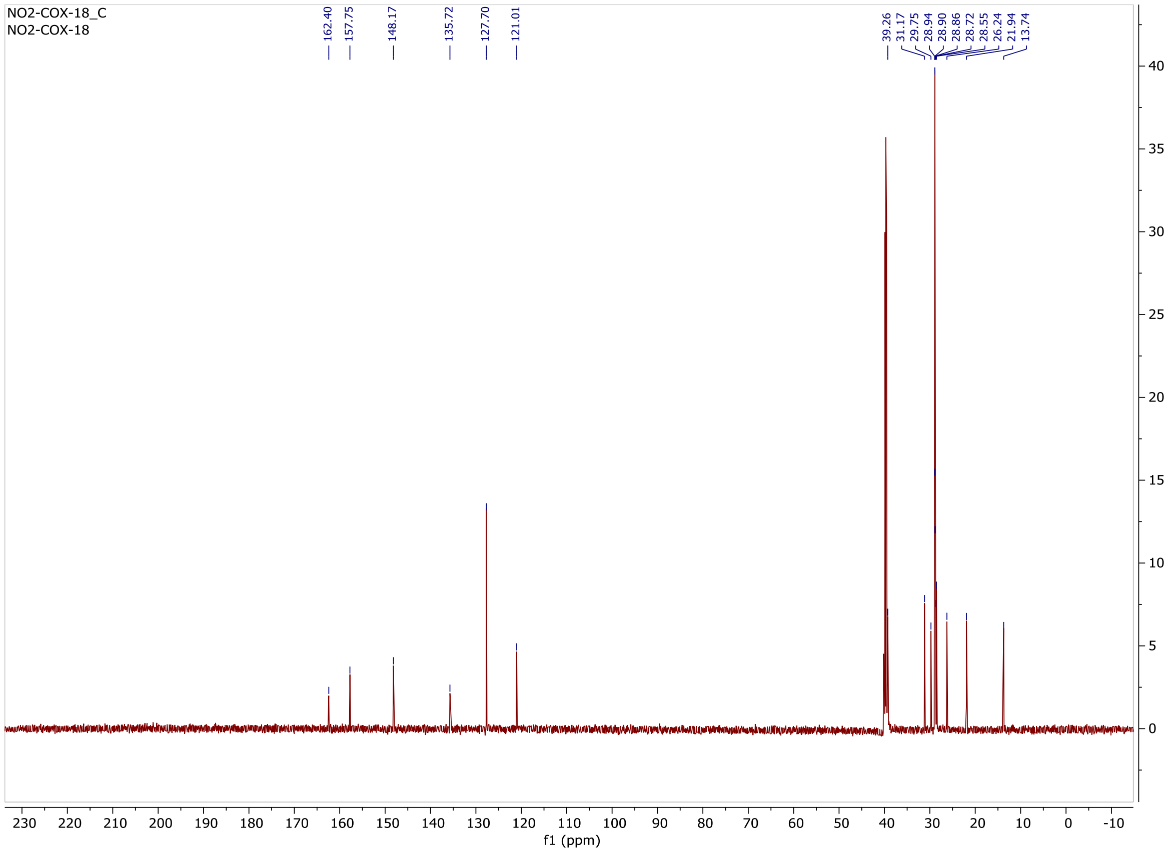

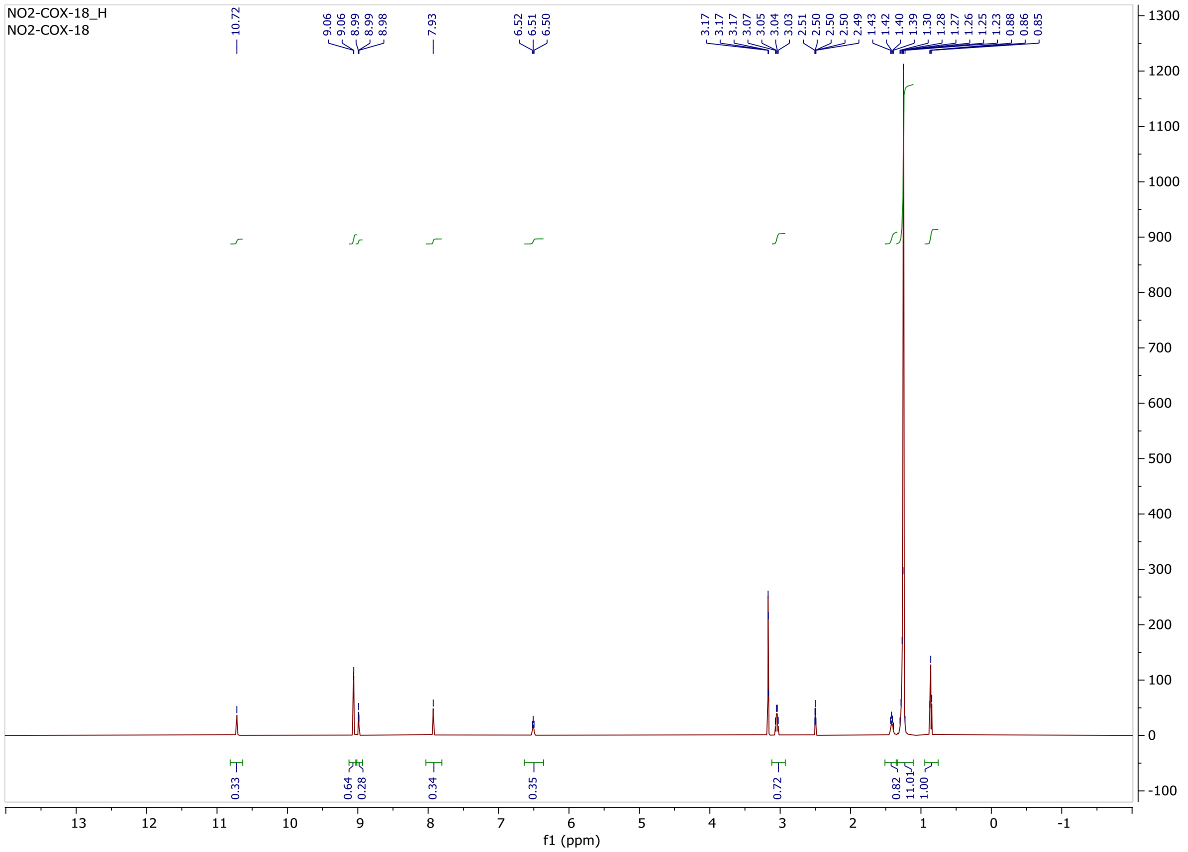
NO2-COX-18_H
NO2-COX-18
f1 (ppm)

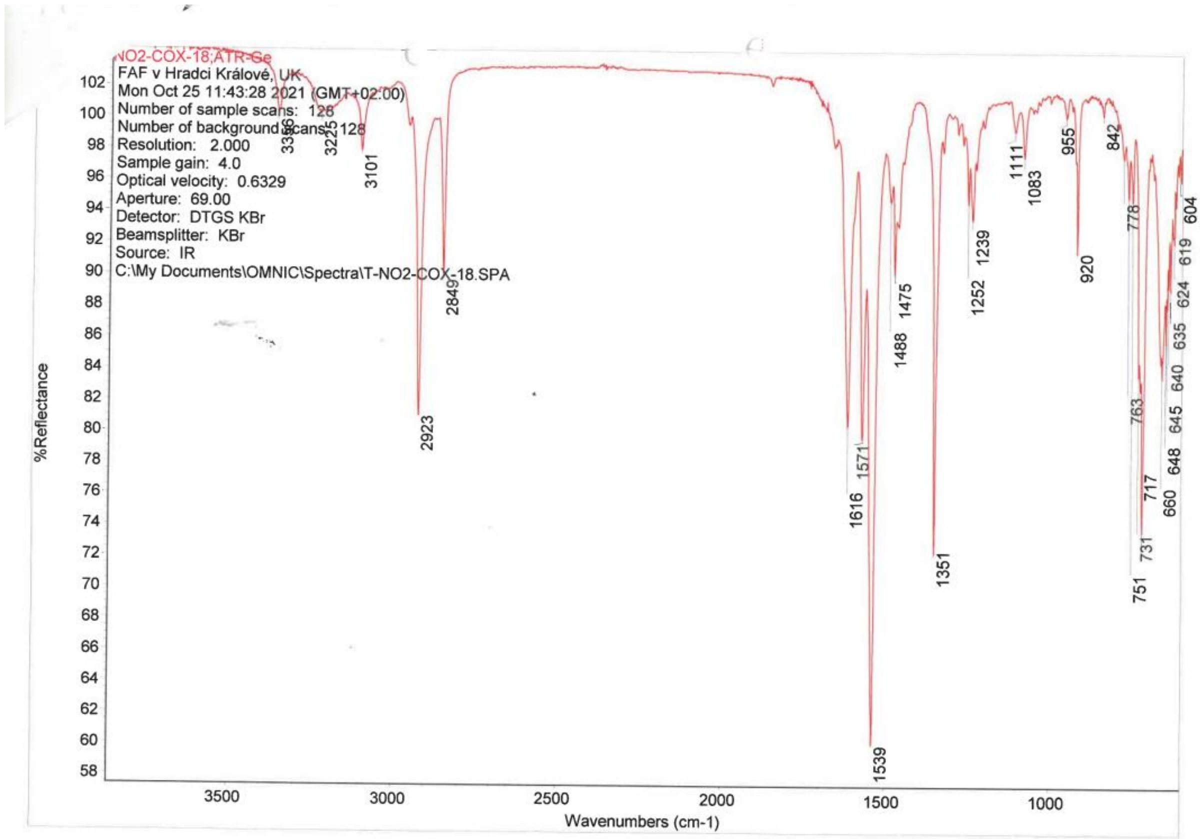
NO2-COX-18;ATR-Ge
FAF v Hradci Králové, UK
Mon Oct 25 11:43:28 2021 (GMT+02:00)
Number of sample scans: 128
Number of background scans: 128
Resolution: 2.000
Sample gain: 4.0
Optical velocity: 0.6329
Aperture: 69.00
Detector: DTGS KBr
Beamsplitter: KBr
Source: IR
C:\My Documents\OMNIC\Spectra\T-NO2-COX-18.SPA
%Reflectance
Wavenumbers (cm-1)

2-(3,5-Dinitrobenzoyl)-*N*-dodecylhydrazine-1-carbothioamide **4s**

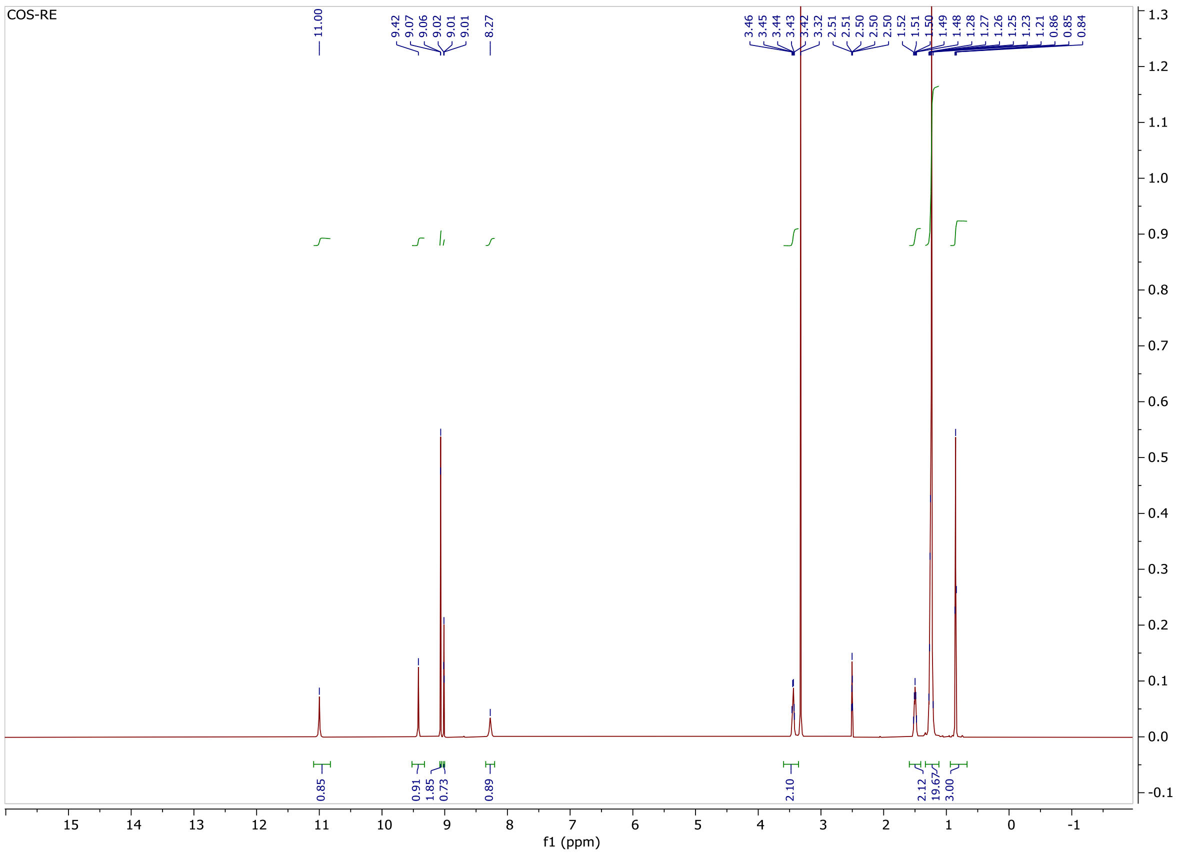

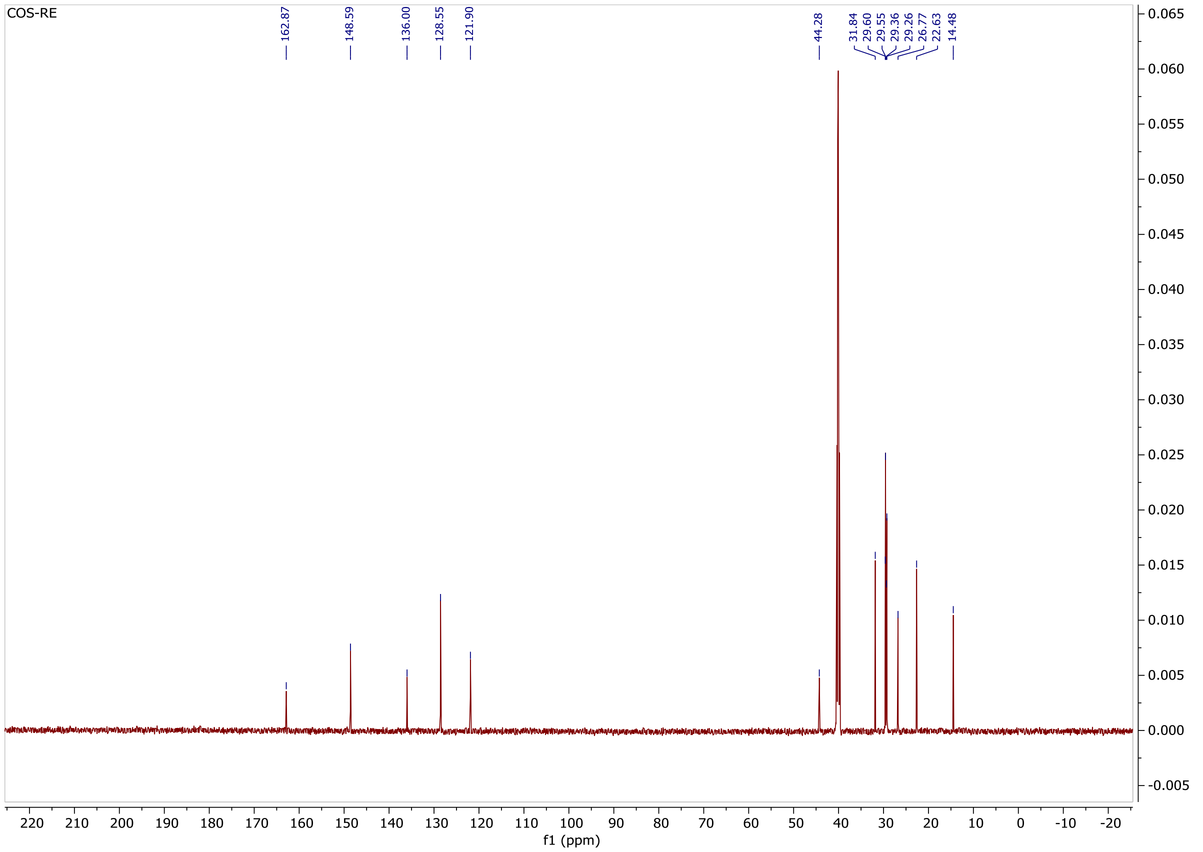

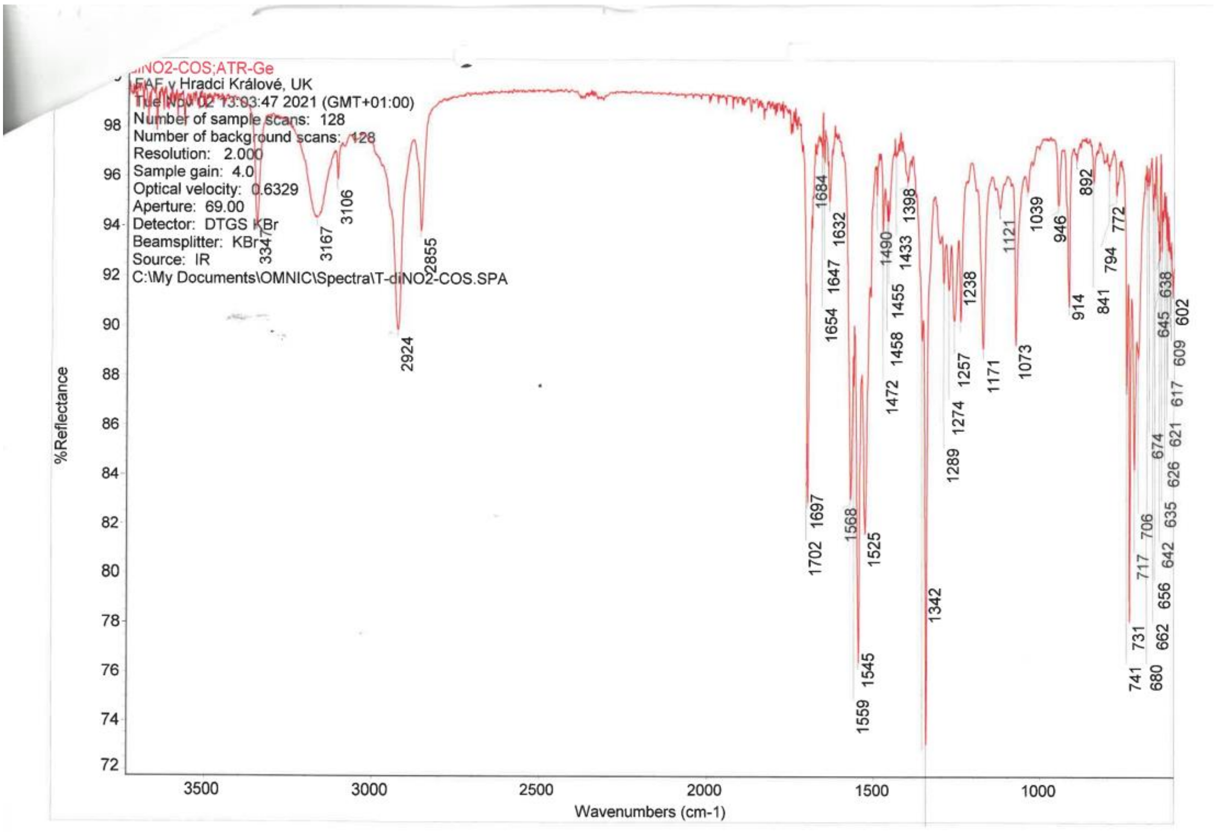

5-(3,5-Dinitrophenyl)-*N*-methyl-1,3,4-oxadiazol-2-amine **5a**

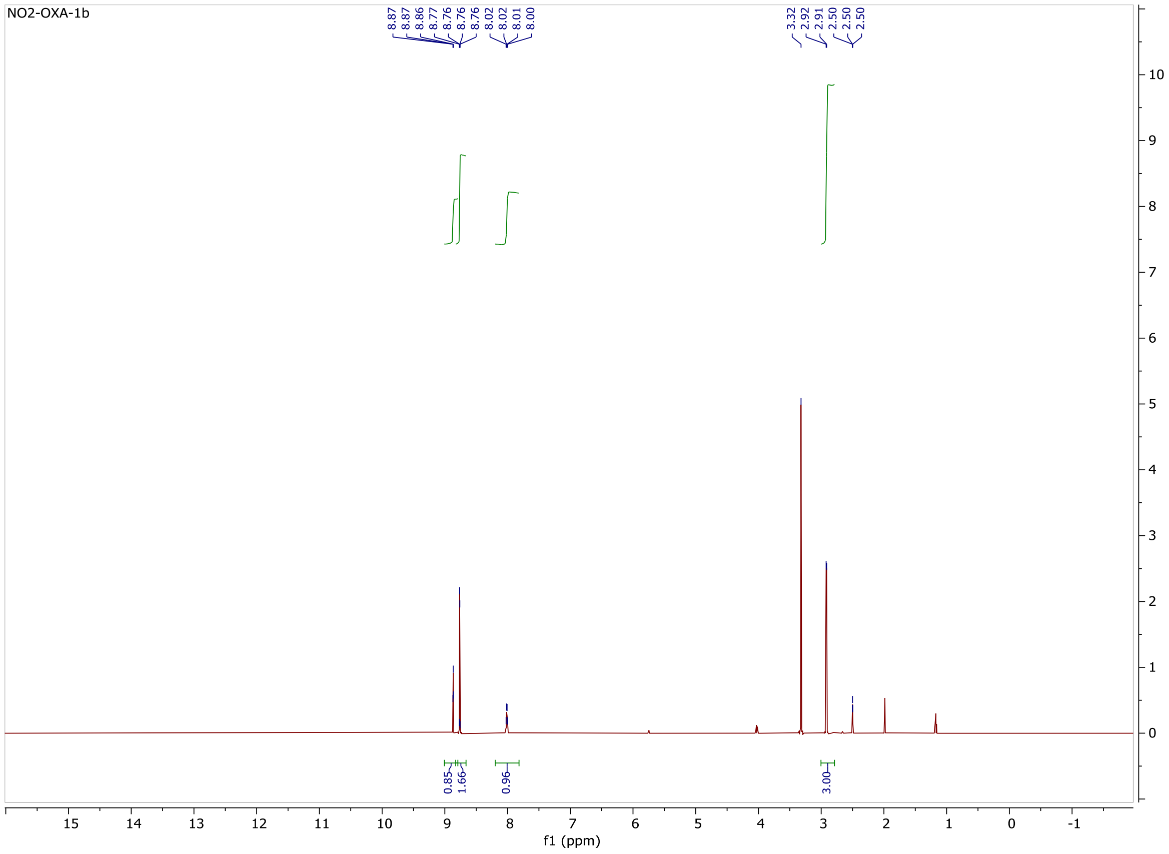

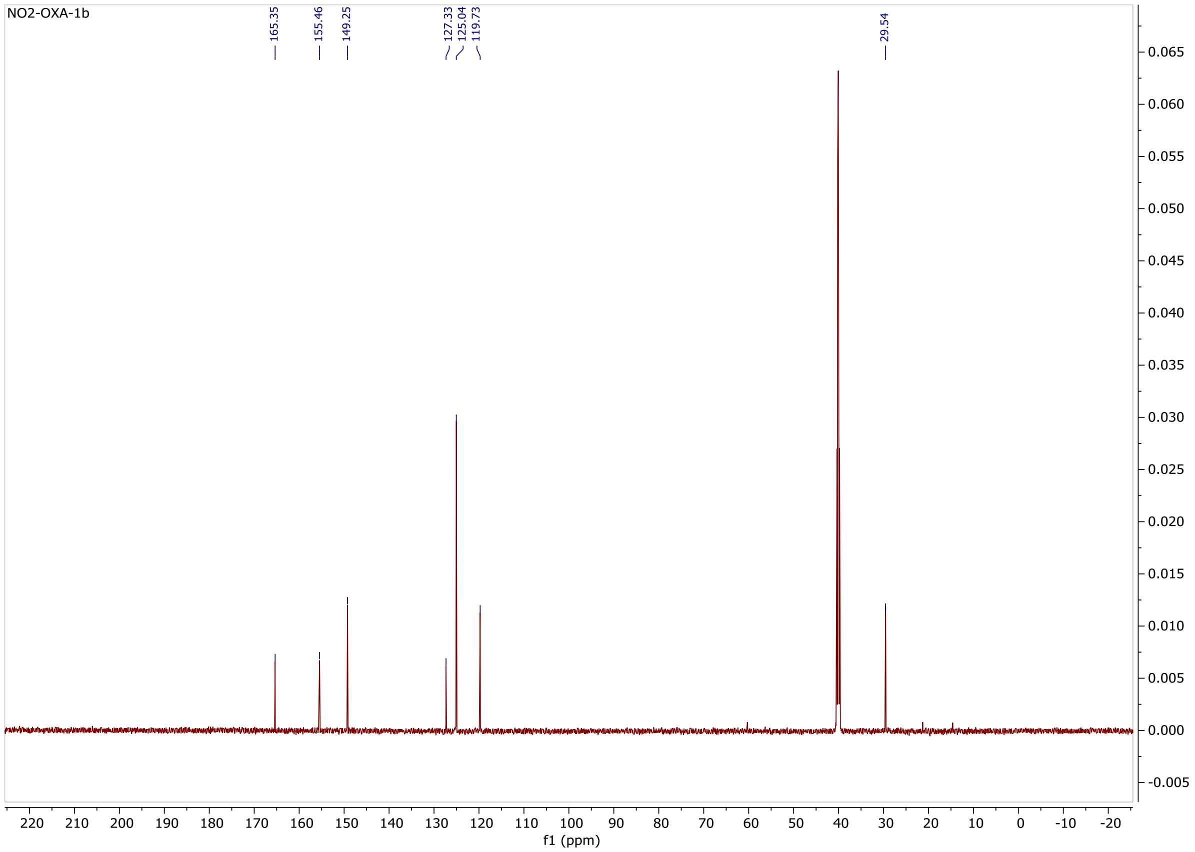
NO2-OXA-1b
165.35
155.46
149.25
127.33
125.04
119.73
29.54
0.065
0.060
0.055
0.050
0.045
0.040
0.035
0.030
0.025
0.020
0.015
0.010
0.005
0.000
-0.005
220 210 200 190 180 170 160 150 140 130 120 110 100 90 80 70 60 50 40 30 20 10 0 -10 -20
f1 (ppm)

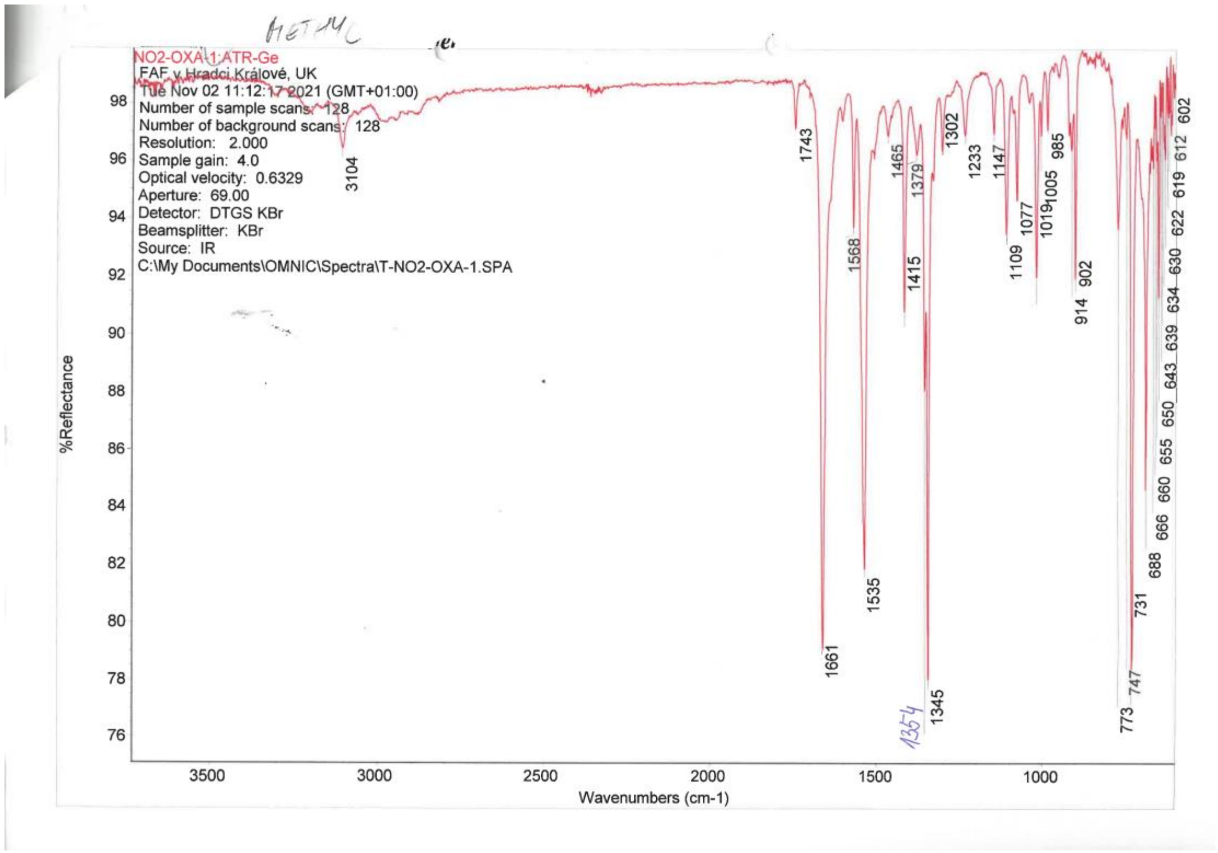
NO2-OXA-1:ATR-Ge
FAF v Hradci Králové, UK
Tue Nov 02 11:12:17 2021 (GMT+01:00)
Number of sample scans: 128
Number of background scans: 128
Resolution: 2.000
Sample gain: 4.0
Optical velocity: 0.6329
Aperture: 69.00
Detector: DTGS KBr
Beamsplitter: KBr
Source: IR
C:\My Documents\OMNIC\Spectra\T-NO2-OXA-1.SPA
%Reflectance
98
96
94
92
90
88
86
84
82
80
78
76
3500 3000 2500 2000 1500 1000
Wavenumbers (cm-1)
3104
1743
1661
1568
1535
1465
1415
1379
1345
1354
1302
1233
1147
1109
1077
1019
1005
985
914
902
773
747
731
688
666
660
655
650
643
639
634
630
622
619
612
602

5-(3,5-Dinitrophenyl)-*N*-ethyl-1,3,4-oxadiazol-2-amine **5b**

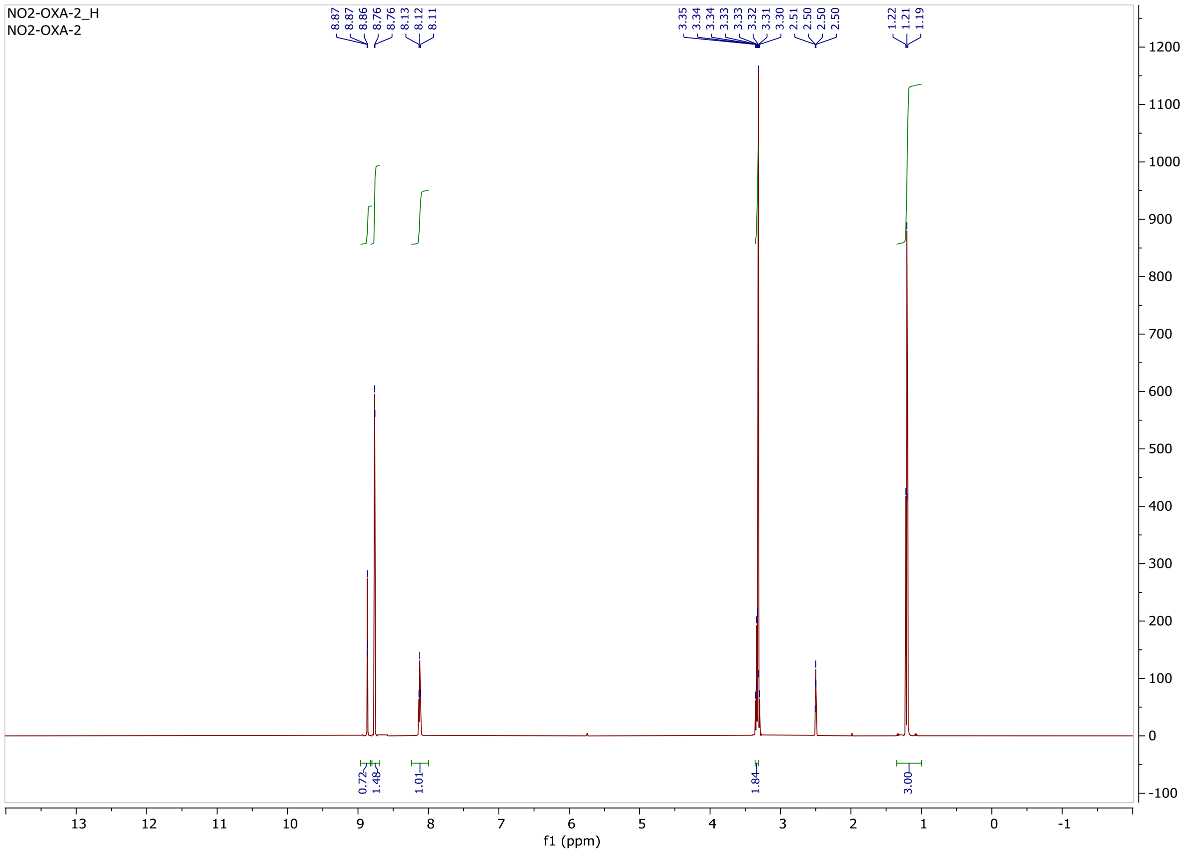

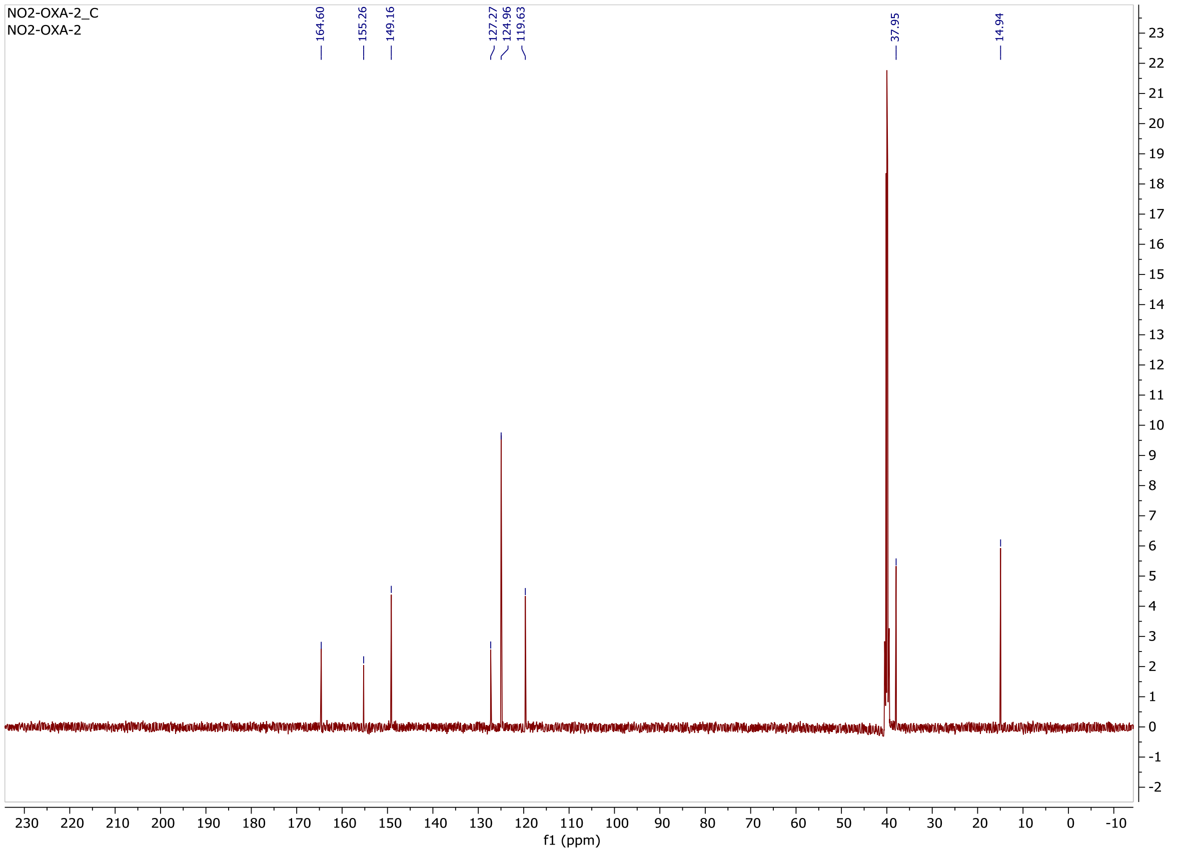

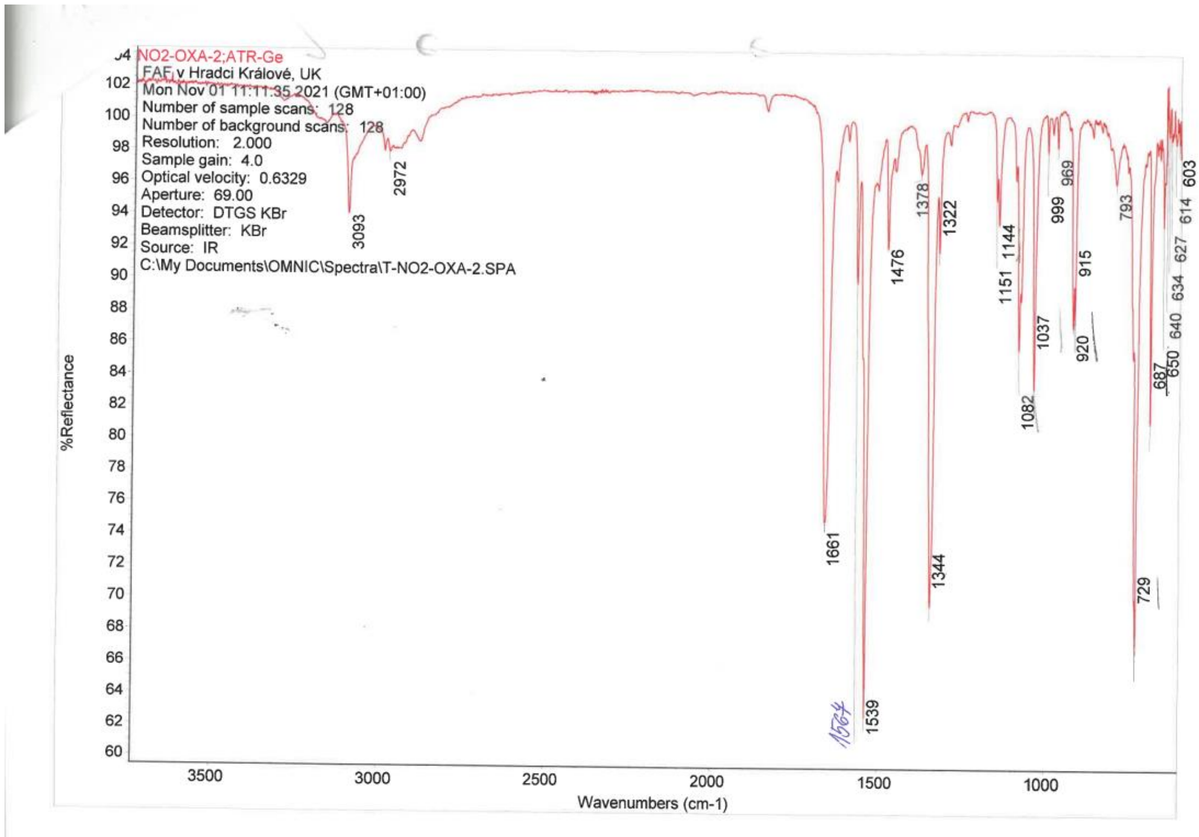

5-(3,5-Dinitrophenyl)-*N*-propyl-1,3,4-oxadiazol-2-amine **5c**

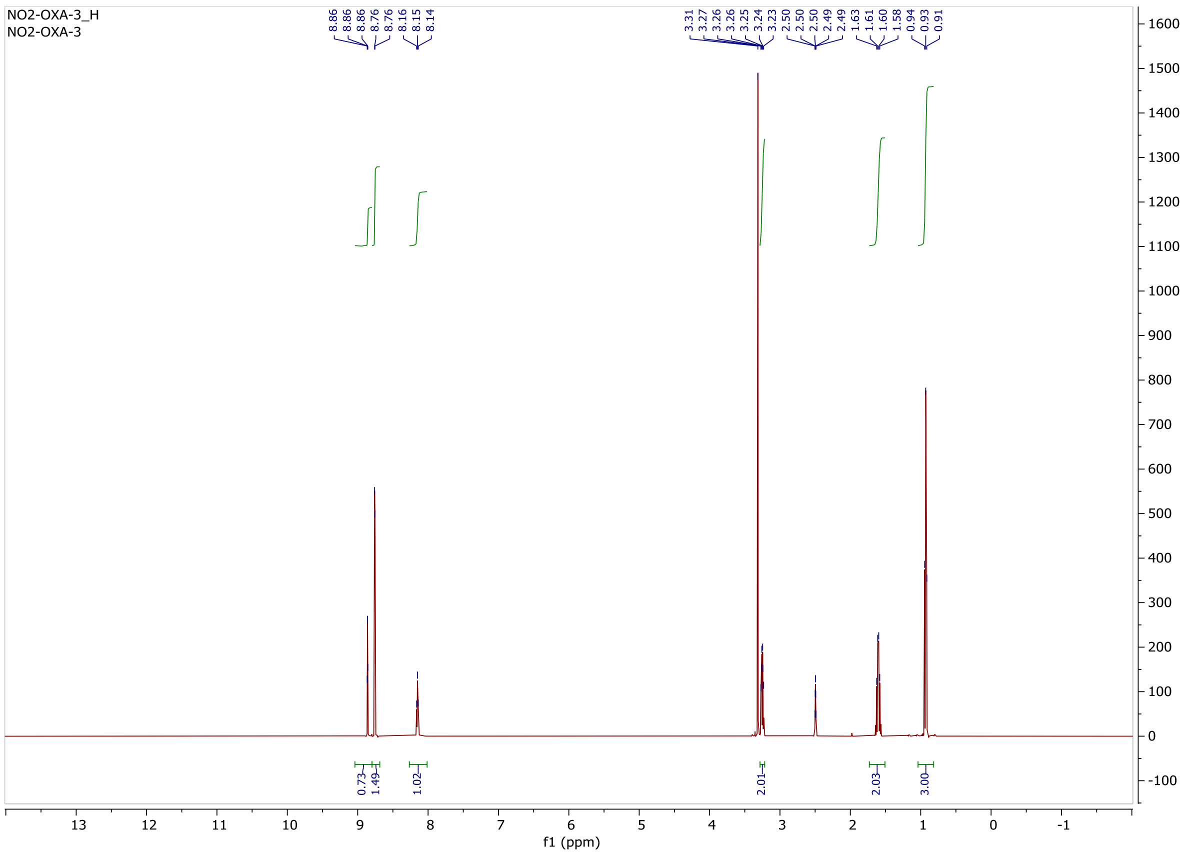

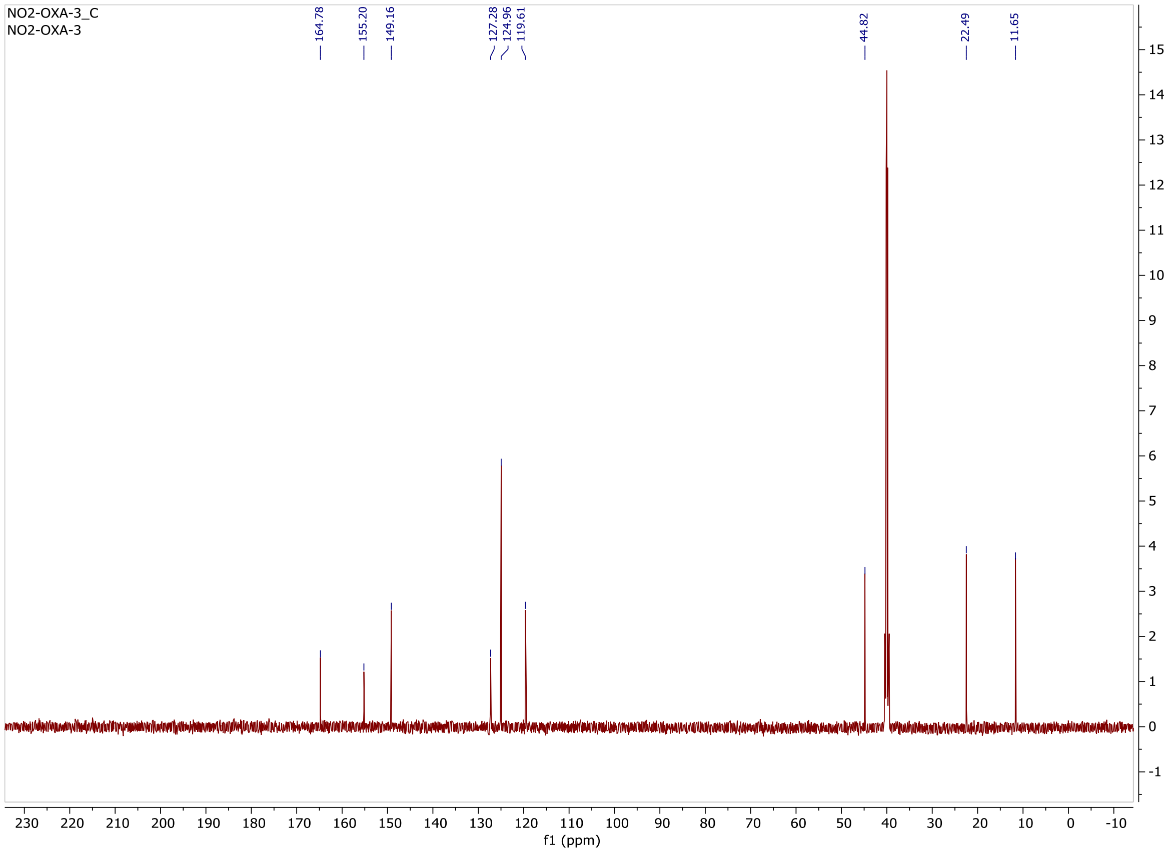
NO2-OXA-3_C
NO2-OXA-3
164.78
155.20
149.16
127.28
124.96
119.61
44.82
22.49
11.65
f1 (ppm)

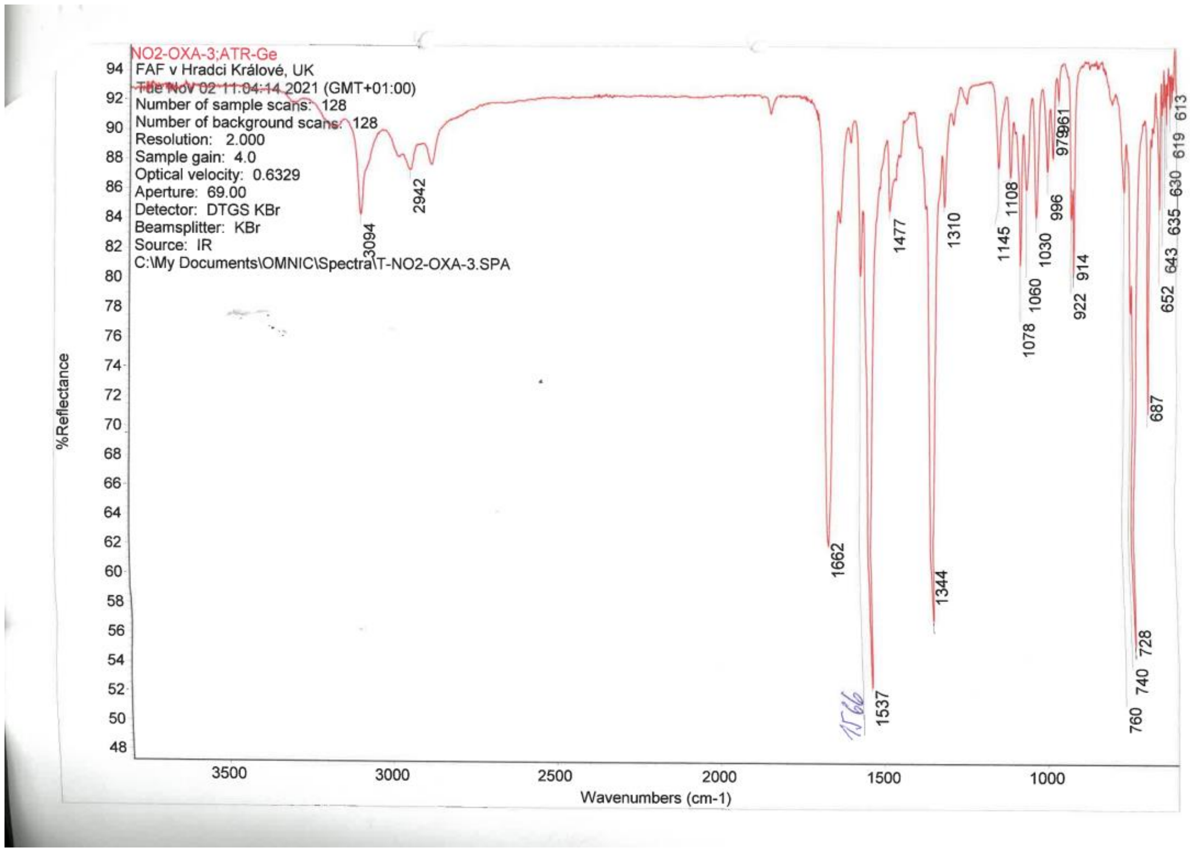
NO2-OXA-3;ATR-Ge
FAF v Hradci Králové, UK
Tue Nov 02 11:04:14 2021 (GMT+01:00)
Number of sample scans: 128
Number of background scans: 128
Resolution: 2.000
Sample gain: 4.0
Optical velocity: 0.6329
Aperture: 69.00
Detector: DTGS KBr
Beamsplitter: KBr
Source: IR
C:\My Documents\OMNIC\Spectra\T-NO2-OXA-3.SPA
3094
2942
1662
1566
1537
1477
1344
1310
1145
1108
1078
1060
1030
996
979
961
922
914
760
740
728
687
652
643
635
630
619
613
%Reflectance
Wavenumbers (cm-1)

N-Butyl-5-(3,5-dinitrophenyl)-1,3,4-oxadiazol-2-amine **5d**

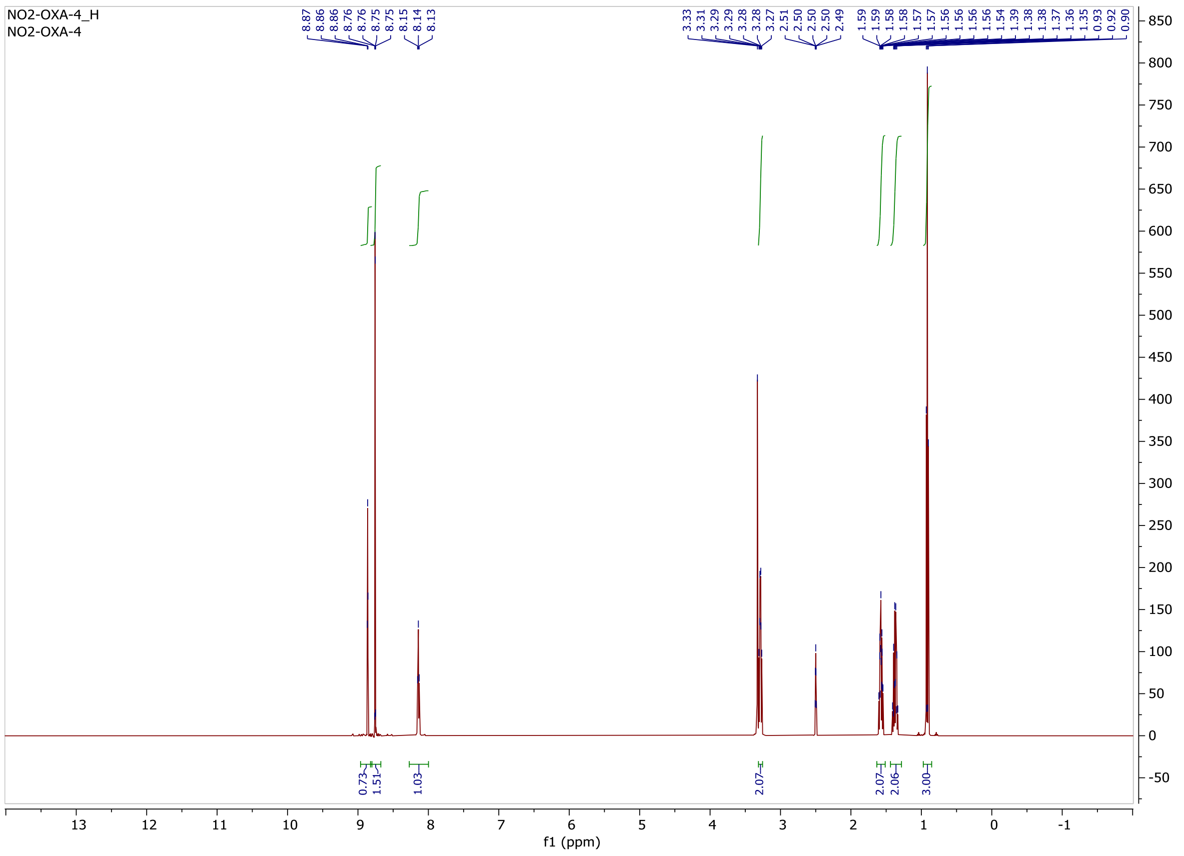

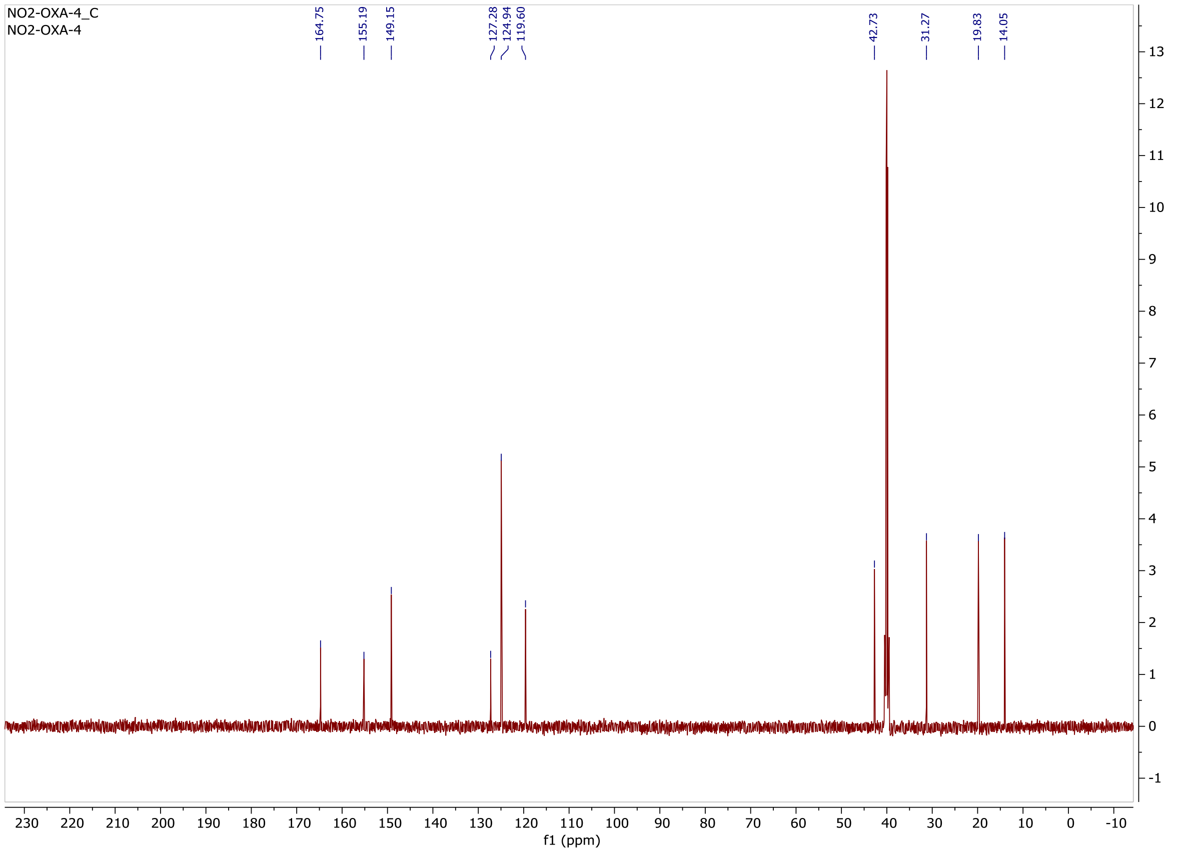

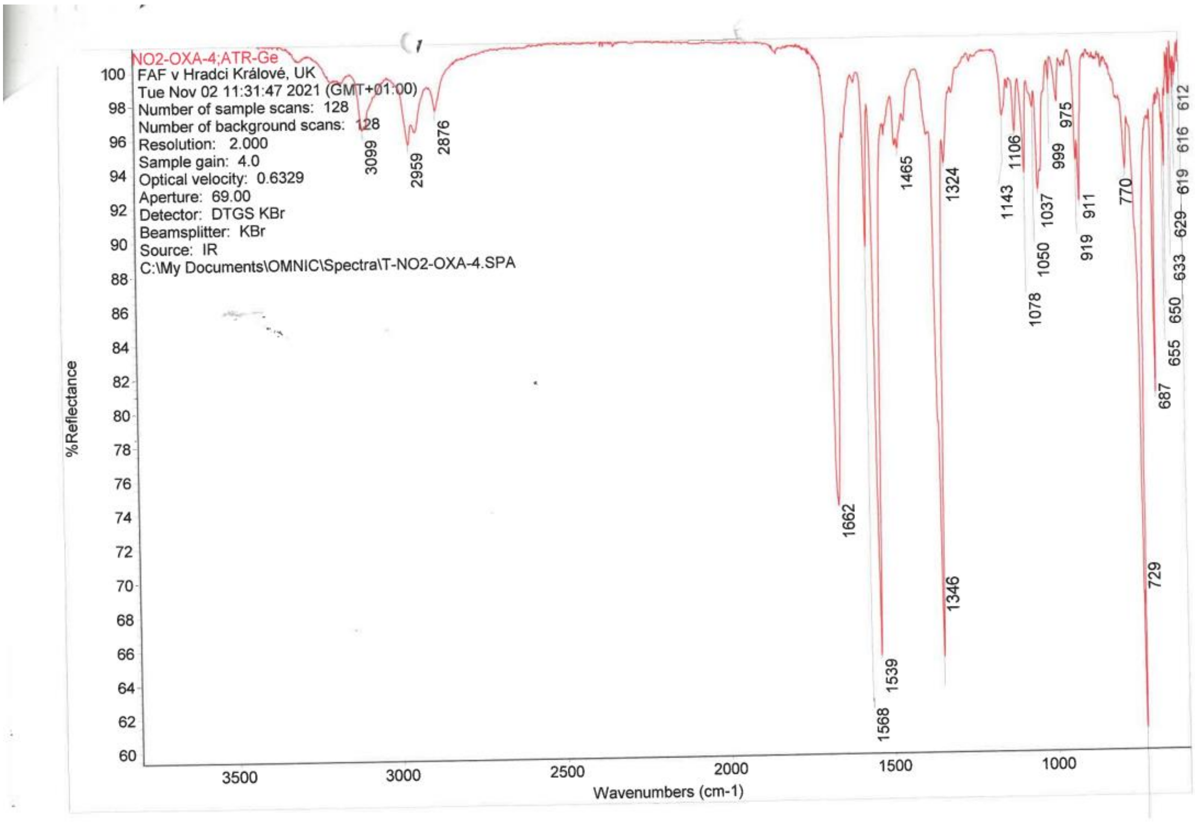

5-(3,5-Dinitrophenyl)-*N*-pentyl-1,3,4-oxadiazol-2-amine **5e**

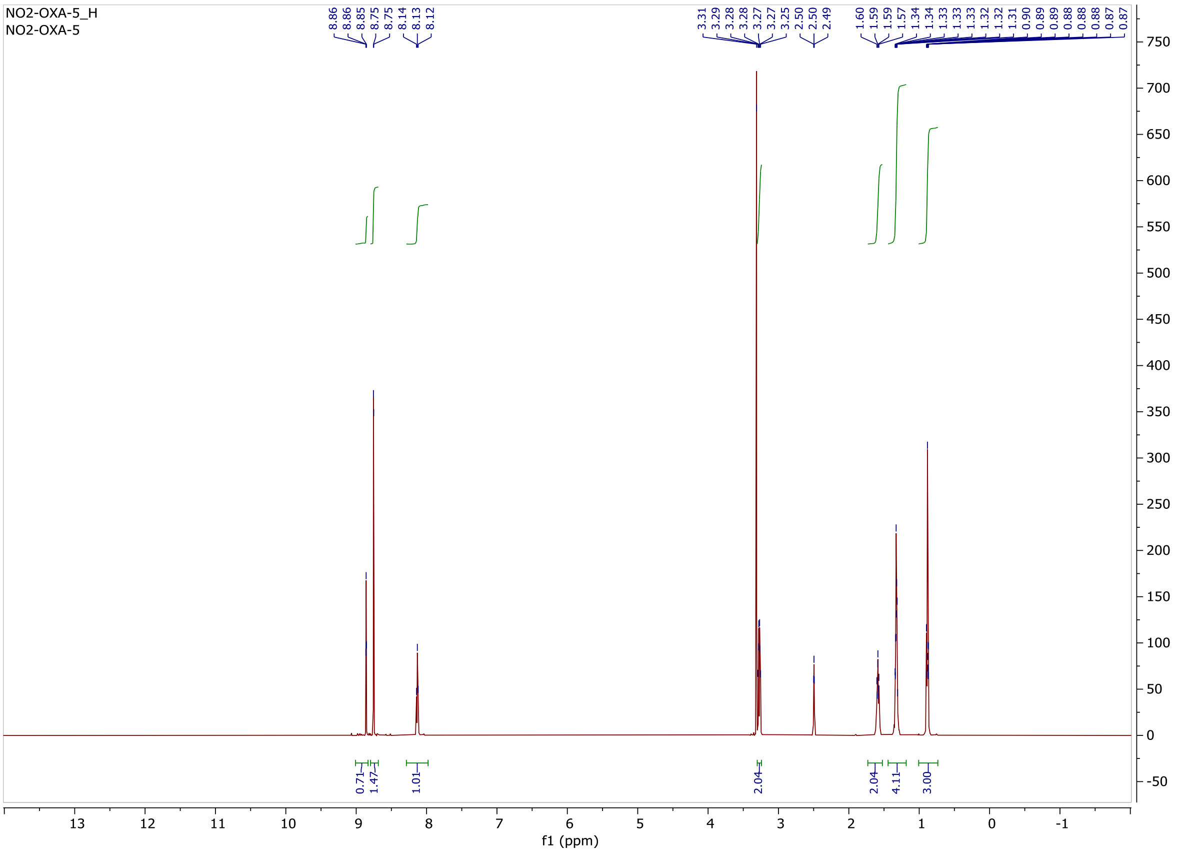

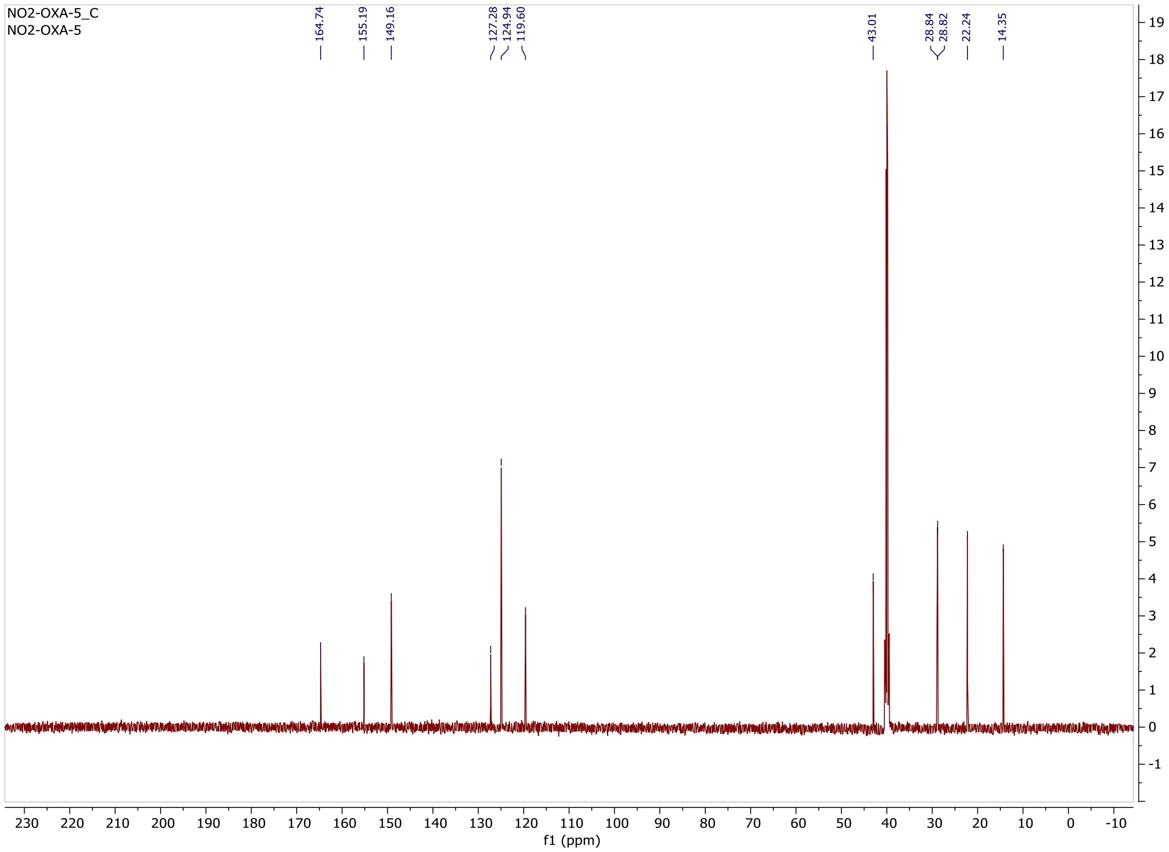
NO2-OXA-5_C
NO2-OXA-5
164.74
155.19
149.16
127.28
124.94
119.60
43.01
28.84
28.82
22.24
14.35
f1 (ppm)

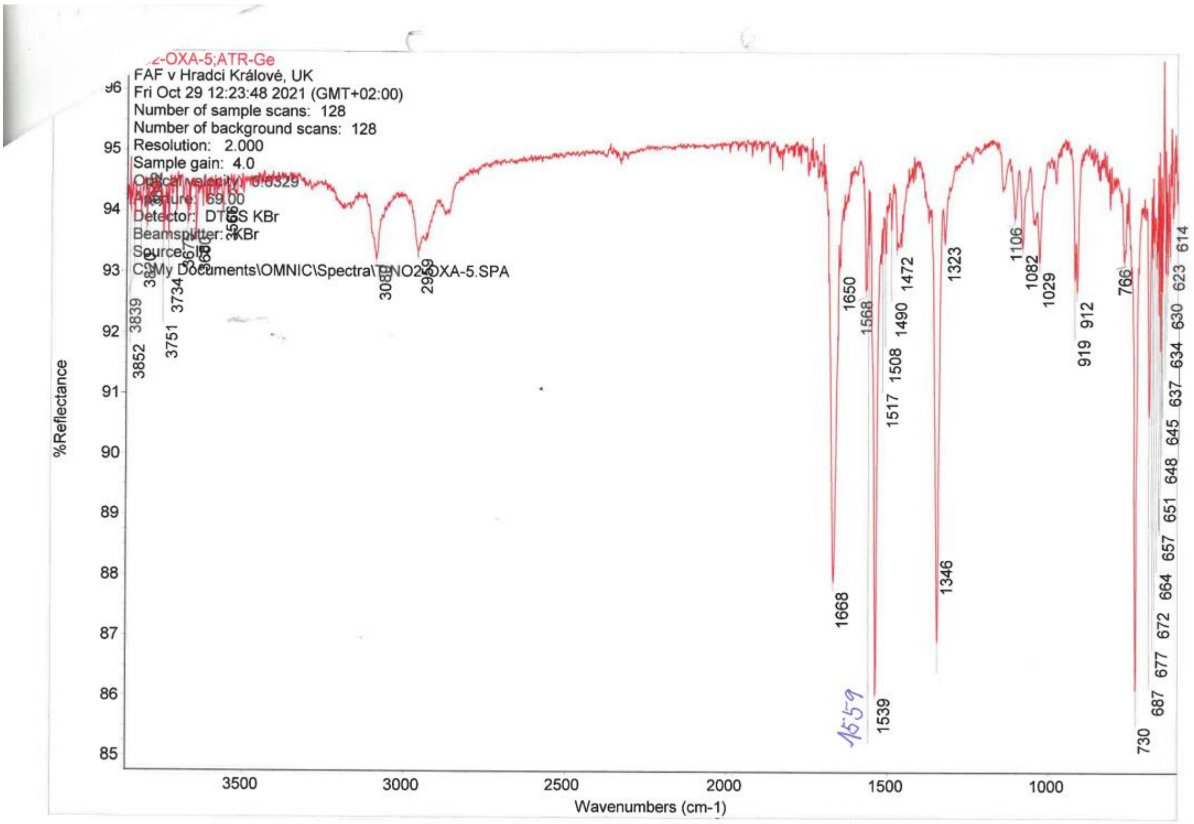
FAF v Hradci Králové, UK
Fri Oct 29 12:23:48 2021 (GMT+02:00)
Number of sample scans: 128
Number of background scans: 128
Resolution: 2.000
Sample gain: 4.0
C:\My Documents\OMNIC\Spectra\NO2-OXA-5.SPA
%Reflectance
Wavenumbers (cm-1)
1668
1650
1568
1539
1517
1508
1490
1472
1346
1323
1106
1082
1029
919
912
766
730
1559

5-(3,5-Dinitrophenyl)-*N*-hexyl-1,3,4-oxadiazol-2-amine **5f**

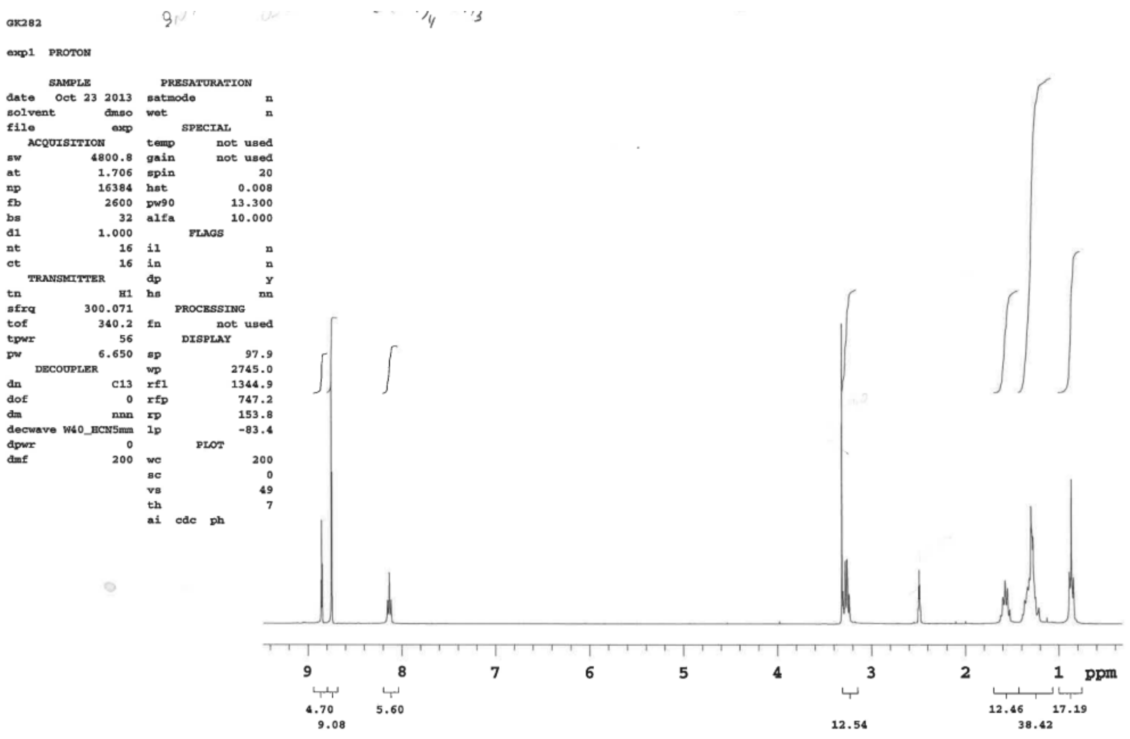

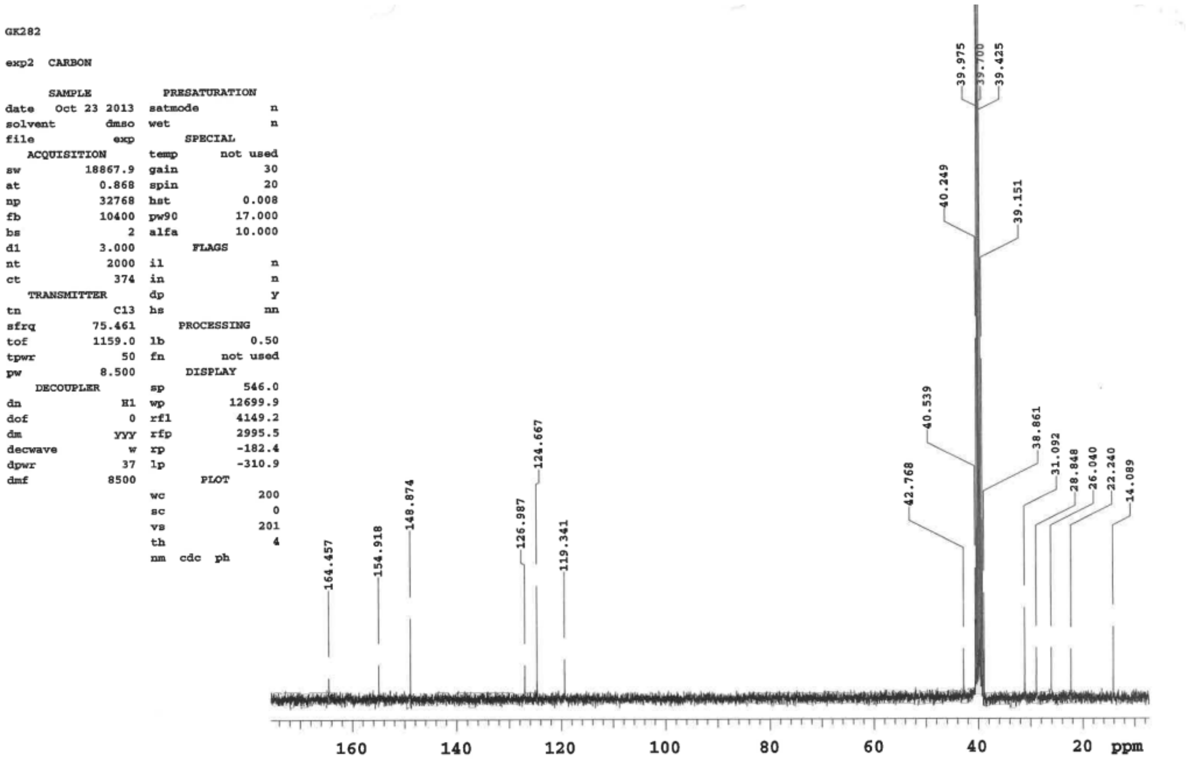

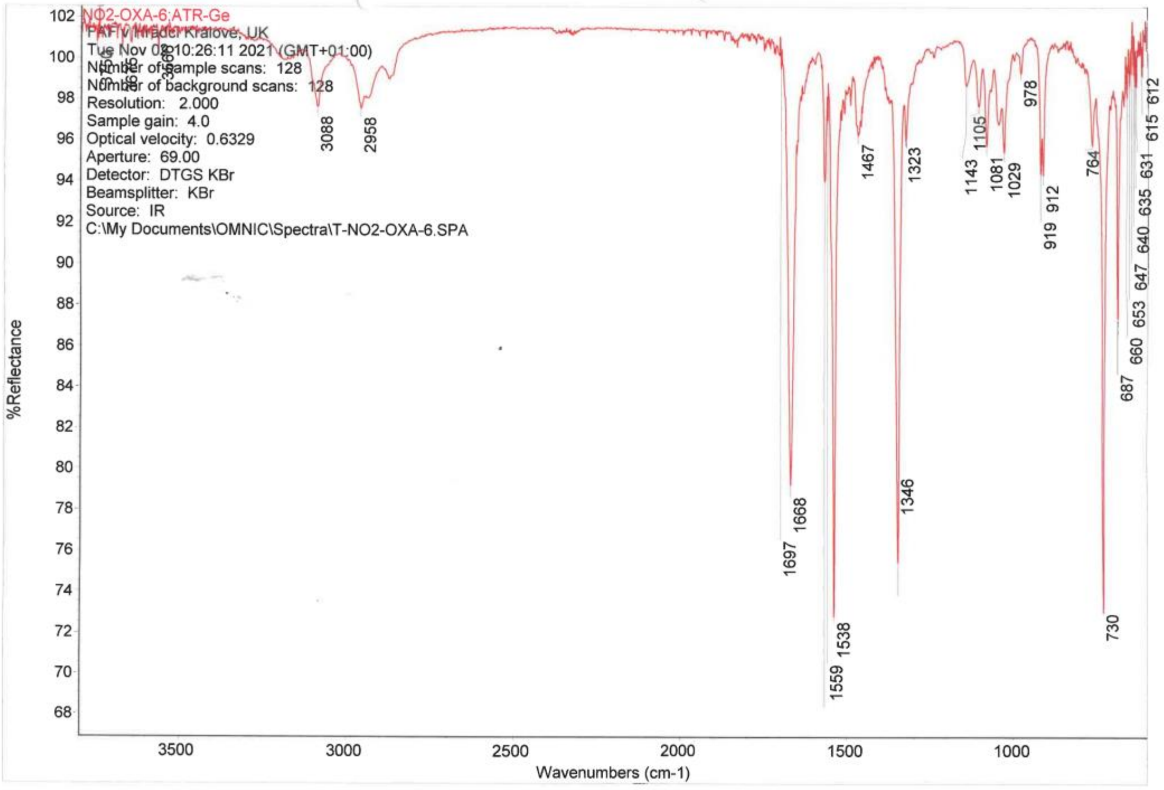

5-(3,5-Dinitrophenyl)-*N*-heptyl-1,3,4-oxadiazol-2-amine **5g**

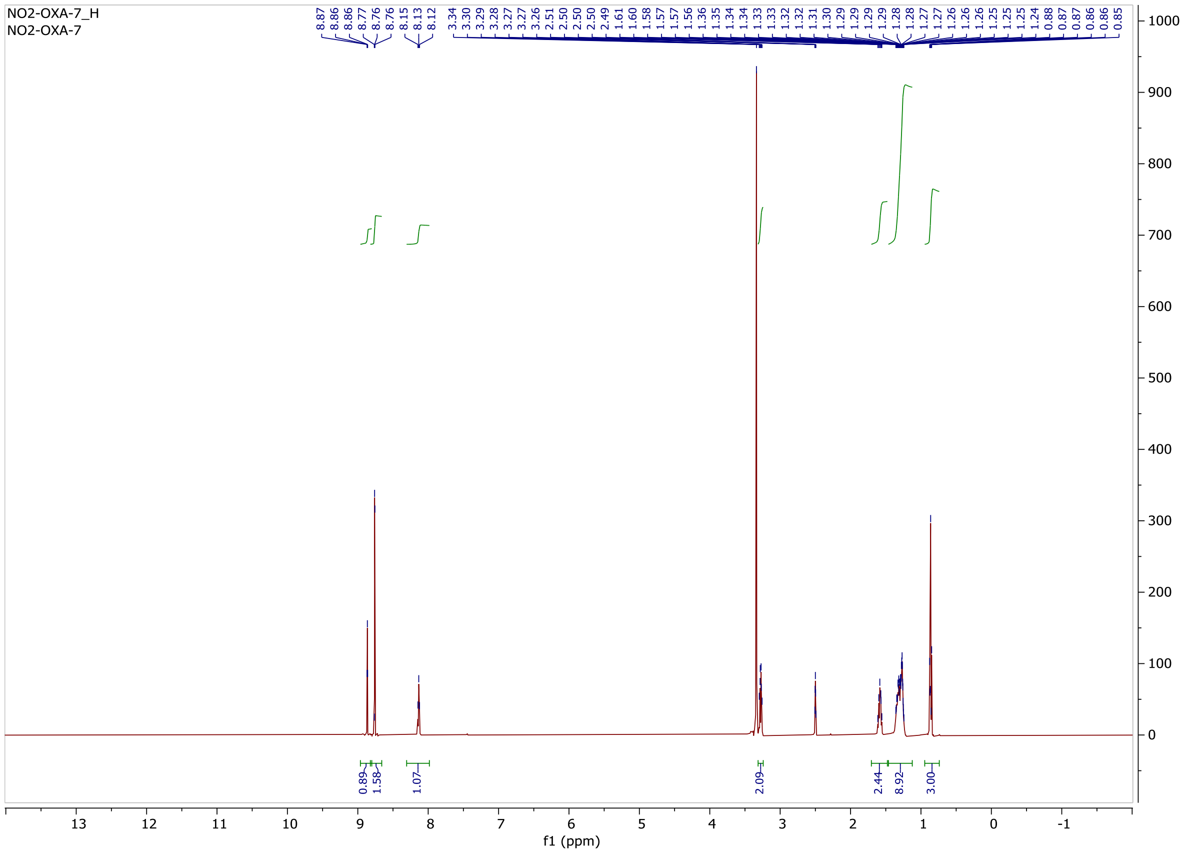

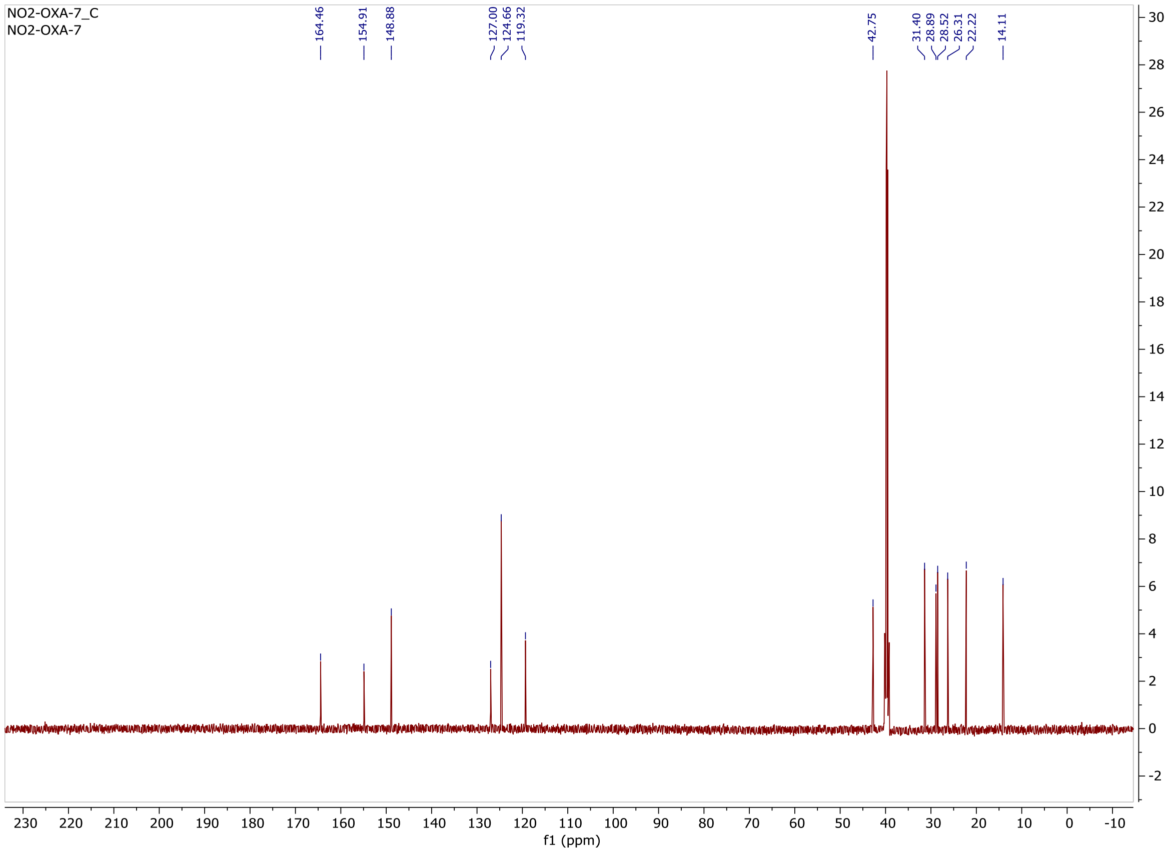
NO2-OXA-7_C
NO2-OXA-7
164.46
154.91
148.88
127.00
124.66
119.32
42.75
31.40
28.89
28.52
26.31
22.22
14.11
f1 (ppm)

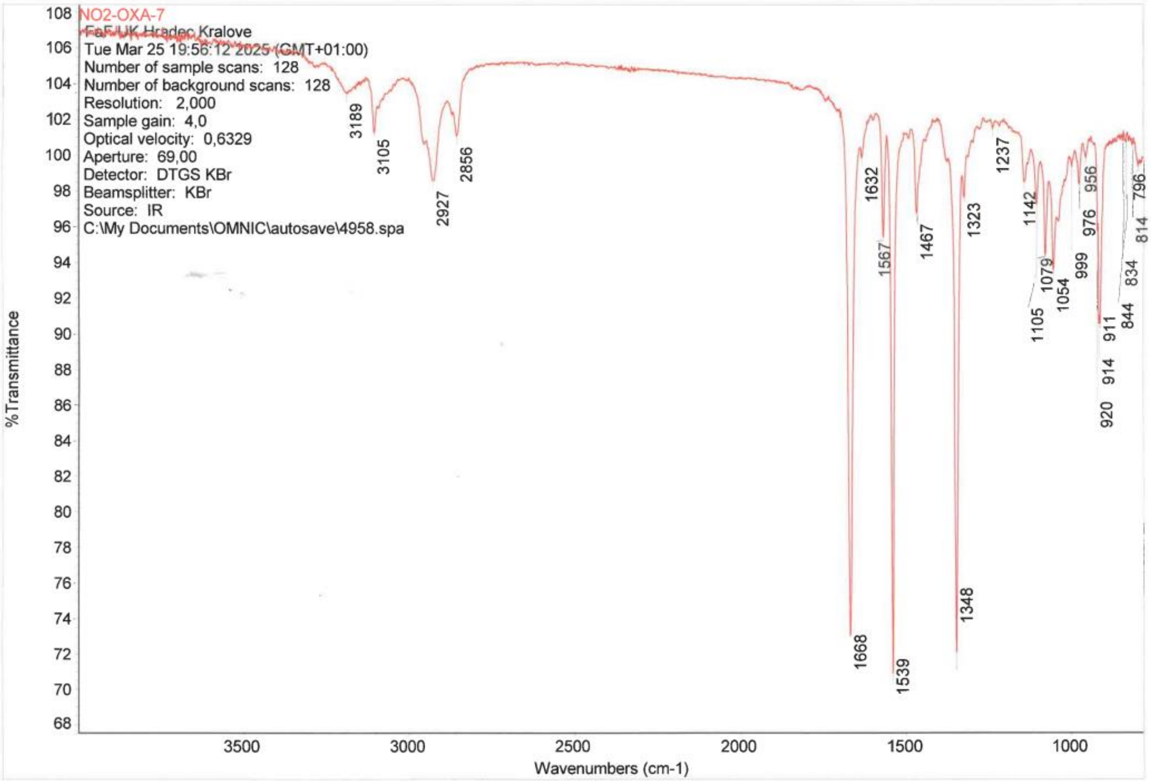
NO2-OXA-7
FaF/UK Hradec Kralove
Tue Mar 25 19:56:12 2025 (GMT+01:00)
Number of sample scans: 128
Number of background scans: 128
Resolution: 2,000
Sample gain: 4,0
Optical velocity: 0,6329
Aperture: 69,00
Detector: DTGS KBr
Beamsplitter: KBr
Source: IR
C:\My Documents\OMNIC\autosave\4958.spa
%Transmittance
Wavenumbers (cm-1)
3189
3105
2927
2856
1668
1632
1567
1539
1467
1348
1323
1237
1142
1105
1079
1054
999
976
956
920
914
911
844
834
814
796

5-(3,5-Dinitrophenyl)-*N*-octyl-1,3,4-oxadiazol-2-amine **5h**

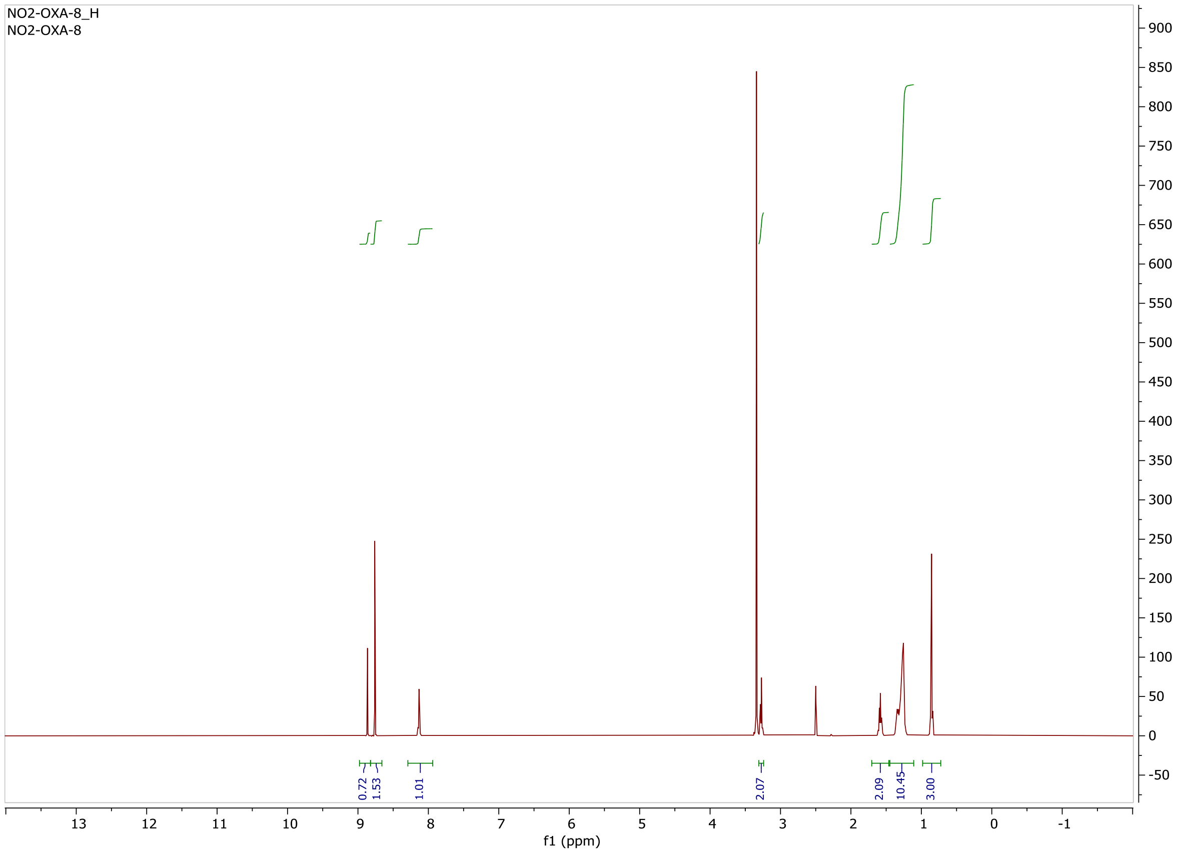

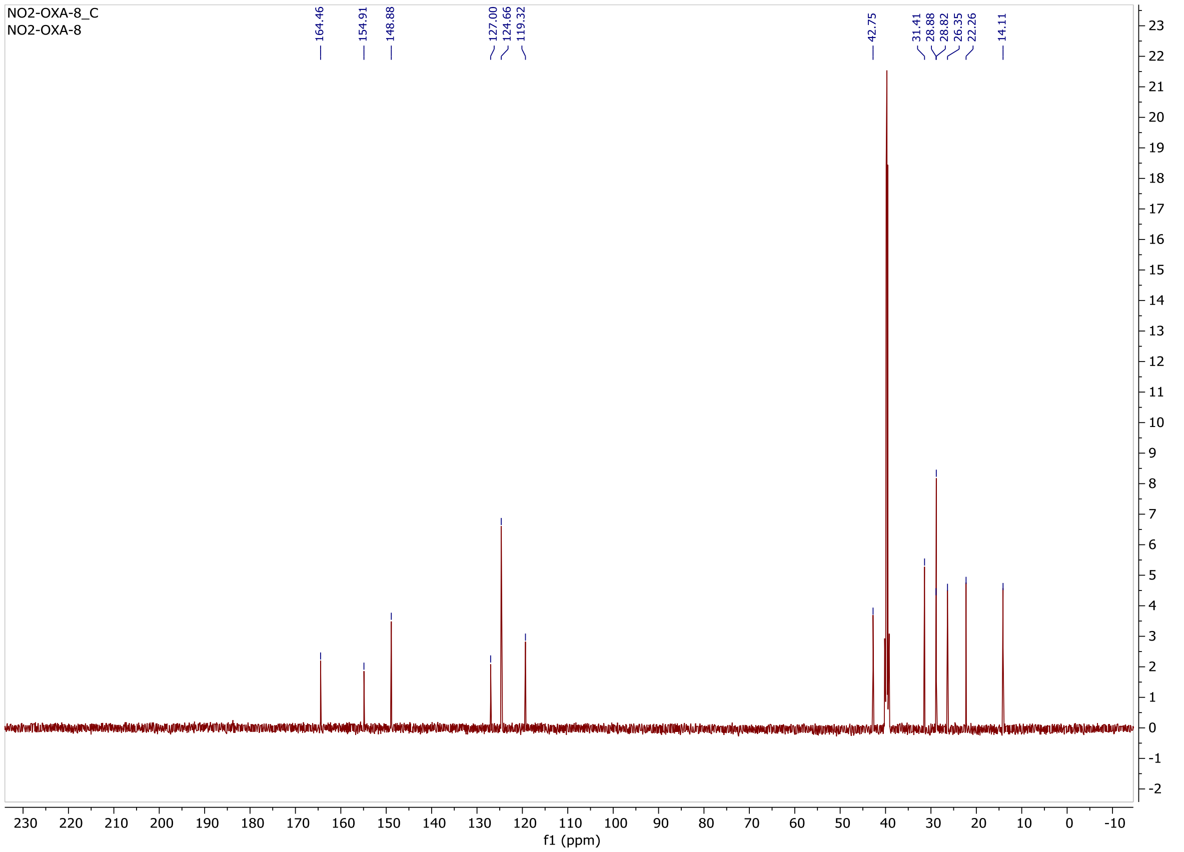

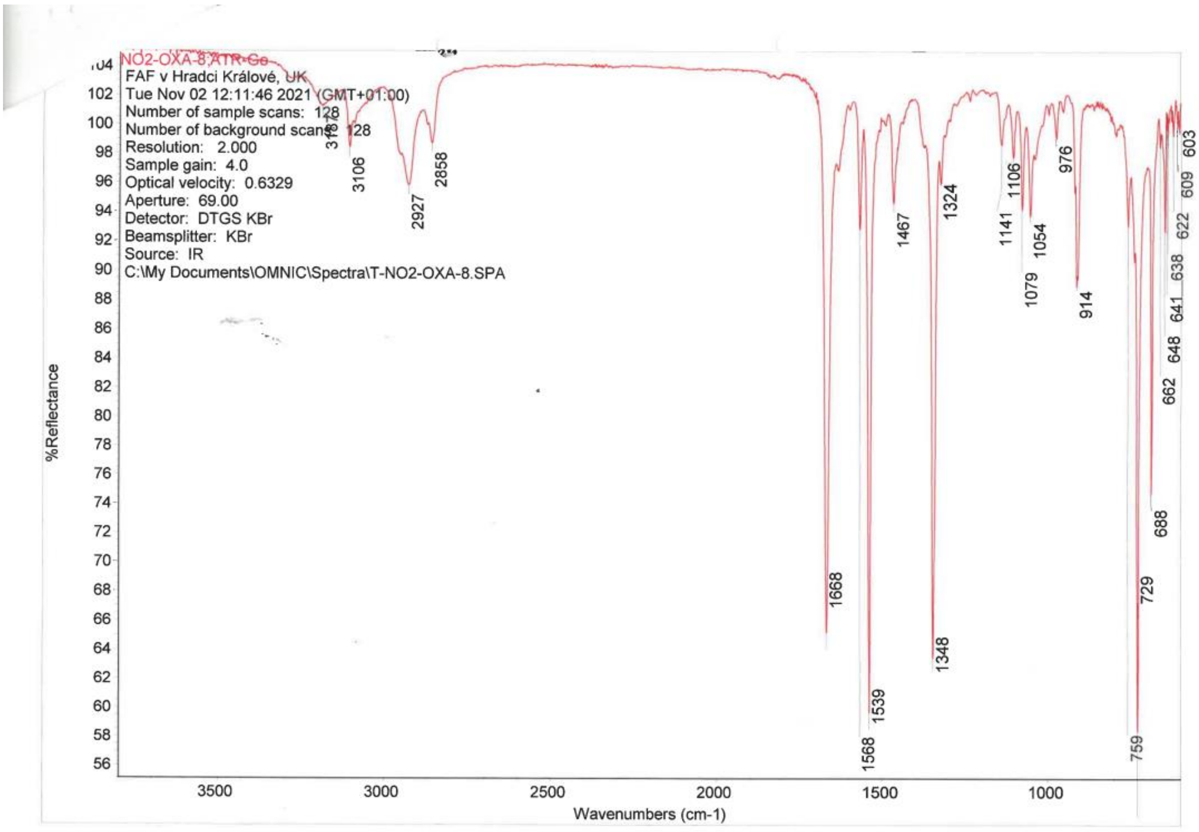

5-(3,5-Dinitrophenyl)-*N*-nonyl-1,3,4-oxadiazol-2-amine **5i**

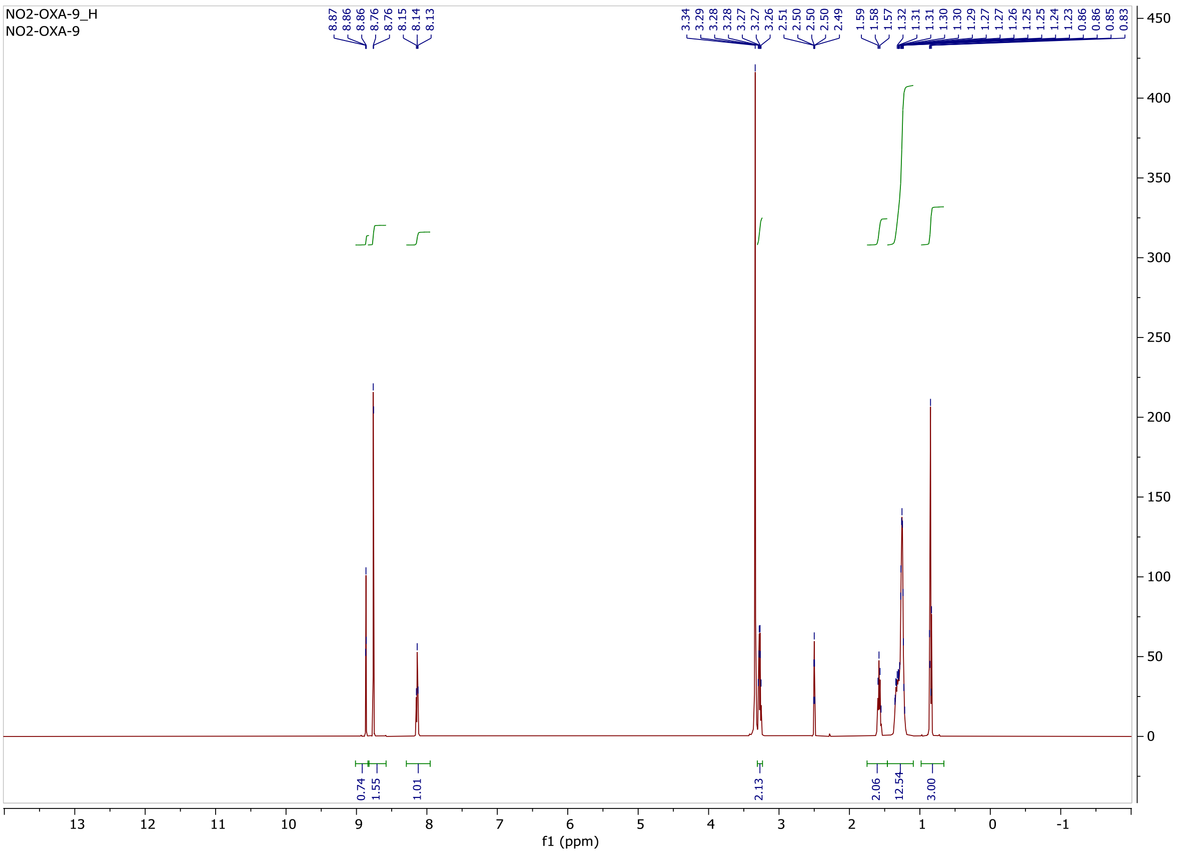

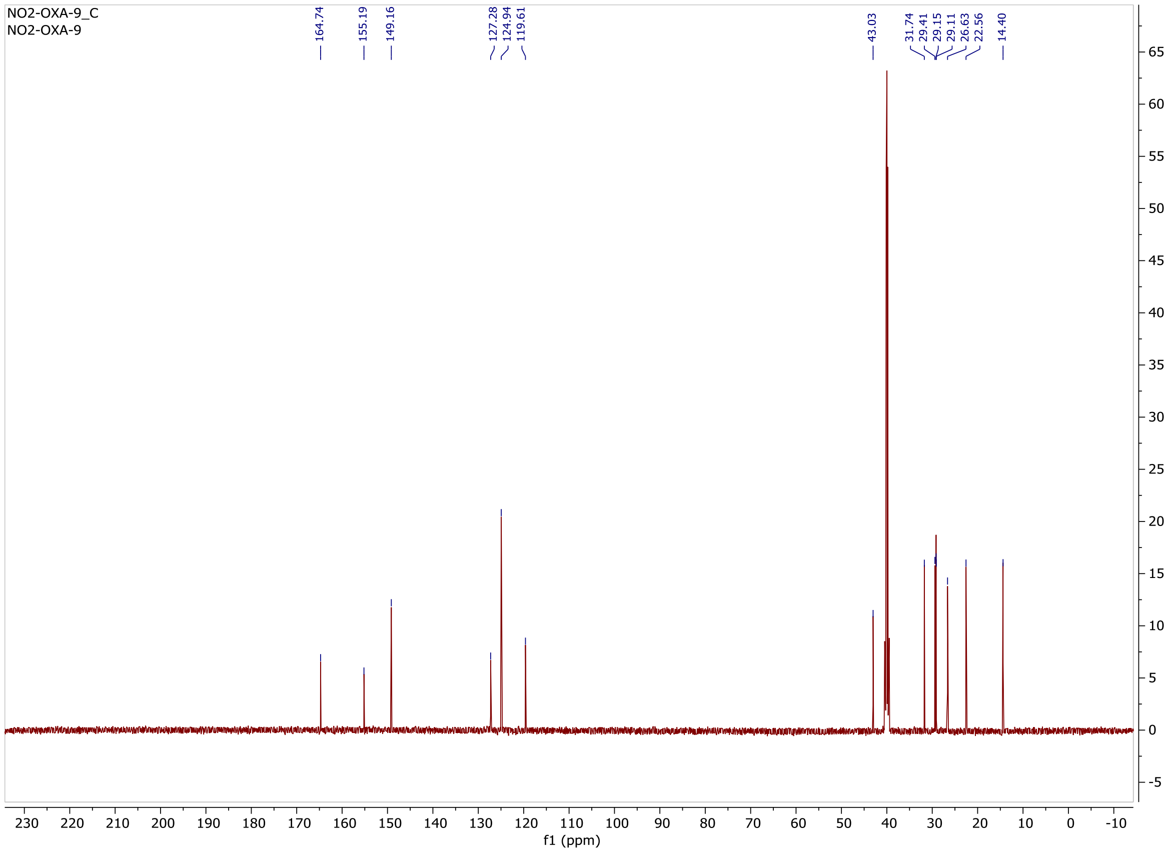
NO2-OXA-9_C
NO2-OXA-9
164.74
155.19
149.16
127.28
124.94
119.61
43.03
31.74
29.41
29.15
29.11
26.63
22.56
14.40
f1 (ppm)

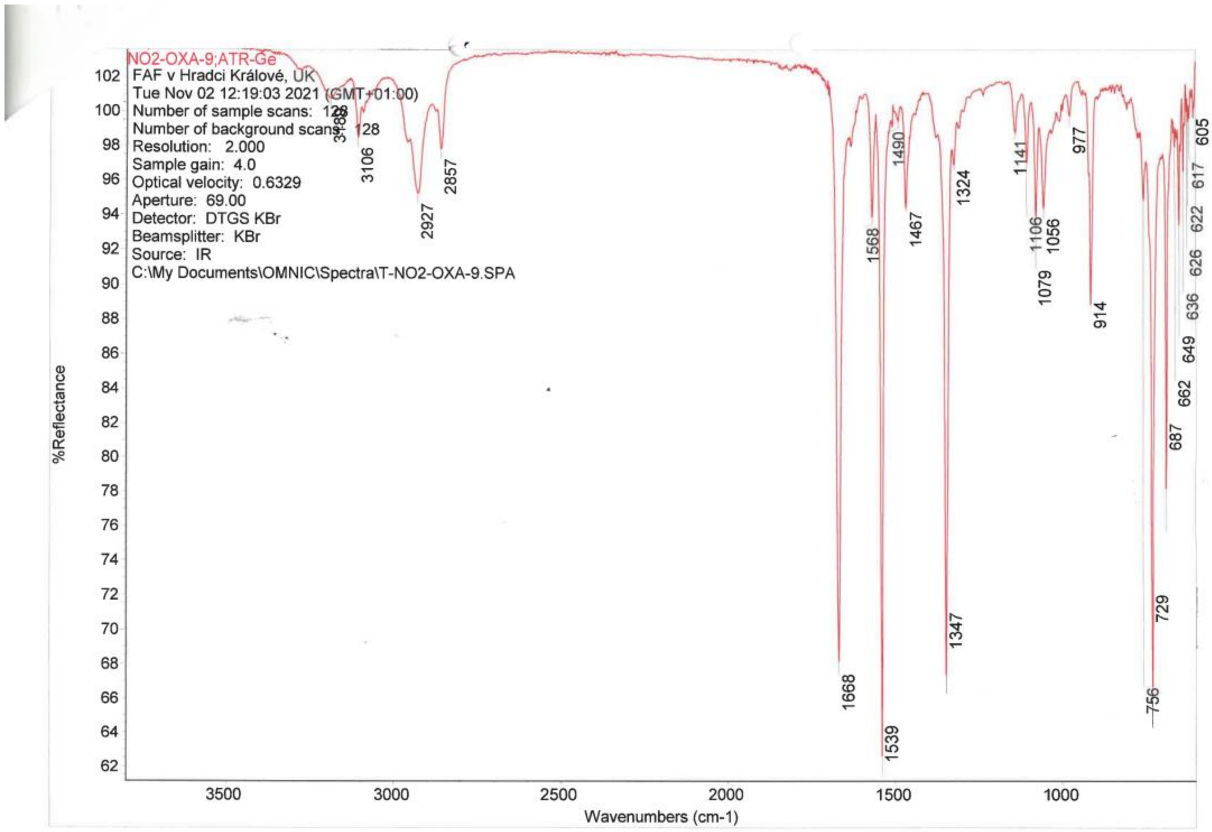
NO2-OXA-9;ATR-Ge
FAF v Hradci Králové, UK
Tue Nov 02 12:19:03 2021 (GMT+01:00)
Number of sample scans: 128
Number of background scans: 128
Resolution: 2.000
Sample gain: 4.0
Optical velocity: 0.6329
Aperture: 69.00
Detector: DTGS KBr
Beamsplitter: KBr
Source: IR
C:\My Documents\OMNIC\Spectra\T-NO2-OXA-9.SPA
%Reflectance
Wavenumbers (cm-1)
3106
2927
2857
1668
1568
1539
1490
1467
1347
1324
1141
1106
1079
1056
977
914
756
729
687
662
649
636
626
622
617
605

N-Decyl-5-(3,5-dinitrophenyl)-1,3,4-oxadiazol-2-amine **5j**

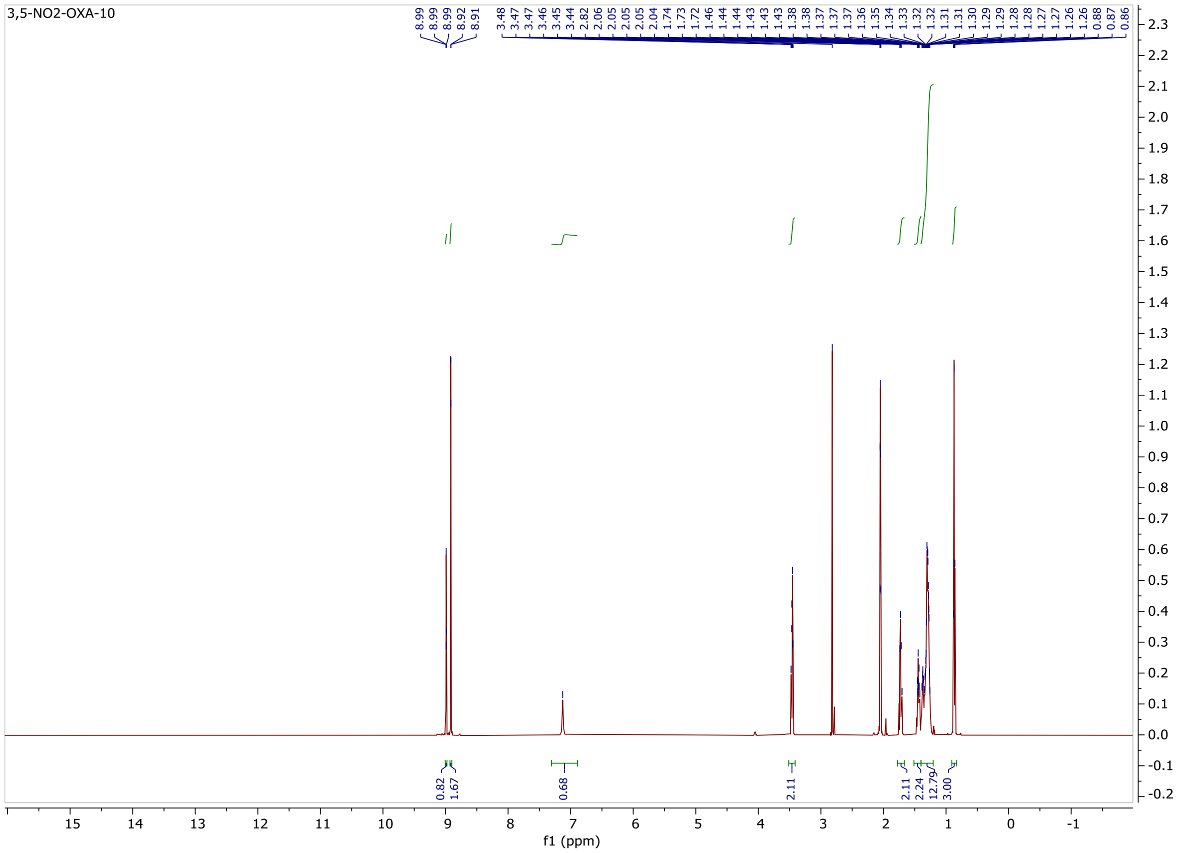

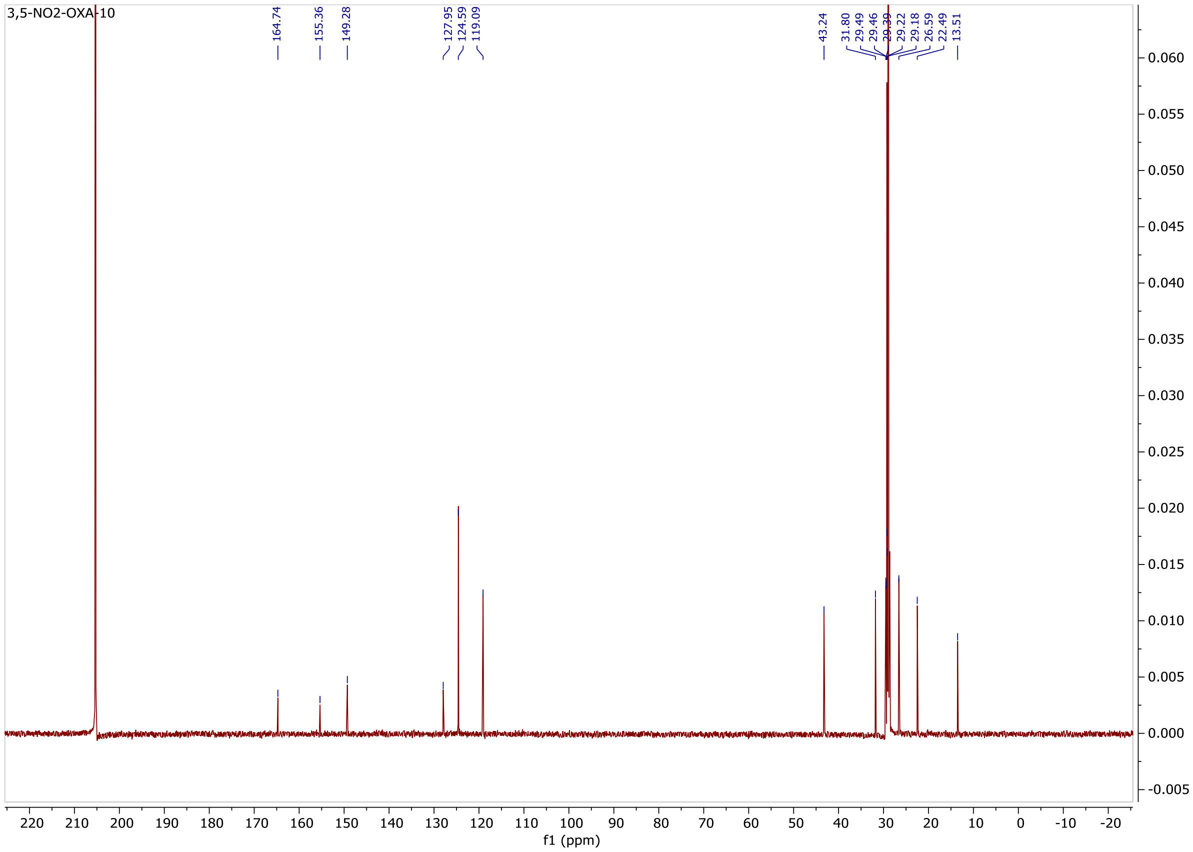

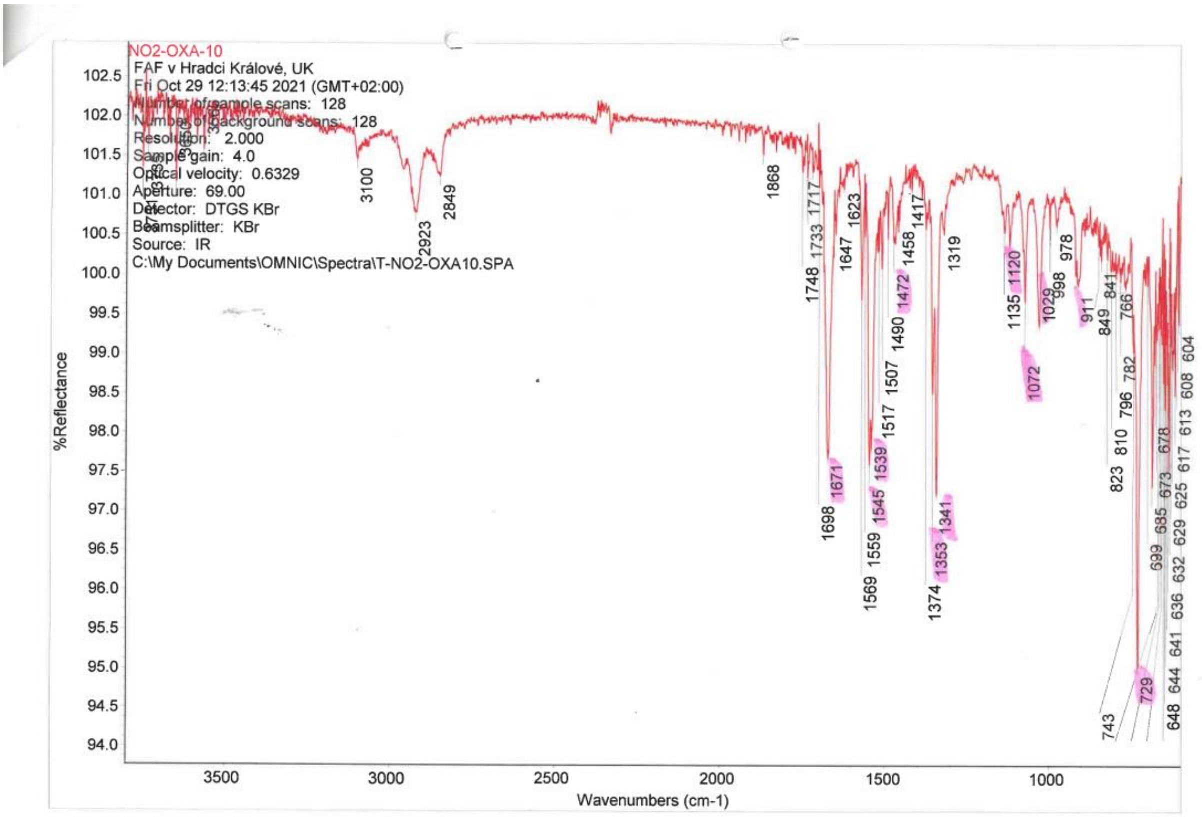

5-(3,5-Dinitrophenyl)-*N*-undecyl-1,3,4-oxadiazol-2-amine **5k**

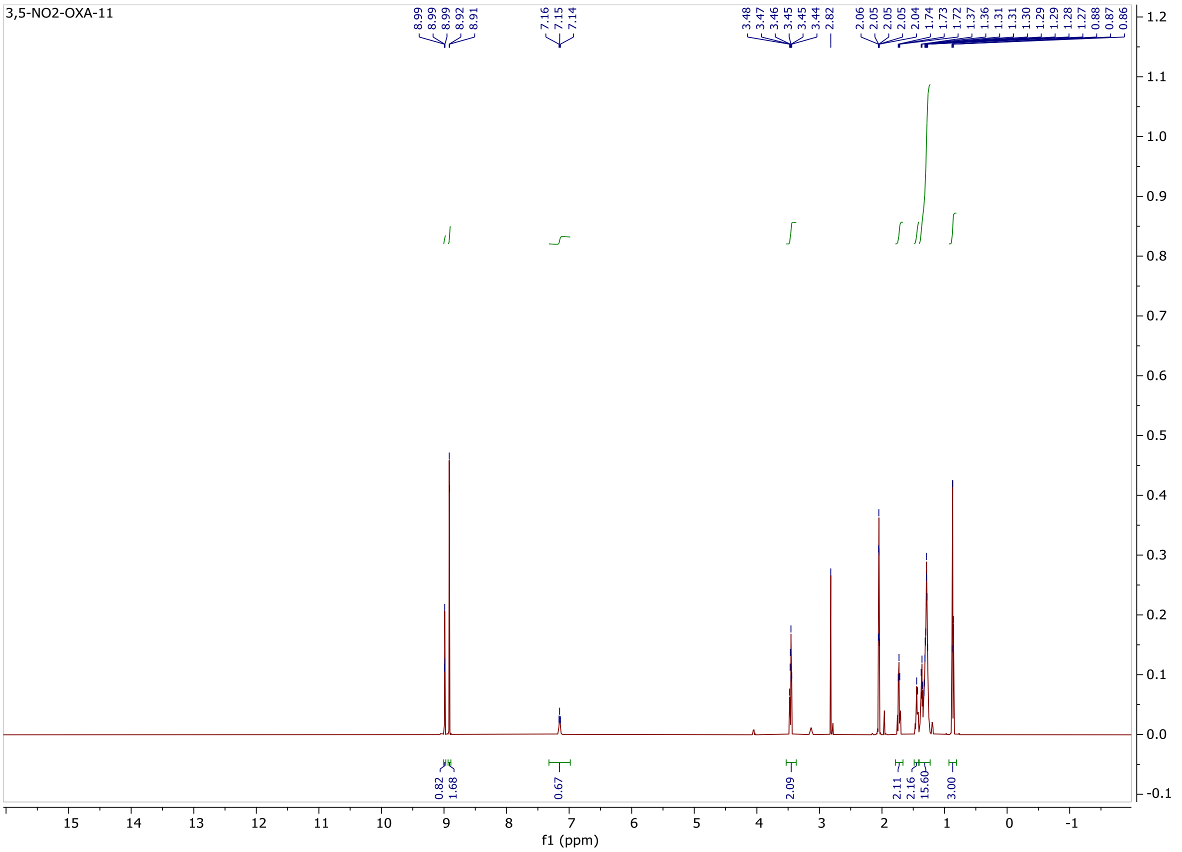

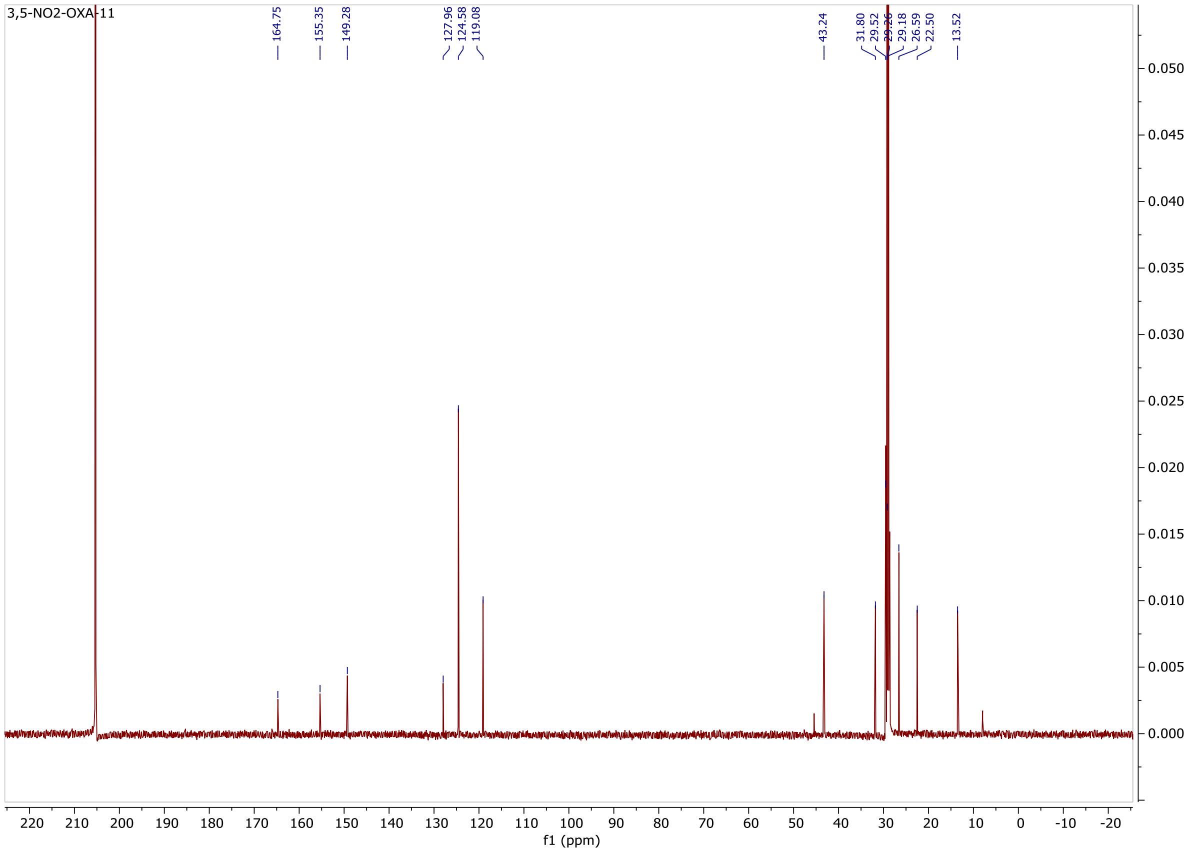
3,5-NO2-OXA-11
164.75
155.35
149.28
127.96
124.58
119.08
43.24
31.80
29.52
29.26
29.18
26.59
22.50
13.52
f1 (ppm)

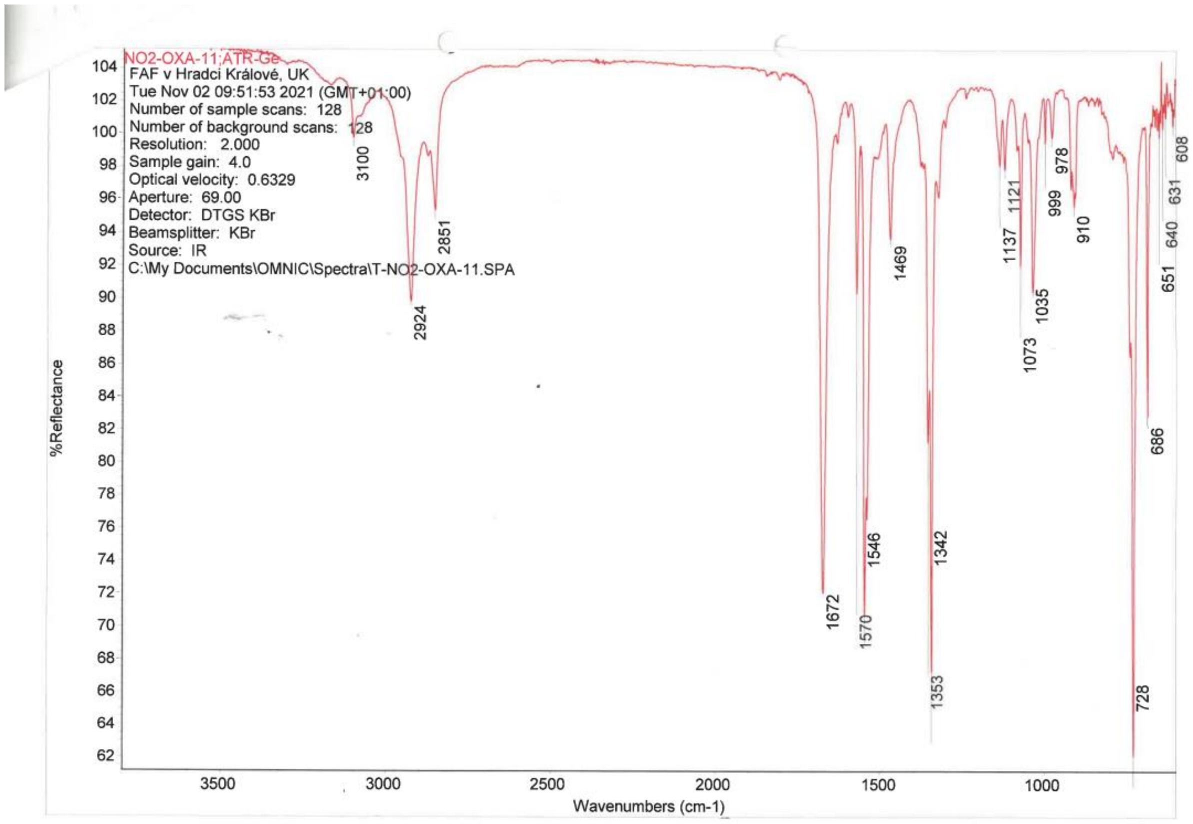
NO2-OXA-11;ATR-Ge
FAF v Hradci Králové, UK
Tue Nov 02 09:51:53 2021 (GMT+01:00)
Number of sample scans: 128
Number of background scans: 128
Resolution: 2.000
Sample gain: 4.0
Optical velocity: 0.6329
Aperture: 69.00
Detector: DTGS KBr
Beamsplitter: KBr
Source: IR
C:\My Documents\OMNIC\Spectra\T-NO2-OXA-11.SPA
%Reflectance
Wavenumbers (cm-1)
3100
2924
2851
1672
1570
1546
1469
1353
1342
1137
1121
1073
1035
999
978
910
728
686
651
640
631
608

5-(3,5-Dinitrophenyl)-*N*-dodecyl-1,3,4-oxadiazol-2-amine **5l**

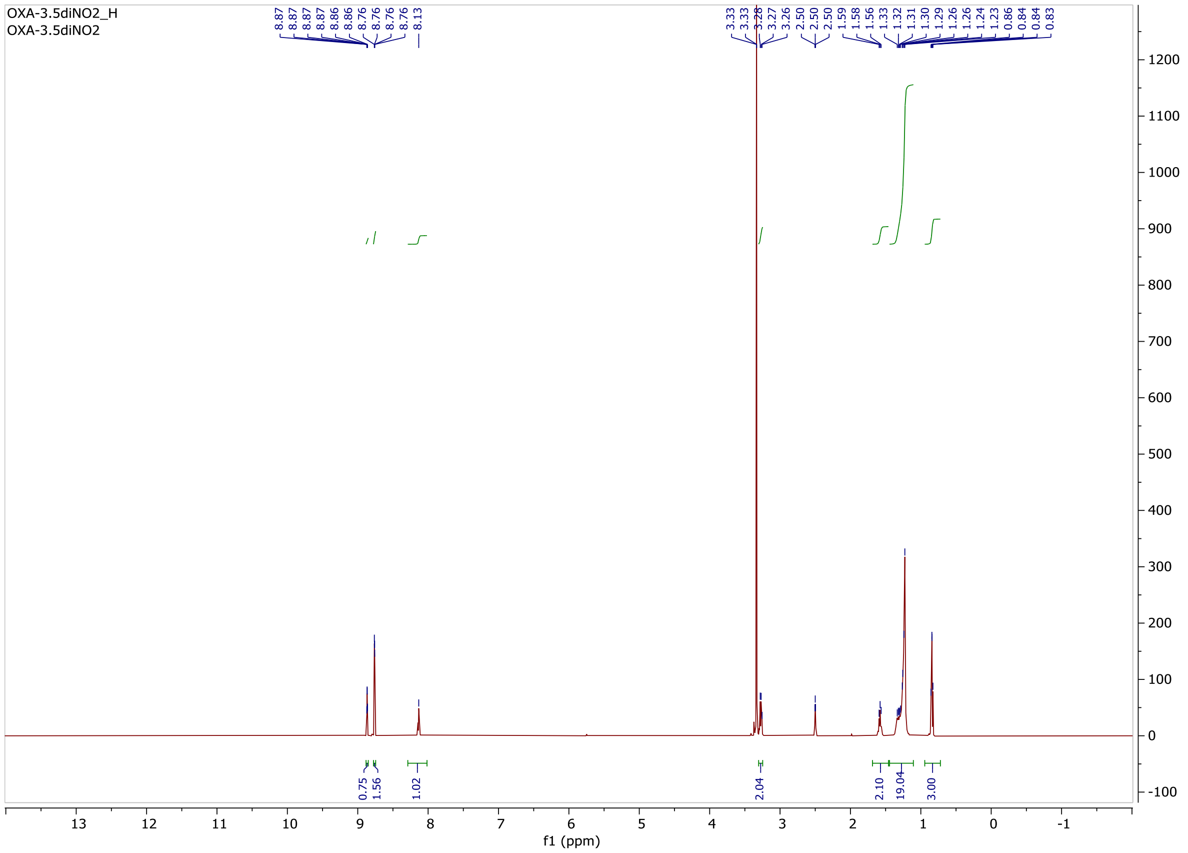

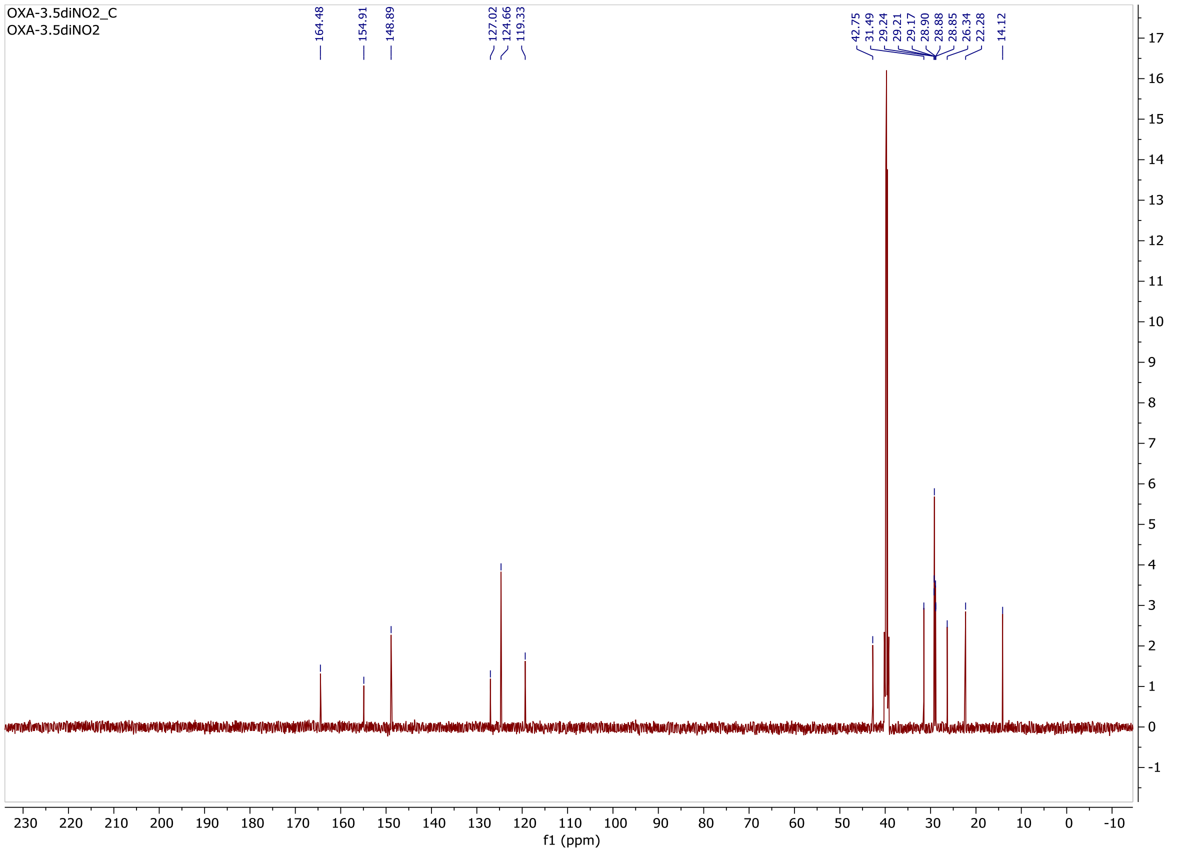

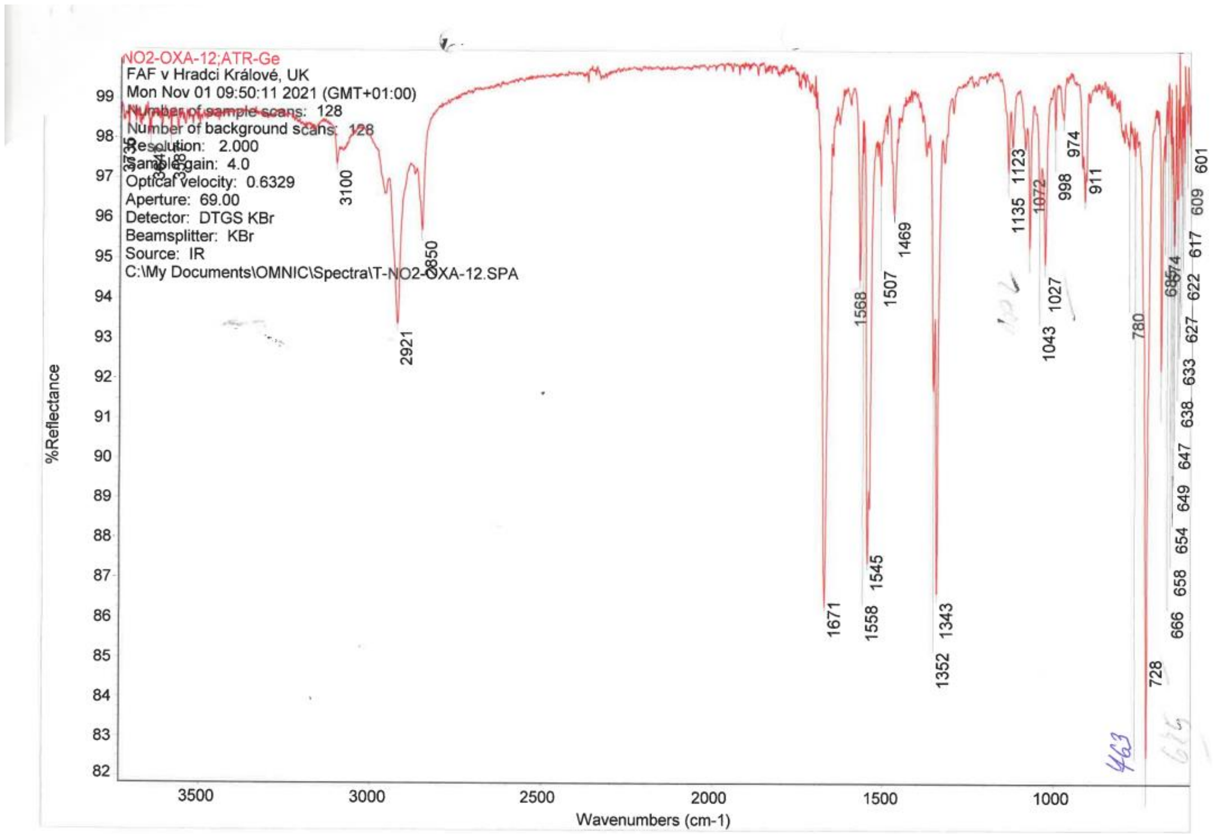

5-(3,5-Dinitrophenyl)-*N*-tridecyl-1,3,4-oxadiazol-2-amine **5m**

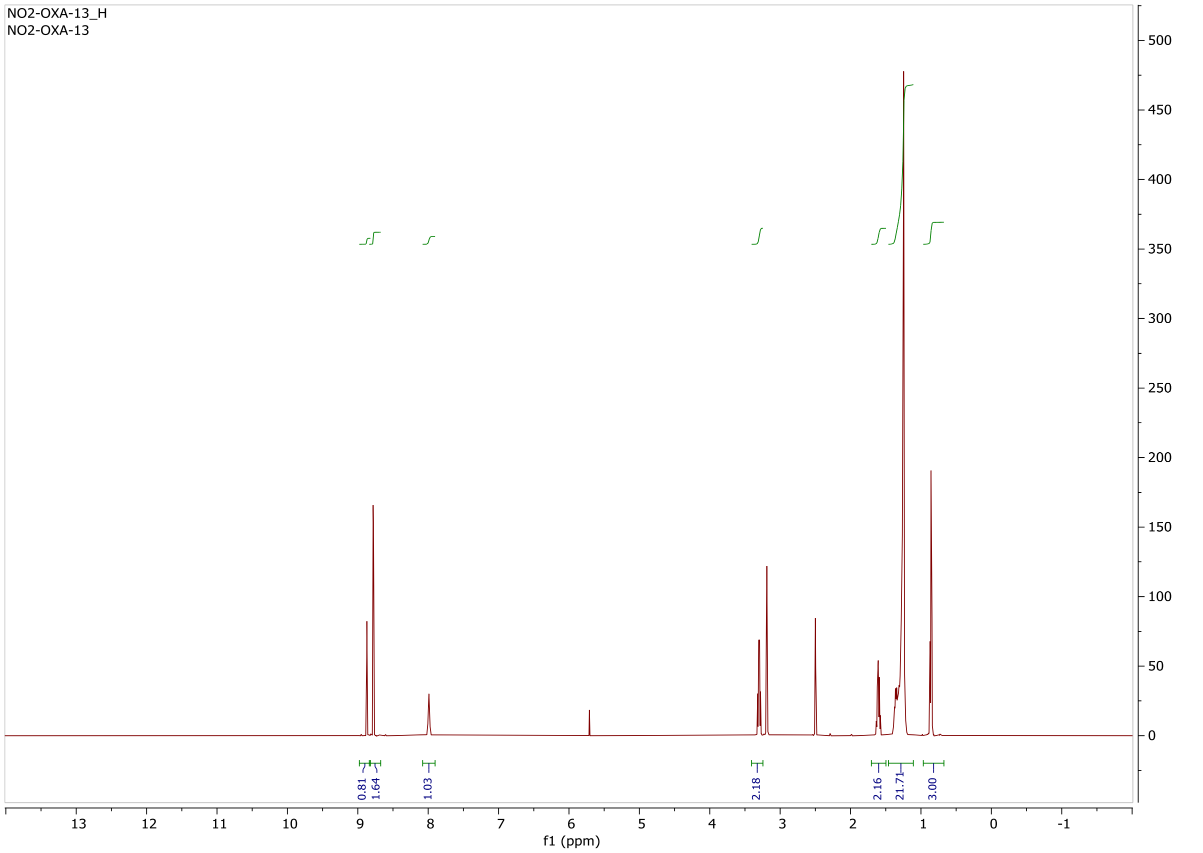

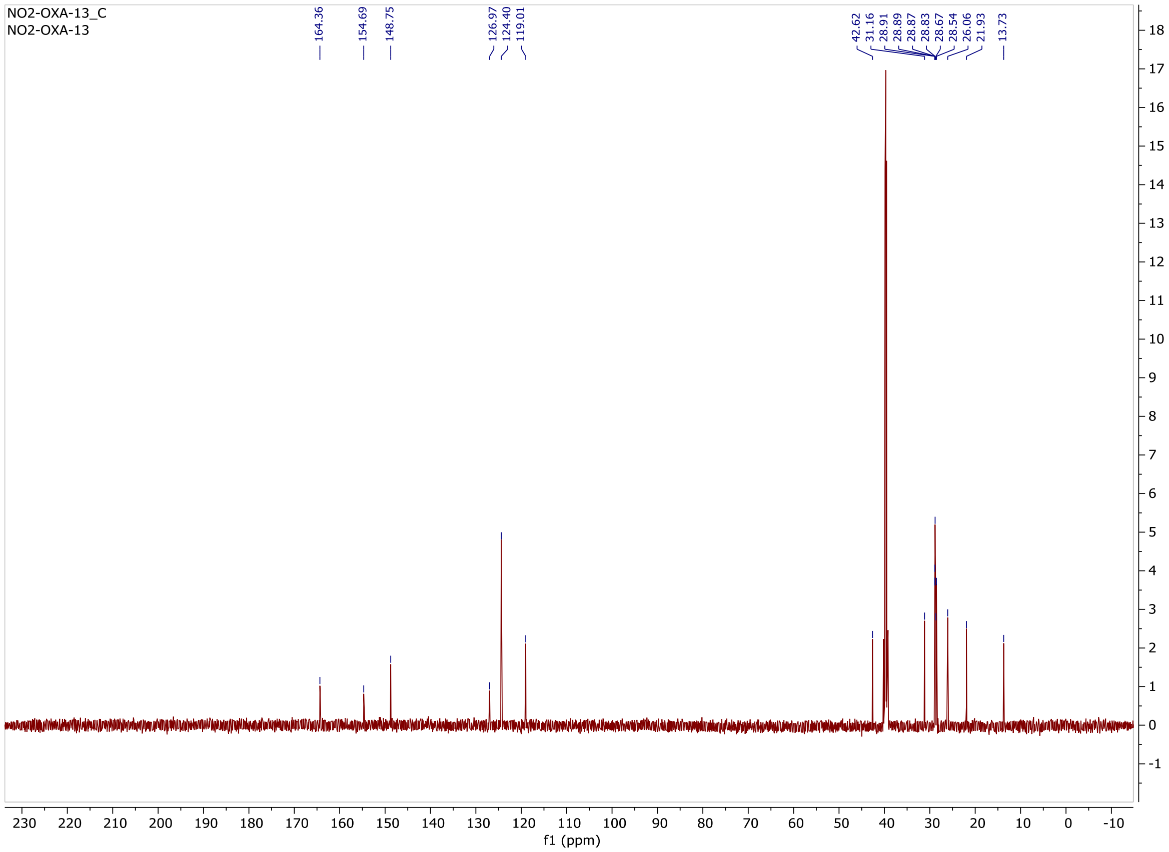
NO2-OXA-13_C
NO2-OXA-13
164.36
154.69
148.75
126.97
124.40
119.01
42.62
31.16
28.91
28.89
28.87
28.83
28.67
28.54
26.06
21.93
13.73
f1 (ppm)

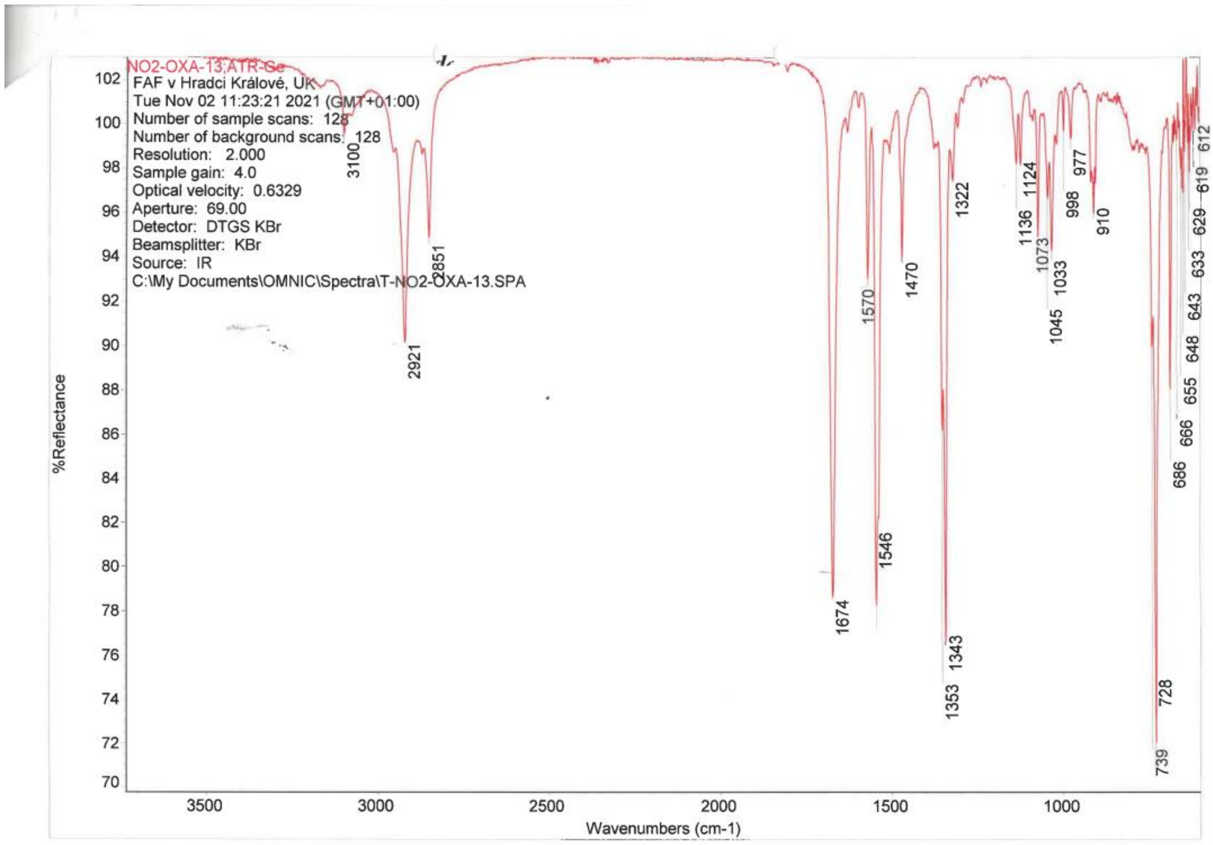
NO2-OXA-13;ATR-Ge
FAF v Hradci Králové, UK
Tue Nov 02 11:23:21 2021 (GMT+01:00)
Number of sample scans: 128
Number of background scans: 128
Resolution: 2.000
Sample gain: 4.0
Optical velocity: 0.6329
Aperture: 69.00
Detector: DTGS KBr
Beamsplitter: KBr
Source: IR
C:\My Documents\OMNIC\Spectra\T-NO2-OXA-13.SPA
%Reflectance
Wavenumbers (cm-1)
3100
2921
2851
1674
1570
1546
1470
1353
1343
1322
1136
1124
1073
1045
1033
998
977
910
739
728
686
666
655
648
643
633
629
619
612

5-(3,5-Dinitrophenyl)-*N*-tetradecyl-1,3,4-oxadiazol-2-amine **5n**

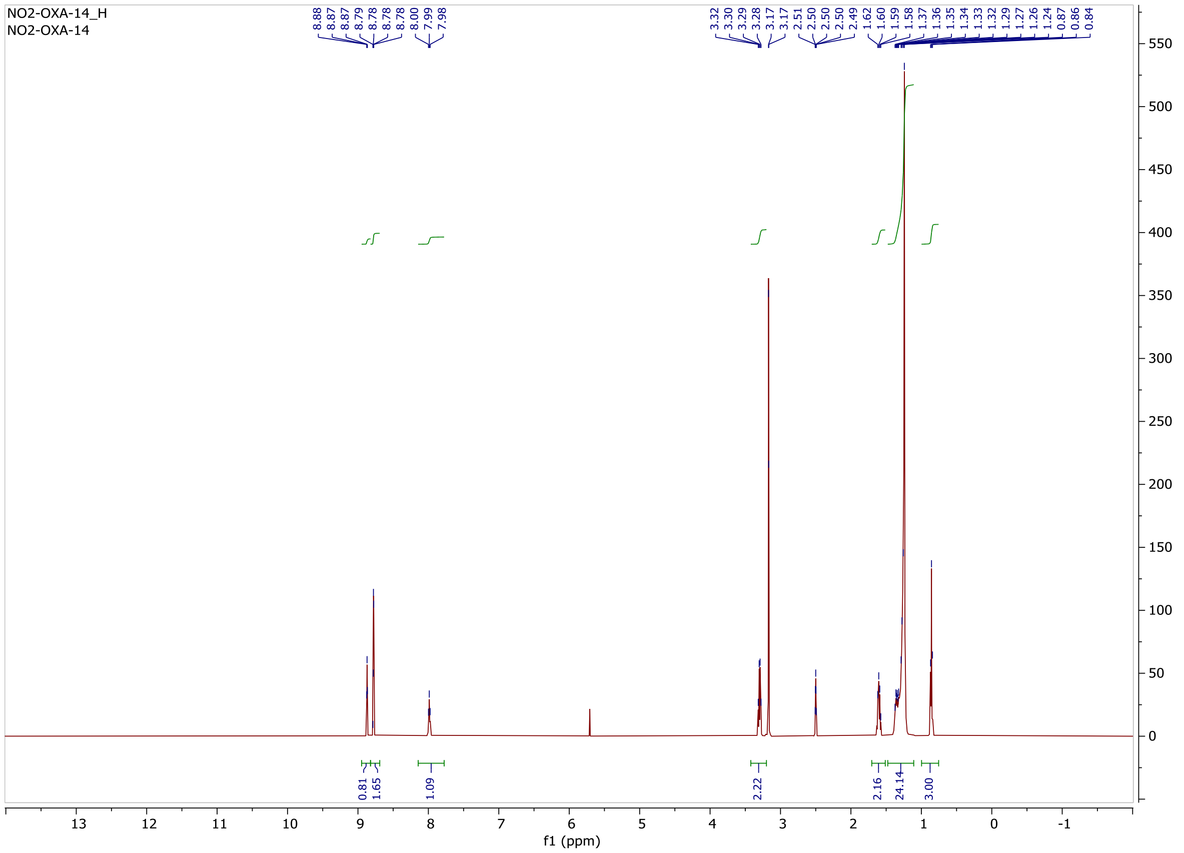

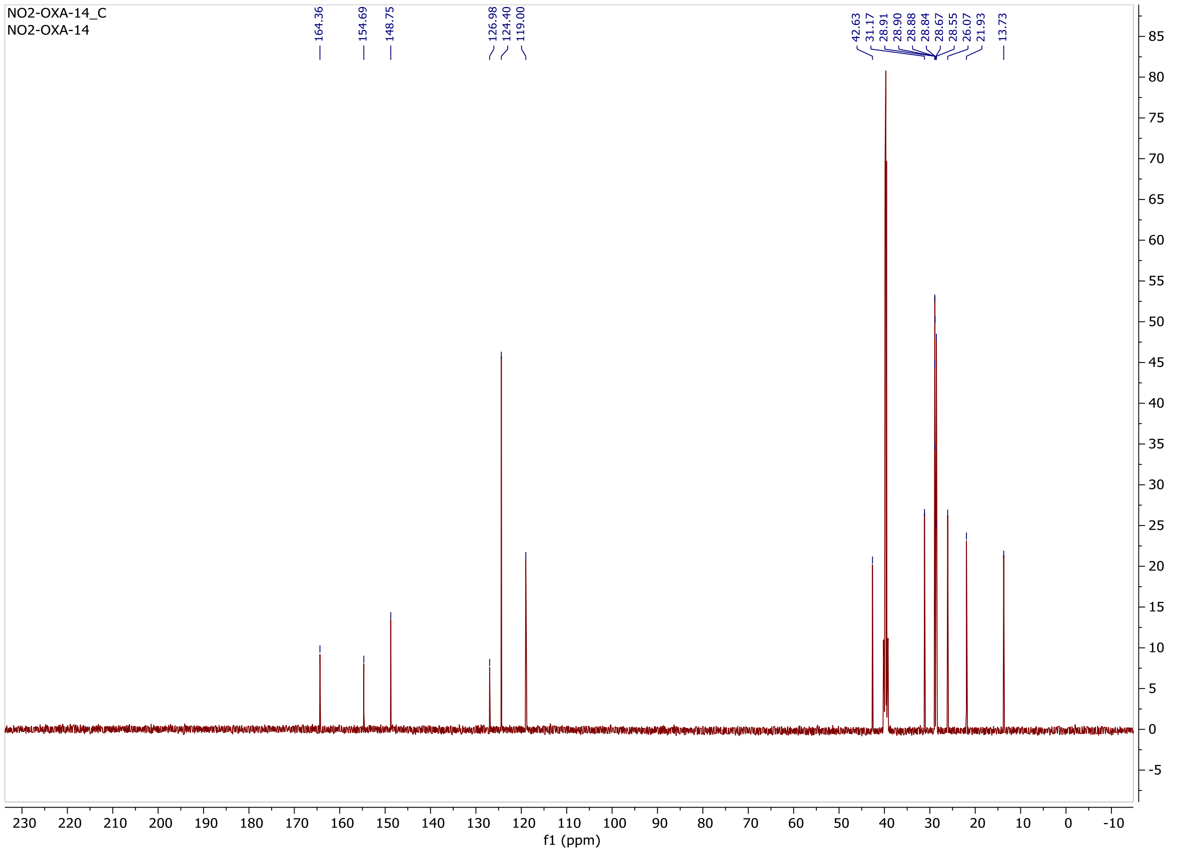

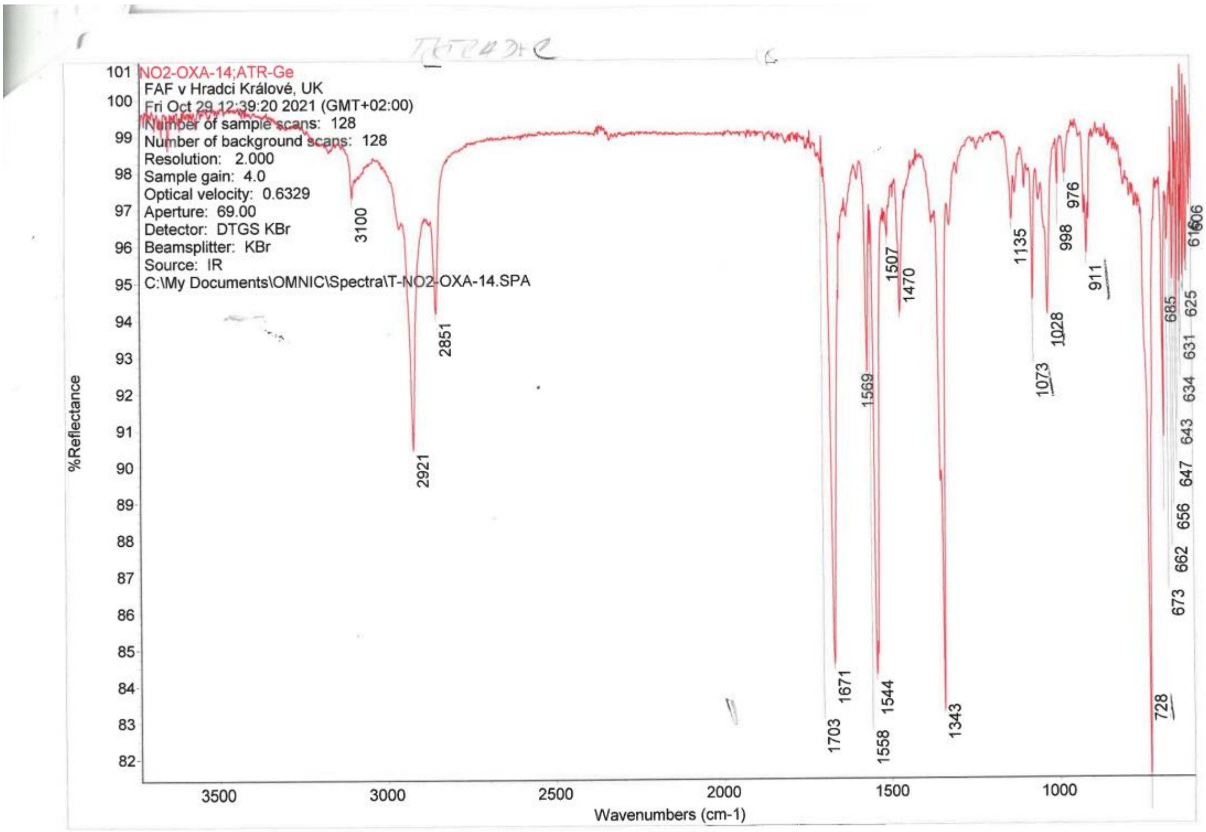

5-(3,5-Dinitrophenyl)-*N*-pentadecyl-1,3,4-oxadiazol-2-amine **5o**

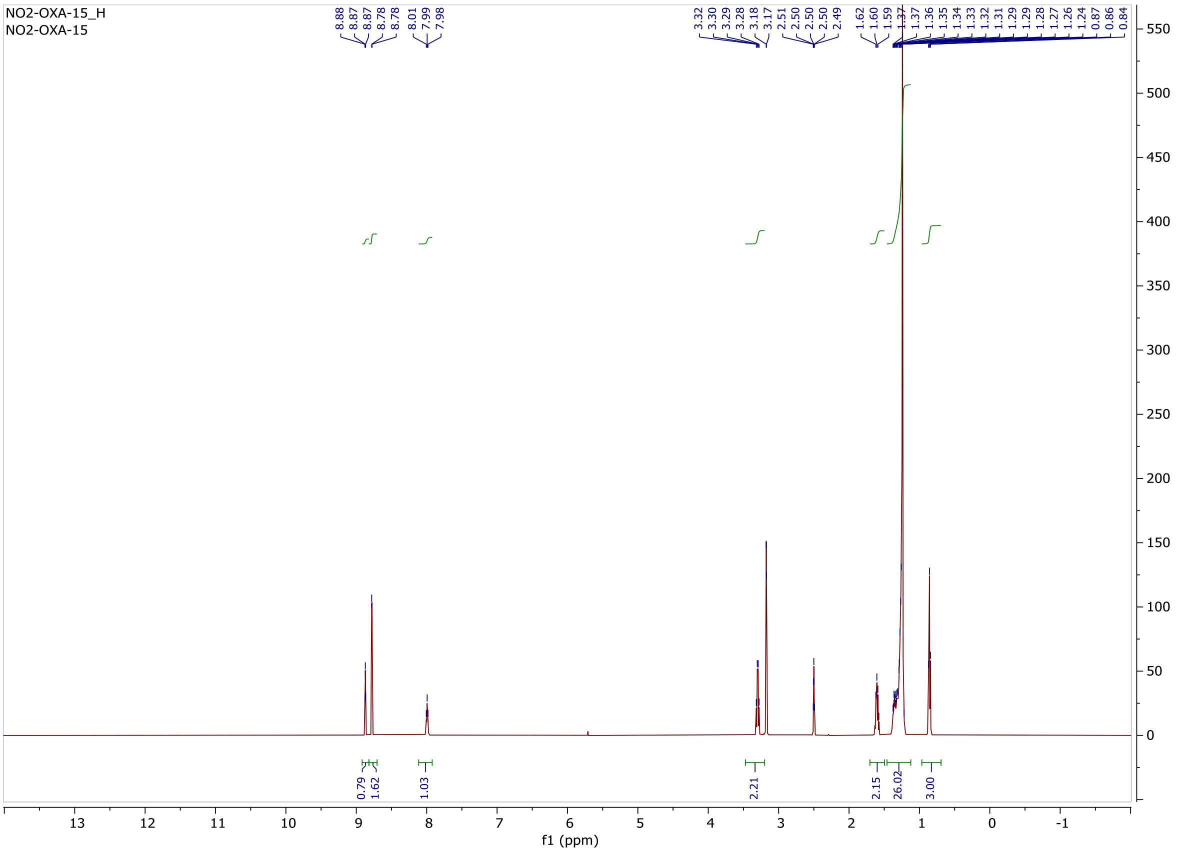

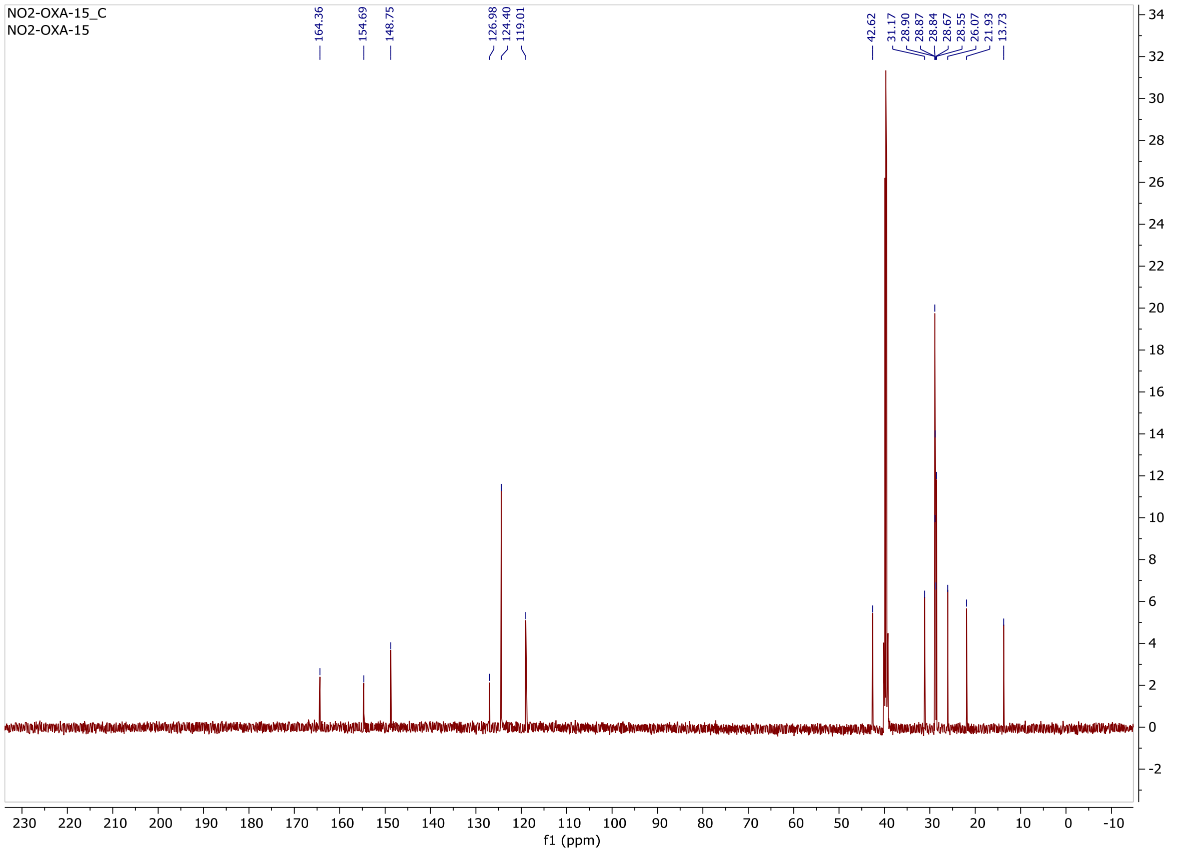
NO2-OXA-15_C
NO2-OXA-15
164.36
154.69
148.75
126.98
124.40
119.01
42.62
31.17
28.90
28.87
28.84
28.67
28.55
26.07
21.93
13.73
f1 (ppm)

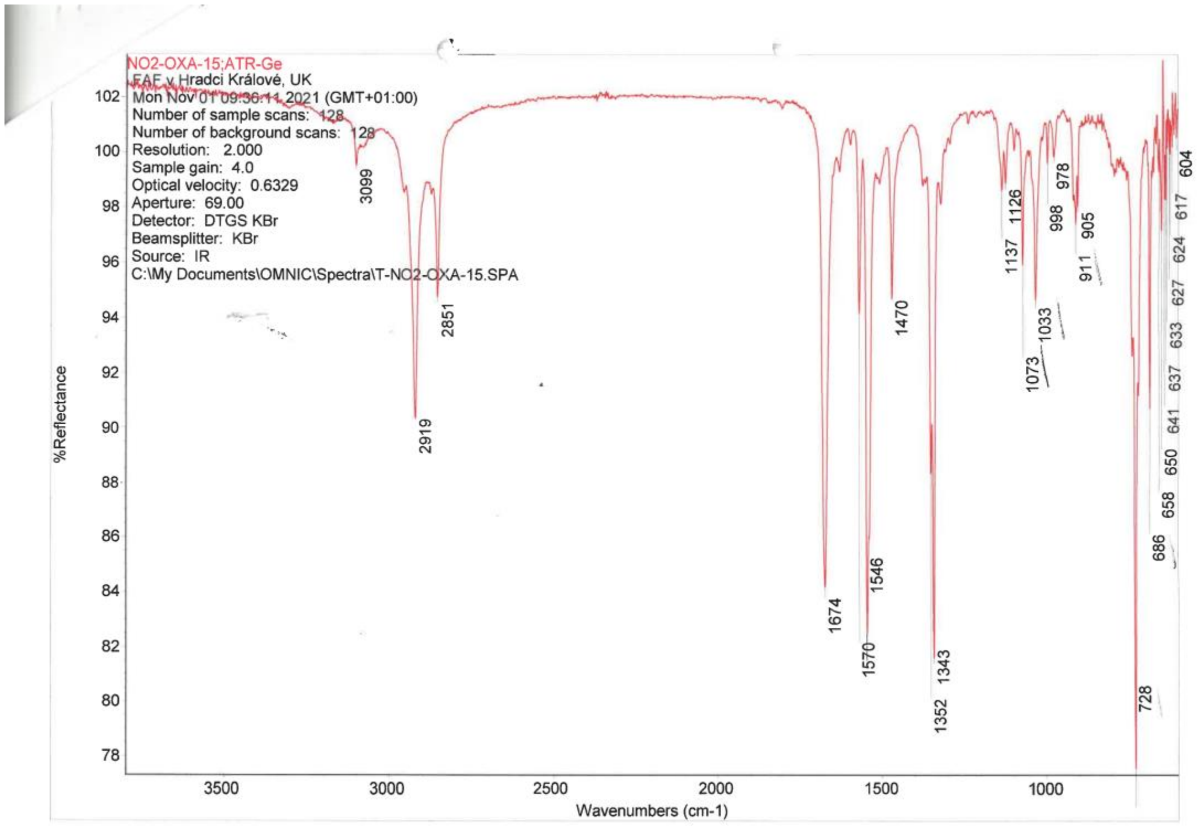
NO2-OXA-15;ATR-Ge
FAF v Hradci Králové, UK
Mon Nov 01 09:36:11 2021 (GMT+01:00)
Number of sample scans: 128
Number of background scans: 128
Resolution: 2.000
Sample gain: 4.0
Optical velocity: 0.6329
Aperture: 69.00
Detector: DTGS KBr
Beamsplitter: KBr
Source: IR
C:\My Documents\OMNIC\Spectra\T-NO2-OXA-15.SPA
%Reflectance
Wavenumbers (cm-1)
3099
2919
2851
1674
1570
1546
1470
1352
1343
1137
1126
1073
1033
998
978
911
905
728
686
658
650
641
637
633
627
624
617
604

5-(3,5-Dinitrophenyl)-*N*-hexadecyl-1,3,4-oxadiazol-2-amine **5p**

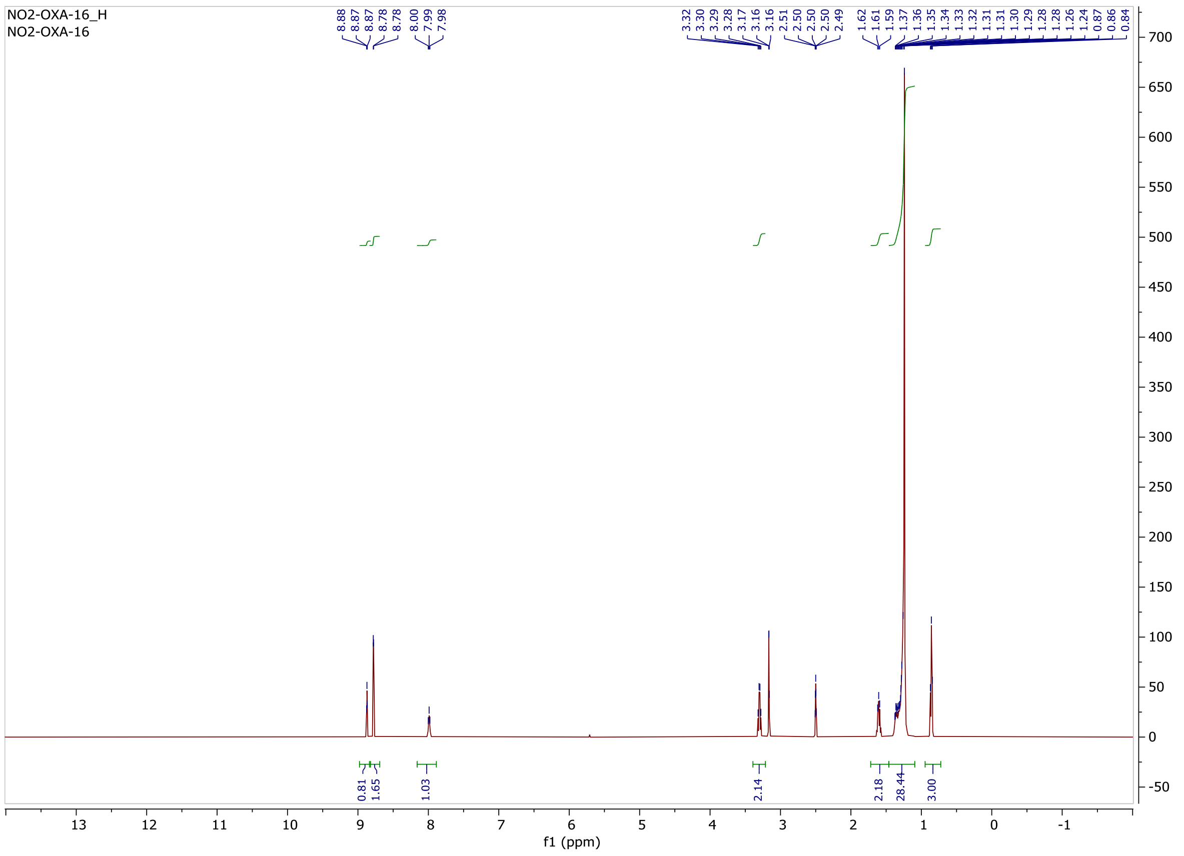

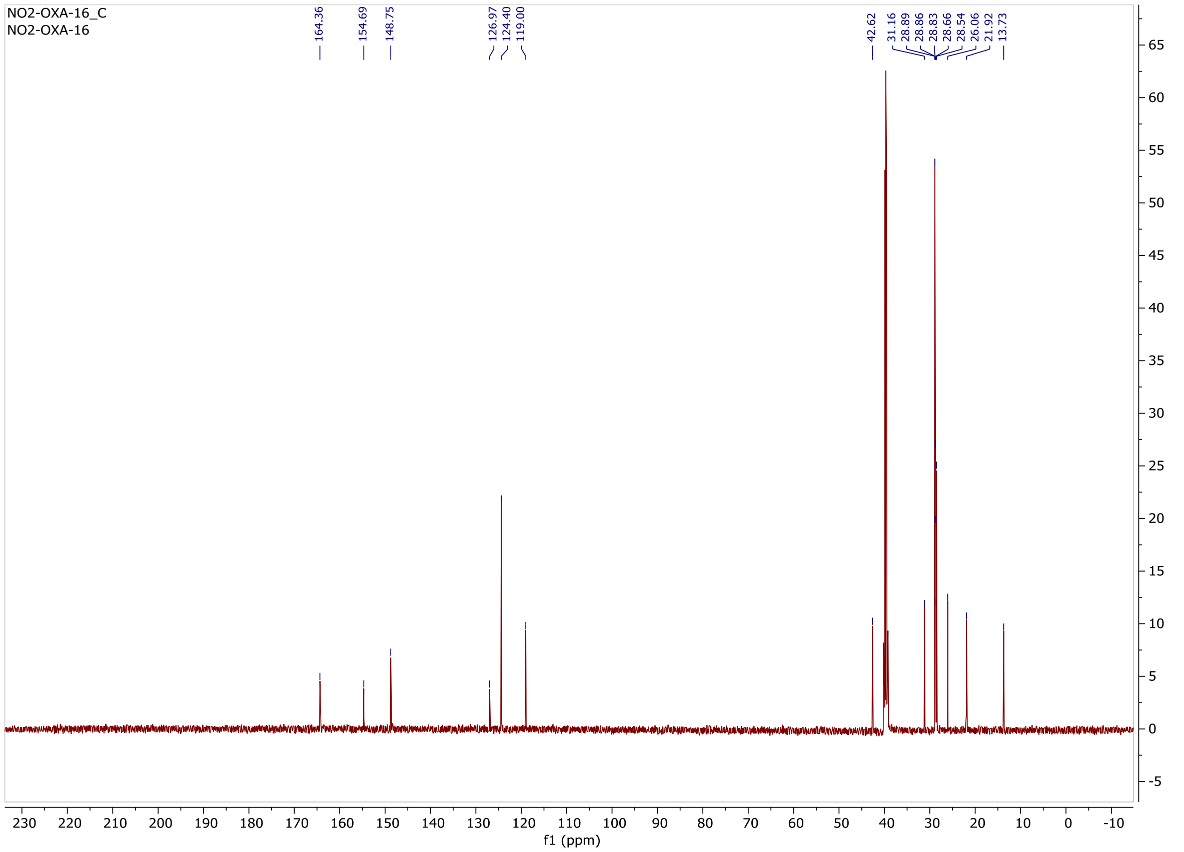

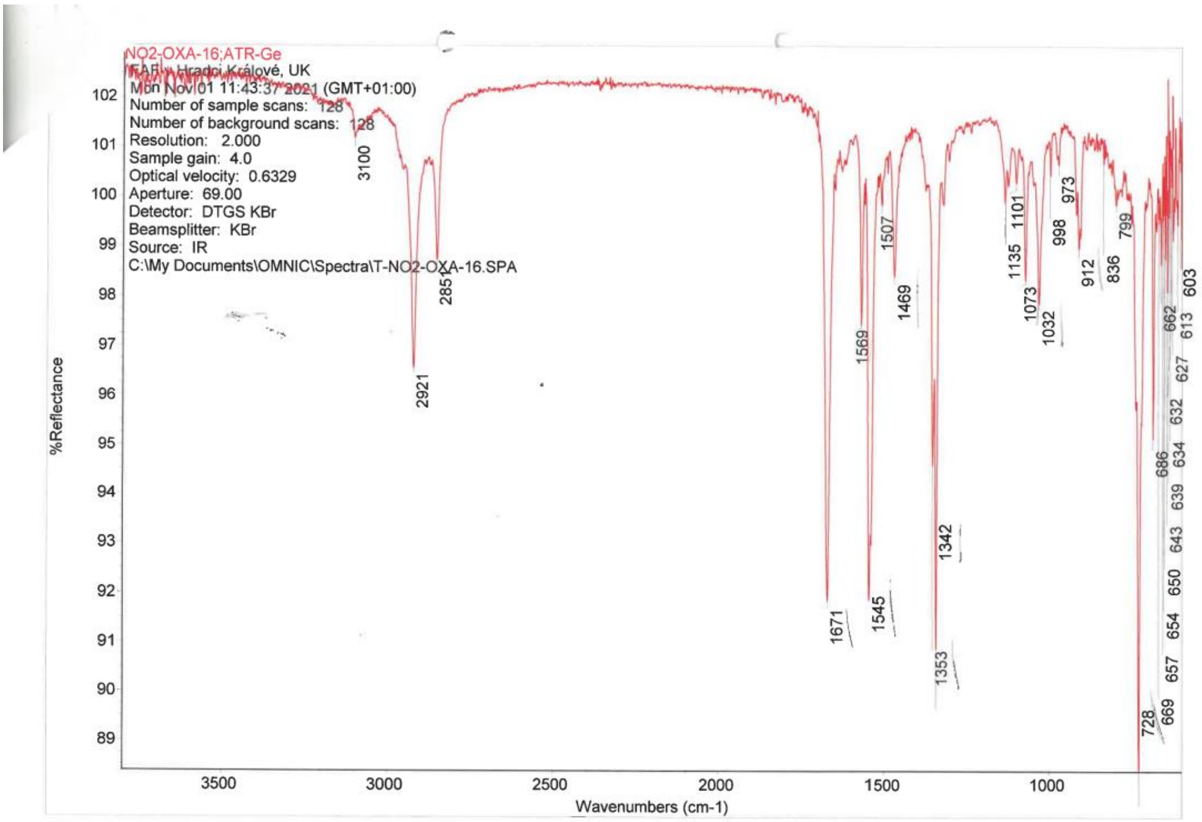

5-(3,5-Dinitrophenyl)-*N*-heptadecyl-1,3,4-oxadiazol-2-amine **5q**

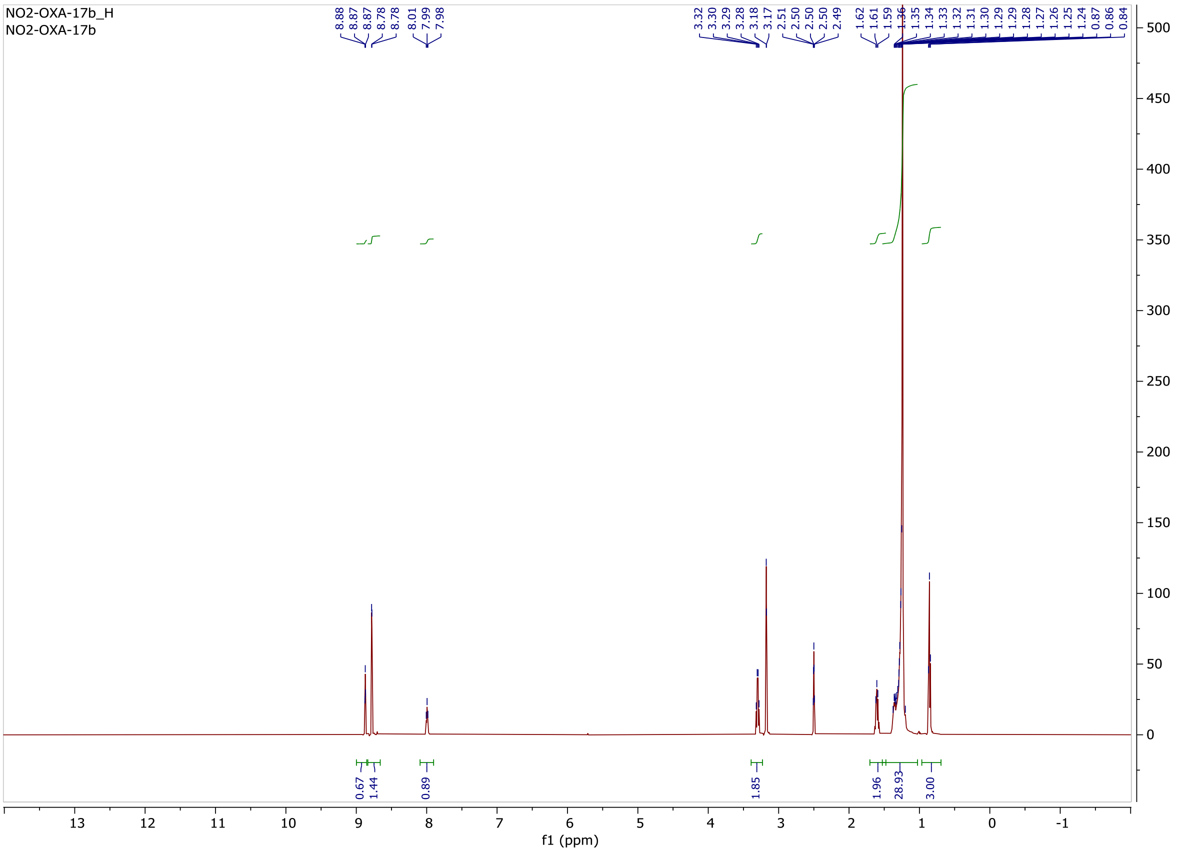

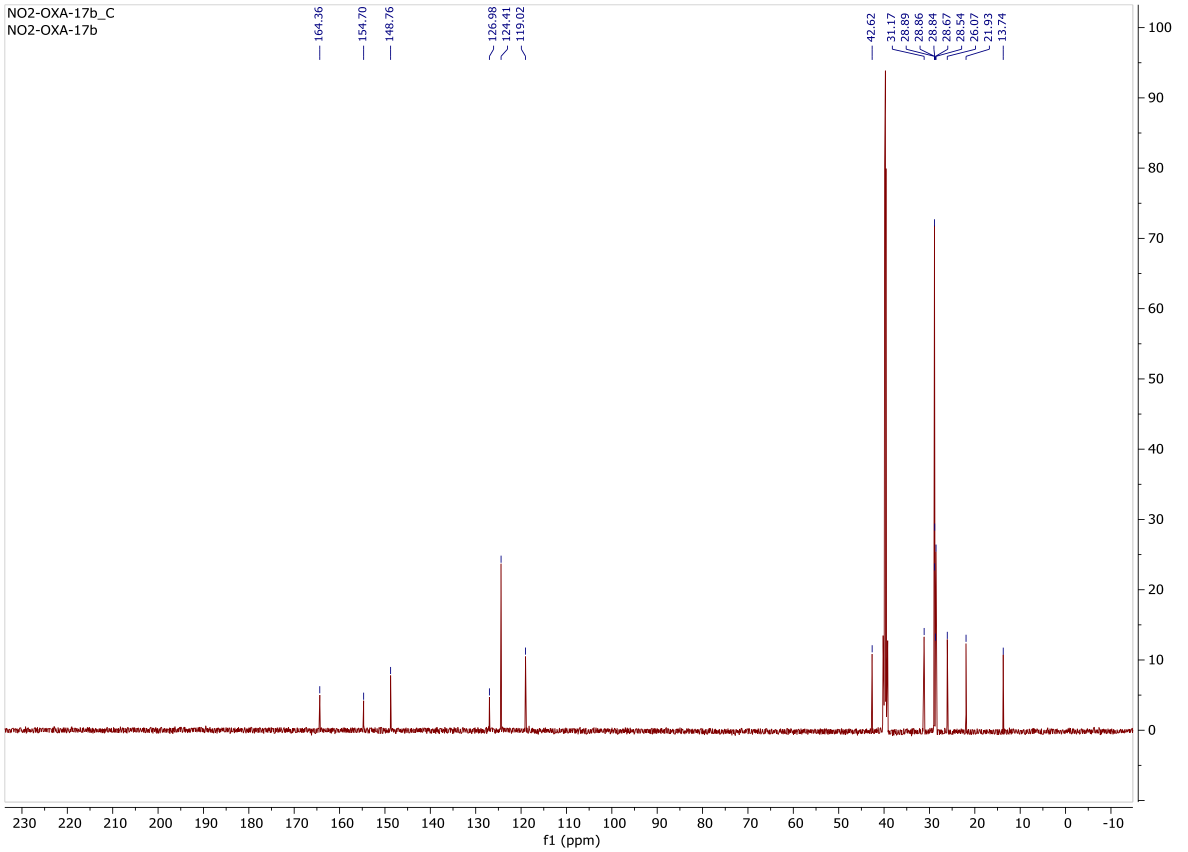
NO2-OXA-17b_C
NO2-OXA-17b
164.36
154.70
148.76
126.98
124.41
119.02
42.62
31.17
28.89
28.86
28.84
28.67
28.54
26.07
21.93
13.74
f1 (ppm)

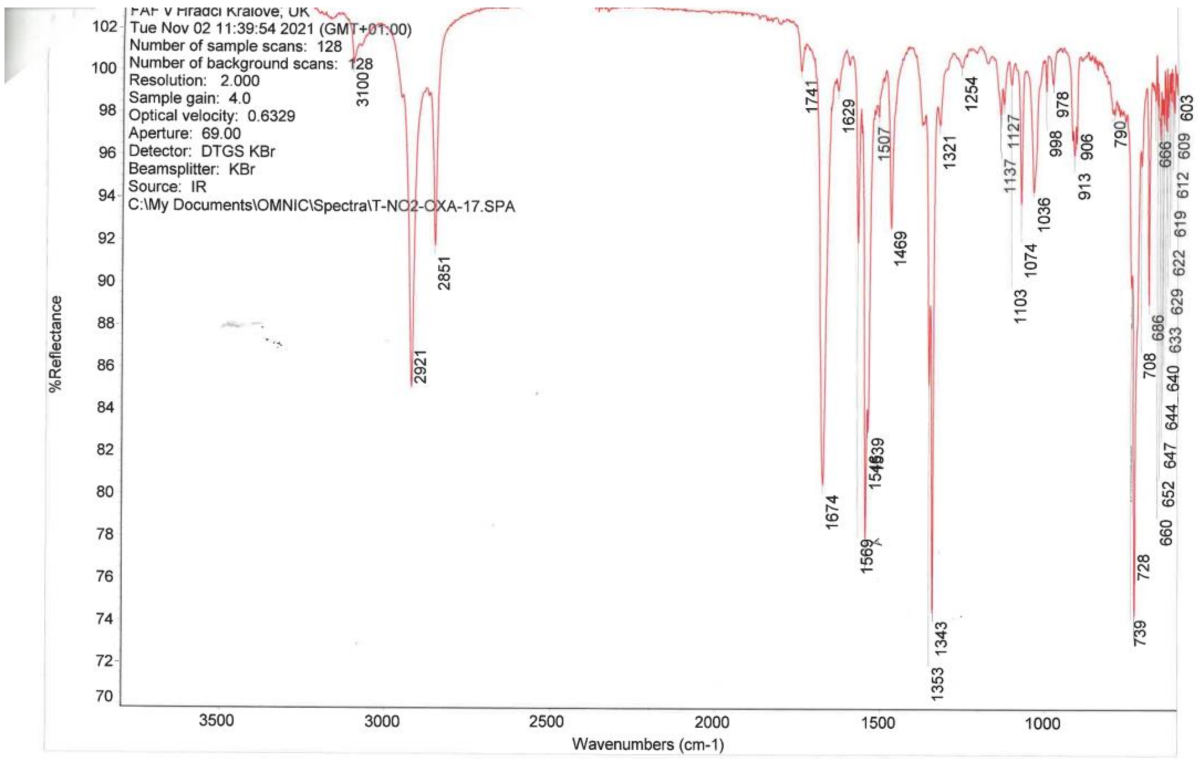
FAF v Hradci Kralove, UK
Tue Nov 02 11:39:54 2021 (GMT+01:00)
Number of sample scans: 128
Number of background scans: 128
Resolution: 2.000
Sample gain: 4.0
Optical velocity: 0.6329
Aperture: 69.00
Detector: DTGS KBr
Beamsplitter: KBr
Source: IR
C:\My Documents\OMNIC\Spectra\T-NO2-OXA-17.SPA
%Reflectance
Wavenumbers (cm-1)

5-(3,5-Dinitrophenyl)-*N*-octadecyl-1,3,4-oxadiazol-2-amine **5r**

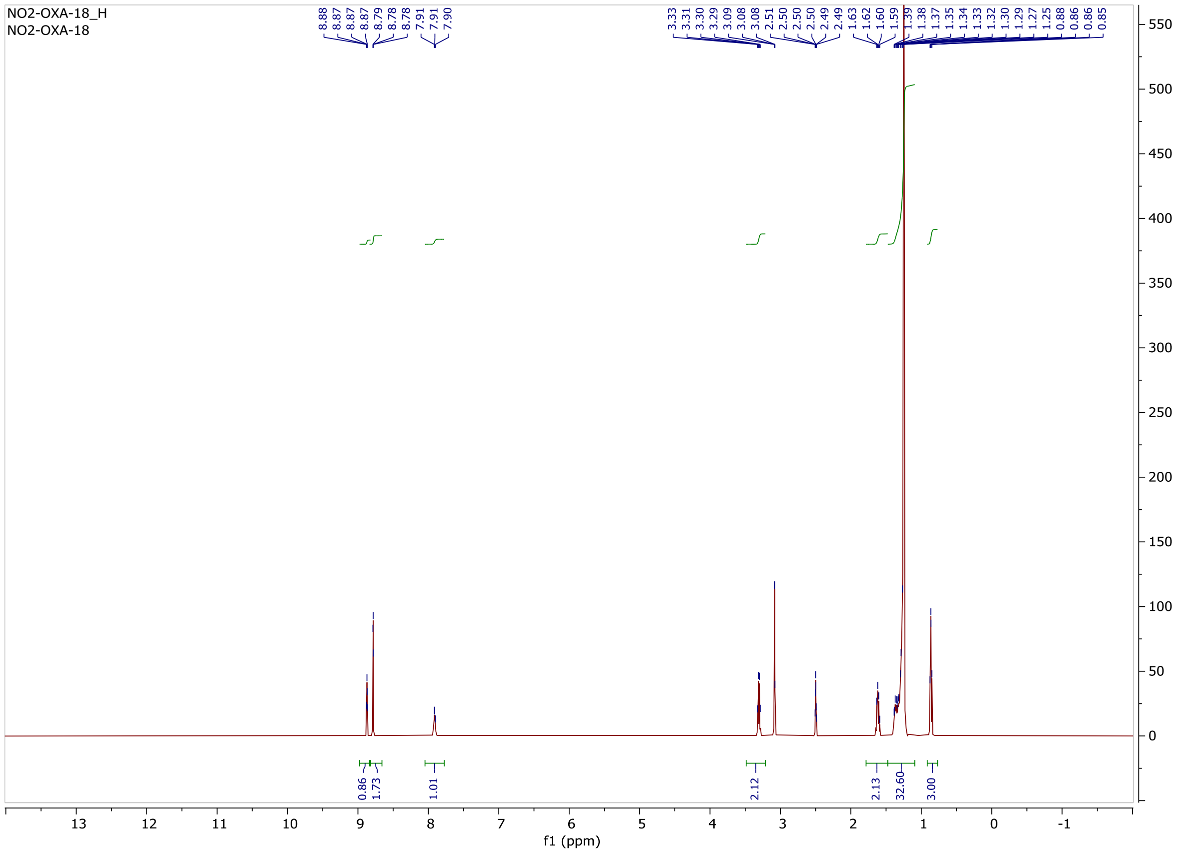

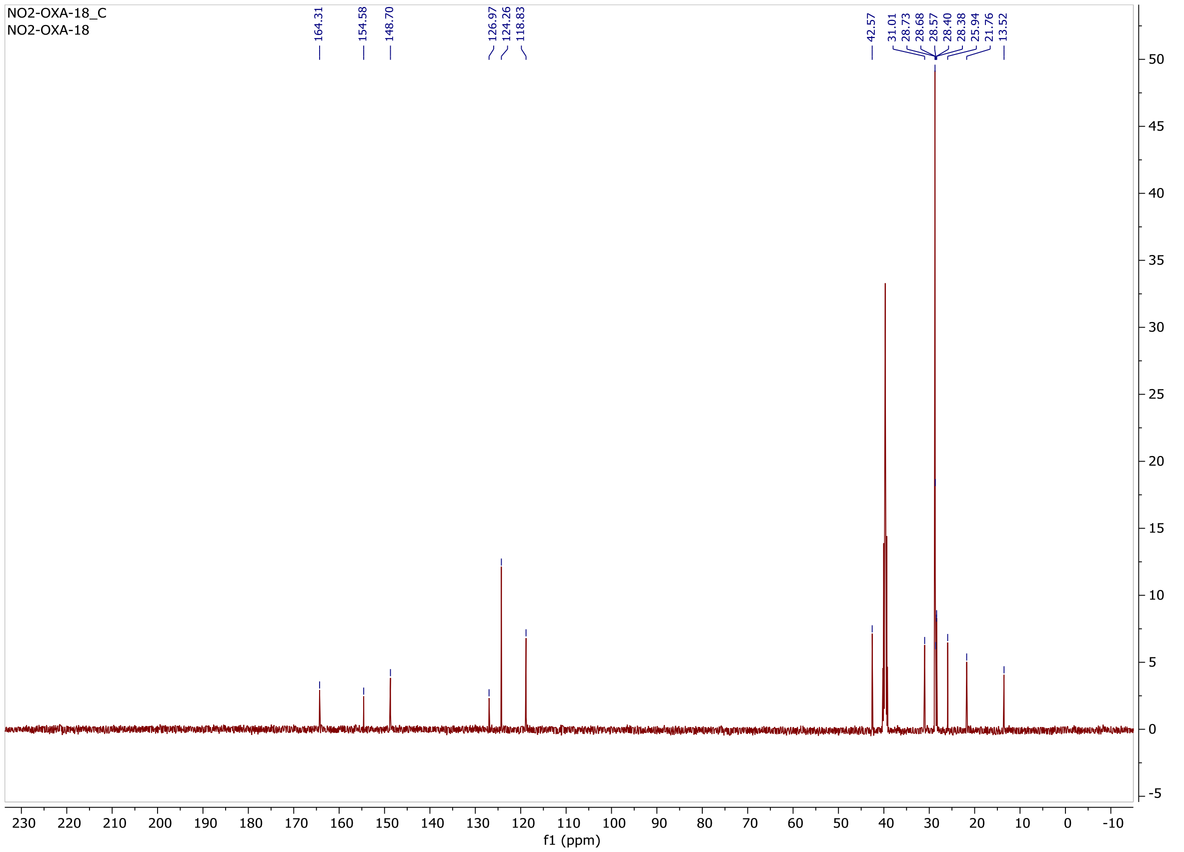

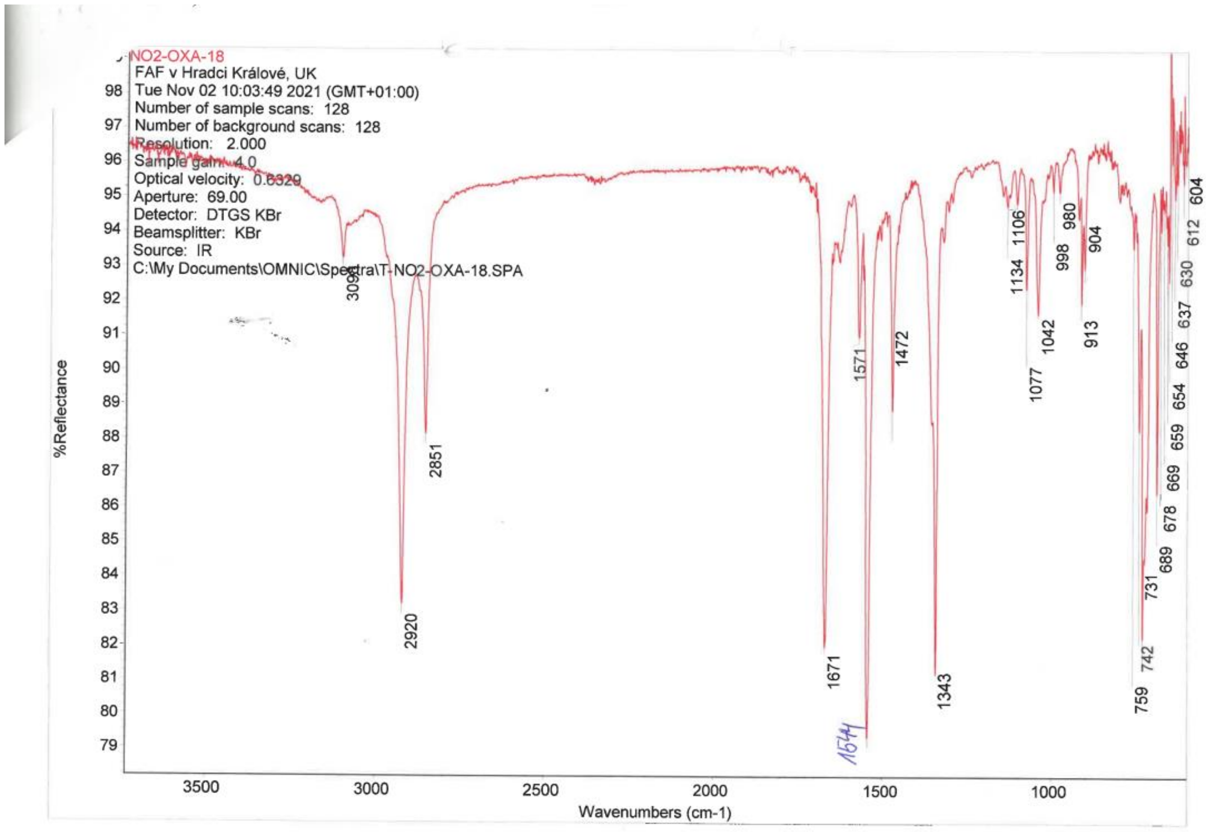

5-(3,5-Dinitrophenyl)-*N*-dodecyl-1,3,4-thiadiazol-2-amine **5s**

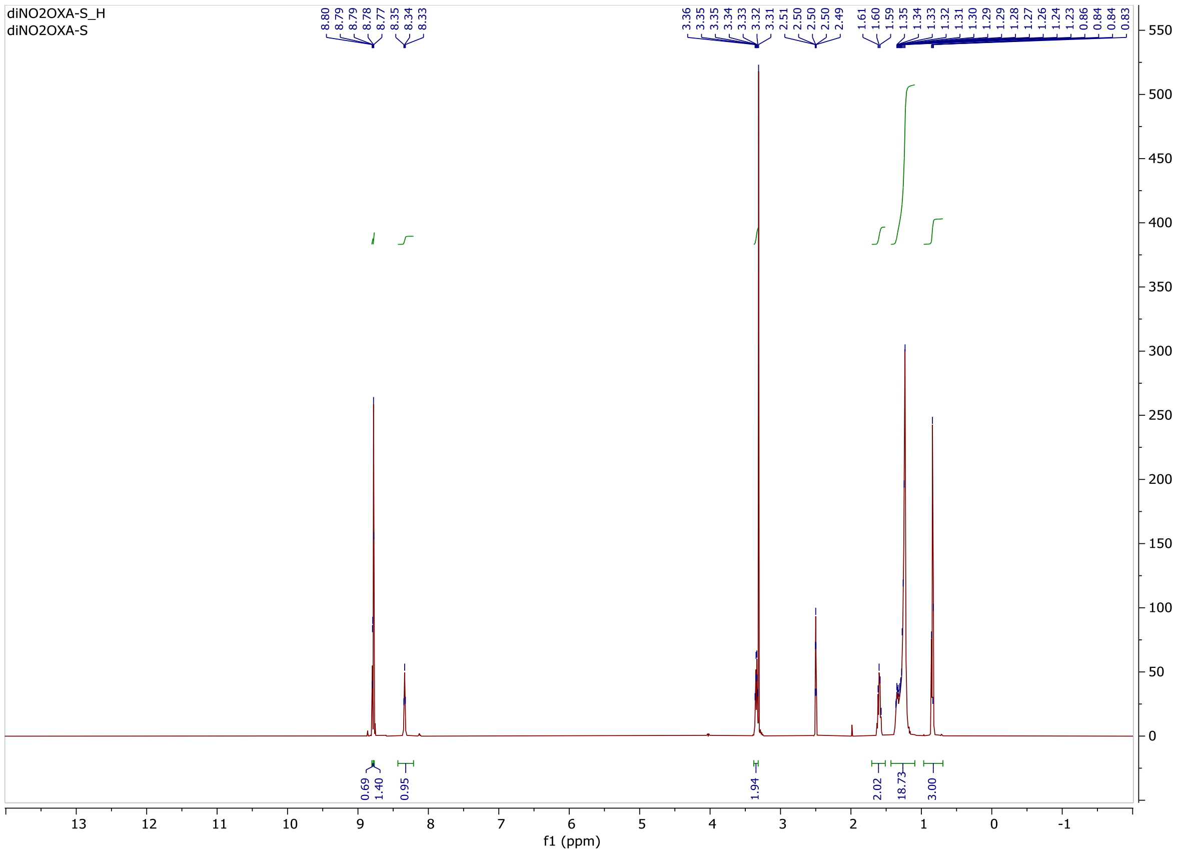

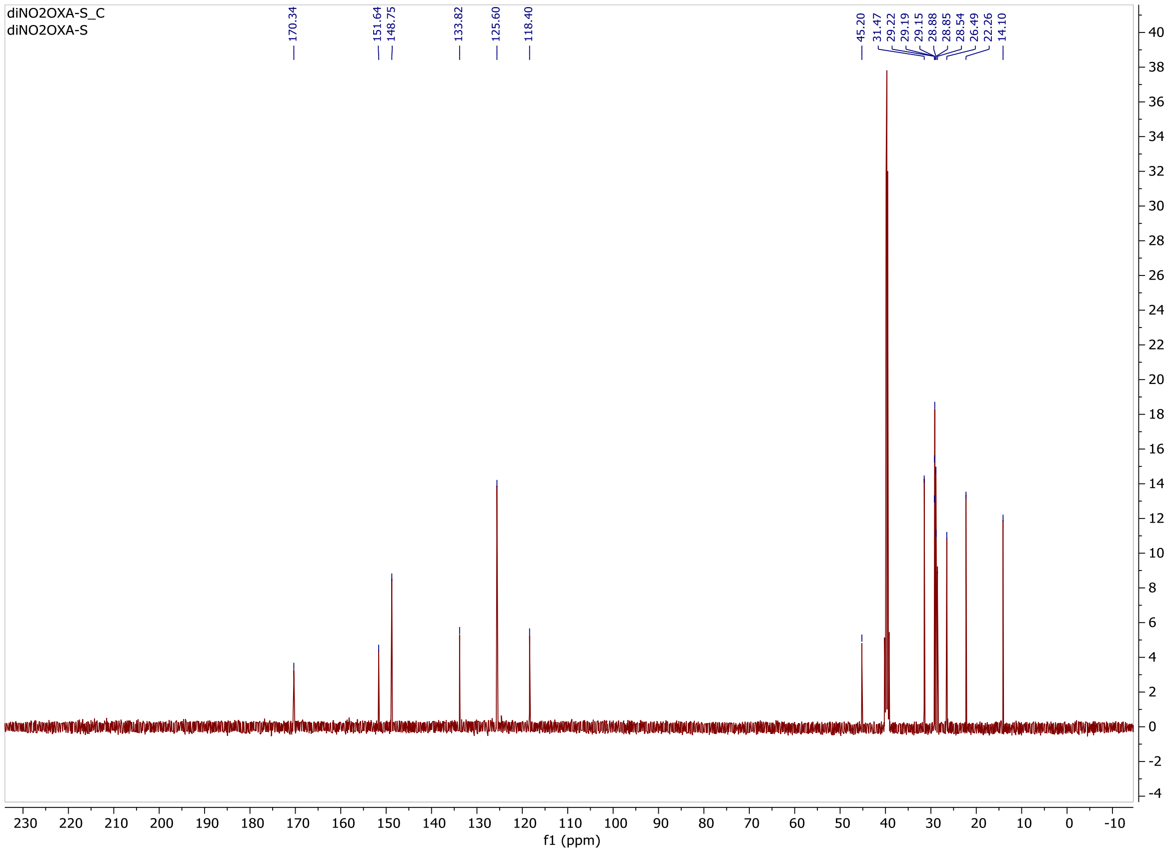

diNO2OXA-S_C
diNO2OXA-S
170.34
151.64
148.75
133.82
125.60
118.40
45.20
31.47
29.22
29.19
29.15
28.88
28.85
28.54
26.49
22.26
14.10
f1 (ppm)

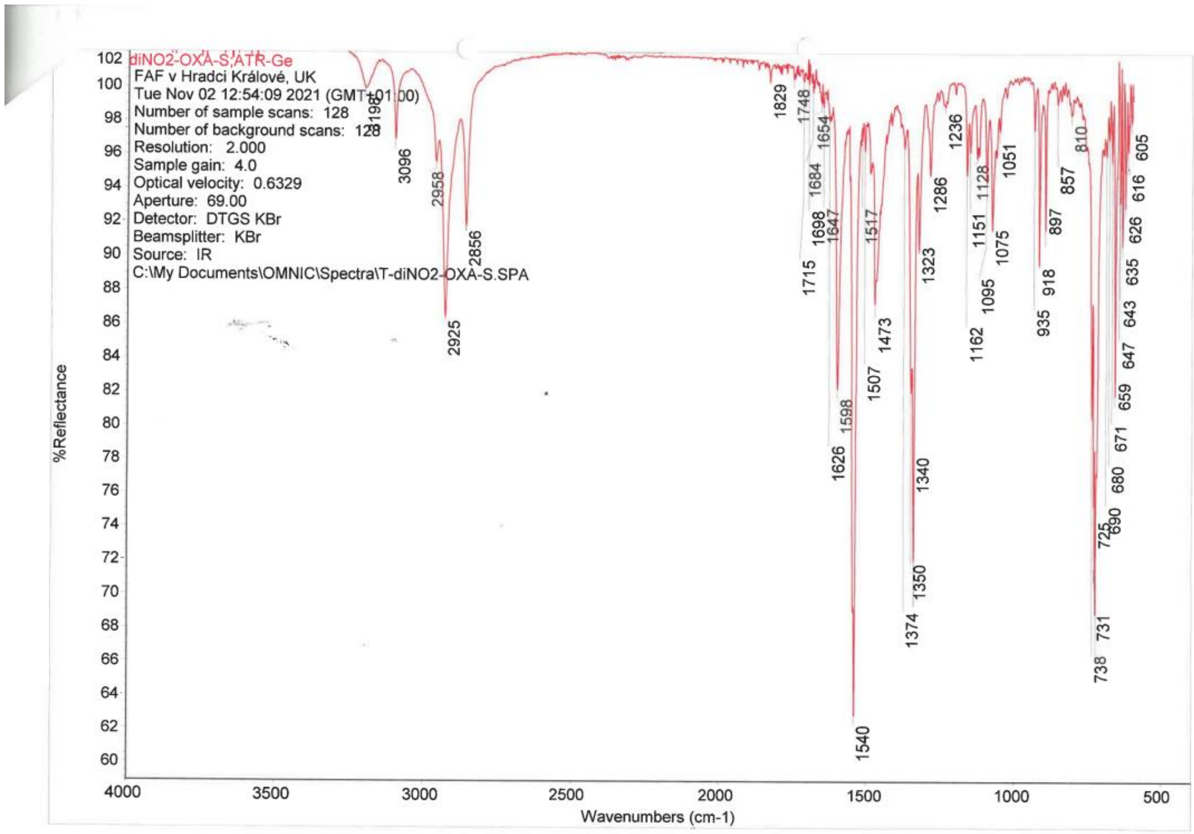

diNO2-OXA-S,ATR-Ge
FAF v Hradci Králové, UK
Tue Nov 02 12:54:09 2021 (GMT+01:00)
Number of sample scans: 128
Number of background scans: 128
Resolution: 2.000
Sample gain: 4.0
Optical velocity: 0.6329
Aperture: 69.00
Detector: DTGS KBr
Beamsplitter: KBr
Source: IR
C:\My Documents\OMNIC\Spectra\T-diNO2-OXA-S.SPA
%Reflectance
Wavenumbers (cm-1)
3096
2958
2925
2856
1829
1748
1715
1698
1684
1654
1647
1626
1598
1540
1517
1507
1473
1374
1350
1340
1323
1286
1236
1162
1151
1128
1095
1075
1051
935
918
897
857
810
738
731
725
690
680
671
659
647
643
635
626
616
605

2-(3,5-Dinitrobenzoyl)-*N*-(4-methoxybenzyl)hydrazine-1-carbothioamide **6b**

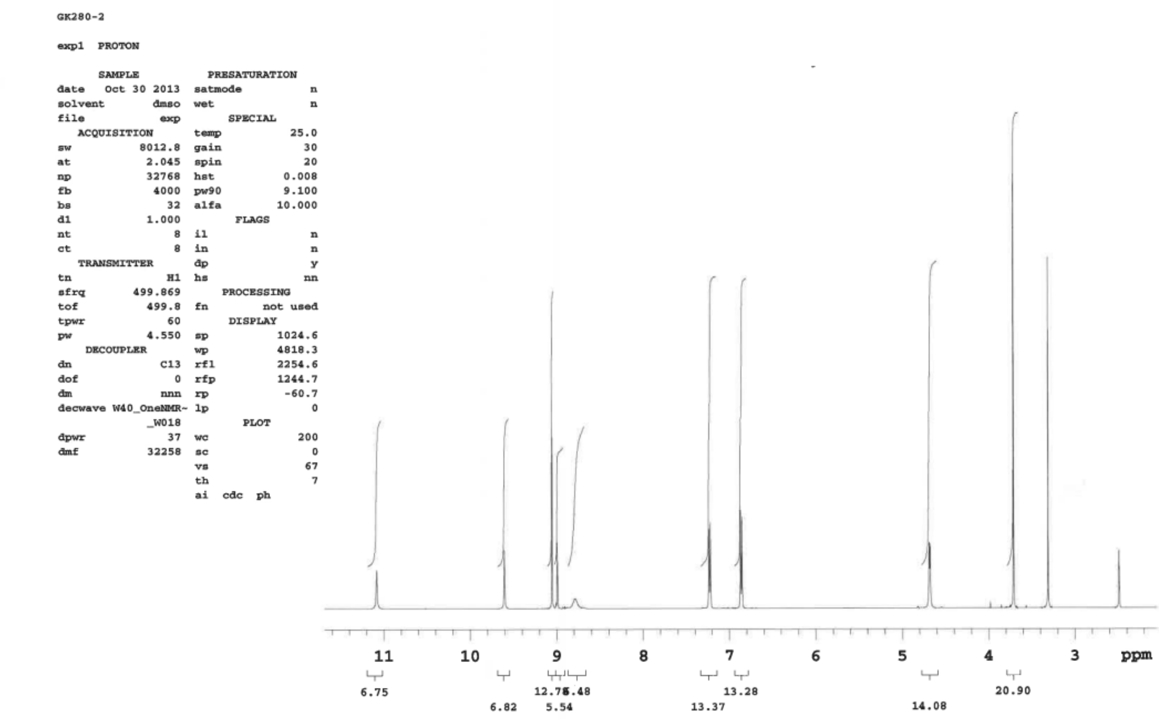

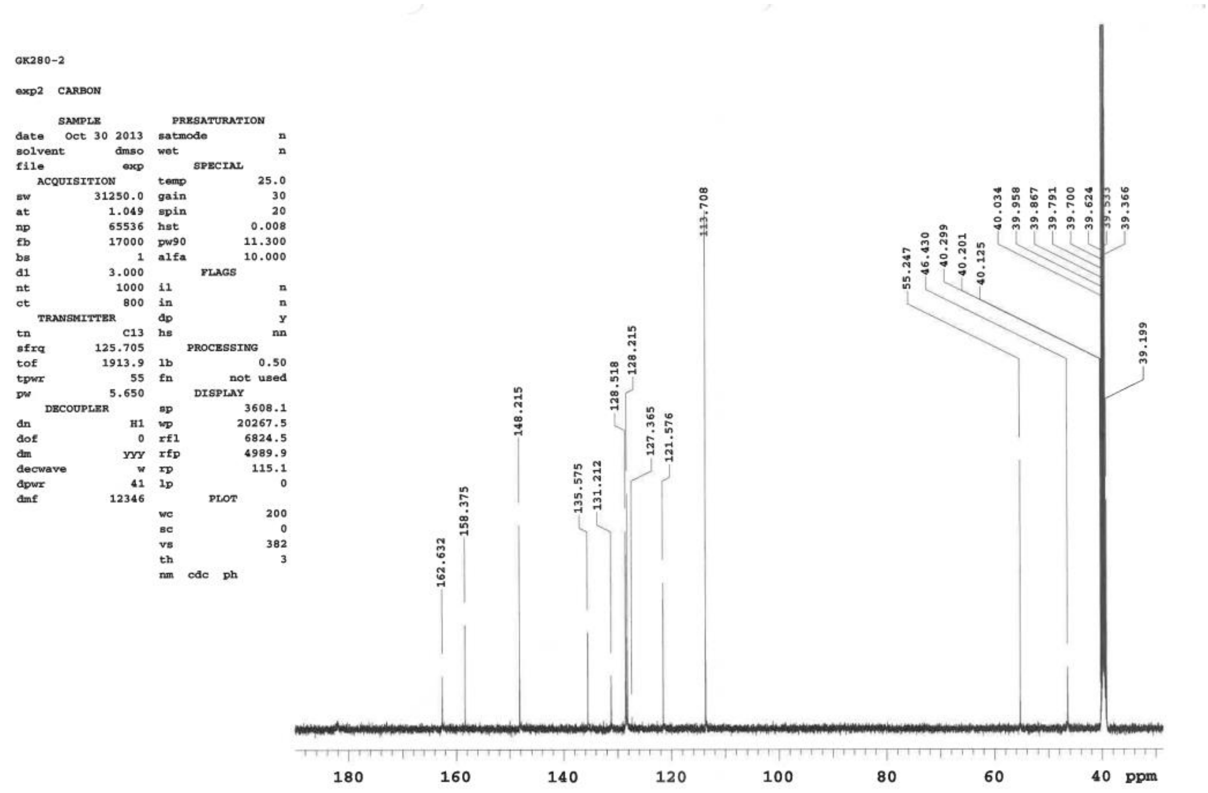

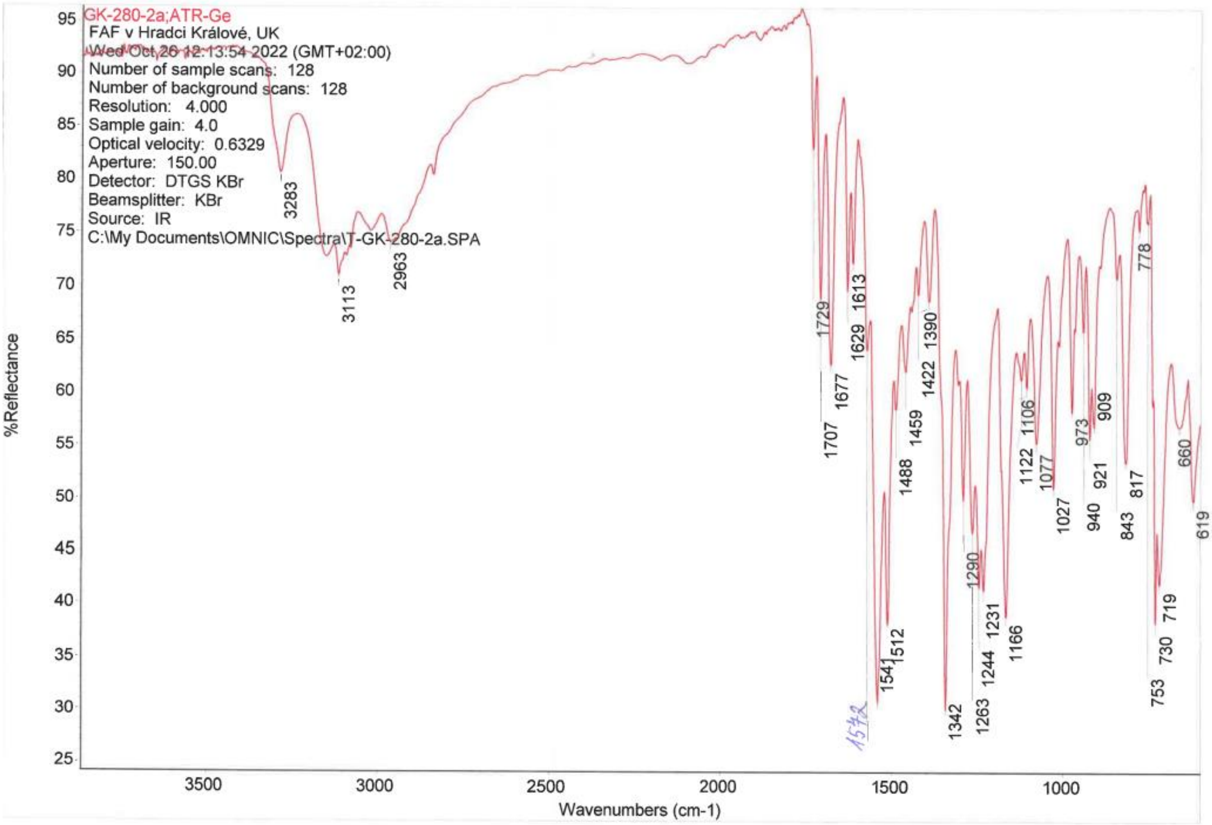

N-(4-Chlorobenzyl)-2-(3,5-dinitrobenzoyl)hydrazine-1-carbothioamide **6c**

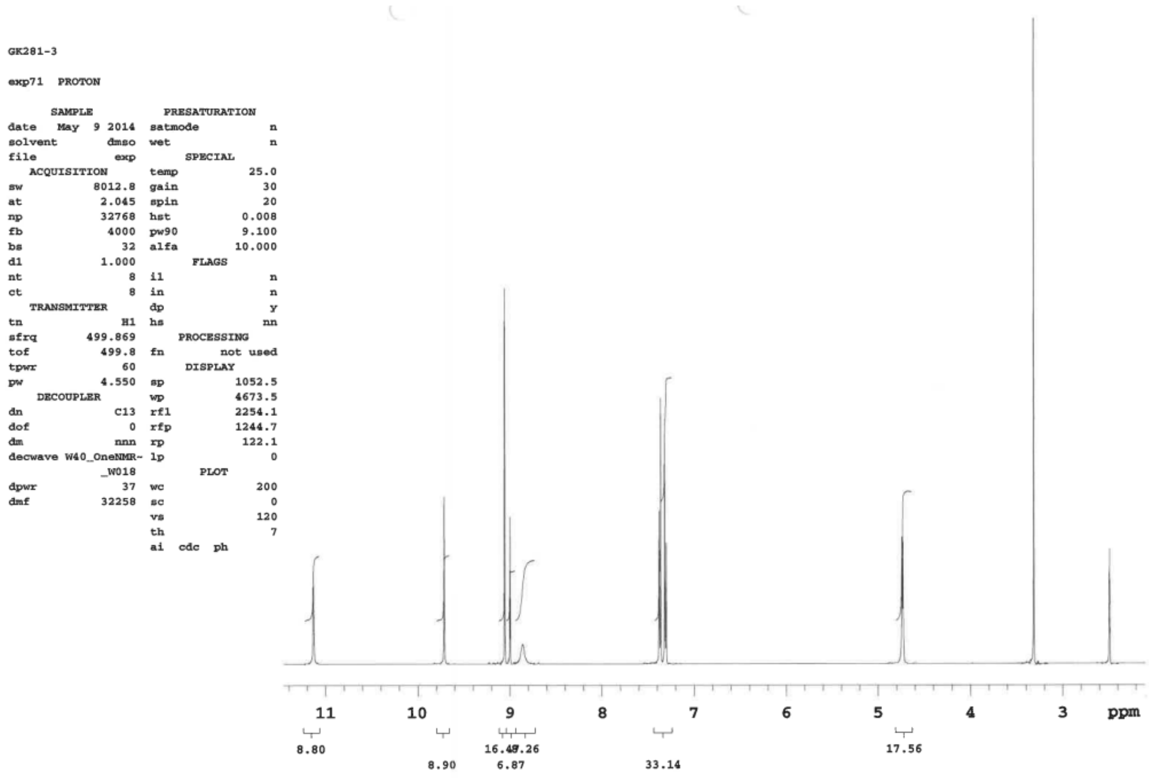

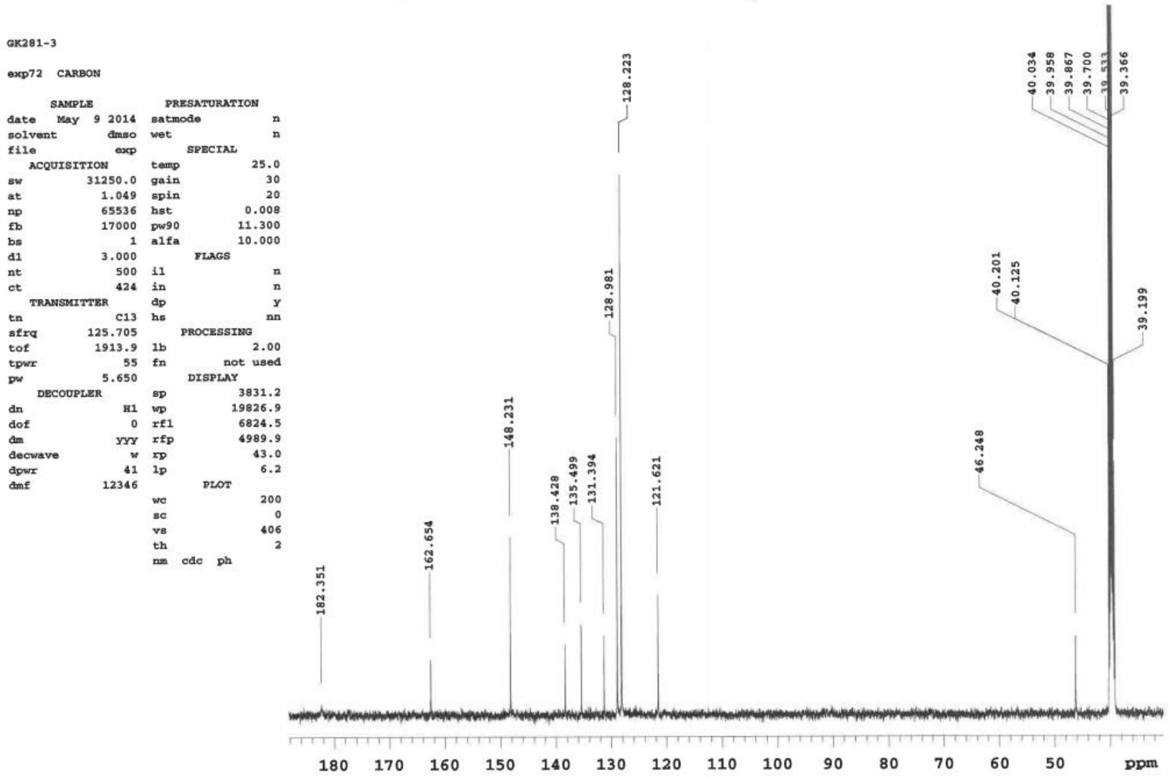
GK281-3
exp72 CARBON
SAMPLE
date May 9 2014
solvent dmso
file exp
ACQUISITION
sw 31250.0
at 1.049
np 65536
fb 17000
bs 1
d1 3.000
nt 500
ct 424
TRANSMITTER
tn C13
sfrq 125.705
tof 1913.9
tpwr 55
pw 5.650
DECOUPLER
dn H1
dof 0
dm yyy
decwave w
dpwr 41
dmf 12346
PRESATURATION
satmode n
wet n
SPECIAL
temp 25.0
gain 30
spin 20
hst 0.008
pw90 11.300
alfa 10.000
FLAGS
il n
in n
dp y
hs nn
PROCESSING
lb 2.00
fn not used
DISPLAY
sp 3831.2
wp 19826.9
rfl 6824.5
rfp 4989.9
rp 43.0
lp 6.2
PLOT
wc 200
sc 0
vs 406
th 2
nm cdc ph
182.351
162.654
148.231
138.428
135.499
131.394
128.981
128.223
121.621
46.248
40.201
40.125
40.034
39.958
39.867
39.700
39.533
39.366
39.199
180 170 160 150 140 130 120 110 100 90 80 70 60 50 ppm

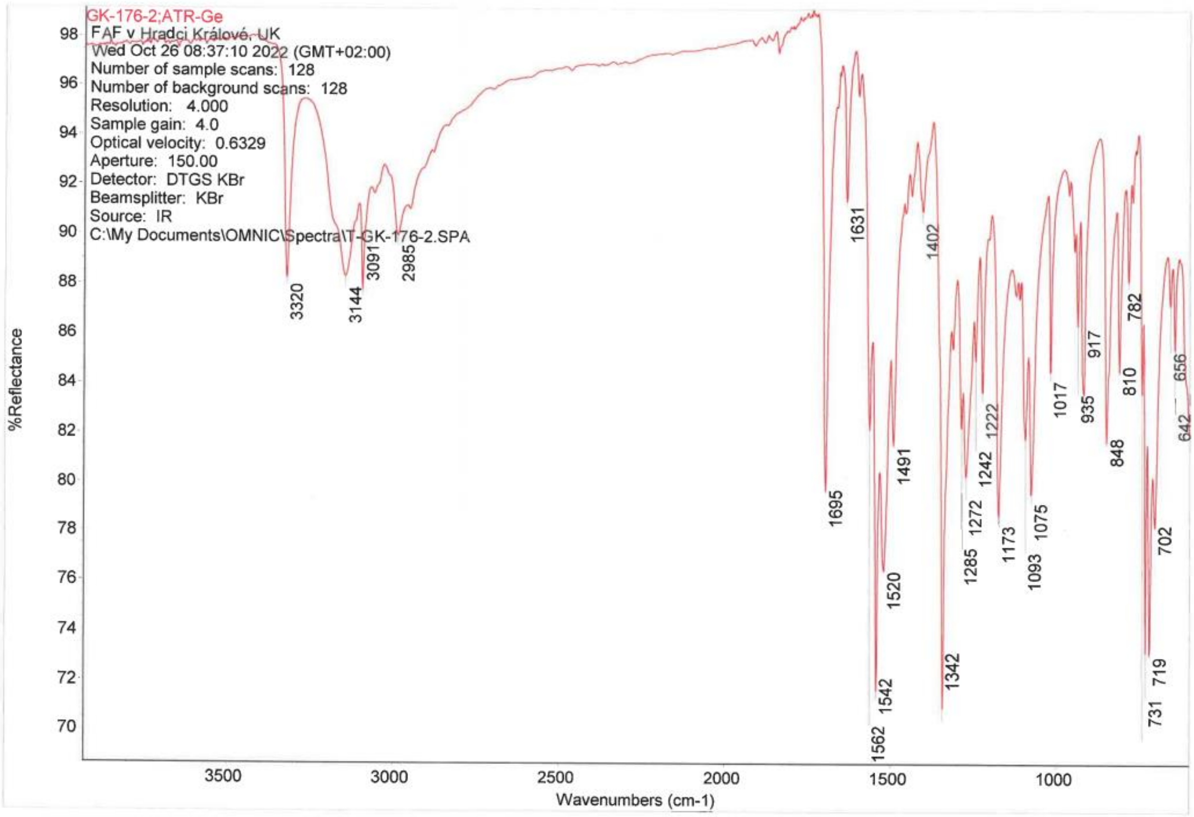
GK-176-2;ATR-Ge
FAF v Hradci Králové, UK
Wed Oct 26 08:37:10 2022 (GMT+02:00)
Number of sample scans: 128
Number of background scans: 128
Resolution: 4.000
Sample gain: 4.0
Optical velocity: 0.6329
Aperture: 150.00
Detector: DTGS KBr
Beamsplitter: KBr
Source: IR
C:\My Documents\OMNIC\Spectra\T-GK-176-2.SPA
%Reflectance
98
96
94
92
90
88
86
84
82
80
78
76
74
72
70
3320
3144
3091
2985
1695
1631
1562
1542
1520
1491
1402
1342
1285
1272
1242
1222
1173
1093
1075
1017
935
917
848
810
782
731
719
702
656
642
3500 3000 2500 2000 1500 1000
Wavenumbers (cm-1)

N-(2,4-Dichlorobenzyl)-2-(3,5-dinitrobenzoyl)hydrazine-1-carbothioamide **6d**

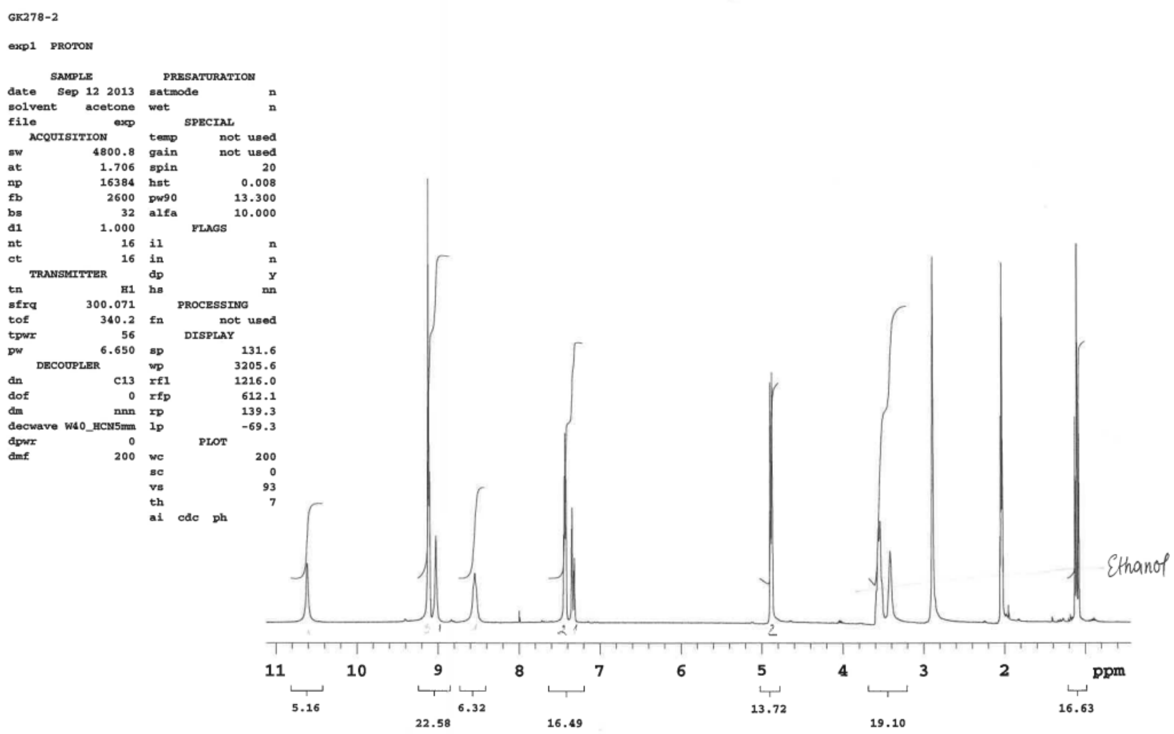

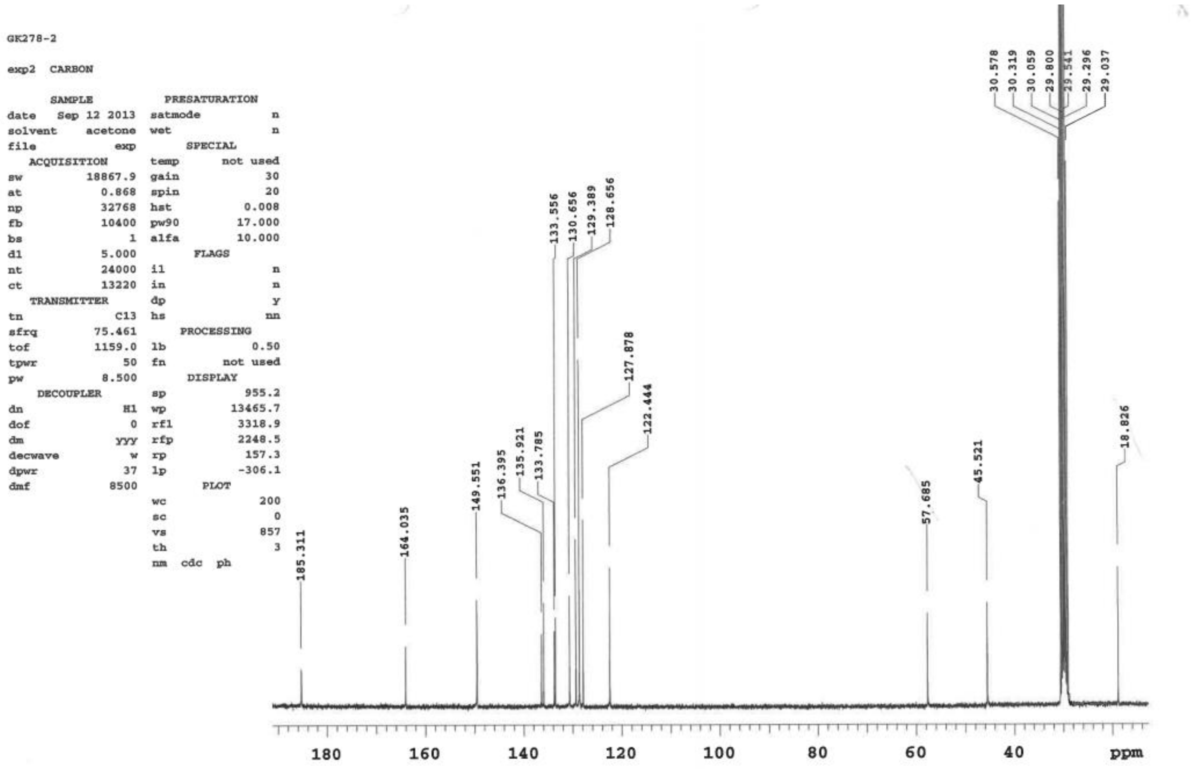

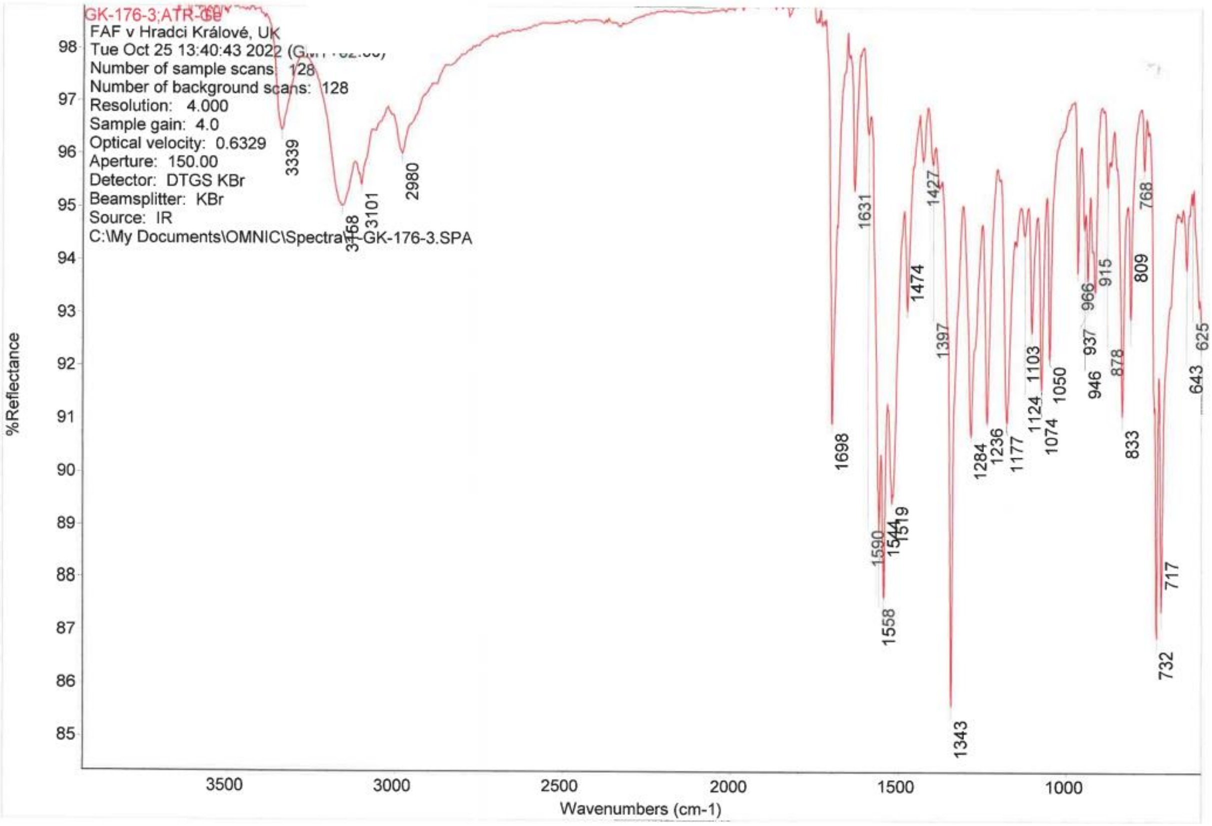

5-(3,5-Dinitrophenyl)-*N*-(4-methoxybenzyl)-1,3,4-oxadiazol-2-amine **7b**

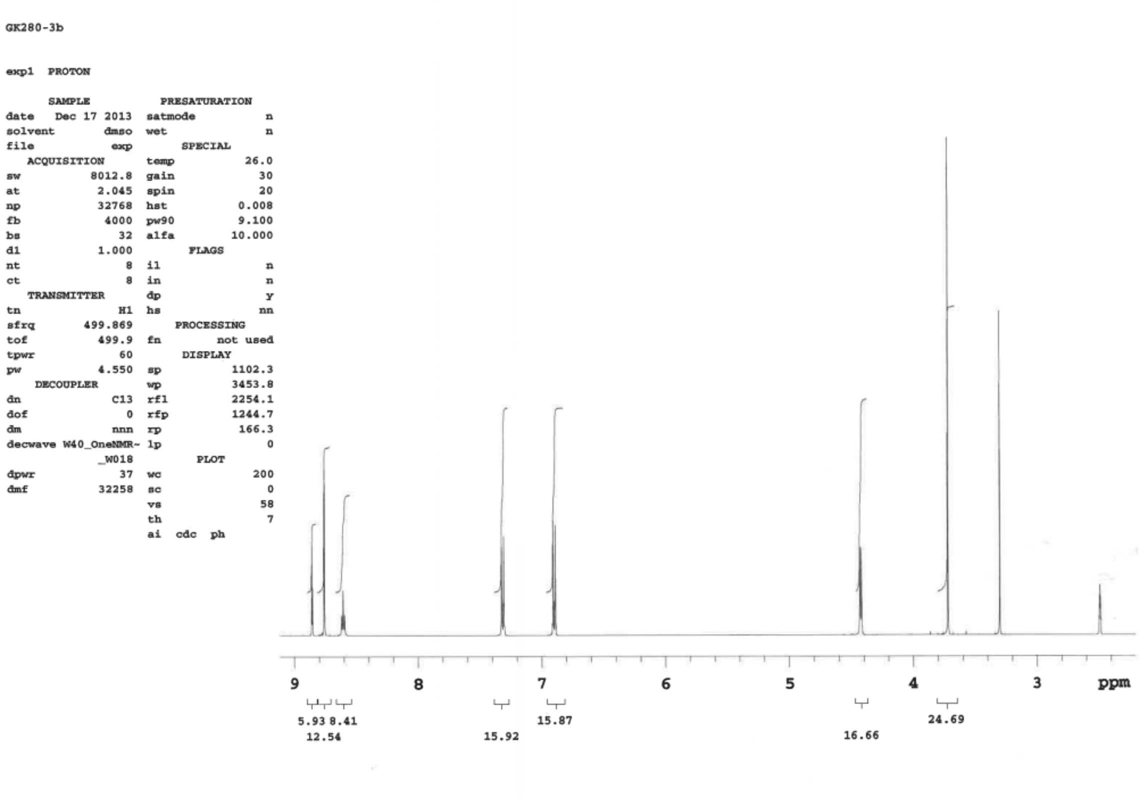

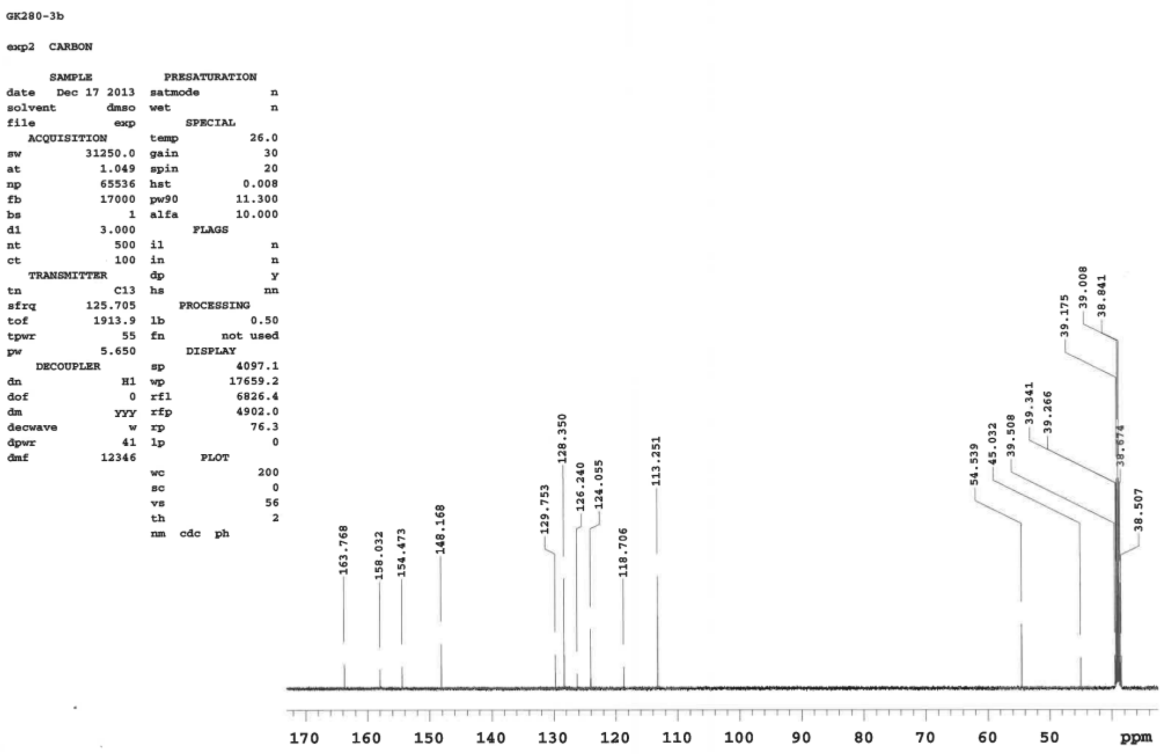
GK280-3b
exp2 CARBON
SAMPLE
date Dec 17 2013
solvent dmso
file exp
ACQUISITION
sw 31250.0
at 1.049
np 65536
fb 17000
bs 1
d1 3.000
nt 500
ct 100
TRANSMITTER
tn C13
sfrq 125.705
tof 1913.9
tpwr 55
pw 5.650
DECOUPLER
dn H1
dof 0
dm yyy
decwave w
dpwr 41
dmf 12346
PRESATURATION
satmode n
wet n
SPECIAL
temp 26.0
gain 30
spin 20
hst 0.008
pw90 11.300
alfa 10.000
FLAGS
il n
in n
dp y
hs nn
PROCESSING
lb 0.50
fn not used
DISPLAY
sp 4097.1
wp 17659.2
rfl 6826.4
rfp 4902.0
rp 76.3
lp 0
PLOT
wc 200
sc 0
vs 56
th 2
nm cdc ph
163.768
158.032
154.473
148.168
129.753
128.350
126.240
124.055
118.706
113.251
54.539
45.032
39.508
39.341
39.266
39.175
39.008
38.841
38.674
38.507
170 160 150 140 130 120 110 100 90 80 70 60 50 ppm

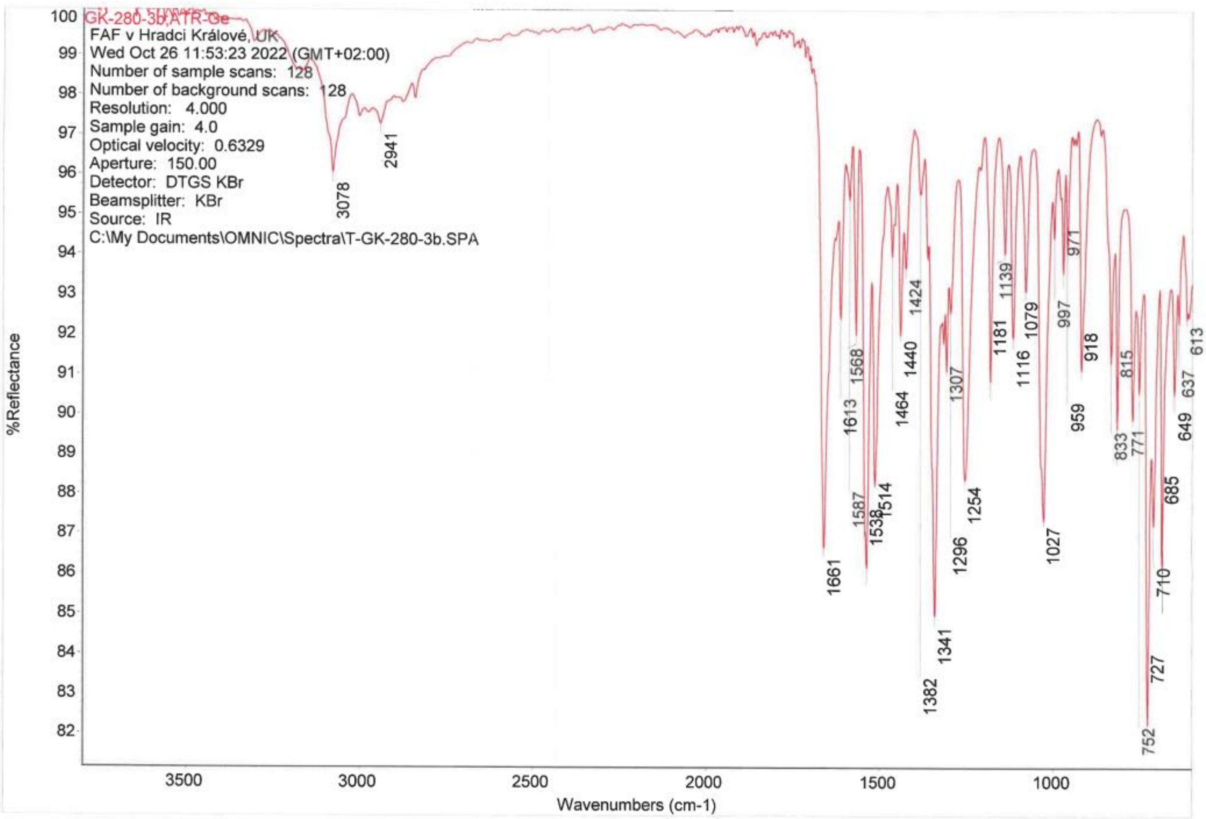
GK-280-3b, ATR-Ge
FAF v Hradci Králové, UK
Wed Oct 26 11:53:23 2022 (GMT+02:00)
Number of sample scans: 128
Number of background scans: 128
Resolution: 4.000
Sample gain: 4.0
Optical velocity: 0.6329
Aperture: 150.00
Detector: DTGS KBr
Beamsplitter: KBr
Source: IR
C:\My Documents\OMNIC\Spectra\T-GK-280-3b.SPA
%Reflectance
Wavenumbers (cm-1)
3078
2941
1661
1613
1587
1568
1538
1514
1464
1440
1424
1382
1341
1307
1296
1254
1181
1139
1116
1079
1027
997
971
959
918
833
815
771
752
727
710
685
649
637
613

N-(4-Chlorobenzyl)-5-(3,5-dinitrophenyl)-1,3,4-oxadiazol-2-amine **7c**

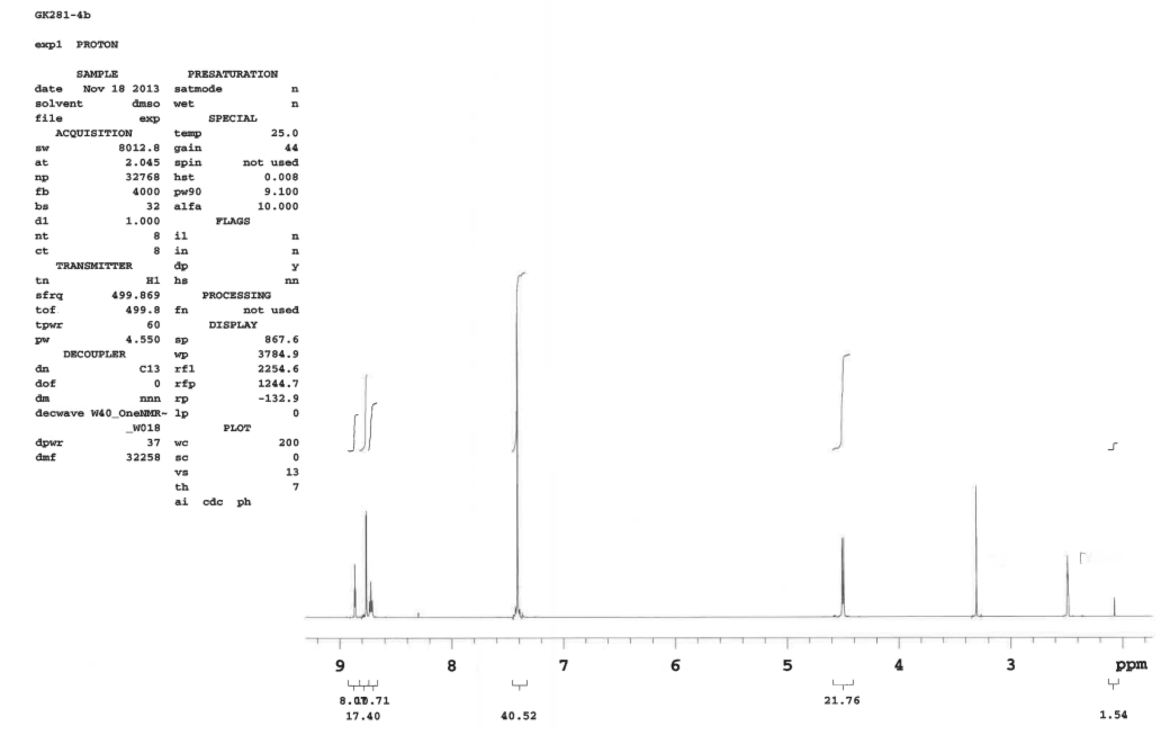

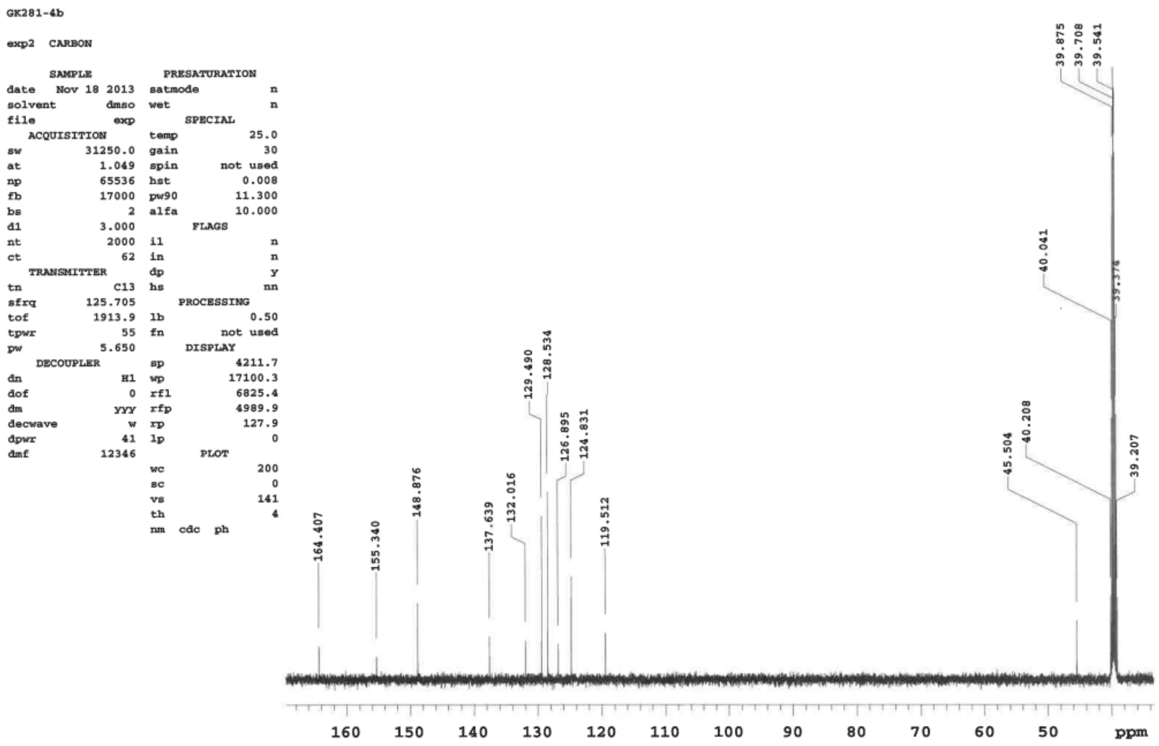

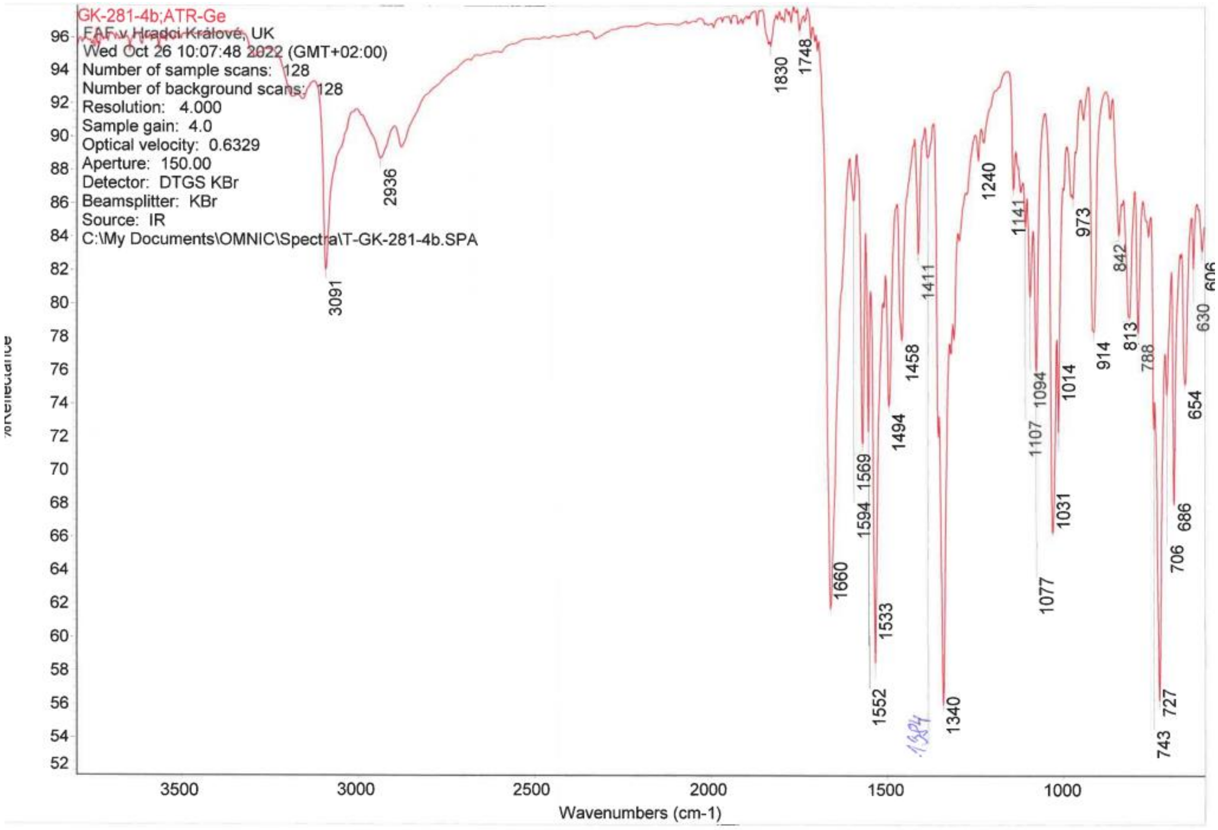

N-(2,4-Dichlorobenzyl)-5-(3,5-dinitrophenyl)-1,3,4-oxadiazol-2-amine **7d**

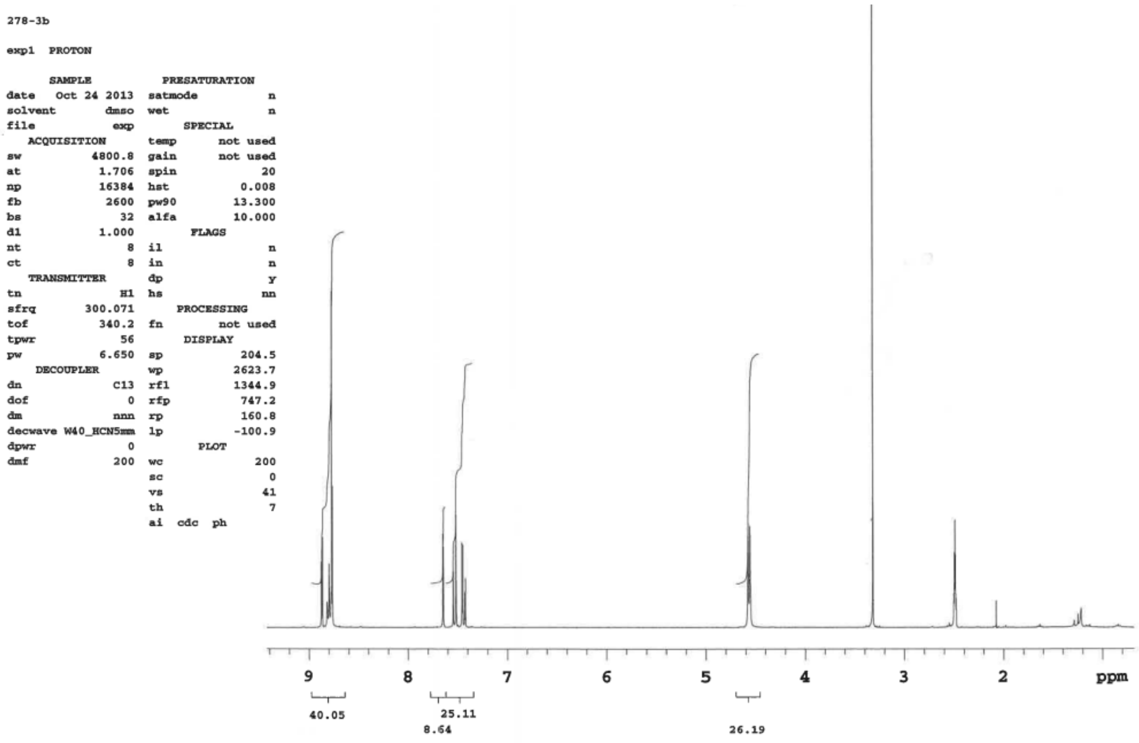

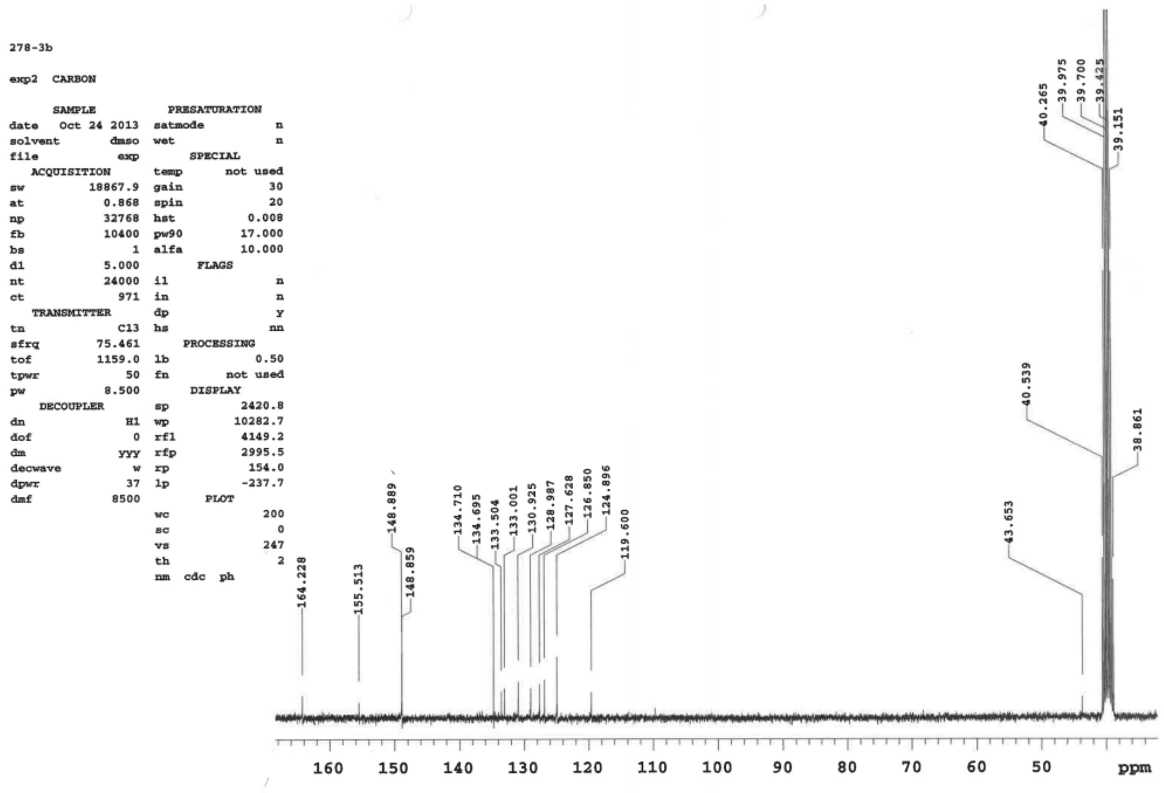
278-3b
exp2 CARBON
SAMPLE
date Oct 24 2013
solvent dmso
file exp
ACQUISITION
sw 18867.9
at 0.868
np 32768
fb 10400
bs 1
d1 5.000
nt 24000
ct 971
TRANSMITTER
tn C13
sfrq 75.461
tof 1159.0
tpwr 50
pw 8.500
DECOUPLER
dn H1
dof 0
dm yyy
decwave w
dpwr 37
dmf 8500
PRESATURATION
satmode n
wet n
SPECIAL
temp not used
gain 30
spin 20
hst 0.008
pw90 17.000
alfa 10.000
FLAGS
il n
in n
dp y
hs nn
PROCESSING
lb 0.50
fn not used
DISPLAY
sp 2420.8
wp 10282.7
rfl 4149.2
rfp 2995.5
rp 154.0
lp -237.7
PLOT
wc 200
sc 0
vs 247
th 2
nm cdc ph
164.228
155.513
148.889
148.859
134.710
134.695
133.504
133.001
130.925
128.987
127.628
126.850
124.896
119.600
43.653
40.539
40.265
39.975
39.700
39.425
39.151
38.861
160 150 140 130 120 110 100 90 80 70 60 50 ppm

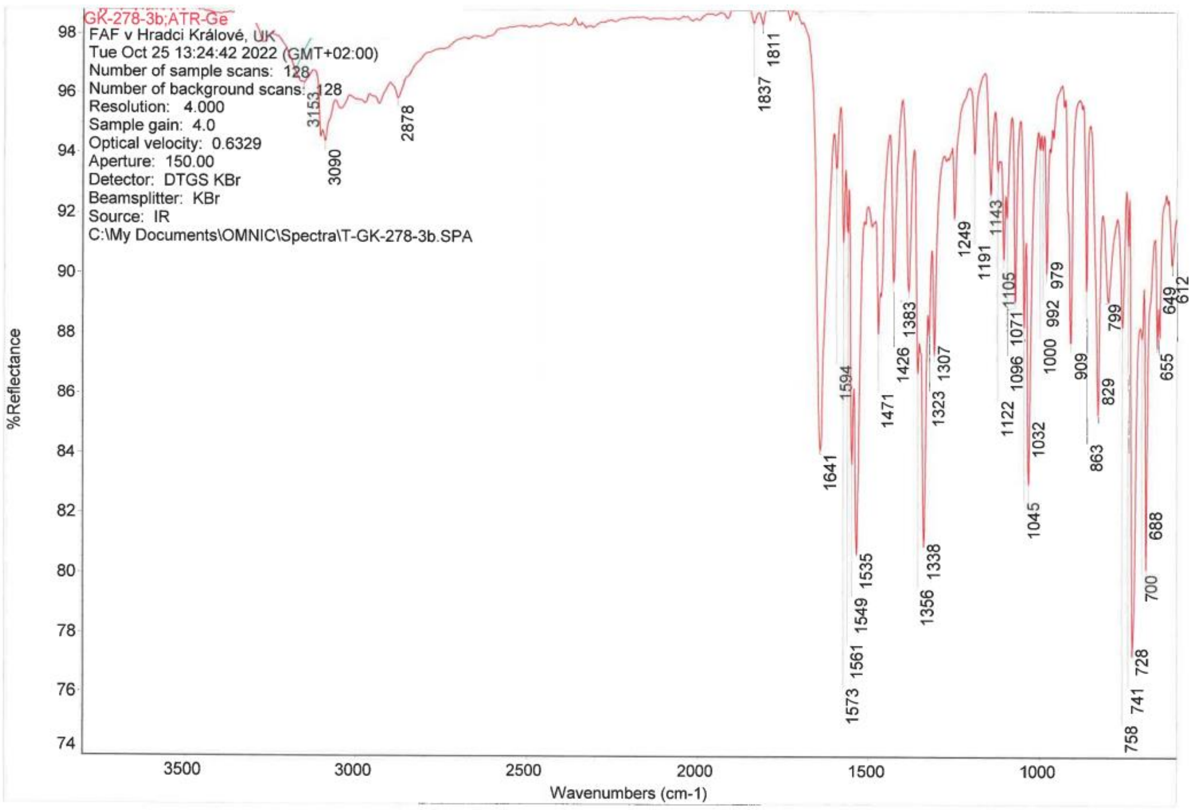
GK-278-3b;ATR-Ge
FAF v Hradci Králové, UK
Tue Oct 25 13:24:42 2022 (GMT+02:00)
Number of sample scans: 128
Number of background scans: 128
Resolution: 4.000
Sample gain: 4.0
Optical velocity: 0.6329
Aperture: 150.00
Detector: DTGS KBr
Beamsplitter: KBr
Source: IR
C:\My Documents\OMNIC\Spectra\T-GK-278-3b.SPA
%Reflectance
Wavenumbers (cm-1)
3153
3090
2878
1837
1811
1641
1594
1573
1561
1549
1535
1471
1426
1383
1356
1338
1323
1307
1249
1191
1143
1122
1105
1096
1071
1045
1032
1000
992
979
909
863
829
799
758
741
728
700
688
655
649
612

N-(4-Bromobenzyl)-5-(3,5-dinitrophenyl)-1,3,4-oxadiazol-2-amine **7e**

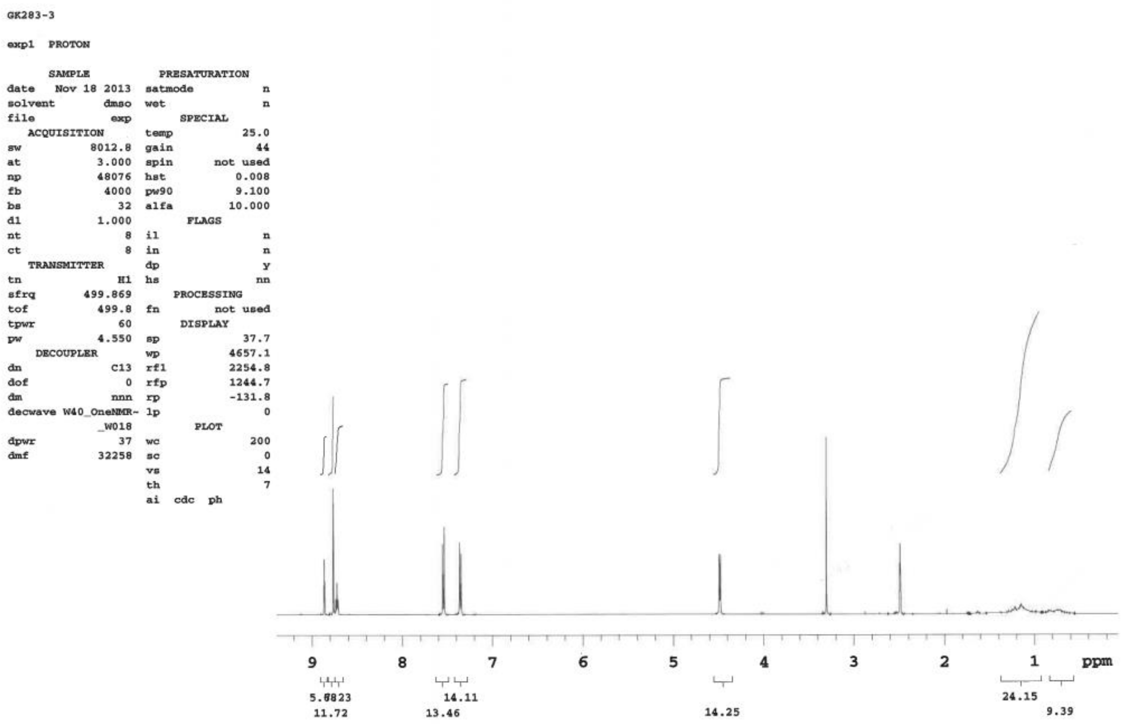

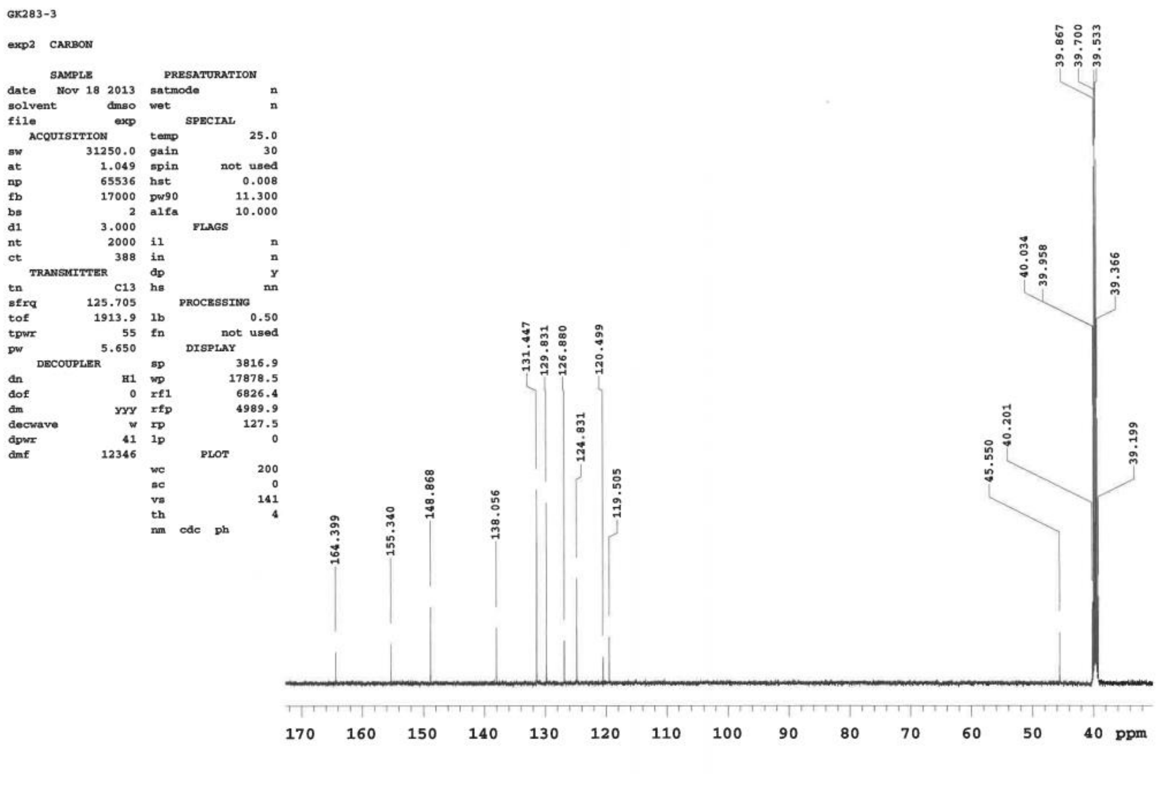

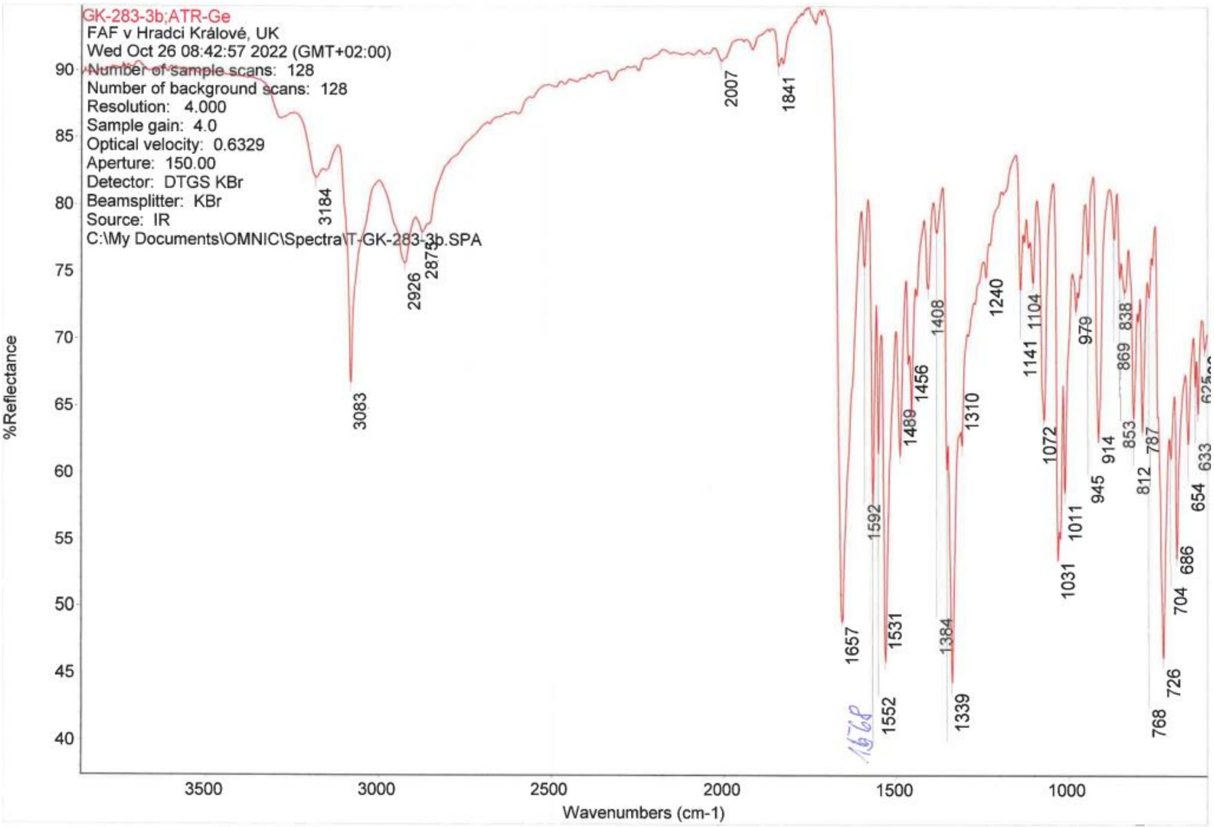

5-(3,5-Dinitrophenyl)-*N*-(4-methoxybenzyl)-1,3,4-thiadiazol-2-amine **8b**

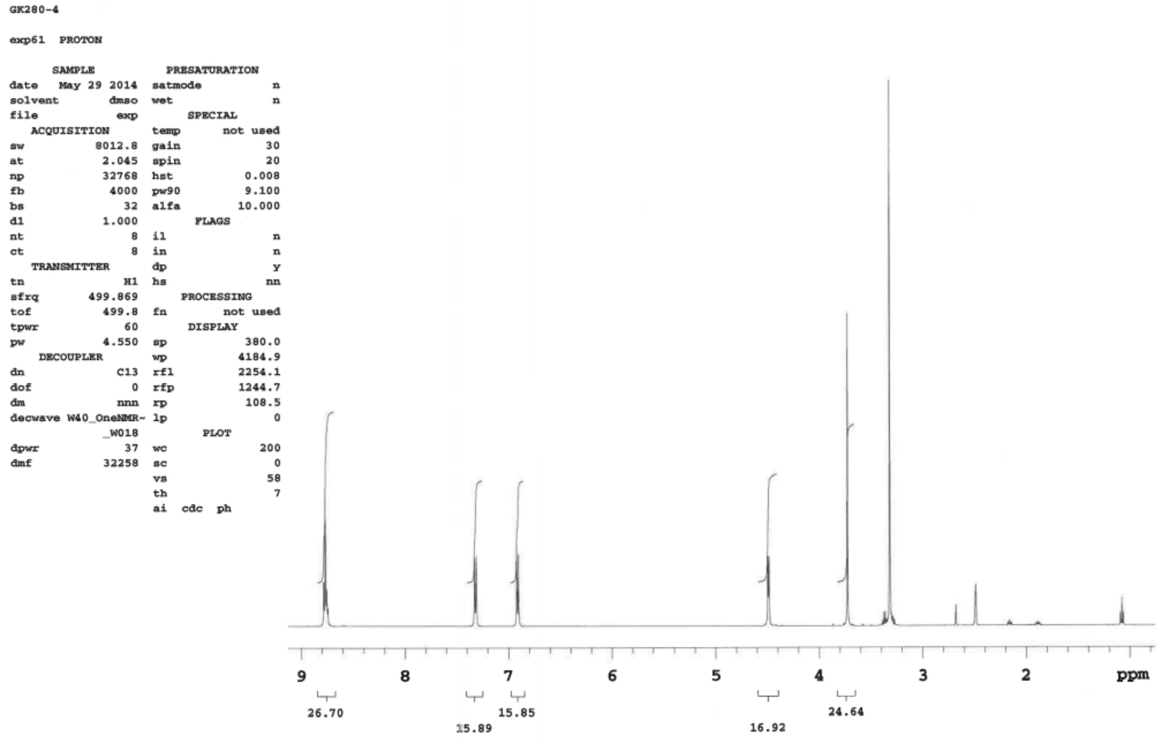

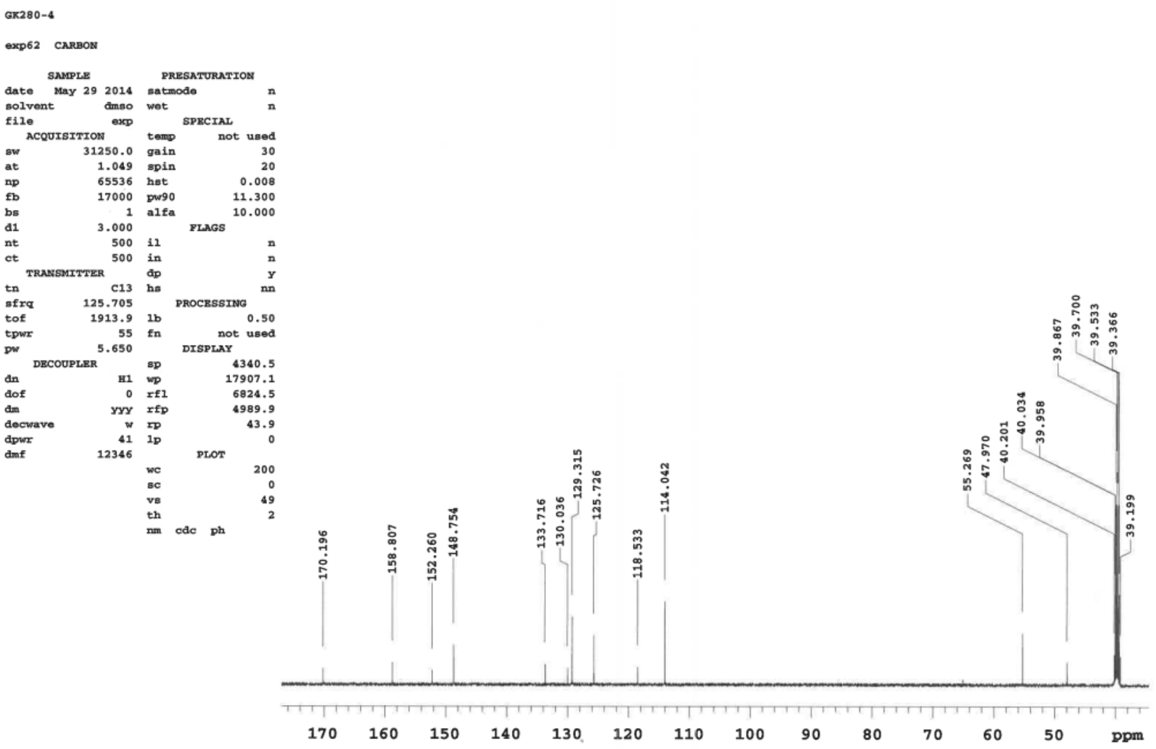
GK280-4
exp62 CARBON
SAMPLE
date May 29 2014
solvent dmso
file exp
ACQUISITION
sw 31250.0
at 1.049
np 65536
fb 17000
bs 1
d1 3.000
nt 500
ct 500
TRANSMITTER
tn C13
sfrq 125.705
tof 1913.9
tpwr 55
pw 5.650
DECOUPLER
dn H1
dof 0
dm yyy
decwave w
dpwr 41
dmf 12346
PRESATURATION
satmode n
wet n
SPECIAL
temp not used
gain 30
spin 20
hst 0.008
pw90 11.300
alfa 10.000
FLAGS
il n
in n
dp y
hs nn
PROCESSING
lb 0.50
fn not used
DISPLAY
sp 4340.5
wp 17907.1
rfl 6824.5
rfp 4989.9
rp 43.9
lp 0
PLOT
wc 200
sc 0
vs 49
th 2
nm cdc ph
170.196
158.807
152.260
148.754
133.716
130.036
129.315
125.726
118.533
114.042
55.269
47.970
40.201
40.034
39.958
39.867
39.700
39.533
39.366
39.199
170 160 150 140 130 120 110 100 90 80 70 60 50 ppm

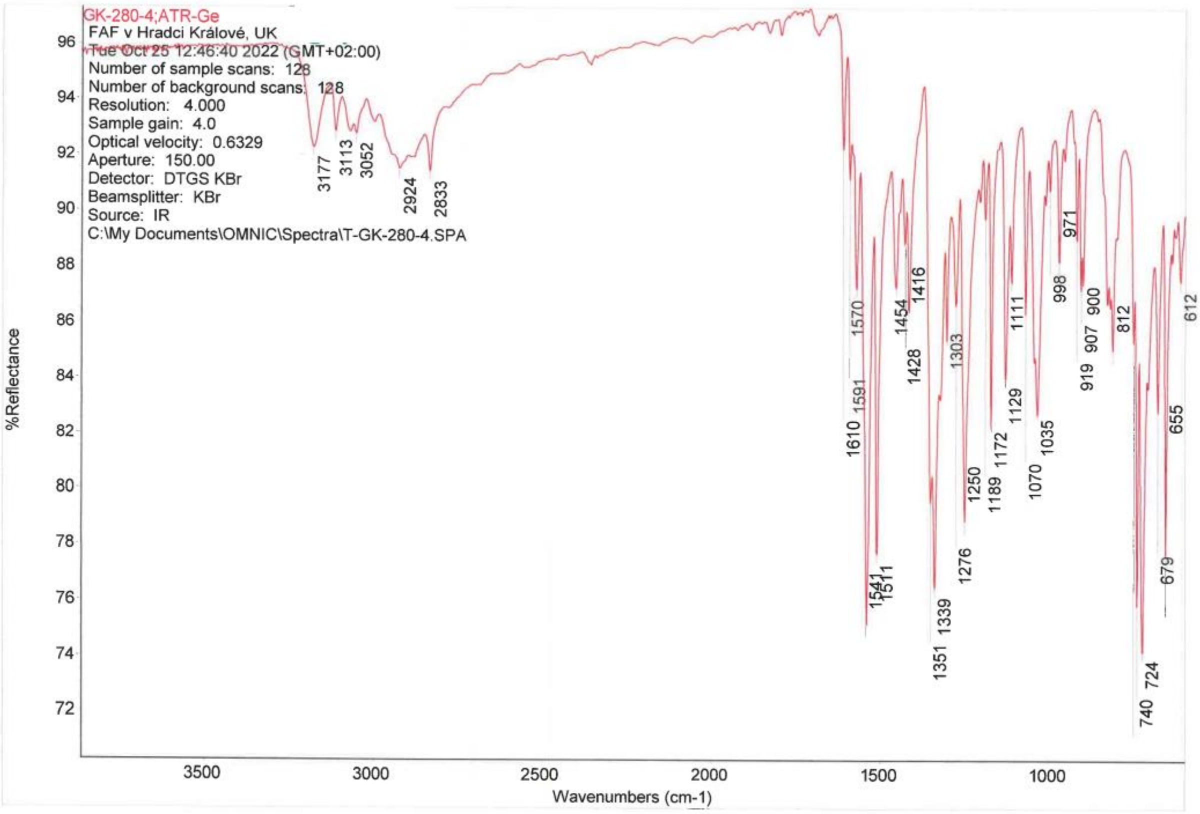
GK-280-4;ATR-Ge
FAF v Hradci Králové, UK
Tue Oct 25 12:46:40 2022 (GMT+02:00)
Number of sample scans: 128
Number of background scans: 128
Resolution: 4.000
Sample gain: 4.0
Optical velocity: 0.6329
Aperture: 150.00
Detector: DTGS KBr
Beamsplitter: KBr
Source: IR
C:\My Documents\OMNIC\Spectra\T-GK-280-4.SPA
%Reflectance
Wavenumbers (cm-1)
3177
3113
3052
2924
2833
1610
1591
1570
1541
1511
1454
1428
1416
1351
1339
1303
1276
1250
1189
1172
1129
1111
1070
1035
998
971
919
907
900
812
740
724
679
655
612

N-(4-Chlorobenzyl)-5-(3,5-dinitrophenyl)-1,3,4-thiadiazol-2-amine **8c**

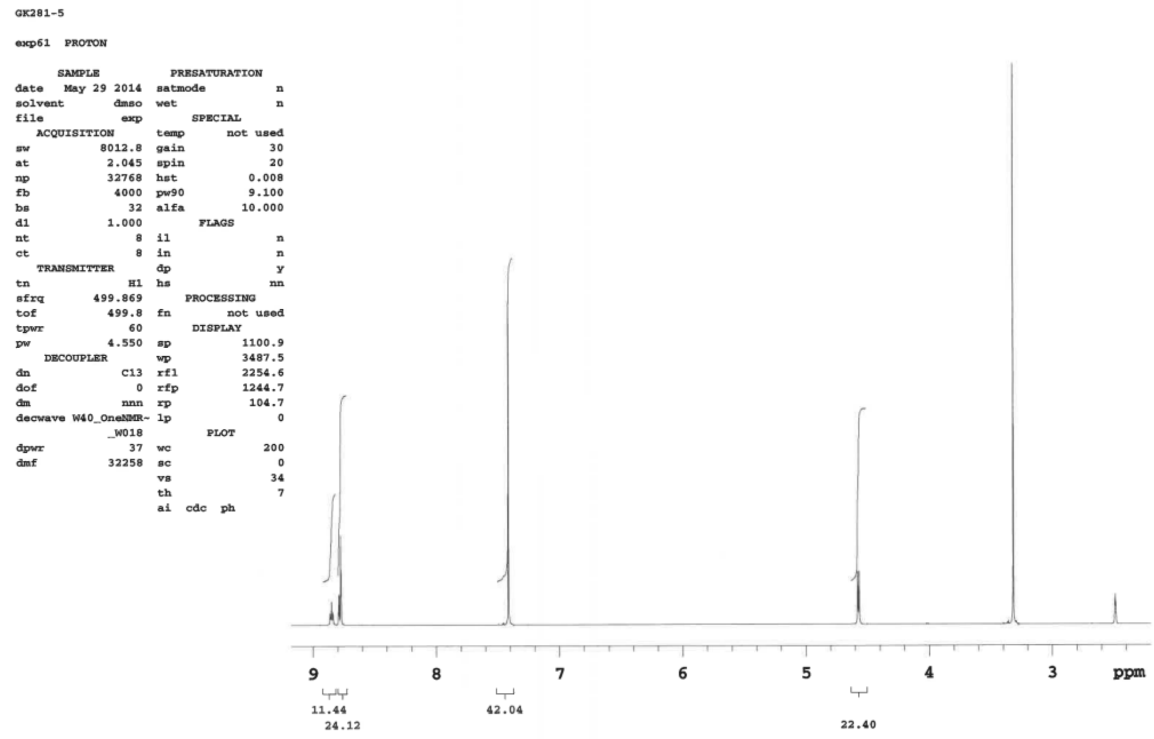

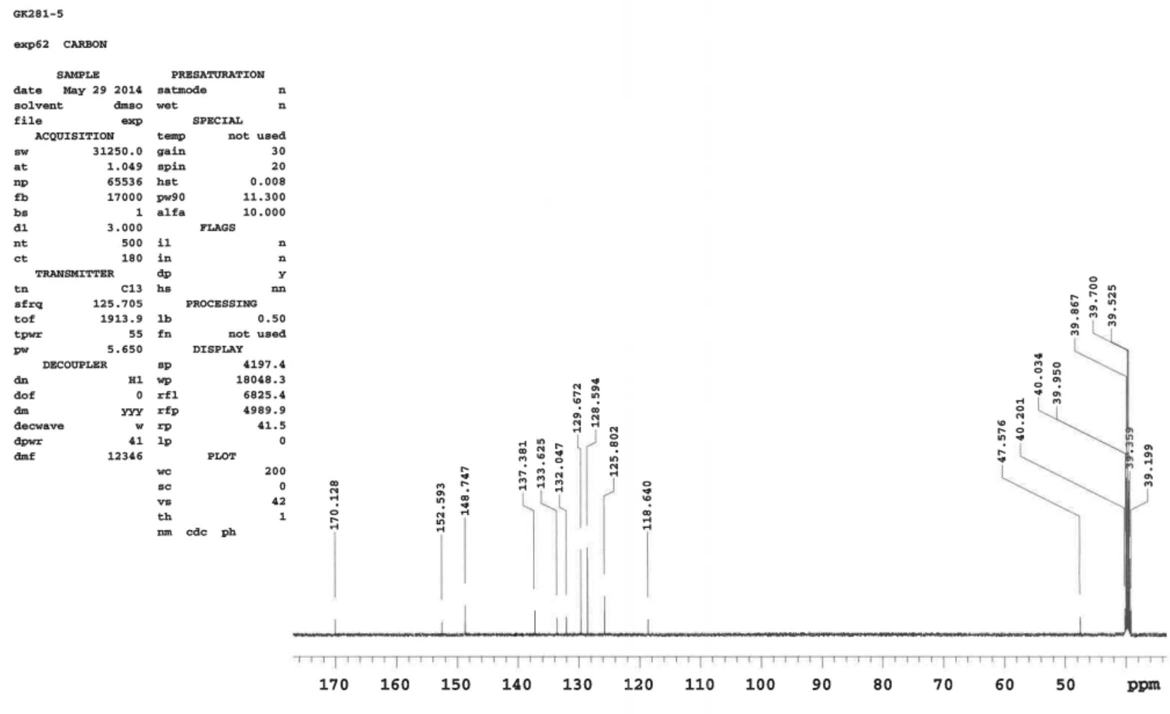

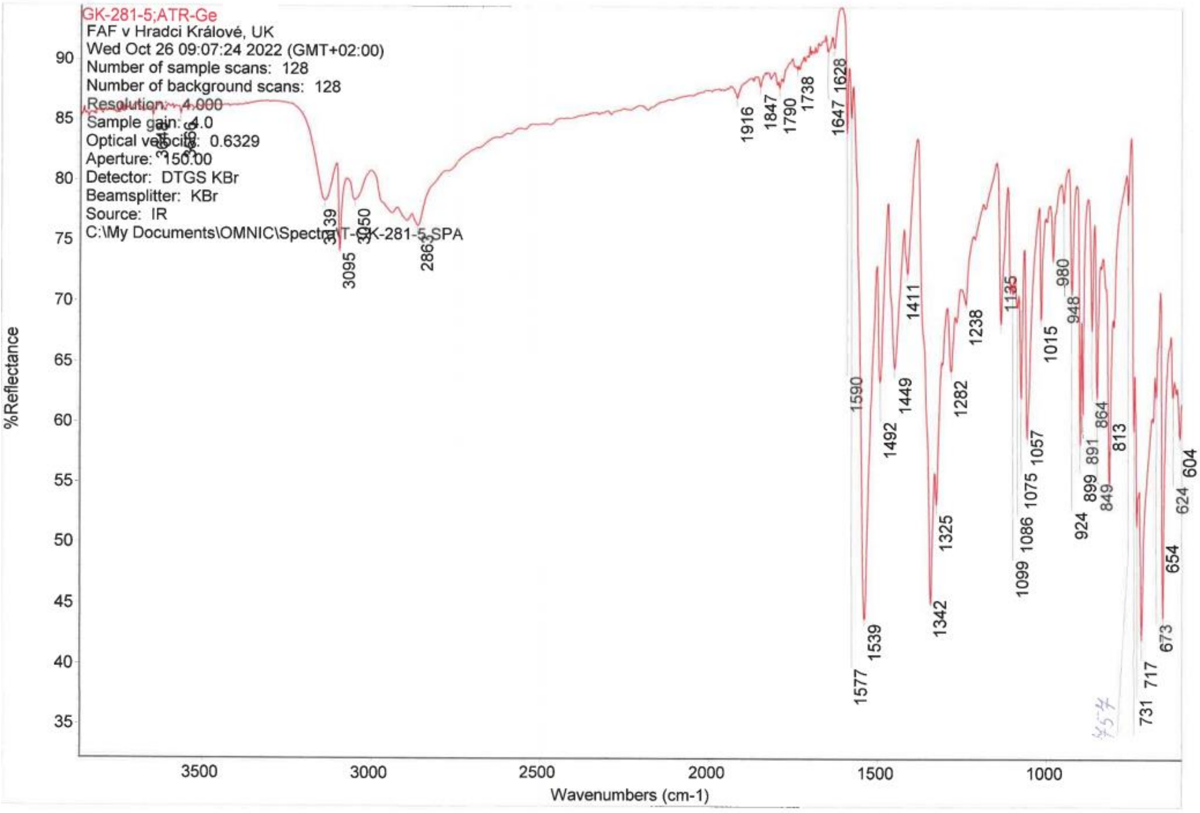

N-(2,4-Dichlorobenzyl)-5-(3,5-dinitrophenyl)-1,3,4-thiadiazol-2-amine **8d**

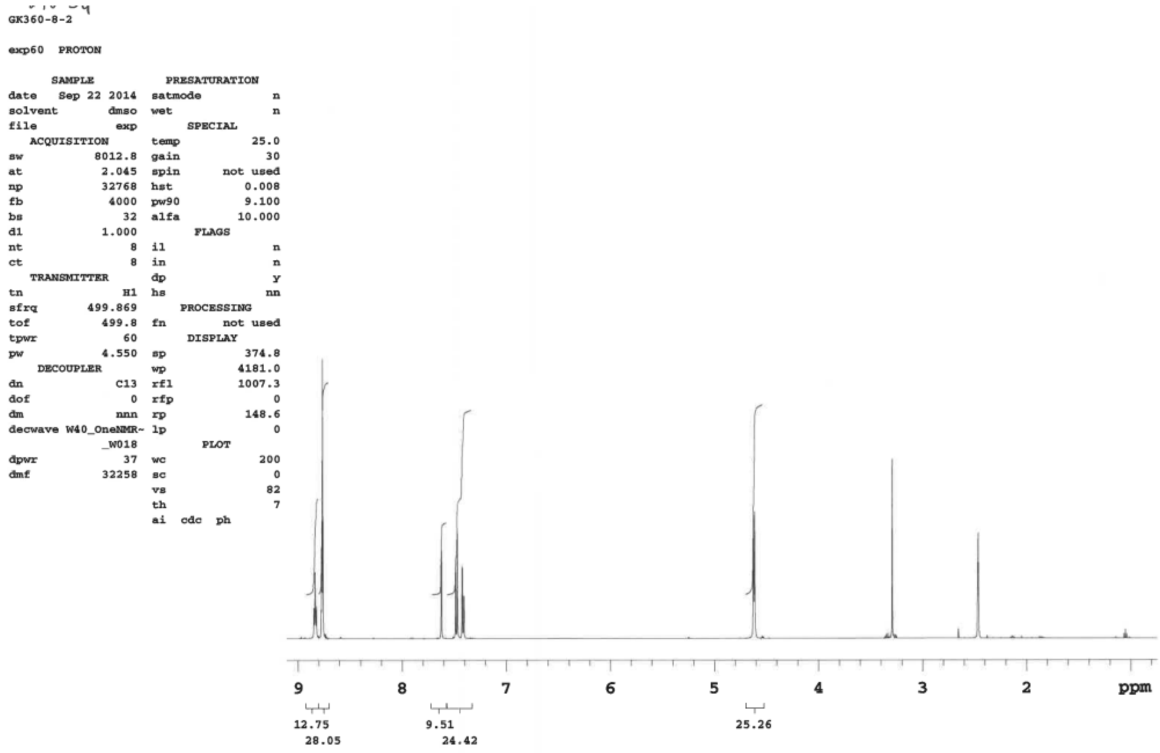

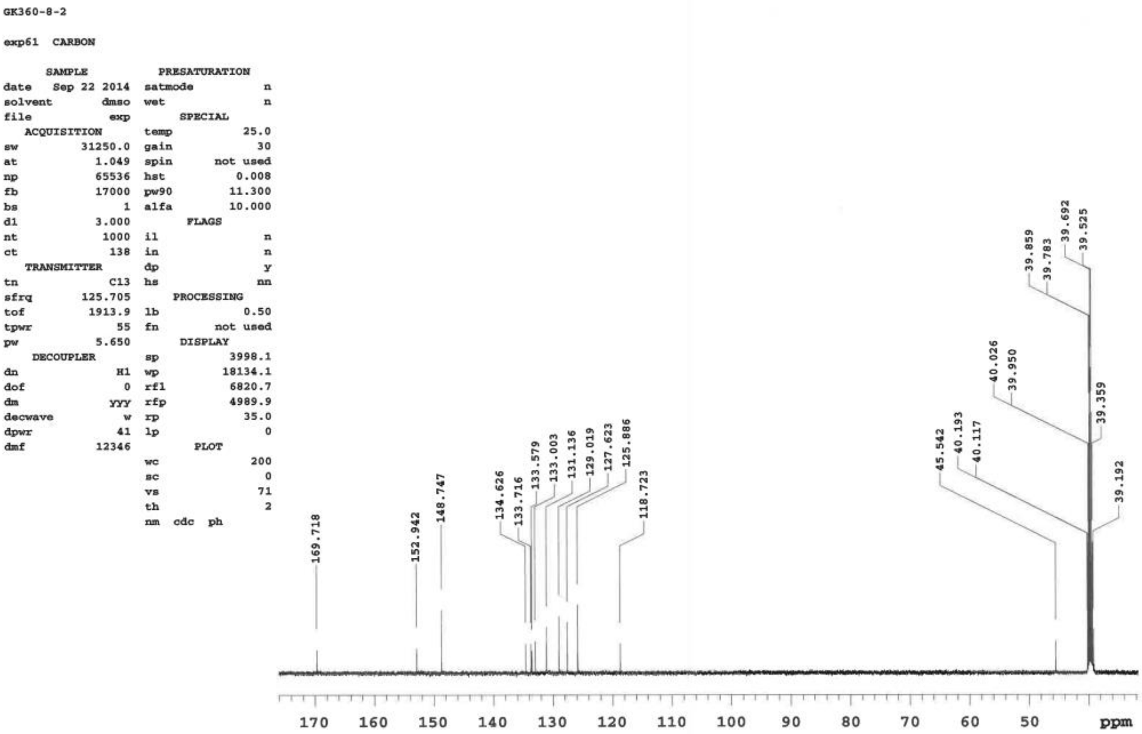
GK360-8-2
exp61 CARBON
SAMPLE
date Sep 22 2014
solvent dmso
file exp
ACQUISITION
sw 31250.0
at 1.049
np 65536
fb 17000
bs 1
d1 3.000
nt 1000
ct 138
TRANSMITTER
tn C13
sfrq 125.705
tof 1913.9
tpwr 55
pw 5.650
DECOUPLER
dn H1
dof 0
dm yyy
decwave w
dpwr 41
dmf 12346
PRESATURATION
satmode n
wet n
SPECIAL
temp 25.0
gain 30
spin not used
hst 0.008
pw90 11.300
alfa 10.000
FLAGS
il n
in n
dp y
hs nn
PROCESSING
lb 0.50
fn not used
DISPLAY
sp 3998.1
wp 18134.1
rfl 6820.7
rfp 4989.9
rp 35.0
lp 0
PLOT
wc 200
sc 0
vs 71
th 2
nm cdc ph
169.718
152.942
148.747
134.626
133.716
133.579
133.003
131.136
129.019
127.623
125.886
118.723
45.542
40.193
40.117
40.026
39.950
39.859
39.783
39.692
39.525
39.359
39.192
170 160 150 140 130 120 110 100 90 80 70 60 50 ppm

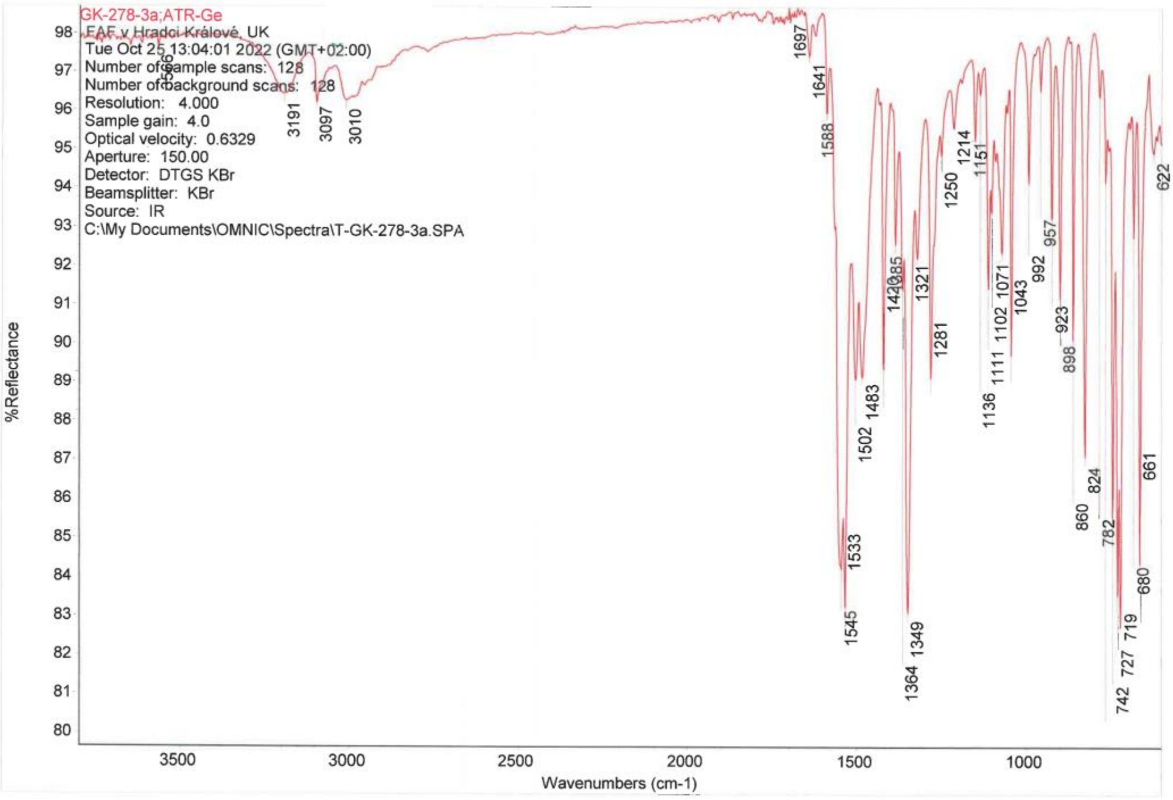
GK-278-3a;ATR-Ge
FAF v Hradci Králové, UK
Tue Oct 25 13:04:01 2022 (GMT+02:00)
Number of sample scans: 128
Number of background scans: 128
Resolution: 4.000
Sample gain: 4.0
Optical velocity: 0.6329
Aperture: 150.00
Detector: DTGS KBr
Beamsplitter: KBr
Source: IR
C:\My Documents\OMNIC\Spectra\T-GK-278-3a.SPA
3191
3097
3010
1697
1641
1588
1545
1533
1502
1483
1420
1385
1364
1349
1321
1281
1250
1214
1151
1136
1111
1102
1071
1043
992
957
923
898
860
824
782
742
727
719
680
661
622
%Reflectance
98 97 96 95 94 93 92 91 90 89 88 87 86 85 84 83 82 81 80
3500 3000 2500 2000 1500 1000
Wavenumbers (cm-1)

2-(3,5-Dinitrophenyl)-5-(dodecylthio)-1,3,4-oxadiazole **10**

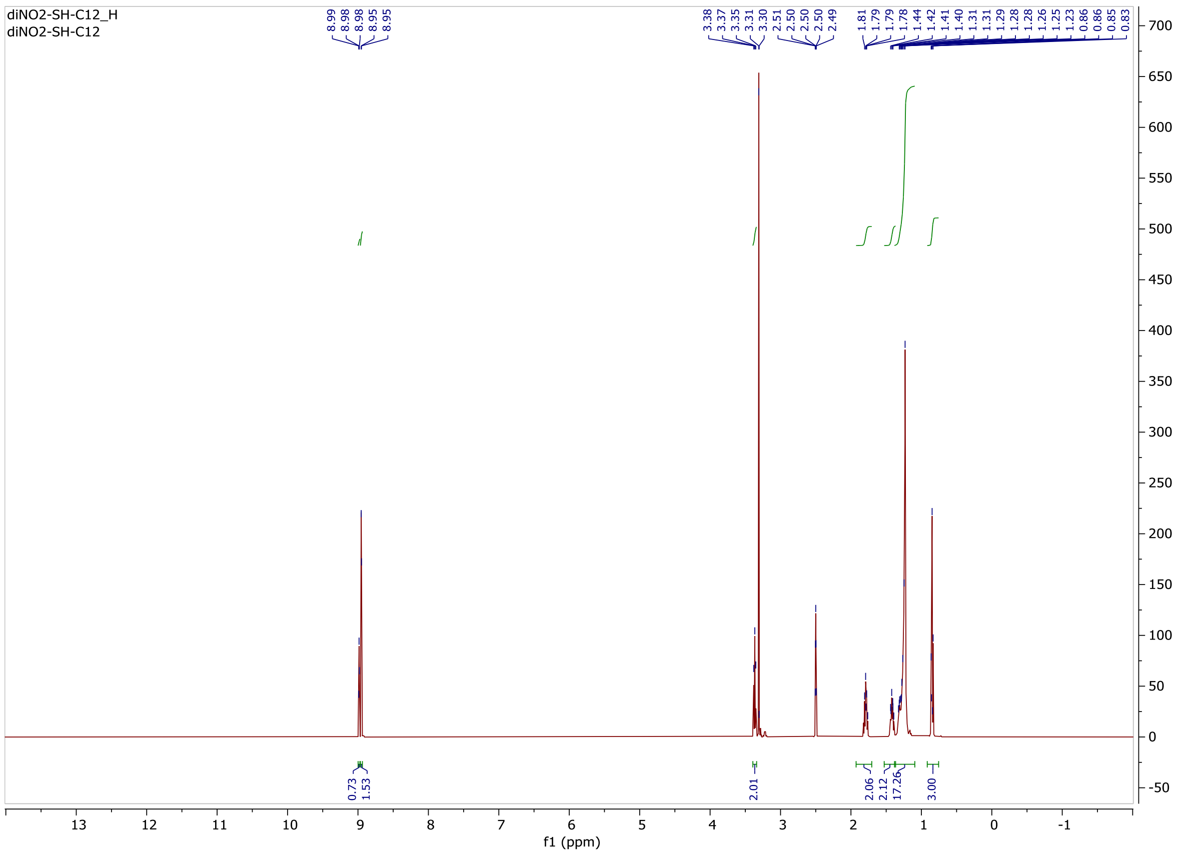

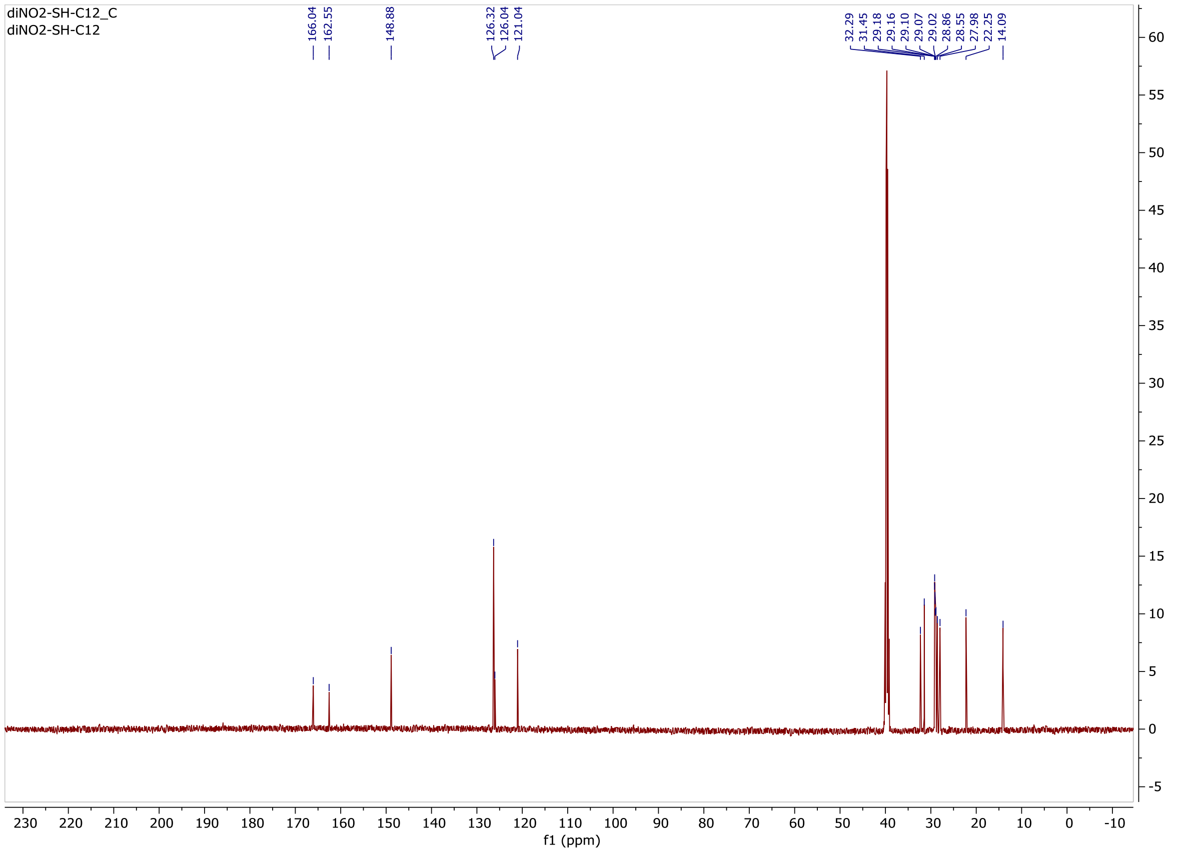

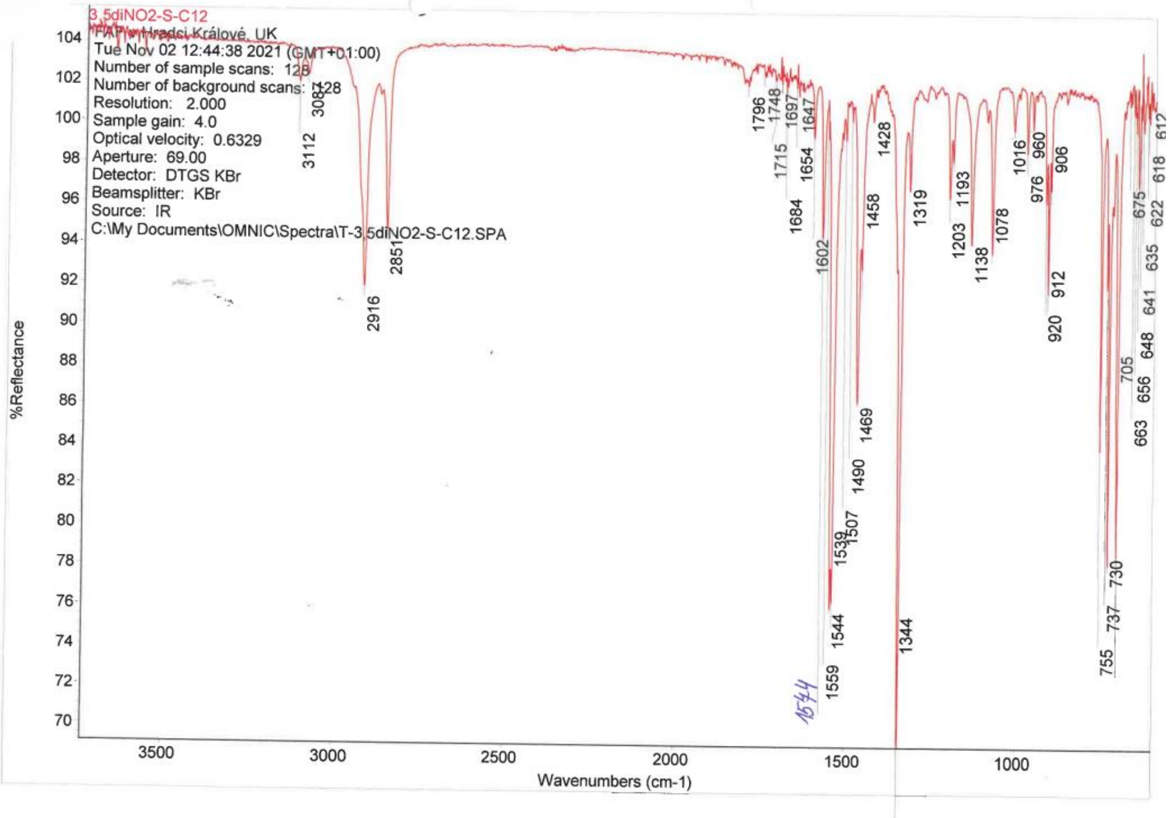
3,5diNO2-S-C12
FAF v Hradci Králové, UK
Tue Nov 02 12:44:38 2021 (GMT+01:00)
Number of sample scans: 128
Number of background scans: 128
Resolution: 2.000
Sample gain: 4.0
Optical velocity: 0.6329
Aperture: 69.00
Detector: DTGS KBr
Beamsplitter: KBr
Source: IR
C:\My Documents\OMNIC\Spectra\T-3,5diNO2-S-C12.SPA
%Reflectance
Wavenumbers (cm-1)
3112
3084
2916
2851
1796
1748
1715
1697
1684
1654
1647
1602
1574
1559
1544
1539
1507
1490
1469
1458
1428
1344
1319
1203
1193
1138
1078
1016
976
960
920
912
906
755
737
730
705
675
663
656
648
641
635
622
618
612
