# Supplementary figures and images for: 5-(3,5-Dinitrophenyl)-1,3,4-oxadiazol-2-amine derivatives, their precursors, and analogues: Synthesis and evaluation of novel highly potent antitubercular agent
Source: PLoS One. 2025 May 29;20(5):e0324608. doi: 10.1371/journal.pone.0324608 (PMC12121777; doi:10.1371/journal.pone.0324608)

X X X X X X

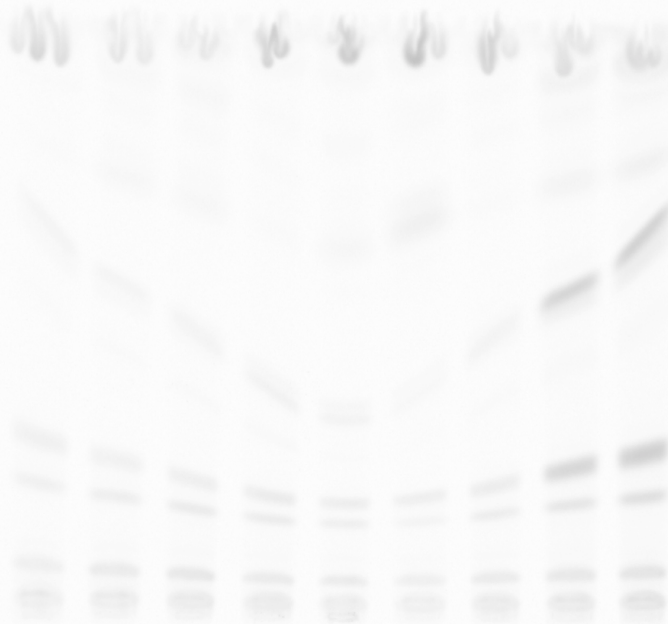

X X X X X X

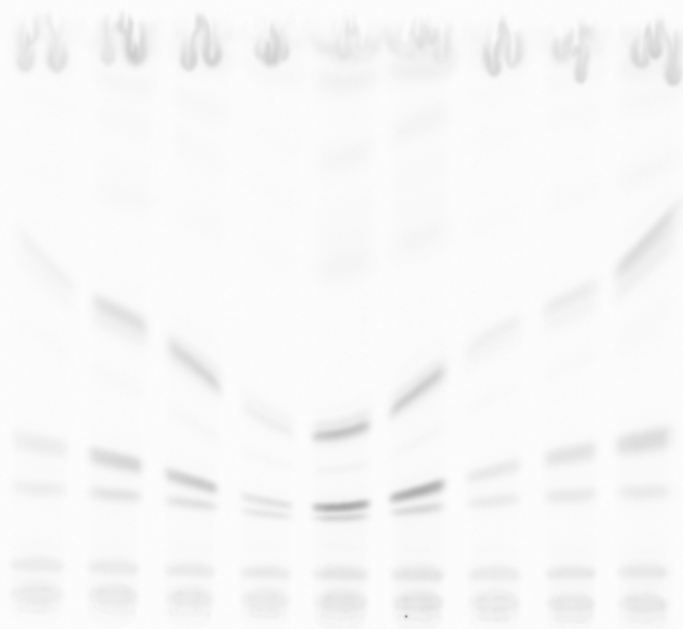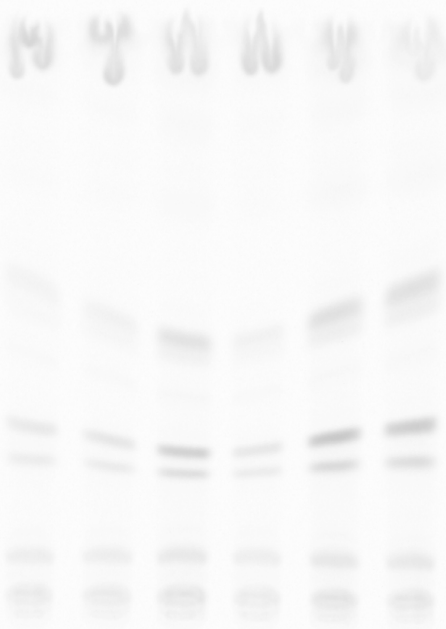

Supplement: S2 raw image — (PDF) [file pone.0324608.s002.pdf]
